# Assessing the genetic diversity of Cu resistance in mine tailings through high-throughput recovery of full-length *copA* genes

**DOI:** 10.1038/srep13258

**Published:** 2015-08-19

**Authors:** Xiaofang Li, Yong-Guan Zhu, Babak Shaban, Timothy J. C. Bruxner, Philip L. Bond, Longbin Huang

**Affiliations:** 1 Centre for Mined Land Rehabilitation, Sustainable Minerals Institute, The University of Queensland, QLD 4072, Australia; 2 Institute of Urban Environment, Chinese Academy of Sciences, Xiamen, 10081, China; 3 Australian Genomics Research Facility, Parkville, Melbourne 3000, Australia; 4 Institute for Molecular Bioscience, The University of Queensland, QLD 4072, Australia; 5 Advanced Water Management Centre, The University of Queensland, QLD 4072, Australia

## Abstract

Characterizing the genetic diversity of microbial copper (Cu) resistance at the community level remains challenging, mainly due to the polymorphism of the core functional gene *copA*. In this study, a local BLASTN method using a *copA* database built in this study was developed to recover full-length putative *copA* sequences from an assembled tailings metagenome; these sequences were then screened for potentially functioning CopA using conserved metal-binding motifs, inferred by evolutionary trace analysis of CopA sequences from known Cu resistant microorganisms. In total, 99 putative *copA* sequences were recovered from the tailings metagenome, out of which 70 were found with high potential to be functioning in Cu resistance. Phylogenetic analysis of selected *copA* sequences detected in the tailings metagenome showed that topology of the *copA* phylogeny is largely congruent with that of the 16S-based phylogeny of the tailings microbial community obtained in our previous study, indicating that the development of *copA* diversity in the tailings might be mainly through vertical descent with few lateral gene transfer events. The method established here can be used to explore *copA* (and potentially other metal resistance genes) diversity in any metagenome and has the potential to exhaust the full-length gene sequences for downstream analyses.

Novel metal resistance genes in the environment, particularly mining-impacted soils, are valuable resources for industrial biomining^1^ and soil remediation^2^. *copA* is one of the core determinants for microbial resistance to Cu and its diversity has been examined in soils in a limited number of recent studies^3^,^4^,^5^,^6^,^7^. These studies are all amplicon-based and rely on the availability of degenerate primers to target conservative regions of *copA*. Unfortunately, available *copA* sequences in the literature are thought to be highly polymorphic and therefore available primers only cover a subset of *copA* sharing high similarity. This hinders the assessment of *copA* diversity and the discovery of novel *copA* in the environment. To overcome this difficulty, we report a method to recover full-length *copA* from a tailings metagenome by combining the methods of metagenome assembly, local BLASTN and evolutionary trace (ET) analysis. The metagenomic strategy has been successfully applied to annotate Cu resistance genes in an activated sludge metagenome^8^, and ET analysis can be used to check the reliability of candidate *copA* genes detected in a metagenome by screening for key conserved domains of their CopA proteins.

*copA* as a part of *cop* system and its homolog gene *pcoA* as a part of *pco* system were first identified in copper resistant strains of *Pseudomonas syringae* (PscopA) and *Escherichia coli* (EcpcoA), respectively. PscopA and EcpcoA were both plasmid-borne but many *copA* homologs were identified soon after, both chromosomal- (e.g. *Enterococcus hirae*) and plasmid-borne (e.g. *Xanthomonas* sp.). Interestingly, many chromosomal *copA* were found to be typical P-type ATPase^9^, while *pcoA* was then identified as multicopper oxidase^10^. Therefore, *copA* is thought to be highly polymorphic^3^,^5^ and the nature of *copA* has always been described as species-dependent in the literature^11^,^12^,^13^,^14^. This caused confusion when studying *copA* diversity in the environment. It is known that multicopper oxidase and ATPase are different protein families using different energy sources^15^,^16^. We thus hypothesize that all *copA* named based on sequence similarity in the literature are not homologs and can be divided into two groups encoding for multicopper oxidase and P-type ATPase, which are both highly conserved.

ET analysis is a method to extract functionally important residues from sequence conservation patterns in homologs, with the assumption that active site residues of a protein family are more conserved during its evolutionary history^17^. While complete or partial crystal structure of some CopA proteins has been available in the literature^18^,^19^,^20^,^21^, it is feasible now to identify the common active sites which may be highly conserved among CopA proteins. Therefore, ET analysis is possible to reveal the underlying root of the polymorphism of *copA*/CopA.

A metagenome obtained by high-throughput sequencing of environmental DNA conceptually provides all the gene resources in a given environment^22^. Metagenomic sequencing successfully overcomes the difficulties facing traditional isolation-based and conventional amplicon-based molecular methods in recovering genetic information from the environment. By means of bioinformatic tools (e.g. MIRA)^23^ which assemble the shorts reads in metagenomes into longer contigs or even nearly complete genomes, metagenomics allow us to explore a functional gene of interest in a high-throughput fashion^24^. BLAST is the most commonly used tool to find homologous sequences based on sequence similarity for functional attributes^25^. By building a local database of antibiotic resistance genes (ARGs), a local BLASTN procedure has been established to screen for the presence of ARGs in bacterial genomes^26^; yet this local-BLASTN method has not been applied to metal resistance genes or to explore metagenomes.

Therefore, this study aimed to develop a method for the recovery of full-length *copA* sequences in a tailings metagenome. A metagenomic library was obtained by assembling 7 individual tailings metagenomes using MIRA. The library was searched using the embedded BLASTN method in BioEdit against a local *copA* database built in this study, followed by recovering full-length *copA* by annotating the contigs containing putative *copA*. All identified putative *copA* were then examined for the presence of highly conserved metal-binding motifs found by ET analysis.

## Results and Discussion

In generating the metagenomes, the MiSeq sequencing yielded >3.9 billion bp and >14.8 million reads after quality control for the 7 tailings samples. The 7 individual tailings metagenomes were pooled together for high-quality assembly and subsequent *copA* gene recalling. In total, 8,566,357 reads (greater than 300 bp) were used for metagenomic assembly which generated 82,334 contigs with an N_50_ of 1,700 bp and a longest contig of 123,516 bp. The data amount used for assembly in this study is much higher than earlier studies^27^,^28^,^29^ and comparable to recent studies^30^,^31^,^32^ where the characterized soil microbial communities have much higher complexity. This allows a deeper coverage of the functional gene diversity in the tailings microbial communities. Moreover, though metagenomes of microbial communities in acid mine drainage have been well studied^33^,^34^, this is, to our knowledge, the first report on microbial metagenomes from neutral mine tailings which more closely resemble natural soils than acidic tailings. While natural soil always harbors a microbial community with a complexity whose fine decoding is still beyond the capacity of current bioinformatics tools^35^, the tailings microbial metagenomes of lower diversity and community structure complexity characterized here may be a useful proxy for studying soil ecosystem functioning.

The Mt Isa Cu-Pb-Zn tailings are neutral and saline (EC > 2 mS/cm; in the dry season it is much higher as found in our previous study^36^) substrates and contain an average Cu concentration more than 16-fold greater than the background soil values^37^ (Table 1). In addition to the high levels of salinity and Cu, the tailings are also high in total Pb and Zn concentrations. The saline and metal stresses may have exerted a strong selective pressure on the microorganisms within the system, as indicated by the extremely low microbial biomass (Table 1) and the microbial diversity, which is dominated by either thermophiles or halophiles as found in our previous studies^38^,^39^. The dominance of extremophiles may explain the high GC contents (all > 60%) of the metagenome libraries^40^.

**Figure 1:**
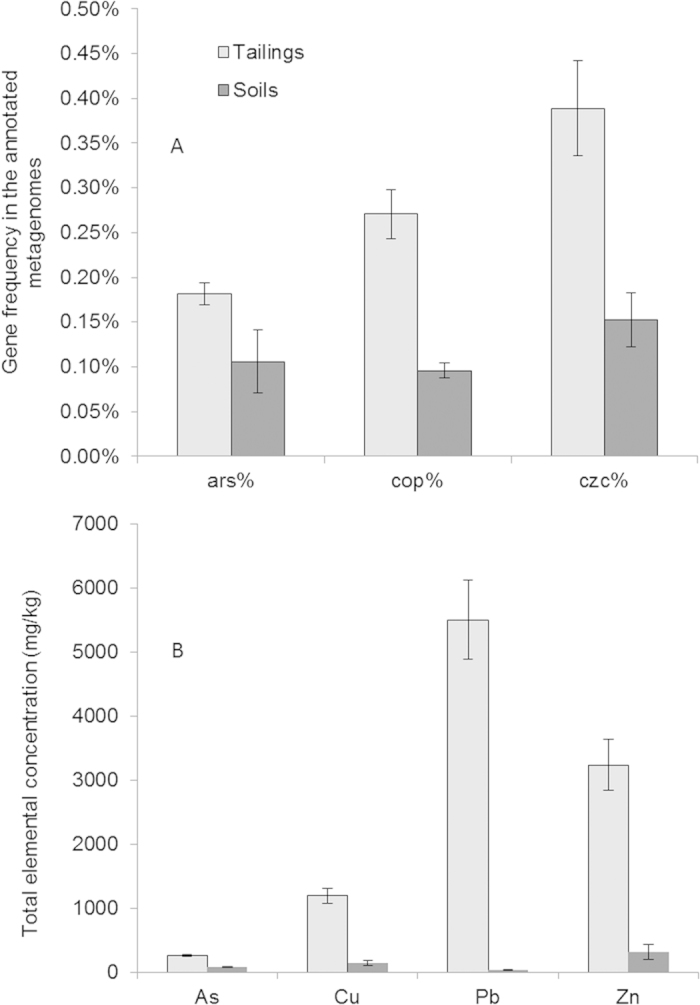
Total elemental concentrations of As, Cu, Pb and Zn in the tailings samples (n = 7) and a reference soil (n = 3) used in this study (**B**) and the corresponding abundances of resistance genes for these metals in the metagenomes from the tailings/soil samples (**A**). *ars*, resistance genes for As; *cop*, resistance genes for Cu; *czc*, resistance genes for multi-metals (e.g., Co, Pb, Zn).

The strong selective effects of metal stresses were also reflected by the significantly high abundance of resistance genes for heavy metals in the metagenomes, based on the annotations by MG-RAST pipeline. In comparison with a local soil close to the tailings site sampled, the tailings metagenomes contain average *cop*, *czc* (coding for multiple metal resistance) and *ars* (coding for arsenic resistance) gene abundances of 2.8, 2.5 and 1.7 folds, respectively, of those of the soil (unpublished results; the metagenome data will be released soon in MG-RAST). The heavy metals in the soil are close to background crustal values (Fig. 1). Increased levels of toxins can lead to the enrichment of relevant resistance genes, as reported for both antibiotic and heavy metal resistance genes^41^,^42^,^43^,^44^,^45^. Enrichment of *copA* has also been reported in various metal-contaminated environments such as paddy soil^3^, arable soil^14^ and sediment^46^. However, to our knowledge, statistical results from shotgun metagenomic sequencing for *cop* genes have not been reported until recently^8^. Considering the polymorphism of *cop* systems, the results in this study may cover a deeper diversity of Cu resistance genes than those which are amplicon-based and rely on the specificity of primers.

A *copA* dataset containing 122 sequences annotated as *copA* obtained from full genomes available in Genbank has been built in this study. The abundance of *copA* selected from each phylum generally reflects the abundance of the available *copA* for that phylum in Genbank. Specification tests of the local BLASTN were done using *copA* sequences from the local database as well as full genomes containing putative *copA*. For the *copA* sequences from the database, the method was able to unambiguously identify all these genes with sensitivity and specificity at 100% and full coverage; at an elevated e-value, fragmented sequences of identity >80% can be found. At an e-value of 10^−4^, we were also able to locate *copA*-like genes in the three genomes of *Acidithiobacillus ferrooxidans*, *Rubrobacter radiotolerans* and *Thioalkalivibrio sulfidophilus*, who are phylogenetically close to those of abundant species in the tailings, with more than 10 hits with an identity >80% and an alignment length >40 bp (the cutoff used for subsequent contig annotation in this study). Additionally, 6 hits were returned with an identity >86% and alignment length >40 bp when the BLASTN was applied to the genome of *Thermodesulfobacterium geofontis*. These results demonstrate a good coverage of *copA* diversity in the database and the tool established here is theoretically able to cover the novel *copA* diversity in our tailings metagenome.

In total, 99 full-length putative *copA* genes were recovered from the tailings metagenome. All of these genes had high similarity (>70%) to *copA* sequences from the genomes in Genbank. As the local BLASTN method is similarity-based, the number of *copA* genes identified can vary largely upon how novel the subjected microbial gene pool is and the diversity of the *copA* database. The threshold of alignment length in local BLASTN results was arbitrarily set as 40 bp to limit the number of contigs subject for subsequent *copA* gene annotation. However, the number of *copA* sequences recovered can be increased if the threshold is reduced, since Gupta *et al.*^26^ found a minimum alignment length of 17 bp for putative new genes using a similar method for ARGs. Considering that the tailings microbial communities in this study are fairly low in species diversity, the local BLASTN method we have established has the potential to exhaust the *copA* diversity therein and can also be a novel tool to explore *copA* diversity in more complex soil environments.

**Figure 2:**
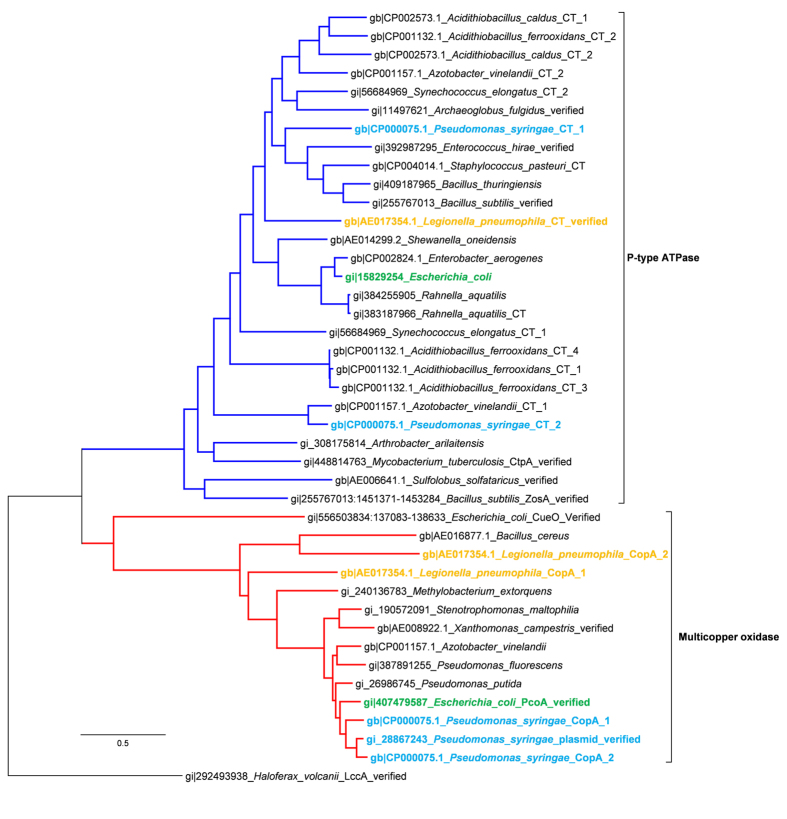
A phylogenetic tree based on the alignment of the CopA and reference protein sequences used for ET analysis in this study. Microbial species containing CopA sequences from both the P-type ATPase and Multicopper oxidase groups are highlighted in colour. When the Cu resistance function has been verified this is mentioned for those taxa.

Annotation of new genes is mostly sequence-similarity-based^47^, including the early studies detecting *copA* homologs. While both multicopper oxidase-like and P-type ATPase-like *copA* have been referred to in the early literature^9^,^48^, more recent studies state these as two “types” of *copA*^14^. However, we propose that the so-called two types of *copA* are not homologs and are incorrectly associated due to the limited number of available sequences used for similarity comparison in early studies. A wide range of protein sequences was used for phylogenetic analysis in this study. These were sequences that were verified experimentally or annotated as CopA and CopA-like Cu translocating proteins obtained from reported Cu resistant microorganisms. The sequences clearly separated into two distinct groups, one containing typical multicopper oxidases CueO and the other containing the typical P-type ATPase CtpA and ZosA (Fig. 2; Table 2). The former group includes the protein products of *copA* genes detected in early studies from the plasmids of *P. syringae* and *E. coli*., and the latter group includes P-type ATPase Cu translocating proteins from *A. fulgidus*, *E. hirae* and *L. pneumophila* whose crystal structure has been resolved. These two groups of CopA may have distinct roles in Cu resistance, as implied by the experimental assays *in vitro* of typical CopA. Members of multicopper oxidase group CopA have been reported to be able to oxidize substrates of laccase, like phenol^10^,^49^, while members of ATPase CopA have been found to bind and transport Cu(I)^19^,^50^,^51^. Meanwhile, in the *E. coli* genome, the model microorganism for *cop* system studies, both groups of *copA* are present and their roles are found to be different. Plasmid-borne *pcoA* functions synergistically with chromosomal *copA*, and the protein PcoA can oxidise Cu(I) carried by PcoC and substitute the role of chromosomal CueO^10^,^52^,^53^. Our analysis here indicated that many model species for *copA* studies contain both groups of *copA*, such as *E. hirae* and *P. syringae* (Fig. 2). The different roles of the two groups of *copA* may also be implied by their different sequence and tertiary structure. Multicopper oxidase CopA has no crystal structure resolved so far but prediction by SWISS-MODEL^54^ showed that EcPcoA and PsCopA have similar crystal structure with laccase, which is distinct from the transmembrane figuration of the ATPase CopA detected in *A. fulgidus* and *L. pneumophila*^16^,^19^,^50^,^55^. ET analysis indicated that the two groups of CopA have different metal binding motifs (Supplementary Figure 1). The ATPase group typically uses Cys as a metal binding residue in the motifs of CXXC (with HXXH as a variant) and CXC (the typical transmembrane metal binding motif), while the multicopper group uses His for metal binding in the form of HXH which is highly conservative within the group. Taken together, we suggest that CopA homologous to PcoA should be named as PcoA which is a multicopper oxidase, to differentiate it from ATPase CopA. Consequently, the metal binding motifs detected here can be used to screen for *copA* candidates with a high potential of functioning in Cu resistance.

The 99 full-length putative *copA* sequences were translated and aligned with the known CopA used for ET analysis. The presence of metal binding motifs abovementioned was screened in the putative CopA; For ATPase like CopA, the ATP binding motifs (GDGIN) were also used for screening. In total, 70 putative CopA were thought to have a high chance of functioning in Cu resistance.

**Figure 3:**
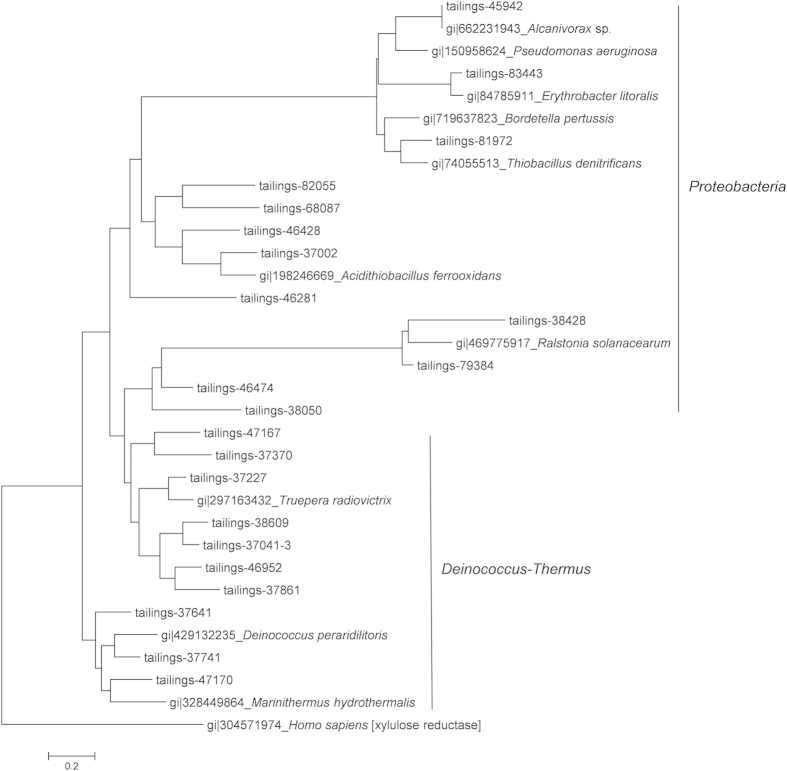
A phylogenetic tree showing the representative *copA* sequences affiliated with *Proteobacteria* and *Deinococcus-Thermus* recovered from the tailings metagenome after assembly.

Phylogenetic analysis was performed on the CopA sequences affiliated with the dominant species detected in the tailings (Fig. 3). The topology of the tree is largely consistent with that of the 16S rRNA gene based phylogeny. This indicates that the *copA* gene diversity was mainly controlled by vertical descent in the history of tailings community evolution, at least among the dominant species. Similar conclusions have been drawn for *merA* genes responsible for mercury resistance^2^. P-type ATPase metal homeostasis genes are probably ancient genes and have been essential for microbial survival, and thus lateral gene transfer plays a minor role in their evolution^56^.

It is worth noting that the 29 putative *copA* sequences with no metal binding or phosphatase binding domains found may also function for Cu-resistance. For one thing, it is possible the combined presence of all the metal binding motifs found here are not essential for Cu resistance. Studies on EcCopA have found that the metal binding domain C^479^PC^481^ motif is essential for Cu resistance but the other two C^14^XXC^17^ and C^110^XXC^113^ are not^57^,^58^. Meanwhile, some Cu resistance ability may have evolved with sequences containing alternative metal binding domains. For example, in the alignment of ATPase group CopA, about half of the members lack the C^84^XXC^87^ motif; and CopA of *Sulfolobus solfataricus*, in which the Cu resistance function has been experimentally verified, uses YPC instead of CPC as a transmembrane metal binding domain (Supplementary Figure 1). Furthermore, it must be highlighted that the CopA proteins are relatively divergent in terms of both sequence composition and length. Inaccurate alignments can be made and lead to failure for detecting motifs when the sequences are too divergent^59^. Therefore, the accuracy of alignment methods may largely determine the ability to detect the metal binding motifs of the subject sequences. For example, the alignment method used in this study failed to align CueO of *E. coli* with the others, and manual adjustments were required to locate the three experimentally-verified metal binding domains^60^.

## Conclusions

A local BLASTN method was established in this study to recover full-length *copA* genes in an assembled tailings metagenome. The detected *copA* genes were further screened for potentially functioning *copA* by screening active sites in their encoding proteins, which were inferred by ET analysis using known CopA. The method established here can be used to recover full-length *copA* (and potentially other resistance genes) in any assembled metagenomes.

## Materials and Methods

### Sampling and DNA extraction

Tailings samples were sampled in June in 2013 from a field trial site located at Mount Isa (Mt Isa) tailings impoundment. Mt Isa (20.73 °S, 139.5 °E) is located in northwest Queensland, Australia. Mt Isa has a semi-arid climate with an annual pan evaporation of 2800 mm and an average rainfall of 400 mm, with the vast majority of the rain falling during the wet season (November to February). The tailings used for field trial were highly weathered and collected from a tailings storage facility (TD5) that contained mixed streams of Cu and Pb-Zn tailings and was decommissioned about 40 years ago, which had received streams of Cu and Pb-Zn tailings for decades. The details of tailings properties and treatment setup can be found in our previous studies^38^,^39^. Briefly, the tailings mainly consist of quartz, dolomite, pyrite, gypsum and kaolinite, and contain 0.13 ± 0.03% of total Cu, 0.18 ± 0.02% of total Pb, and 0.29 ± 0.01% of total Zn. A field revegetation trial was established in 2010, and within 3 years the woodchips amendment and/or revegetation treatments did not substantially change the tailings physiochemical properties and the dominant microbial species. Dominant microbial species were affiliated with *Rubrobacter* spp. of *Actinobacteria*, *Truepera* spp. of *Deinococcus-Thermus* and *Thioalkalivibrio* spp. and *Thiobacillus spp.* of *Proteobacteria*^39^,^61^.

Tailings sample were stored in an iced container and shipped to the laboratory within 24 hours for DNA extraction. For physiochemical analyses part of the tailings were oven-dried at 40 °C, sieved through a 2 mm screen and mixed thoroughly before use. Methods for physiochemical analyses, such as electrical conductivity (EC), cation exchange capacity (CEC), total organic carbon (TOC), microbial biomass carbon (MBC) and total elemental concentrations (Table 1), can be found in our previous studies^39^,^61^.

DNA was extracted from 24 tailings samples (8 plots with 3 replicates each plot). DNA extraction was done using commercial kits after cell enrichment by sucrose density centrifugation; detailed methods can be found in our previous studies^39^,^61^. For MiSeq shotgun sequencing, replicates were combined to obtain enough DNA, and one sample of pure tailings failed the quality test for sequencing. Therefore, 7 independent tailings DNA samples representing the gene pool in the tailings landscape were sequenced using Illumina MiSeq platform in this study. The DNA concentrations and quality were measured using the Qubit^®^ 2.0 Fluorometer (Thermo Fisher Scientific). DNA concentrations of the 7 samples ranged from 4.5 to 25.2 ng/μl, giving between 0.23 to 1.51 μg of DNA to use for the sequencing.

### MiSeq sequencing

DNA sequencing libraries were prepared using the Illumina TruSeq DNA LT Sample Prep Kit (Illumina), following the standard manufacturer’s protocol (Part #15026486 Rev. C July 2012) with modifications as described below. Genomic DNA was fragmented by sonication (Covaris S2) using the “Whole-genome Resequencing” settings in the protocol, after which the DNA was purified using AMPure XP beads. The purified, fragmented DNA was end repaired and again purified using AMPure XP beads. The purified, end repaired DNA was size-selected using a double-SPRI method to obtain insert sizes of approximately 600 bp (actual average insert size ranges from 456–667 bp). The size-selected DNA was A-tailed and then had adapters ligated, followed by an AMPure XP purification. This ligated product was amplified by PCR to produce the final library. The final individual libraries were visualized and quantified on the Agilent BioAnalyzer 2100 using the High Sensitivity DNA Kit. The libraries were then pooled in an equimolar ratio, and the pool was quantified by qPCR using the KAPA Illumina Library Quantification Kit (KAPA Biosystems). The library pool was sequenced on the Illumina MiSeq (MiSeq control software v2.0.5/Real Time Analysis 1.18), using the MiSeq Reagent Kit v3 (600-cycle) with paired-end 300 bp reads.

### Shotgun metagenomic analyses

For functional annotations, the paired-end raw data was uploaded to MG-RAST server (Rapid Annotation using Subsystems Technologies for Metagenomes)^62^. Sequences were annotated to functional categories against M5NR database using BLASTX at an e-value cutoff of 1 × 10^−5^. Results of general descriptors and annotations were downloaded from MR-RAST server for download analyses. Gene abundance was counted manually for *cop*, *czc* and *ars* genes, which are responsible for Cu, Pb/Zn and As resistance, respectively. For metagenomic assembly, the raw reads were trimmed using Skewer^63^ to have a Q > 25 and a minimum length of 200 bp. Quality trimmed reads were used for *de-novo* assembly using the open source software package Mira (4.0rc5)^23^ on a 24 core, 192 GB server with multithreading enabled. The seven tailings libraries were combined to improve the quality of assembly^64^. The contig dataset was then subjected to local BLASTN for searching of *copA*-like genes.

### Local BLASTN

A local BLASTN method, similar to ARGAnnot by Gupta *et al.*^26^, was established to recall *copA*-like genes in the assembled metagenomic database. Briefly, nucleotide sequences annotated as *copA* in Genbank were retrieved manually and formed a local *copA* database (Supplementary Material 1). Then the assembled metagenomic dataset was aligned against the *copA* database using the embedded BLASTN method in BioEdit^65^. Then the results were sorted based on aligned length and a threshold of 40 bp was used for screening *copA*-like genes in the metagenomic dataset. All the contigs containing candidate *copA* were picked out manually and then subjected to gene finding using Glimmer^66^. For *copA*-like gene finding, the selected contigs were aligned against the Genbank database using BLASTN one-by-one and the sequence region annotated as *copA* or heavy metal resistance genes were compared with the open reader framework (ORF) found by Glimmer. The candidate ORF was then manually curated again against the Genbank database. The closest *copA* sequences were also retrieved for phylogenetic analysis. Maximum-likelihood phylogenetic trees were constructed for all the *copA* sequences in the database and selected found *copA* sequences from the tailings metagenome, using the method described below.

Local BLASTN against the *copA* database was done on a Window 7 computer equipped with dual-core 2.8 GHz CPU and 8 G RAM. The assembled dataset was cut into 6 sub-datasets and BLASTN for each dataset used up to 6 computing hours.

Specificity tests were done for the local BLASTN method using 1) 4 *copA* sequences included in the database (gi_294009986_*Sphingobium japonicum*; gi_258541105_*Acetobacter pasteurianus*; gi_347756788_*Micavibrio aeruginosavorus*; gi_162145846_*Gluconacetobacter diazotrophicus*), 2) three complete genomes (gi_198282148_*A. ferrooxidans*, gi_627776062_*R. radiotolerans*, and gi_220933193_*T. sulfidophilus* who are phylogenetically close to those of abundant species in the tailings) containing *copA* novel but with homologs in the *copA* database and 3) a complete genome (gb_CP002829.1_*T. geofontis*) belonging to phylum *Thermodesulfobacteria* which is not included in the database and probably containing novel *copA*.

### Evolutionary trace analysis

Evolutionary trace was done following the method by Wilkins *et al.*^67^. Since microorganisms harbouring *copA* homologs can also be Cu-sensitive^68^, gene sequences for ET analysis in this study were selected based on three criteria (Table 2): 1) the microorganisms must be reported as Cu-resistant; 2) the full genome and/or complete sequence of plasmid(s) must be available in the Genbank; and 3) the gene must be annotated as *copA* or *copA*-like translocating genes in the full genome. In addition, five CopA protein sequences whose gene functions have been verified experimentally through mutagenesis or whose crystal structure has been resolved were also obtained from UniProt^69^. Information on active sites of these verified CopA was gathered from the literature^10^,^16^,^17^,^18^,^19^,^50^. Information on active sites of plasmid-encoded PsCopA was obtained from the predictions of UniPro since no crystal structure has been resolved so far for this group of CopA, and crystal structure of EcCueO and other multicopper oxidases were used as references. Furthermore, two multicopper oxidases, LccA (laccase) of *Haloferax volcanii* and CueO of *E. coli* for Cu homeostasis, and two P-type ATPases, CtpA of *Mycobacterium smegmatis* for cadmium transport and ZosA of *Bacillus subtilis* for Zn transport, 38 CopA sequences of known or highly possible to have Cu-resistance functions were aligned using the method of MUSCLE^70^ embedded within MEGA 6^71^. The crystal structures of reference proteins, LccA, CueO, CtpA and ZosA, have also been resolved^72^,^73^,^74^,^75^. For active sites comparison, the Cu-binding motifs were searched manually based on the available information of verified proteins said above. For phylogenetic analysis, the alignment in FASTA format was used to determine the conserved regions using Gblocks 0.91 b online^76^. Two highly conserved regions, one at the C-terminal and one at the N-terminal, were found, and the variable beginning and tail parts were trimmed in MEGA 6. The aligned regions of the alignment was then used for the construction of a Maximum-Likelihood tree using default values within MEGA.

### Screening of CopA containing active sites found in ET analysis

All the CopA sequences detected from the tailings metagenome were aligned with the reference CopA (those with verified functions) using the alignment method of MUSCLE, as described above. The active site regions were checked manually based on the alignment for screening of CopA, gaps were manually adjusted to refine the alignment, and those containing the conserved motifs were determined as highly possible to be functioning as P-type ATPase Cu translocating proteins.

## Additional Information

**How to cite this article**: Li, X. *et al.* Assessing the genetic diversity of Cu resistance in mine tailings through high-throughput recovery of full-length *copA* genes. *Sci. Rep.* **5**, 13258; doi: 10.1038/srep13258 (2015).

## Supplementary Material

Supplementary Information

Supplementary Information

## Figures and Tables

**Table 1:** General descriptors of the tailings samples and the metagenomes analysed in this study.

Sample	pH	EC (μS/cm)	CEC (cmol+/kg)	TOC (g/kg)	MBC (mg/kg)	Total Cu (mg/kg)	Total Pb (mg/kg)	Total Zn (mg/kg)	Raw metagenome data size (Mb)	Mean GC content after quality control (%)	Total gene copy annotated	Number of contigs after assembly	Maximum contig size (bp)	N50 of the contigs
Tailings_1	6.70	5,490	30.47	5.55	132.35	1316	5080	3914	706	63 ± 9	2,153,536			
Tailings_2	6.80	5,420	48.30	5.85	92.65	1294	5300	3234	707	62 ± 10	1,046,050			
Tailings_3	6.90	3,860	39.04	5.54	73.64	1285	4700	2620	551	60 ± 11	1,136,742			
Tailings_4	6.90	2,080	42.06	4.95	51.38	1247	5090	3164	551	64 ± 7	1,044,817	82,334	123,516	1,734
Tailings_5	6.70	3,120	15.63	4.43	38.49	1205	6350	2991	544	62 ± 9	1,115,745			
Tailings_6	6.80	3,040	12.19	3.77	28.04	1056	5860	3261	542	62 ± 9	1,265,601			
Tailings_7	6.80	3,390	20.51	3.54	27.78	1143	6140	3485	611	62 ± 9	1,130,746			

Notes: EC, electrical conductivity; CEC, cation exchange capacity; TOC, total organic carbon; MBC, microbial biomass carbon.

**Table 2:** Selected microbial species used for evolutionary trace analysis in this study.

Species	MIC (mM)^a^
*Acidithiobacillus caldus* DSM 9239	24^1^
*Acidithiobacillus ferrooxidans* isolate N39-30-03	800^1^
*Agrobacterium tumefaciens* CCNWRS33-2	>5^77^
*Archaeoglobus fulgidus*	Protein functioning verified^b^^50^
*Arthrobacter arilaitensis* 11J	3.1^78^
*Azotobacter vinelandii* GZC24	4.7^79^
*Bacillus cereus* BC21	3.9^79^
*Bacillus subtilis*	Protein functioning verified^80^
*Bacillus thuringiensis* N2	>5^77^
Cyanobacterium *Synechococcus*	Unknown^c^^81^,^82^
*Enterobacter aerogenes* NTG-01	2^83^
*Enterococcus hirae*	Protein functioning verified^17^
*Escherichia coli* RJ92	400^84^
20^85^	
Protein functioning verified^18^	
*Methylobacterium extorquens*	Unknown^86^
*Pseudomonas fluorestens* 09906	1.6^87^
*Pseudomonas putida* CZ1	>5^77^
*Pseudomonas putida* S4	>1^88^
*Pseudomonas syringue*	1.0–3.2^89^
1.2–2.0^90^	
Protein functioning verified	
*Rahnella aquatilis* MT7	3.9^79^
*Staphylococcus pasteuri* N2	>5^77^
*Stenotrophomonas maltophilia* AAP56	>0.4^91^
*Synechococcus elongatus*	Unknown^92^
*Xanthomonas campestris* BrC2	0.3^93^
Protein functioning verified^49^	
*Haloferax volcanii* LccA	Protein functioning verified^72^
*Escherichia coli* CueO	Protein functioning verified^73^
*Mycobacterium smegmatis* CtpA	Protein functioning verified^74^
*Bacillus subtilis* ZosA	Protein functioning verified^75^

The MIC of Cu, and whether the protein function has been determined is included. Genomes of these species all harbour *copA* or *copA*-like copper translocating genes.

^a^minimum inhibitory concentration as of the strains of the species studied and under the specific test conditions in the corresponding references; ^b^functions of *copA* genes have been experimentally verified or the crystal structure of CopA proteins has been resolved; ^c^referred as Cu resistant but MIC is unknown or not provided.

## References

[b1] Orell A., Navarro C. A., Arancibia R., Mobarec J. C. & Jerez C. A. Life in blue: Copper resistance mechanisms of bacteria and Archaea used in industrial biomining of minerals. Biotechnol Adv 28, 839–848 (2010). 20627124 10.1016/j.biotechadv.2010.07.003

[b2] Boyd E. S. & Barkay T. The mercury resistance operon: from an origin in a geothermal environment to an efficient detoxification machine. Front Microbiol 3, 349 (2012). 23087676 10.3389/fmicb.2012.00349 PMC3466566

[b3] Li X. F., Yin H. B. & Su J. Q. An attempt to quantify Cu-resistant microorganisms in a paddy soil from Jiaxing, China. Pedosphere 22, 201–205 (2012).

[b4] Roosa S. *et al.* Bacterial metal resistance genes and metal bioavailability in contaminated sediments. Environ Pollut 189, 143–151 (2014). 24662000 10.1016/j.envpol.2014.02.031

[b5] Lejon D. P. H. *et al.* Fingerprinting and diversity of bacterial *copA* genes in response to soil types, soil organic status and copper contamination. FEMS Microbiol Ecol 61, 424–437 (2007). 17696885 10.1111/j.1574-6941.2007.00365.x

[b6] De la Iglesia R. *et al.* Novel polymerase chain reaction primers for the specific detection of bacterial copper P-type ATPases gene sequences in environmental isolates and metagenomic DNA. Lett Appl Microbiol 50, 552–562 (2010). 20337927 10.1111/j.1472-765X.2010.02832.x

[b7] Besaury L., Pawlak B. & Quillet L. Expression of copper-resistance genes in microbial communities under copper stress and oxic/anoxic conditions. Environ Sci Pollut Res Int 10.1007/s11356-014-3254-4 (2014). 25009094

[b8] Li L. G., Cai L., Zhang X. X. & Zhang T. Potentially novel copper resistance genes in copper-enriched activated sludge revealed by metagenomic analysis. Appl Microbiol Biotechnol 98, 10255–10266 (2014). 25081552 10.1007/s00253-014-5939-5

[b9] Odermatt A., Suter H., Krapf R. & Solioz M. An ATPase operon involved in copper resistance by *Enterococcus-Hirae*. Ann Ny Acad Sci 671, 484–486 (1992). 1288347 10.1111/j.1749-6632.1992.tb43836.x

[b10] Djoko K. Y., Xiao Z. G. & Wedd A. G. Copper resistance in *E. coli*: The multicopper oxidase PcoA catalyzes oxidation of copper(I) in (CuCuII)-Cu-I-PcoC. Chembiochem 9, 1579–1582 (2008). 18536063 10.1002/cbic.200800100

[b11] Nies D. H. Microbial heavy-metal resistance. Appl Microbiol Biot 51, 730–750 (1999). 10.1007/s002530051457 10422221

[b12] Teixeira E. C., de Oliveira J. C. F., Novo M. T. M. & Bertolini M. C. The copper resistance operon *copAB* from *Xanthomonas axonopodis* pathovar citri: gene inactivation results in copper sensitivity. Microbiol-Sgm 154, 402–412 (2008). 10.1099/mic.0.2007/013821-0 18227244

[b13] Silver S. & Phung L. T. Bacterial heavy metal resistance: New surprises. Annu Rev Microbiol 50, 753–789 (1996). 8905098 10.1146/annurev.micro.50.1.753

[b14] Altimira F. *et al.* Characterization of copper-resistant bacteria and bacterial communities from copper-polluted agricultural soils of central Chile. BMC Microbiol 12, 193 (2012). 22950448 10.1186/1471-2180-12-193 PMC3496636

[b15] Sakurai T. & Kataoka K. Basic and applied features of multicopper oxidases, CueO, bilirubin oxidase, and laccase. Chem Rec 7, 220–229 (2007). 17663447 10.1002/tcr.20125

[b16] Axelsen K. B. & Palmgren M. G. Evolution of substrate specificities in the P-type ATPase superfamily. J Mol Evol 46, 84–101 (1998). 9419228 10.1007/pl00006286

[b17] Lichtarge O., Bourne H. R. & Cohen F. E. An evolutionary trace method defines binding surfaces common to protein families. J Mol Biol 257, 342–358 (1996). 8609628 10.1006/jmbi.1996.0167

[b18] Gonzalez-Guerrero M. & Arguello J. M. Mechanism of Cu^+^-transporting ATPases: Soluble Cu^+^ chaperones directly transfer Cu^+^ to transmembrane transport sites. P Natl Acad Sci USA 105, 5992–5997 (2008). 10.1073/pnas.0711446105 PMC2329688 18417453

[b19] Lubben M. *et al.* Structural model of the CopA copper ATPase of *Enterococcus hirae* based on chemical cross-linking. Biometals 22, 363–375 (2009). 18979168 10.1007/s10534-008-9173-4

[b20] Rensing C., Fan B., Sharma R., Mitra B. & Rosen B. P. CopA: An *Escherichia coli* Cu(I)-translocating P-type ATPase. P Natl Acad Sci USA 97, 652–656 (2000). 10.1073/pnas.97.2.652 PMC15385 10639134

[b21] Gourdon P. *et al.* Crystal structure of a copper-transporting PIB-type ATPase. Nature 475, 59–U74 (2011). 21716286 10.1038/nature10191

[b22] Wong D. W. S. in Metagenomics: Theory, Methods and Applications (ed. Marco D. ) 141–158 (Caister Academic Press, Norfolk UK, 2010).

[b23] Chevreux B. *et al.* Using the miraEST assembler for reliable and automated mRNA transcript assembly and SNP detection in sequenced ESTs. Genome Res 14, 1147–1159 (2004). 15140833 10.1101/gr.1917404 PMC419793

[b24] Monier J. M. *et al.* Metagenomic exploration of antibiotic resistance in soil. Curr Opin Microbiol 14, 229–235 (2011). 21601510 10.1016/j.mib.2011.04.010

[b25] McGinnis S. & Madden T. L. BLAST: at the core of a powerful and diverse set of sequence analysis tools. Nucleic Acids Res 32, W20–W25 (2004). 15215342 10.1093/nar/gkh435 PMC441573

[b26] Gupta S. K. *et al.* ARG-ANNOT, A new bioinformatic tool to discover antibiotic resistance genes in bacterial genomes. Antimicrob Agents Ch 58, 212–220 (2014). 10.1128/AAC.01310-13 PMC3910750 24145532

[b27] Tringe S. G. *et al.* Comparative metagenomics of microbial communities. Science 308, 554–557 (2005). 15845853 10.1126/science.1107851

[b28] Xie W. *et al.* Comparative metagenomics of microbial communities inhabiting deep-sea hydrothermal vent chimneys with contrasting chemistries. ISME J 5, 414–426 (2011). 20927138 10.1038/ismej.2010.144 PMC3105715

[b29] Fierer N. *et al.* Cross-biome metagenomic analyses of soil microbial communities and their functional attributes. P Natl Acad Sci USA 109, 21390–21395 (2012). 10.1073/pnas.1215210110 PMC3535587 23236140

[b30] Luo C. W. *et al.* Soil microbial community responses to a decade of warming as revealed by comparative metagenomics. Appl Environ Microb 80, 1777–1786 (2014). 10.1128/AEM.03712-13 PMC3957593 24375144

[b31] Mendes L. W., Kuramae E. E., Navarrete A. A., van Veen J. A. & Tsai S. M. Taxonomical and functional microbial community selection in soybean rhizosphere. ISME J 8, 1577–1587 (2014). 24553468 10.1038/ismej.2014.17 PMC4817605

[b32] Steven B., Gallegos-Graves L. V., Yeager C., Belnap J. & Kuske C. R. Common and distinguishing features of the bacterial and fungal communities in biological soil crusts and shrub root zone soils. Soil Biol Biochem 69, 302–312 (2014).

[b33] Chen L.-X. *et al.* Comparative metagenomic and metatranscriptomic analyses of microbial communities in acid mine drainage. ISME J 10.1038/ismej.2014.245 (2014). PMC4478699 25535937

[b34] Tyson G. W. *et al.* Community structure and metabolism through reconstruction of microbial genomes from the environment. Nature 428, 37–43 (2004). 14961025 10.1038/nature02340

[b35] Howe A. C. *et al.* Tackling soil diversity with the assembly of large, complex metagenomes. P Natl Acad Sci USA 111, 4904–4909 (2014). 10.1073/pnas.1402564111 PMC3977251 24632729

[b36] Li X., You F., Huang L., Strounina E. & Edraki M. Dynamics in leachate chemistry of Cu-Au tailings in response to biochar and woodchip amendments: a column leaching study. Environ Sci Eur 25, 32 (2013).

[b37] Sparks D. L. in Environmental Soil Chemistry (Second Edition) (ed. Sparks D. L. ) 43–73 (Academic Press, Burlington, 2003).

[b38] Li X. F., Huang L. B., Bond P. L., Lu Y. & Vink S. Bacterial diversity in response to direct revegetation in the Pb-Zn-Cu tailings under subtropical and semi-arid conditions. Ecol Eng 68, 233–240 (2014).

[b39] Li X., You F., Huang L. & Bond P. L. Establishing microbial diversity and functions in weathered and neutral Cu-Pb-Zn tailings with native soil addition. Geoderma 247-248, 108–116 (2015).

[b40] Suslov V. V., Afonnikov D. A., Podkolodny N. L. & Orlov Y. L. Genome features and GC content in prokaryotic genomes in connection with environmental evolution. Paleontol J+ 47, 1056–1060 (2013).

[b41] Ji X. L. *et al.* Antibiotic resistance gene abundances associated with antibiotics and heavy metals in animal manures and agricultural soils adjacent to feedlots in Shanghai; China. J Hazard Mater 235, 178–185 (2012). 22868748 10.1016/j.jhazmat.2012.07.040

[b42] Knapp C. W. *et al.* Antibiotic Resistance Gene Abundances Correlate with Metal and Geochemical Conditions in Archived Scottish Soils. Plos One 6, e27300 (2011). 22096547 10.1371/journal.pone.0027300 PMC3212566

[b43] Xie J. P. *et al.* GeoChip-based analysis of the functional gene diversity and metabolic potential of microbial communities in acid mine drainage. Appl Environ Microb 77, 991–999 (2011). 10.1128/AEM.01798-10 PMC3028740 21097602

[b44] Poirel J., Joulian C., Leyval C. & Billard P. Arsenite-induced changes in abundance and expression of arsenite transporter and arsenite oxidase genes of a soil microbial community. Res Microbiol 164, 457–465 (2013). 23396038 10.1016/j.resmic.2013.01.012

[b45] Giloteaux L. *et al.* Characterization and transcription of arsenic respiration and resistance genes during *in situ* uranium bioremediation. ISME J 7, 370–383 (2013). 23038171 10.1038/ismej.2012.109 PMC3554400

[b46] Besaury L. *et al.* Abundance and diversity of copper resistance genes *cusA* and *copA* in microbial communities in relation to the impact of copper on Chilean marine sediments. Mar Pollut Bull 67, 16–25 (2013). 23298430 10.1016/j.marpolbul.2012.12.007

[b47] Guigó R. *et al.* in Genomics and Proteomics (ed. Suhai S. ) 95–106 (Springer: US, , 2002).

[b48] Cha J. S. & Cooksey D. A. Copper resistance in *Pseudomonas syringae* mediated by periplasmic and outer-membrane proteins. P Natl Acad Sci USA 88, 8915–8919 (1991). 10.1073/pnas.88.20.8915 PMC52621 1924351

[b49] Hsiao Y. M. *et al.* Functional characterization of *copA* gene encoding multicopper oxidase in *Xanthomonas campestris* pv. campestris. J Agr Food Chem 59, 9290–9302 (2011). 21790191 10.1021/jf2024006

[b50] Agarwal S. *et al.* Structure and interactions of the C-terminal metal binding domain of *Archaeoglobus fulgidus* CopA. Proteins 78, 2450–2458 (2010). 20602459 10.1002/prot.22753 PMC2919055

[b51] Singh S. K. *et al.* Crystal structures of multicopper oxidase *cueO* bound to copper(I) and silver(I) functional role of a methionine-rich sequence. J Biol Chem 286, 37849–37857 (2011). 21903583 10.1074/jbc.M111.293589 PMC3199526

[b52] Brown N. L., Barrett S. R., Camakaris J., Lee B. T. O. & Rouch D. A. Molecular-genetics and transport analysis of the copper-resistance determinant (*pco*) from *Escherichia coli* plasmid prj1004. Mol Microbiol 17, 1153–1166 (1995). 8594334 10.1111/j.1365-2958.1995.mmi_17061153.x

[b53] Lee S. M. *et al.* The Pco proteins are involved in periplasmic copper handling in *Escherichia coli*. Biochem Bioph Res Co 295, 616–620 (2002). 10.1016/s0006-291x(02)00726-x 12099683

[b54] Biasini M. *et al.* SWISS-MODEL: modelling protein tertiary and quaternary structure using evolutionary information. Nucleic Acids Res 42, W252–W258 (2014). 24782522 10.1093/nar/gku340 PMC4086089

[b55] Arguello J. M., Mandal A. K. & Mana-Capelli S. Heavy metal transport CPx-ATPases from the thermophile *Archaeoglobus fulgidus*. Na,K-Atpase and Related Cation Pumps 986, 212–218 (2003). 10.1111/j.1749-6632.2003.tb07162.x 12763798

[b56] Coombs J. M. & Barkay T. New findings on evolution of metal homeostasis genes: Evidence from comparative genome analysis of bacteria and archaea. Appl Environ Microb 71, 7083–7091 (2005). 10.1128/AEM.71.11.7083-7091.2005 PMC1287752 16269744

[b57] Fan B., Grass G., Rensing C. & Rosen B. P. *Escherichia coli* CopA N-terminal Cys(X)(2)Cys motifs are not required for copper resistance or transport. Biochem Bioph Res Co 286, 414–418 (2001). 10.1006/bbrc.2001.5367 11500054

[b58] Fan B. & Rosen B. P. Biochemical characterization of CopA, the *Escherichia coli* Cu(I)-translocating P-type ATPase. J Biol Chem 277, 46987–46992 (2002). 12351646 10.1074/jbc.M208490200

[b59] Thompson J. D., Gibson T. J., Plewniak F., Jeanmougin F. & Higgins D. G. The CLUSTAL_X windows interface: flexible strategies for multiple sequence alignment aided by quality analysis tools. Nucleic Acids Res 25, 4876–4882 (1997). 9396791 10.1093/nar/25.24.4876 PMC147148

[b60] Li X. *et al.* Crystal structures of *E. coli* laccase CueO at different copper concentrations. Biochem Bioph Res Co 354, 21–26 (2007). 10.1016/j.bbrc.2006.12.116 17217912

[b61] Huang L., Baumgartl T. & Mulligan D. An examination of options and strategies for tailings revegetation at Mt Isa and Ernest Henry Mines (Phase 2) (Centre for Mined Land Rehabilitation, Sustainable Minerals Institute, University of Queensland, Brisbane, 2014).

[b62] Meyer F. *et al.* The metagenomics RAST server - a public resource for the automatic phylogenetic and functional analysis of metagenomes. BMC Bioinformatics 9, 386 (2008). 18803844 10.1186/1471-2105-9-386 PMC2563014

[b63] Jiang H. S., Lei R., Ding S. W. & Zhu S. F. Skewer: a fast and accurate adapter trimmer for next-generation sequencing paired-end reads. BMC Bioinformatics 15, 182 (2014). 24925680 10.1186/1471-2105-15-182 PMC4074385

[b64] Sentchilo V. *et al.* Community-wide plasmid gene mobilization and selection. ISME J 7, 1173–1186 (2013). 23407308 10.1038/ismej.2013.13 PMC3660673

[b65] Hall T. A. Bioedit: a user-friendly biological sequence alignment editor and analysis program for Windows 95/98/NT. Nucleic Acids Symposium Series 41, 95–98 (1999).

[b66] Delcher A. L., Harmon D., Kasif S., White O. & Salzberg S. L. Improved microbial gene identification with GLIMMER. Nucleic Acids Res 27, 4636–4641 (1999). 10556321 10.1093/nar/27.23.4636 PMC148753

[b67] Wilkins A., Erdin S., Lua R. & Lichtarge O. Evolutionary trace for prediction and redesign of protein functional sites. Methods Mol Biol 819, 29–42 (2012). 22183528 10.1007/978-1-61779-465-0_3 PMC4892863

[b68] Brown N. L., Rouch D. A. & Lee B. T. O. Copper resistance determinants in bacteria. Plasmid 27, 41–51 (1992). 1741459 10.1016/0147-619x(92)90005-u

[b69] UniProt C. UniProt: a hub for protein information. Nucleic Acids Res 43, D204–12 (2015). 25348405 10.1093/nar/gku989 PMC4384041

[b70] Edgar R. C. MUSCLE: multiple sequence alignment with high accuracy and high throughput. Nucleic Acids Res 32, 1792–1797 (2004). 15034147 10.1093/nar/gkh340 PMC390337

[b71] Tamura K., Stecher G., Peterson D., Filipski A. & Kumar S. MEGA6: Molecular Evolutionary Genetics Analysis Version 6.0. Mol Biol Evol 30, 2725–2729 (2013). 24132122 10.1093/molbev/mst197 PMC3840312

[b72] Uthandi S., Saad B., Humbard M. A. & Maupin-Furlow J. A. LccA, an Archaeal laccase secreted as a highly stable glycoprotein into the extracellular medium by *Haloferax volcanii*. Appl Environ Microb 76, 733–743 (2010). 10.1128/AEM.01757-09 PMC2812995 19966030

[b73] Roberts S. A. *et al.* Crystal structure and electron transfer kinetics of CueO, a multicopper oxidase required for copper homeostasis in *Escherichia coli*. P Natl Acad Sci USA 99, 2766–2771 (2002). 10.1073/pnas.052710499 PMC122422 11867755

[b74] Raimunda D., Long J. E., Sassetti C. M. & Arguello J. M. Role in metal homeostasis of CtpD, a Co^2+^ transporting P-1B4-ATPase of *Mycobacterium smegmatis*. Mol Microbiol 84, 1139–1149 (2012). 22591178 10.1111/j.1365-2958.2012.08082.x PMC3370075

[b75] Ogura M. ZnuABC and ZosA zinc transporters are differently involved in competence development in Bacillus subtilis. J Biochem 150, 615–625 (2011). 21813502 10.1093/jb/mvr098

[b76] Talavera G. & Castresana J. Improvement of phylogenies after removing divergent and ambiguously aligned blocks from protein sequence alignments. Systematic Biol 56, 564–577 (2007). 10.1080/10635150701472164 17654362

[b77] Andreazza R., Pieniz S., Okeke B. C. & Camargo F. A. O. Evaluation of copper resistant bacteria from vineyard soils and mining waste for copper biosorption. Braz J Microbiol 42, 66–74 (2011). 24031606 10.1590/S1517-83822011000100009 PMC3768903

[b78] Konstantinidis K. T. *et al.* Microbial diversity and resistance to copper in metal-contaminated lake sediment. Microbial Ecol 45, 191–202 (2003). 10.1007/s00248-002-1035-y 12545313

[b79] He L. Y. *et al.* Characterization of copper-resistant bacteria and assessment of bacterial communities in rhizosphere soils of copper-tolerant plants. Appl Soil Ecol 44, 49–55 (2010).

[b80] Singleton C. & Le Brun N. E. The N-terminal soluble domains of Bacillus subtilis CopA exhibit a high affinity and capacity for Cu(I) ions. Dalton T 4, 688–696 (2009). 10.1039/b810412c 19378562

[b81] Mann E. L., Ahlgren N., Moffett J. W. & Chisholm S. W. Copper toxicity and cyanobacteria ecology in the Sargasso Sea. Limnol Oceanogr 47, 976–988 (2002).

[b82] Rocap G. *et al.* Genome divergence in two *Prochlorococcus* ecotypes reflects oceanic niche differentiation. Nature 424, 1042–1047 (2003). 12917642 10.1038/nature01947

[b83] Huang Q. Y., Chen W. L. & Xu L. H. Adsorption of copper and cadmium by Cu- and Cd-resistant bacteria and their composites with soil colloids and kaolinite. Geomicrobiol J 22, 227–236 (2005).

[b84] Santo C. E., Taudte N., Nies D. H. & Grass G. Contribution of copper ion resistance to survival of *Escherichia coli* on metallic copper surfaces. Appl Environ Microb 74, 977–986 (2008). 10.1128/AEM.01938-07 PMC2258564 18156321

[b85] Tetaz T. J. & Luke R. K. J. Plasmid-controlled resistance to copper in Escherichia. coli. J Bacteriol 154, 1263–1268 (1983). 6343346 10.1128/jb.154.3.1263-1268.1983 PMC217599

[b86] Kunito T. *et al.* Characterization of Cu-resistant bacterial communities in Cu-contaminated soils. Soil Sci Plant Nutr 43, 709–717 (1997).

[b87] Yang C. H., Menge J. A. & Cooksey D. A. Role of Copper Resistance in Competitive Survival of *Pseudomonas. fluorescens* in Soil. Appl Environ Microb 59, 580–584 (1993). 10.1128/aem.59.2.580-584.1993 PMC202147 8434924

[b88] Saxena D., Joshi N. & Srivastava S. Mechanism of copper resistance in a copper mine isolate Pseudomonas putida strain s4. Curr Microbiol 45, 410–414 (2002). 12402081 10.1007/s00284-002-3787-5

[b89] Cazorla F. M. *et al.* Copper resistance in *Pseudomonas syringae* strains isolated from mango is encoded mainly by plasmids. Phytopathology 92, 909–916 (2002). 18942971 10.1094/PHYTO.2002.92.8.909

[b90] Bender C. L. & Cooksey D. A. Indigenous plasmids in *Pseudomonas. syringae* pv tomato - conjugative transfer and role in copper resistance. J Bacteriol 165, 534–541 (1986). 3003029 10.1128/jb.165.2.534-541.1986 PMC214452

[b91] Galai S., Lucas-Elio P., Marzouki M. N. & Sanchez-Amat A. Molecular cloning of a copper-dependent laccase from the dye-decolorizing strain *Stenotrophomonas maltophilia* AAP56. J Appl Microbiol 111, 1394–1405 (2011). 21973274 10.1111/j.1365-2672.2011.05164.x

[b92] Stuart R. K., Dupont C. L., Johnson D. A., Paulsen I. T. & Palenik B. Coastal strains of marine synechococcus species exhibit increased tolerance to copper shock and a distinctive transcriptional response relative to those of open-ocean strains. Appl Environ Microb 75, 5047–5057 (2009). 10.1128/AEM.00271-09 PMC2725496 19502430

[b93] Lugo A. J., Elibox W., Jones J. B. & Ramsubhag A. Copper resistance in *Xanthomonas campestris* pv. campestris affecting crucifers in Trinidad. Eur J Plant Pathol 136, 61–70 (2013).

