# Supplementary material for: Assessing the genetic diversity of Cu resistance in mine tailings through high-throughput recovery of full-length copA genes
Source: Sci Rep. 2015 Aug 19;5:13258. doi: 10.1038/srep13258 (PMC4541151; doi:10.1038/srep13258)
Supplement: Supplementary Information [file srep13258-s1.pdf]

# Assessing the genetic diversity of Cu resistance in mine tailings through high-throughput recovery of full length *copA* genes

Xiaofang Li[1], Yong-Guan Zhu[2], Babak Shaban[3], Timothy J. C. Bruxner[4], Philip L. Bond[5], Longbin Huang[1]*

[1]Centre for Mined Land Rehabilitation, Sustainable Minerals Institute, The University of Queensland, QLD 4072, Australia

[2]Institute of Urban Environment, Chinese Academy of Sciences, Xiamen, 10081, China

[3]Australian Genomics Research Facility, Parkville, Melbourne 3000, Australia

[4]Institute for Molecular Bioscience, The University of Queensland, QLD 4072, Australia

[5]Advanced Water Management Centre, The University of Queensland, QLD 4072, Australia

***Corresponding authors**

Longbin Huang: l.huang@uq.edu.au; also to Yong-Guan Zhu: ygzhu@iue.ac.cn

**Running title: Metagenomics for *copA* diversity in mine tailings**

| 16 | 84 | 455 |
| --- | --- | --- |
| GMTCASCSAR | ----------- | VLVVACPCA- |
| GMTCASCSSR | GMTCASCVGRV | VLVVACPCA- |
| GMTCASCSSR | GMTCASCVGRV | VLVVACPCA- |
| GMSCASCVGR | GMTCASCVGRV | VLIIACPCA- |
| GMGCAACAGR | ----------- | VLIIACPCA- |
| GMTCAMCVKS | ----------- | VLVVACPCA- |
| GMTCASCAGR | ----------- | VLVIACPCS- |
| GMTCANCSAR | ----------- | VLVIACPCA- |
| GMTCAACSNR | GMTCAACSNRI | VLVIACPCS- |
| GMTCAACANR | GMTCAACANRV | VLVIACPCA- |
| GMTCAACAAR | GMTCAACANRI | VLVIACPCA- |
| G--HACHHEH | ----------- | VLIIACPCA- |
| NMNCAGCVAK | ----------- | VLIIACPCA- |
| GLSCGHCVKR | GMSCASCVSRV | VLIIACPCA- |
| GLSCGHCVKR | GMSCASCVTRV | VLIIACPCA- |
| GLSCGHCVAS | GMSCASCVSKV | VLIIACPCA- |
| GLSCGHCVAS | GMSCASCVSKV | VLIIACPCA- |
| GMKCAGCVAA | ----------- | VLVVACPCA- |
| HARHQEHGMG | ----------- | VMVIACPHA- |
| ---HQEHGMG | ----------- | VMVIACPHA- |
| ---HSGHQKQ | ----------- | VMVIACPHA- |
| ---CYHCGLP | GVSCAACGWLI | LLVATCPCA- |
| ---CYHCGLP | GISCAACGWLI | MLVATCPCA- |
| -------VNR | ----------- | LLVIGCPGA- |
| WVRCDSRRAV | ----------- | MLLIACPCA- |
| GMACAFCAST | ----------- | VSTIAYPCA- |
| ---------- | ----------- | FMVVASPCA- |

| 100 | 142 | 570 |
| --- | --- | --- |
| LHWHGL | PHQH | AHCHL |
| IHWHGY | YHSH | FHCHD |
| LHWHGL | MHSH | NHCHN |
| IHWHGL | YHAH | FHCHH |
| IHWHGL | YHSH | FHCHQ |
| IHWHGI | YHSH | FHCHL |
| VHWHGL | YHSH | YHCHL |
| LHWHGI | YHSH | YHCHL |
| IHWHGI | YHSH | YHCHL |
| IHWHGI | YHSH | YHCHL |
| IHWHGI | YHSH | YHCHL |
| IHWHGI | YHSH | YHCHL |
| IHWHGI | YHSH | YHCHM |
| IHWHGI | YHSH | YHCHM |
