# Supplementary material for: Assessing the genetic diversity of Cu resistance in mine tailings through high-throughput recovery of full-length copA genes
Source: Sci Rep. 2015 Aug 19;5:13258. doi: 10.1038/srep13258 (PMC4541151; doi:10.1038/srep13258)
Supplement: Supplementary Information [file srep13258-s2.pdf]

Assessing the genetic diversity of Cu resistance in mine tailings through high-throughput recovery of full length *copA* genes

Xiaofang Li[1], Yong-Guan Zhu[2], Babak Shaban[3], Timothy J. C. Bruxner[4], Philip L. Bond[5], Longbin Huang[1]*

[1]Centre for Mined Land Rehabilitation, Sustainable Minerals Institute, The University of Queensland, QLD 4072, Australia

[2]Institute of Urban Environment, Chinese Academy of Sciences, Xiamen, 10081, China

[3]Australian Genomics Research Facility, Parkville, Melbourne 3000, Australia

[4]Institute for Molecular Bioscience, The University of Queensland, QLD 4072, Australia

[5]Advanced Water Management Centre, The University of Queensland, QLD 4072, Australia

***Corresponding authors**

Longbin Huang: l.huang@uq.edu.au; also to Yong-Guan Zhu: ygzhu@iue.ac.cn

Running title: Metagenomics for *copA* diversity in mine tailings

Notes:
1-12 Alpha Proteobacteria
13-22 Deta Proteobacteria
23-47 Gamma Proteobacteria
48-54 Delta Proteobateria
55-59 Epsilonproteobacteria
60-74 Firmcutes
75-91 Actinobacteria
92-97 Cyanobacteria
98-110 CFB group bacteria
111-113 Mycoplasmas
114-116 Chlamydias
117-119 Acidobacteriaceae
120-122 GNS bacteria

>gi_294009986_Sphingobium japonicum UT26S chromosome_1

ATGACCCTTTCCGATGTCGATCGCCGCAGCCTTTTCCGCAGCCTTTGCGGGTGGGGCGGCGCGCTGGCCC

TCAGCCGGATTTTTCCCGCCTGGGCGCAGAGCGGAACGGCGGGCCTGTCCCTGGCTCCCGGAGCGCTGAG

CGGCGAGACCATCGCGCTGACCGTGGCGGAGGGGCATTTTTCGACCGGCGGGCGTTCGGGCCACGCGGTG

ATGATCAACGGCACGCTGCCTGCGCCGCTCATCCGCTTGAGGGAAGGGCAGAATGTCCGGCTGTCCGTCA

CCAATCGCCTGCGGGAGGACACGTCGATCCACTGGCACGGCCTGATCGTGCCGTTCCAGATGGACGGGGT

GCCGGGGATCAGCTTTCCCGGCATCCGCCCGGGCGAGACCTTCACCTATGAATTTCCGGTCCGCCAGTCG

GGCACCTACTGGTATCACAGCCATTCTGGAATGCAGGAGGCGATGGGCCATTATGGACCGATCGTGATCG

ACCCCGCCGGGCCGGACCCCGTGCAGGCGGAGCGCGAGCATGTCGTCATTCTGTCGGACTGGAGTCCGGT

TCACCCGCATGTCCTGCTGCGGCGGCTGAAGCAGATGGGCGGCTATTTCAACATGCAGAAGCAGACCTTG

TCGGGCCTGCTTGCGGGCAAGGACCAGAACGCGAAGGAGCGCCTTCAATGGGGCGCCATGCGCATGGACC

CGACCGACATATCCGACGTCACCGGCTCGACCTACAGCTTCCTGGCGAACGGCCATGGCACGGCGGAGAA

CTGGACAGGACTCTTCACGCCTGGCGAGCGGGTGCGGCTGCGCATCGTCAATGCGGCCGCCATGACCAAT

TTCAACCTGCGCCTGCCGGGACTGCCGATGACGGTGGTGCAGTGCGACGGCCAGCATGTCCAGCCGGTGG

AGACCGACGAGATCCAGATCGGCATCGCGGAGACCTATGATGTGGTGGTGCGGCCCACCGAAGCGAAGCC

CTATGCCCTGATCGCGGAGGCGATAGACCGCTCCGGACTGGTGCGGGCGACCCTGGCGCCCCGGATCGGC

ATGGCGGCGGACGTGCCCGCGCTGCGCGAAAGGCCGCTGCTGGGCATGAAGGACATGGGCATGGACATGT

CCGGCATGGGGGATATGCCCGGACATTCCATGAAGATGCGCGACCCGTCCGTCGCGCCGCAGGTGAAGAT

GGGACCGGGCGTCGCCACCCTCTCCCCCATGCCGGTGGACCGGACCGCCGACCGGCCCACGGGGCTGGAG

AGCGTCGAGCATCGGGTGCTGACCTATGCCGACCTGAAATCGCTTGAGCCGAACAGCGACAGGCGCGCGC

CGACCCGCACGCTGGACATCCACCTGACCGCGAACATGGAGCGCTACATGTGGTCCTTCGACGGGGTGAA

GCTGTCCGACGGGGCCGAACCCATCGCCTTCCGCCATATGGAGCGGGTGCGGGTGAACCTGATCAACGAC

ACGATGATGCCGCACCCGATCCATCTGCACGGCCATTTCTTCGAGCTTGTGACCGGCGCCGGCGACCATA

ATCCGCTCAAGCATACGGTGAACGTGCTGCCCGGAGGGAAAGTGAGCTTCGACCTTACGGCAGACGCGCT

GGGCGATTGGGCCTTTCACTGCCATATGCTGATGCACATGCATGCAGGGATGATGCGCGTCGTGACCGTT

CGGCATGCGGCGGACGCGGCATGA

>gi_258541105_Acetobacter pasteurianus IFO 3283-01_2

GTGCAGCATGATGCAGAATGGACAATACCCATGCATATTCCCCCTGTTTTGAAAGGGAGAAATGTGTCTC

GTCGGCAGTTTATAACTGGCGGCAGCGCATTGGCGGCCCTGTATGCCCTTAAGCCACCCAAACAGGTTTT

TGCTTCTGCCCACACATCCGGAATTGTGCCCCAAAACGCCAGCAACACCTTTGATCTGACCATCGGCTGG

GCGAATGTAAATATTCTGGACAAACCACAGCGCGTGCCCACCATGAATGGCGGTGTACCCGGCCCCATCC

TGCGGTGGCGTGAAGGCGATAATTTACACCTGAACATCCATAATACATTGGACGAAACAACCTCCATCCA

CTGGCATGGCATCCGCTGCCCGGCACATATGGATGGCGTGCCGGGGCTAAGCTTTGCCGGAATTGAACCC

GGCGCACGCTTTACCTATCGCTTTCCTGTAAAACAGAGCGGCACATACTGGTATCACAGCCACTCCAACA

TGCAGGAAGCTATGGGGCTGTATGGGGCCATTGTGATTGACCCCAAGGAGCCAGACCCCAATAGTTATGA

CCGGGATTACGTCATCCTGCTTTCAGATTGGTCTGATGTGGCACCGCACACCATCATCAACAAACTGAAG

TTTCAGGATGACTACTATAATTTCCGGCAACGCACGGCTGTTTCGTTCTTTCATGATGCCAAGAAATCCG

GCGTGTGGCCGACACTAAAAGACCGCATGCAGTGGGCTGGCATGAACATGTCCGCCACGGATATTTCAGA

CGTAAGCGGCATTATCTACACATATCTGATGAACGGCTGCGCCCCGGCTGCGAACTGGACCGGTATTTTT

AAACCCGGTGAACGTATCAGGCTGCGGTTTATCAACGCTTCCGCCATGACCTTTTACGATATCCGCATTC

CCGGCCTCCAGATGGATATCATTCAGGCGGATGGAAATAATGTTGAACCCGTGCGGGTAGATGAATTCCG

CATTGGTGTGGCTGAAACGTATGATTGCATTGTGCAGCCGCAAAACGATCAGGCCTACACCATTTTTGCC

CAAAGTGAGGACAGAACCGGCTACGCCCGCGGCACGCTCGCGCCTCATTCCGGCATGAGCGCACCTGTTC

CCCCCATGGATGAACGGCCCGTACGCACCATGGTGGATATGGGCATGGCCATGCACGAAGGGCACGATAT

GGGTGGCATGGATATGCACGACATGCCCATGCACCAAGCCACAAAAACGCATGCGCCGCCTTTGAATGTA

GAAAACCAGAACCGTGCCGCCATGCCGATCAATCGTCTGGCTGACCCCGGTGACGGCTTACAAAACAACG

GCCGACGGGTTCTGACATATGCAGACCTGCGCGCCACTCACCCCGCCACAGATCAACGCCCACCATCGCG

CGAGATCACGTTGCACCTTACCGGCAACATGGAACGTTATATCTGGGGGTTTGATGGCAAAAAGTTCTCG

GAAGCCGAGCCTATCCCTCTGCGTTTGGGTGAACGGGTGCGCTTTACCCTGATAAATGACACCATGATGG

AACACCCCATCCATCTGCATGGTTTGTGGAGCGAACTGGAAAACGGGAACGGCCTGTACAACCCGATAAA

ACACACCATTATTGTGCAACCGGGCGGAAAGCTGAGCTATCTGGTTTCTGCCGATACACCGGGTTTATGG

GCTTTTCACTGCCACCTGCTTTACCACATGGATTTAGGCATGTTCCGCACGGTGGTGGTGTCATGA

>gi_347756788_Micavibrio aeruginosavorus ARL-13 chromosome_3

ATGAAATTTTACGCCTATACTCTATTTTTGTGCCTGTTCTTTAGCTTTGCCAGCCCGGCCAAGGCGGAGG

AATACAATCTTGTCATTGCCCATGAACCCGTGAATATCACCGGTAAGACCGTTGAAAAAATCACCATTAA

CGGCACCATTCCCGGCCCGACCTTGCGGTTTACCGAAGGCGAGGATGCCATCATTCATGTCACAAACAAG

ATGGATGAGGATACGTCCGTCCATTGGCACGGCCTGATTTTGCCCGGTGAAATGGACGGTGTTCCCGGCT

TCAATGGTTTTCCGGGTATCAAGCCGGGCGAAACCTTCACCTATCGCTTCCCTGTTAATCAAACCGGCAC

CTATTGGTATCACGCGCATAGTTCCGAACAGGAACAGGACGGACATTACGGCTCTCTCGTGCTTGTCCCG

AAAGGCAAAGACCCGATCCGCGCCGACCGCGATTATGTTGTCCTTTTATCAGATTTCCATGATGAAGACG

GAAAAGATATTTTCAGCAATTTGAAAATGTCGTCTGAGTATTACCAATATGCTCGCCGCACGATTGGAGA

TTTCTTTGCGGACGCGGAAGAAAAAGGATTTGGCAAAGCATGGGCCAACGCCAAAATGTGGGGCGAAATG

CGGATGCTGCCGACCGACCTTTCTGATGTTTCCGGCTATACATTTCTCGTGAACGGCAGAACGCCTGAAC

AAAACTGGACGGCCCTGTTCAAACCCGGCGAACGGGTGCGCCTGCGTTTCATCAACGCTTCGGCCATGAG

CATTTATGATGTGCGAATTCCCGGTCTGAAAATGAGTGTGGTTGAGGCGGACGGCCAAGGCGTAGAGCCG

GTTACGGTTGATGAATTCCGCTTTGGCGTTGCGGAAACCTATGACGTTATTGTGACGCCCAAGGAAGACA

AAGCCTTCACGATTGCAGCGGAATCTATCGACCGCACTGGCTTTGCACTTGGCACCCTTGCACCGCGCGA

AGGCATGAAAGGCCCGGCACCGCAGACGCGCCCGCGTGCTTTGCTGACTATGGCGGATATGGGTATGGGC

CACGATATGGCGGGCATGGAAGGCATGGACCATTCCAAAATGAACCATGATATGCCGATGGGCGATATGG

CAGGGATGGATCATGGTAACATGGGACATGACATGGTCGGAATGTCTATGGAGGATATGCAAAGCGGCTG

GGCGCAAGCGGGAACCCCGCCGGGCGACAAGGCTTTAAGCTATGCCGATCTTCGCTATGTCGGCATTCAG

GCAGACACGCGCCAGCCGGAACGTGAAATTCTTGTCCGTCTTGGCGGCAACATGGAGCGTTTCATATGGA

CAATTAATGACAAGAAATTTGAAGATTCCGGGCCGATCAATCTGAAATACGGCGAGCGCGTTCGCCTGAA

ATTCGTCAATGATACGATGATGGCGCACCCTATGCACTTGCACGGAATGTTTGTTCAACTTGAAAACGGC

CAGCCCGCCGAAAAATTACCGAACAAGCACACGGTCATTGTCGCACCGGGGCAAACCTATTCGGTATTGC

TGACCGCCGATCAACCCGGCGAATGGGCGTTCCATTGCCATCTCCTGTTTCATATGTCCGCAGGTATGAT

GAACAAGGTGGTTGTAGCAAAGCTTGACCCTAATGCCGTGCCGCCGCCGAAAGCATCCACCACGAAGGCT

GCGCCGCAAAAAGAAAAGCCGGAACAGGCTGATCCGCATGAAGGGCATTCCATGCCGATGAACCATCAAG

GCATGGACATGAAAGGCATGGACGACATGCCGAGTATGGATCATTCCACGCCTCAAAAAAATGATACTTC

GTCAAATCAAAATTCTTCTCACGGAGGCCACGATGCACACTAA

>gi_162145846_Gluconacetobacter diazotrophicus PAl 5 chromosome_4

ATGATTTTTCCATCCTCGACGGGGCGGGGCGCGATGACGCGCCGCCGCTTCGTGGTCGGGGCCGGCGTGT

GGGGTGCGGCGGCCGCGACCGGCGCCGGTGCGCGGGCGTTCGCGCCGCCTTCCGGCATCCCCGCCCCCCG

GCCCGGCACCCGTTTCGACCTTGCGATCGGTCACGTCCCGGTCAACGTCACGGGCGCGCGCGTCCGCGCG

GTCGGCGTCAACGGATCGACCCCGGCGCCGATCCTGCGCTGGCGACAGGGCGATACGGTCGAACTGACCG

TCACCAACCGGCTGCACGAACCCAGTTCGATCCACTGGCACGGTATTCGGCTGCCGGCCGGCATGGACGG

TGTCCCGGGCCTCAGCTTCGGTGGCATCGCGCCGGGCGAAAGCTTCACCTACCGTTTCCCCGTACGGCAG

AGCGGCACCTACTGGTACCACAGCCATTCCGGCTTCCAGGAGCAGACCGGCCTGTACGGCGCCATCGTCA

TCGACCCGGGCAGCGGCGACACCCGGCGGTGCGACCGCGATTATGTGCTGGTGCTGTCGGACTGGACCGA

CGTGGACCCGGCGGACATCGTCTCCAACCTGAAATTCCAGAGCGACTATTACAATTTCCGCCAGCGCACG

GTCGGCACCTTCTTCCGCGACGCGCACCGCCAGGGGCTGGGCGCCGCGGTGCGGGACCGCCTGGCCTGGG

GGGGCATGAACATGGCGCCGACCGACATCCTGGACGTGTCGGGCATCGTCTACACCTACCTGGTCAACGG

GCAGTCCCCCCGCGCCAACTGGACCGGCCTGTTCCGCCCCGGCGAACGCATCCGTCTGCGCATCATCAAT

GCCGCGTCGATGACCCTGTTCGACGTGCGGATTCCCGGACTGGCGATGACCGTCGTGCAGGCCGACGGGA

ACGACGTGGAACCGGTGACGGTGGACGAATTCCGCATCGGGCCCGCCGAAACCTACGACGTGATCGTGAC

CCCCGCCGCCGCCGGCCCCTATACGATCTTCGCGCAGGCCGAGGATCGCACGGGCTACGCGCGCGGCACG

CTGGCCACCGCCCCGGGCCTCGCCGGCCCCGTGCCGCCCATGGACCCCCGCCCGCTGCGCACCATGGCGG

ACATGGGCATGGGCGGCATGGCCCCGCCCGCGAAAACCGGCAACGCGCCCGCCATGGACGGTATGGACAT

GTCCGGAATGTCCGGAATGGACATGGACCCCAAGGAGATGGAACCCCCGGCCGCGCCACATCAGGCGCCG

CCCGCGCCGAAGCTGAAGCCGGGGGTCGAGGTGCAGAATGTCGCCGCCATGCCCATGGACCGGCTGGCGG

AACCGGGGGACGGGCTGGACCATAACGGCCGCCGCGTCCTGACCTATGCCGATCTGCGGGCGACCGTCCC

CGGCATCGATCCCCGCCCGCCGTCGCGCGAGATCACCCTGCACCTGACCGGCAACATGGAACGCTTCATC

TGGGGCTTCGACGGCAGGAAGTTCTCGGAAGCCGCGCCGATCCGGCTCGCGCGGGGGGAGCGGGTCCGCT

TCGTCCTGATCAACGACACGATGATGGAACACCCGATCCACCTGCACGGGTTGTGGAGCGAGCTGGAAAA

CGGGCAGGGGGACCGCCGCCCCTACAAGCACACGATTACCGTCAAGCCGGGCGAGCGGCTGAGCTATCTG

GTCACGGCCGACGAACCCGGATTATGGGCCTATCACTGCCATCTGCTCTACCATATGGCAGTCGGCATGT

TCCGCACGGTGGTGGTGTCGTGA

>gi_56708791_Ruegeria pomeroyi DSS-3 megaplasmid_5

ATGCCTCACATCTCAGCTTGGCGCGCGGCCCTTGCCGGCGCCACCCTCTTGCTGTCCGCGGCGACGGCGG

CACAAGCGGGAACCTATGACATAACAGTGGACAAGATCCGAATCGACGCAGGAAACTTCCGCAAGAACGG

GATCGGCTATAACAACAGCCAGACCCCGACGATCCTGCGCTTCAAGGAGGGTGAAAAGGTCACGCTGAAC

GTGACCAACAACCTGCGCGAGGATACCTCGATCCACTGGCACGGGCTGATCCTGCCCTTTGACCAGGACG

GGGTGCCAAAGATCAGCTTTGACGGCATCAAGCCGGGCGAGACCTTTACCTATAACTTTCCGATCGTCCA

GGCCGGAACCTATTGGTTTCACAGCCATTCGGGTTTCCAGGAACCGGACGGGGCCTATGGCGCCATCGTG

ATCGAGCCCAAGGGCGGAGAGCGCATCCGCGCCAACCGCGATTATGTGGTGCAACTGACCGACACCCACC

CGCAGCGCGGCAAGCGGATCATGCGCAACCTGAAAATGTCGGCGGATTACTACAACCGCTCGCAGCGTAC

ATTGCAGGACCTGATCAAGGATTCGGGCGATATCGGCCTCAAGGCGGCGCTGGAAGACCGCAAGATGTGG

GGCCGGATGCGGATGATGCCCACGGATGTCGAGGACGTCCAGGGATTCACCCCCATGATCAACGGAGAAA

GCACCGAGCAGAACTGGACCGGCCTGTTCAAGCCGGGCGAAAAGGTGCGGCTGCGGCTGATCAACTCCTC

GGCAATGGCCTATTTCGACGTGCGGATCCCGGGCCTCAAGATGACCGTAGTTCAGGCCGACGGCAATGAT

GTCAAGCCGGTCACGGTGGACGAACTGCGGATCTCGGTGGCCGAAACCTATGACGTGATCGTGCAGCCCA

AGGAAAACCGCGTCTACAGCATCGTGGCTGAATCGATGGGCCGCACGGCCCTGGTGCGCGGCACCCTGGC

CCCGCGCGAAGGCATGGCCGGACCGGTGCCGCCGCTGCGGGAAAAACCGCGCCTGACCATGGCCGACATG

AGCGGCATGATGGGCAGCATGGGCATGGAAGGCCATGTCATGCAAAGCGCCGCCAAACCTCTCGACGACA

TGAGCGGCATGGATCACAGCCAGATGAACATGCAGCCCAAACCGCAACCGATGGAGGGCATGGACCACAG

CCAGATGGGCCACGCCGCACCGGCCCCCAAGGCCGAGGCCGCGCCGATGGATCACTCTGCCATGACCGGC

ACGGACCAACCGATGGCCGGTATGGATCACAGCCAGATGGGTCATGCCGCGCCGGCGCCTCAGGCCAAGG

CCGCGCCGATGGACCACTCCACCATGACCGGCGCGGACAAGCCGATGGCCGGTATGGATCACTCCAGGAT

GACCGGGTCCGGCAACTCGATGGCGGGCATGGATCATTCCGCGATGGCCAATGCCAAGGGCCAGGGCGAC

CCGTTCTACGCCCCCGGCAGCGGGCTGACCCCGAAGGCGGCCAATGGCGGCAAGTTCCTCAGCTATGACG

ACCTGACGGCGCGCAAACCGCTCTACAAGGATCGCCCCGCCACCCGCGAGATCGAGCTGCGCCTGACCGG

CAACATGGAACGCTACATCTGGTCGATCAACGGCAAGAAACTGTCCGAGGCAGAGCCGATCGTGCTGAAG

TACGGCGAGCGGGTGCGGTTCAAATTCGTCAATGAAACCATGATGTCCCACCCGATGCACCTGCATGGCA

TGTGGTCGATCCTGGATACCGGCAAGGGCCAGTACGACCCGATCAAACACACGGTCAGCGTCGCGCCGGG

CACCACCGTCTATACTGAGACCGAGGTCGATGTGACCGGCCAATGGGCCTTTCACTGCCACCTGTCCTAT

CACGCCGACGCGGGCATGTTCCGCAAGGTCGTGATCCAAGGCGGCCCCGCCTGA

>gi_240136783_Methylobacterium extorquens AM1_6

ATGACGAACTTATTGTCCCGCCTTTCCGCCTGCCTTCTCGGGGCCGCGCTCGCGGTCATGGTTCTTGTGC

CGGTTTCCGCCCGTGCCGGCACCTACGACCTCGTGCTCGAGCGCCGCACGGTGAACGTCACGGGACGCGA

GCGCGTCGGCATGCTCATCAATGGCCAGCTTCCCGGGCCTACGCTGCGGTTCAAGGAAGGCGAGGACGTG

GTGATCAACGTCACCAACCGCCTCGAAGAGAATGCCTCGATCCACTGGCACGGCCTGATCGTGCCGCCCG

AGATGGACGGCGTTCCCGGCATCTCGCCGGGATTCGGCAACGGCATCAAGCCGGGCGAGACGTTCACCTA

CCGTTATAAAATCCGGCAGGCGGGAACGTATTGGTACCATAGCCACTCCTCCGGCGAGCAGGAGCAGCTC

GGGATTTACGGGCCCCTGATCATCGAGCCCCGCGAGCGCGAGCCGTTCAAGCATGACCGCGATTACGTGA

TCATGCTCTCGGACTGGACCGACGAGGACCCGAAGCGGATCATCAAGAACCTGAAAAGCGAAAGCGGCTG

GTACAACCGCAACCAGCGCACCCTCGTGAGCCTGATCCAGGAGCTCGGCAAGGCTCCGAACGCCGAGGCA

CGGGAGGCCGTCATCAGGAGCCACGTCGCCTGGAGGGTGATGCGCATGGACCCGACCGACATCGCGGATG

TCGGGGGTCTCCACTACCTGGTGAACGGCCTTACGCCGGAGCGCAACTTCACCGCCCTCTTCAAGCCCGG

CGAGCGGGTCCGGCTGCGCTTCATCAACGCTTCCGCGATGACCTATTTCGACGTGCGCATCCCGGGGCTA

AAGATGACGGTCGTTCAGGCGGACGGCAACAACGTCCAGCCGGTGCCGATCGACGAGTTCCGTTTCGGGA

ACGCCGAGACCTACGACGTGATCGTGCAGCCGACCGAGGACCGCGCCTACACCATCGTGGCCGAGCCCCT

GGACCGCACCGGGTTCGCCCGCGCGACCTTGGCGACACGACCTGGGATGGTGGGCGAACTGCCGCCCCAT

CGCGTCCGCCCGCTCACGCAGATGTCCGAAATGCTGATGAACCCCGGCCCCAAAGGCGGCGACCGGGGCA

CGCCCCAGCCCGAGCAGGATATGCCCGGCCATGTGCAGCCCGCGAGCCCCCTCGCGATGGCGATTGGCGG

GATGGGCGACATGAACATGGGCGGCATGGGCGGTATGGGCGGCCAGCAAGGCGGGCACGGCGGTATGGCC

GGCATGCAGGGCGGATCAATGCCTCAGGGCGGCATGTCCGGCATGGGCCAGGGCCGTGCGGGGCAGGGCG

GCGGCAGGAGCGCCACGGCGAGGAAGCCGGCCGGTTCTGCTGCGATGCCGGGAATGGACCACGGCGCCAT

GCCGGGGATGAGCCAGGGGCGCCAGGCCCGAACGACGGCCGGGGCGCAGATGCAGGGCATGGATCACGGC

GGCATGCCGGGGATGAGCCAGGGGCGCCAGGCCCGAACGACGGCCGGGGCGCAGATGCAGGGCATGGATC

ACGGCGGCATGCCGGGCATGGGCTCGAGCGCCTCGCCCGGCGGCCAGAACCAAGGGATGTTCGCGGCCCT

GAACGGGGGACTGCAGCAAGGCCCCCAGGCCAAGGTGCTCCACGGAGGCGAGGACATGCCGAACGAGGGT

CCGACGCTTGAGAACGCCATGCCCGATCCCCTCGTGAACCGGACGGGCGCGCCGCCCGGCACCAAGGTGC

TGTCGTACCGGGATCTCAAGGCGCTGAAGCCTTATCCGTACAAGCGCTACGACCGCATCATCGAGATGCG

TCTGACCGGCAACATGCAGCGCTACATCTGGTCGATCAACGGCCGCAAGTTCAGCGAGGCGCAGCCGATC

GTGCTGCGCTACGGCGAGCGAGCCCGCTTCCGGTTCATCAACGAGACCATGATGCCCCATCCGATGCACA

TCCACGGCACCTGGTTCCTGCCGGAGGTGGGCAACGGCGCGCGCAACCCGCTCAAGCACACCGTCAACAT

CAACCCCGGCGCGACGCTCGACGTCGATGTGCCGGCGGATGCGGAAGGACCGTGGGCGTTCCACTGCCAC

CAACTTTACCACATGGAGACCGGGATGATGCGCAAGATCATCGTGGTCCGAGAGACCGCCCGGCGGTGA

>gi_27375111_Bradyrhizobium japonicum USDA 110 chromosome_7

ATGTATTCCCGCCGAGGATTCTTGGGCACTGCCGCGCTTGCCAGCGCGTCGGCCATCAGCGGCCGCGTCC

AGGCTGCGTCGATTCCGGAAGCGCCCACTATGGACAAGGTGGTGATGCAGCCGCCGCTGCATCCGATCAG

CGGCCCGGACTATCGCCCCGTGGTCACGCTGAACGGCTGGTCGCTGCCGTTCCGCATGAACGGTGACTGG

AAGGAATTCCACCTCGTCGCCGAACCCGTGGTGCGCGAGTTCGCCGAAGGCATGAAGGTGAACCTGTGGG

GCTATAACGGCCAGTCGCCGGGCCCGACCATCGAGGCCGTCGAGGGCGACAAGGTCCGCATCTTCGTCAC

CAACAAATTGCCCGAATACACCACCGTTCACTGGCACGGCATGATCGTGCCGAGCGGCATGGACGGCGTC

GGCGGGCTGAATCAGCCGCACATCGATCCGGGCAAGACCTTCGTCTACGAGTTCGAGATGAAGAAGAGCG

GGACCTTCATGTACCACCCGCATTCCGACGAGATGGTGCAGATGGCTATGGGCATGATGGGCATGGTCGT

CGTGCATCCGCGCGACCCGAATTTCCGGCCCGTCGACCGCGACTATGTCTTCGTGATGAGCACCTATCGC

GTCGATCCCGGCAGCTACCTGCCGCGGGTCAACGAGATGACCGACTTCAACATGTGGACCTGGAATTCGC

GGGTGTTTCCGGGGATCGATCCGCTGCCGGTGCGGCTCGGCGACAAGGTGCGCGTGCGCATCGGCAATCT

CAGCATGACCAACCATCCGATCCATCTGCACGGCCACAGCTTCGCGGTGACCTGCACCGACGGCGGCTGG

ATTCCGGAGAGCGCGCAGATTCCCGAGACGACCACCGACGTCCCCGTCGGCGCTGTGCGGGTGTTCGACG

TGCTCGCCGACAATCCCGGCGACTGGGCGTTCCACTGCCACAAGTCGCATCACACCATGAACGCAATGGG

ACACGAGGTGCGCACCCTCATTGGCGTGTCGCGCAAGGATCTTGCCAAGGCCGTCGGCAAGCTCGCACCA

GACAGCATGGCGATGGGCTCGACCGGCATGGCGATGGGCAACATGGAGATGCCCGCGCCCGACAACACGC

TGCCGATGATGACCGGCACCGGCCAGTTCGGCCCGATCGAGATGGGCGGCATGTTCACGGTGATGAAGAT

CCGCGAGGACCTCGCGCGCGACGATTACCGCGATCCCGGCCCGTACAAATTCCCGCAAGGCACCGTCGCC

TACGAAGTGACGCCGCCGGCCGCGGAACCGGCGCGGCAGCAAGGCGGCACGCCGCCGATGAAGAACATGA

AGATGTGA

>gi_222102329_Agrobacterium vitis S4 plasmid_8

ATGTTTAACAGAAGACAAATGCTCGGTGCAGGCGCGGCGCTCGTTTCGACTGCGGCGTGGGCAAAAACGT

CAAACATGGGCCTGCCGGAAGCCGCGCTCATGGAAAATGCCGAAACACAGACGCCAGTCAGACCATCGTC

AGGACCCAACTATACCCCGGTCGTGACGCTGAATGGCTGGACACTGCCTCACCGCATGAACAACGGCGTC

AAGGAGTTCCACCTGGTCGCCGAACCGGTGGAGCGTGAAATGGCCGAGGGGATGACAGCGCATCTCTGGG

GCTACAACGGCCAGTCTCCAGGGCCGACGATCGAAGCTGTCGAAGGCGATCGGGTCCGCATCTTCGTCAC

CAACAAGTTGCCGGAACATACAACCGTCCATTGGCATGGCATGATCCTGCCGTCTGGCATGGACGGCGTC

GGCGGTCTGTCGCAACCCCACATCCCCGTCGGCAAGACCTTCGTCTACGAGTTCGACCTCGTAAAATCCG

GCACTTTCATGTACCACCCGCATTCGGACGAAATGGTCCAGATGGCGATGGGCATGATGGGATTTTTCGT

CATTCACCCCAAGGACGAGACGTTCATGCCGGTGGACCGTGACTTCGTGTTCCTGCTCAGCGCCTACGAC

ATCGATCCCGGAACTTACGTTCCGCGCATCATGGAAATGACGGACTTCAACCTCTGGGCCTGGAATAGCC

GCATCTTCCCCGGCATCAGTCCGCTCGTCGTCTCCAAGGACGACCGCGTGCGCGTCCGTGTCGGCAATCT

GACCATGACCAATCACCCGATCCACATGCACGGTTACGATTTTGAGGTTACCTGCACGGATGGCGGTTGG

GTCAGGCCCGAGGCCCGCTGGCCCGAGGTCAGCATCGACATACCCGTCGGCGCGATGCGTGCCTACGAGT

TTGACGCCAAATACCTCGGTGACTGGGCAATCCATTGCCACAAGTCGCACCACACGATGAATGCCATGGG

ACATGACGTTCCGACCTTCATCGGCGTCGATAAGAAAACGGTCACGGAGAAAATCAGGAAAATCCGCCCG

GAATACATGCCCATGGGTACAGCTGGGATGGCCGATATGGGCGAAATGGAGATGGAGATACCGGAGAACA

CTGTCCCGATGATGACGGGATGGGGAAAGCACGGTCCCATCGAGATGGGCGGAATGTTCTCCGTCGTGAA

GGTCCGCGAGGGTATCGCTGCCGACGATTACACCGATCCCGGCTGGTACGAAAACCCGCCCGGTACGCAG

GCTTGGGAATGGACTGGCGAACTGCCCGATACGACCAAGGCCAAAGACGCAAAAACCCAGATCACGCCAA

AACCAGTGAAACACGGCTGA

>gi_398349897_Sinorhizobium fredii USDA 257 chromosome_9

ATGTCCCCTGGTCTATGCCTTCGCCGCCCAGGCGGCGAGCACCGCCGGAGCGCTTCCCGGGTTCCTCGTC

ATGGCATGGTTCGGACTGGGAACCTTCCCCGCTATGTTGATGATGGGTGGCGTTGGCCGGATACTCGGGC

CAGCCTCGCGGCGGCGCGGCGTGCGGCTGGCCGGTGGCTTTATTCTCTTGCTCGGTCTCATCACGCTCGG

CCGTGGTCTCCTGCCTTCTCACGCGCATATCGGCCTTGCCTGGCTGAACGGACATCCCGCATGACTGCGC

ATGACGACATACTCTGTAGCCACTGTTCACTGCCCGTCGGCCTGCGCCCGATGGAGCGCAGGCTCAACGG

CGAGGCTTGCGCGTTCTGCTGCTACGGCTGCTGCATCGCCTTTCAGGTGAAGAGCGGCAGACACGAAGAG

TGGGAAGCCGCCTGGCTTCTGATCCAGCTCGGCGTGGGAGGCTTTCTCTCCATGAATATCATGATGTTCA

GCCTCCTCCTCTATTCGGACGCGTTCACCGGGGTCGATGCGGGCGTGCTGCCGTGGGTTCACTTTTTGCT

CTGGATCTTCGCGACGCCGGCGGTCGTGATTCTCGGCGGACCTTATCTGCGCGAAATGTGGCTCAATGGC

ATTCAGGGACGAGTGACGTCGTCGGCACTCATCGTGCTTGGCGTCGCTGCCGCGTACCTCTATTCGGCAT

TGGCCGTGTTCGAGGGCAGCACGCATGTCTATTTCGACACCGCCGTGATGGTGCTGATGCTATTCACCGT

TGGCCGCTATCTCGAGGCTGTTGGCCGCGCAAGGGCGGCGCGCGACCTCGAACCTCTTCTGGCCGCAGAA

AGCGAGAGCGCGACCGTGGTCCATGGCGCGGCAGAGATCCGCCGTCCGGTGCGGGAGGTGTCGGCCGGCA

TGCTGGTGCGGGTCCGGCCAGGCGAGCGCATTCCGGTCGACGGTGTGGTTGTCGAGGGGGAATCGAACAC

CGATGAAGCCGTGATTACCGGAGAAAGCCGGCAAGTGACAAAGGGCGCCGGCTCCAAGGTGATTGCCGGT

AGCATCAATATCGACGGCCCGCTCCTGATACAAAGCAGCGGTGACGGAACCGCAACCCGCTGGGCGCGGA

TTTGCCGGTCGGTGCGCGAAGCGCTGGGCCGTCGCAGTTCAACCCAGCGCGTTGCAGACCGCGCCGTGGG

CGTGTCCGTGCCGCTGGTCCTCGCCCTCGGCGGTCTAACGATCGTTTACTGGGCGCAGTCATTGCCGTTC

GATAGGGCGTTGCTGATTGGCCTCTCGGTACTGGTGGTTGCCTGTCCTTGTGCCGTCGGCCTCGCTGCTC

CGATGGCGACTTCTCTCGGCATCGGCCGGCTCGCGCGGCACGGCTGCCTCGTCCGCGACCCCGGCGTGCT

CGAGACTCTCGCGCGTGTACGGTTACTCGCCTTCGACAAGACCGGCACTCTGACTGCGGGCAAAGCGCGT

CTCGTCGGCGTCGACCGCGAGGAGGTCGGCATCGACGAAGTACTGGCGCGGGCCGCCGGTCTGGAGCGGC

TTTCCGAGCATGGTCTGGCGCGGGCTATCGCCGCAGGCGCCGCTCAGCGGGACATCACGCCGCTCGTCAC

CCGCGATGTTCGGACGATTCCCGGCCGTGGCATCCGCGGCAGCGCCGAAGGCCGGCCCGTGGCGGTAGGG

AACGGCAGGTTGATGAATGATCTGGGCTGGCCGCTTTCCCCGTTCCTGACCGAGCGTGCGCGAACGCTGG

AGGCGAGCGGTCATTCGGTGGTCTATGTCGGTTGGGGCGAGCGGGTTCAAGCCGTGCTGTCGCTCGATGA

CTCACCGCTTCCAGAGGCGCAGGGCGCGATCGCGGCCCTGCGTGAGCGCGGGCTCGACGCCACACTGTTG

ACCGGCGACTTGGCGCAAGCGGCGGAGCGGATCGCGGCCCTGGTTGGGATCAAGGACGTCAAAGCGGGCC

TTTCGCCGGAGGCCAAACGGCTGGCCCTGGATCGGCTCCGCCATGACCACGGTTCGGTGGCGATGGTCGG

CGATGGACTGAATGACGGCCCGGTATTGGCCGCCGCAGATGTCGGTATCGCCGTCGGCTCCGCCACCGAT

CTCGCCCGCGAGACTGCGGCGCTCGTGCTGCCTGCGGATGGATTGTGGATGCTGCCCTGGATCGTCGACG

TGGCCCGCGCGGTCCGCAGGACCATCTTGACCAACCTTATGTGGGCGTTCGGCTACAACCTTGTCGCGCT

GACGTTCGCAACATTCGGACTTCTGCAGCCAATCCTTGCGGCGGCGGTCATGGCCGGCTCGAGCATCCTC

GTGGTGGTGAATTCGCTGCGGCTAGAGCGGCTGCCCGATCCTGTTCCAGCGCTGGTCCCGGAGCAACGAT

CCGGCGCCAAGGCGGAGCGTATACACAATGGCACCGGCATTTCCGCAATGGCCGGTCCGGCCGTCGAGCG

GAGTTGA

>gi_383768277_Bradyrhizobium sp. S23321_10

ATGGACAAGGTGGTGATGCAGCCGCCGCTGCACCCGATCAGCGGGCCCGACTATCGCCCGGTCGTCACGC

TGAACGGCTGGTCGCTGCCATTCCGCATGAACGGCGACTGGAAGGAATTCCATCTCGTCGCCGAACCGGT

GGTGCGTGAGTTCGCCGAGGGCATGAAGGTCAATCTGTGGGGCTATAACGGCCAGTCGCCCGGCCCGACC

ATCGAGGCGGTAGAGGGCGACAAGGTCCGCATTTTCGTCACCAACAAGCTGCCCGAATATACCACCGTGC

ACTGGCACGGCATGATCATTCCGAGCGGCATGGACGGCGTCGGCGGACTGACCCAGCCGCACATCCAGCC

CGGCAGGACCTTCGTCTACGAGTTCGAGATGAAGAAGAGCGGGACCTTCATGTACCACCCGCATTCCGAC

GAGATGGTGCAGATGGCGATGGGCATGATGGGCATGGTCGTGGTGCACCCGCGCGACCTGAATTTCCGCG

CAGCCGATCGCGACTTCGTCTTCGTGATGAGCACCTACCGCGTCGATCCCGGCACCTATTTGCCGAAGGT

CAACGAGATGACCGATTTCAACATGTGGACCTGGAATGCGCGGGTGTTTCCCGGCATCGATCCGTTGCCG

GTGCGGCTCGGCGACAAGGTGCGCGTGCGCATCGGCAATCTCAGCATGACCAACCATCCGATCCATCTGC

ACGGCCACAGCTTTGCAGTGACCTGCACCGATGGCGGCTGGATTCCGGAGAGCGCGCAATATCCGGAGAC

GACGACGGACGTGCCGGTCGGCGCGGTGCGTGTGTTCGACGTGCTCGCCGACAATCCCGGCGACTGGGCG

TTCCACTGCCACAAGTCGCACCACACCATGAATGCGATGGGTCACGACATGCGCAATCTGATCGGGGTGT

CGCGCAAAGATCTCGCCAAGGCCGTCGGCAAGCTTGCCCCCGATGGCATGGCGATGGGCTCGACCGGCAT

GGCAATGGGCAACATGGAGATGCCCGCGCCCGACAACACGCTGCCGATGATGAGCGGCACCGGGCAGTTC

GGCCCGATCGAGATGGGCGGCATGTTCACGGTGATGAAGATCCGCGAAGGCCTCGCGCGCGACGATTATC

GCGATCCCGGCCCCTATCAATTTCCGCAAGGCACCGTCGCCTACGAAGTCGAGGCGCCCGATGCGGCGCC

ATTACGGGAGCAGGGCAGGCCCTCGCACAAAATGAAGATGTGA

>gi_549704410_Rhizobium sp. IRBG74 circular chromosome_11

ATGTCACAAATGGAGATCCGGGCGAGCCATCAGATTGCGATCGATGGCATGACATGCGCCTCCTGCGTCG

GCCGCGTTGAAAAGGCGATCGCCAGGGTGCCCGGGGTTCTCAAGGCTTCGGTCAATCTTGCGACGGAACG

CGCTGACATCTCTTTTTCCGGATCACCTGACGTGCTGGCCGTGATCGCGGCGGTGCGGAATGCCGGCTAT

GGCGTCGAAGAAAAAACCATAGAGCTTGATATTGAGGGTATGACATGCGCCTCCTGTGTGGGCCGTGTTG
AAAAGGCCCTGAAGGCTGTTTCCGGTGTTTCGGAAGCGAGCGTCAACCTGGCGACCGAGCGCGCCACCAT
CCGCGTCGCCAGCAATGCCGCCTCTACCGCCACCTTGGGGGAGGCGATCAGACGGGCGGGTTACACGGCG
AAGGAAATCGTCGCCGACAGGGCCGGCAAAGTGGAACAGGACCGACGCGCCGTGGAGTTGCGCAGCCTGA
AAATCAACCTGGCCGTGGCCACTGCTTTGACCCTGCCAGTCTTCGTTCTGGAAATGGGCTCGCATCTTGT
CCCCGCGATCCACGACATCGTGATGGAAACGGTCGGCATGCGGGAAAGCTGGTATCTGCAATTCGTTCTG
ACGACGCTGGTACTCTTTGGACCGGGTCTGCGTTTCTTCAAAAAAGGCATACCCGCACTTCTGCGGCTCG
CGCCGGATATGAATTCGCTTGTCGTGTTGGGCACCGCCGCCGCCTGGGGATTTTCGGTTGTGGCAACCTT
CCTGCCCGAAATATTGCCGCGCGGCACGGCGAATGTCTATTACGAGGCCGCTGCTGTCATCGTCACGCTC
ATCCTCCTTGGCCGGTTCCTTGAAGCGCGCGCCAAAGGCCGCACCAGCGAGGCGATAAAACGATTGCTCG
GGTTGCAGGCCAAGTCGGCGCGGGTGTTACGCGACGGCGAAACCGTGGATGTGCCGTTGCAGGATGTGCG
CGCCGGTGATGTGATCATTGTCCGTCCTGGCGAGAAAGTGCCGGTCGATGGCGTGATCCTCAGTGGCTCG
TCCTATGTCGATGAATCAATGATAACCGGCGAACCCGTTCCGGTTACGAAAACGGCGGGTTCCGAGGTCG
TGGGCGGTACGGTGAACCGCAACGGCTCTTTCACCTTCCGCGCCACCAAGGTGGGCGCCGATACGCTGAT
CGCCCAGATCATCCGCATGGTCGAGGAAGCACAGGCCGACAAGCTGCCCATTCAGGCGCTGGTCGACAAG
GTCACCAACTGGTTCGTGCCGGCGGTGATGCTGGCCGCAGCCGCCACCTTCCTCGTGTGGTTCTTCTTCG
GACCGGATCCGGCTCTGACCTTCGCGCTCGTCAATGCGGTTGCGGTTCTCATCATCGCCTGTCCCTGTGC
CATGGGCCTTGCCACGCCTACCTCGATCATGGTCGGCACTGGCCGCGCCGCCGAGATGGGCGTGCTGTTC
CGACGCGGTGATGCGCTCCAGACGCTCCGCGATGCCGAGGTGATCGCCGTCGACAAGACCGGCACGTTGA
CGCTCGGCAGGCCGAAACTGGTGCATTTTACAACGACCGAGGGCTTCGGTGCCGATGAGGTGCTGCGCCT
TGTGGCATCCCTCGAAAGCCGATCCGAACACCCGATTGCGGAGGCGATCGTCGAAGCCGCGAAACATGGC
GGCCTGGCGCTTGCCGATGCGGAAGGTTTCGAGGCAACGCCGGGTTTTGGCGTGGGGGCGGTGGTTGAAG
GGCGCAGGGTTGAAGCGGGCGCTGACCGTTTCATGGCGAAGCTCGGTTACGATACCGCGATGTTTGCGCA
TGAAGCAGACCGGCTGGGCAGGGAAGGGCAGTCGCCGCTTTATGCTGCAGTCGACGGCAGGCTGGCCGCG
ATTATCGCAGTGGCCGATCCGGTGAAGCAGACGACCCCGGAGGCGATCGCGGCGCTGCATGCGCTCGGCC
TCAAGGTAACGATGATCACGGGTGACAATCGCCGCACCGCAGAGGCCATCGCCCGTCGCCTCGGCATTGA
CGAGGTGGTGGCGGAAGTGCTGCCGGACGGCAAGGTCGAAGCCGTCAAAAGGCTTGCCGCCGGTGGGCGC

CGGGTCGCCTTCGTAGGCGATGGCATCAACGATGCACCGGCGCTTGCGGCAGCCGATGTCGGCCTCGCCA

TCGGAACGGGCACGGATGTCGCGATTGAGAGCGCCGATGTGGTGCTGATGTCGGGCGATCTGCGCGGTGT

GGCCAATGCCATCGCCCTGTCCAAGGCCACGATCCGCAATATCGGCCAGAACCTGTTCTGGGCCTTCGCT

TACAACGCCGCGCTCGTTCCCGTGGCGGCCGGAATATTGTATCCTGTCAACGGCGTGTTGCTGTCACCCG

TCCTTGCTGCCGGCGCAATGGCACTTTCCAGCGTCTTTGTCCTGACGAACGCGCTGCGCCTGAAAAGTTT

CCGTGCGCCGCTCACGGACAGACCCGCGGGCTTGCAGCTCGCAGCAGCGGAATAG

>gi_8719802_Novosphingobium aromaticivorans DSM 12444 chromosome_12

ATGGACAAGAACCTCCTGCTCGACCGCCGCCGCCTGCTTGTCGGCTCCGGCGCCCTGGCCCTCGCGCCGC

TGTTTCCGGCCTGGGCGCAATCCGGGAATCTGGCCGGCCATGGCGGCCATTCCCGTGGCTCCGGCCCGAT

GGCCGGGCGCAACAGCGGGGGGACGCTCTCGGGCGACACCATCGCGCTGAACATCGGCGAGGCGCGCTAT

TCGGTGGGGCGGCGCTCTGCCTCGGCGGTTGCGGTAAACGGCACGATCCCCGCCCCCCTCATCCGCCTGC

GCGAAGGCCAGACGGTTCGCATCGCGGTTACCAACCATCTGAAGGAACAGTCTTCCATCCACTGGCACGG

GCTGCTCGTGCCCTTCCAGATGGACGGCGTGCCCGGCCTGTCGTTCCCGGGCATCGACCCGGGCGAGACC

TTTACCTACCAGTTTCCGATCCTCCACTCCGGCACGTTCTGGTACCACTCCCACTCCGGAATGCAGGAAG

CGGTCGGCCTTTACGGCCCGATCGTGATCGACCCCGCCCTGCCCGATGCCGCCGCACCGTTCGACCGCGA

ATACGTGCTGATGCTGGCCGACTGGAGCCCCGTCGATCCGCATGCCCAGCTCAGGAAGCTGAAGACCATG

GGCGGCTACTACAACTGGCAGCGCCAGACCGTGGGCGGCCTGATTGCCGGCAAGGACCAGAGCCTGAAGG

ACCGGCTCGAATGGGCGAAGATGCGCATGGACGCGACCGACATCTCGGACGTGACCGGCGCCACCTACAG

CTTCCTCGTCAACGGCCACGACACCGAAGCCAACTGGACCGCCCTGTTCCGTCCCGGCGAGCGGGTGAAG

CTGCGGATCATCAATGCCTCGGCCATGACCAACTTCAACTTCCGCATCCCCGGCCTGCCGATGACGGTGG

TTGCCGCCGACGGCTGCAACGTCGCGCCGGTCGAGACCGACGAGATCCAGATCGCCATCGCCGAAACCTA

TGACGTGATCGTCCAGCCCGGCGAGGCGGCACCGTTCGGCATCATTGCCGAAGCCATCGACCGGTCCGGC

CTGGTCCGCGCGACGCTGGCCCCGCAGGCCGGCATGGTCGGCCCGGTGCCGAAGCTGCGCCAGCGCCCGA

TCCTGACCATGCGCGACATGGGCATGGACATGTCGGGCATGGACATGGGCGCGGGCGGCGAGATCGACCT

GTCGAAGCCCGCAAACGAGAGCATGGACGGCCATTCGATGTCGATGCGCGACTTCTCTGCCGCGCCAGGC

GTAAAGAAAGGCCCGGGCGTGGCGACGATATCGCCGATGCCGGCCGACCGGCTTGCCGACCGGGGAACGG

GCCTCGAGAACGAAGACCACCGCGTCCTGACCTATGCCGACCTGCGCTCGCGCGATCCGAACCCCGATCC

GCGCAAACCGACCCGGCAGATCGACGTGAACCTGACCGCGAACATGGAACGCTACATGTGGTCCATCGAC

GGCGAGACCATTTCCGACGGCGCCGCTCCGATCCCCTTCCGCAAGGGCGAACGCGTGCGGGTGAACCTTA

TCAACCACACGATGATGCCGCACCCGATCCATCTCCACGGCCACTTCTTCGAACTGGTGACCGGCGAGGC

GGGCAACCGGCCGCGCAAGCACACCGTCAACGTGCTGCCCGGCGGCAAGGCCAGCTTCGACCTTACGGCC

GATGCCGAGGGAGACTGGGCGTTTCACTGCCACATGCTGCTGCACATGCATTCGGGGATGATGCGCGTCG

TCACCGTCCGCGCCGACAAGGGAGACGCGGCATGA

>gi_33591275_Bordetella pertussis Tohama I chromosome_13

ATGAGCAGAGCAGTTTCGAATCAGCGCCGCCGCTTCGTCCAGGGCCTGGCGGGCGGAGGCGTGCTGGCGG

GCCTGGGGCTGTGGCGCCAGGCCGCCTGGGCCCAGCCCGGCGCGGCGCAGCCCGAAGTGTTGTCGGGCAC

CGAGTTCAACCTGGAGATCGGCCGCACGCCGGCCAACTTCACCGGCGCGGCGCGCATGGCCACGACGGTC

AACGGCAGCATCCCGGCGCCCATCCTGCGCTGGCGCGAGGGCGATACGGTGACGCTGCGCGTGACCAATC

GGCTGGACGAAGACACGTCGATCCACTGGCATGGCATCCTGCTGCCGACCGGGATGGACGGCGTGCCCGG

CCTGAGCTTTCCGGGCATCCGCCCGGGCGAGACCTTCACCTACCGCTTCCAGGTACGGCAAAGCGGCACG

TACTGGTATCACAGCCACTCGGGGTTCCAGGAACAGACGGGCCTGTACGGCGCCATCGTCATCGAGCCCA

GGCGCGCCGATCCGGTGCGAGCCGAGCGCGACCACGTGGTGCTGTTGTCCGACTGGACCGATGAAGACCC

CATGAGCGTGTTCCGCAAGCTCAAGGTCATGCCCGATTACTACAACTACATCCAGCCCACCGTCGACAAC

CTGATCGACGATGCGCGCAAGCAGGGCTGGGCAAGCACGTTGGGCGAGCGCCTGATGTGGCAGCAGATGC

GCATGAACCAGACCGACCTGGCGGACGTCTCGGGCGCGACCTATACCTATCTGGCCAACGGCAAGTCGCC

GGCCGGCAACTGGACCGGGCTGTTCAAGCCGGGCGAGCGGGTGCGGCTGCGCTTCATCAACGGTTCGGCC

ATGACGTACTTCGATGTGCGCATTCCCGGCCTGAAGATGACGGTCGTGGCGGCGGACGGGCTGCCGGTGA

GGCCGGTGGAGGTCGACGAGTTCCGCATCGCCGTGGCCGAGACCTACGACGTGATCGTCGAGCCGGCCGA

GGATCGCGCCTATACGATCTTCTCGCAGGCGATGGACCGCTCGGGCTACGCGCGCGCCACGCTGGCGCCG

CGCGAGGGCATGCAGGCCGAAGTGCCGGCGCTCGATCCGGTGCAGGTGCTCACGATGATGGACATGGGCA

TGGCGCACGACATGCCCGGCATGGACCACGGCGCGGCGGGGCAGCAGGGCGCGGCCATGGACCACGGCGC

CATGGGCGGGATGGACCATGCCGCCATGGGGCATGGCTCGAGCGAGGCGAGCGGGGGCATGGTGGAAGTC

AGGCACCCCTATCCCGCCGAGCACGGCCCGGCCAATTCCATGCTGCCCGATATCGTGACCACGCGGCTGG

ACGATCCGGGCGTCGGTCTGCGCGACAACGGACGCAAGGTCCTGACTTACGCCGACCTGCGCTCCATGGT

CGAACCCGAGGACAACCGCGCGCCGACCCGCGAGATCGAGTTGCACCTGACGGGCAATATGGATCGCTAC

ATGTGGTCGTTCAATGGCCTGAAGTTTTCGCAGGCCAAGCCCATCGTGCTGAAGTACGGCGAGCGCGTGC

GCTTCGTGCTGGTCAACGACACCATGATGACCTACCCCATCCACTTACATGGGTTATGGAGCGATCTCGA

GTCGCCCGACGGCAACTTCCAGGTGCGCAAGCACACGATCAGCCTGAATCCGGCCCAGCGGATCAGCTAC

CGCGTGAGCGCGGACGCCAGGGGGAACTGGGCCTACCACTGCCATCTGCTGTATCACATGGAAGCGGGCA

TGTTCCGCGCCGTCGTCGTGGAGTGA

>gi_56130627_Cupriavidus metallidurans CH34 plasmid pMOL30_14

ATGCGACGTGATCGACTGTCTGGCATCTTGCTGCCCAACCTGCCACGCCGCCGTTTCGTACAAGGTTTGG

CCGCCGGCGGGGTAATGGCGGGGCTGAGTGCTTTGGGCGGAACTGCGTGGGCACAATCATCGGGACTCCC

CGAAAGCGCTTCATCCGGCACCGCCCCGGTATTGACGGGGACGGAGTTTGACCTTGTCATCGCCGAATCG

GTTGTCAACTTTACCGGTACGCCGCGGGTGGCGACAACTATCAATGGGATGCTTCCCGGTCCGACGTTGC

GCTGGCGGCAAGGCGACACCGTGACGATCCGCGTCACGAACCGCTTGCACGAGCACACGTCAATCCACTG

GCACGGCATCATCCTGCCGTTCCAGATGGACGGCGTACCGGGAATCAGCTTTGCCGGTATCGCCCCCGGC

GAGACATTTACCTACCAGTTCAAGGTCGAACAAACCGGCAGCTACTGGTATCACTCGCATTCCGGCTTCC

AGGAAATGACCGGTGTGTATGGCGGCATCGTGATTGATCCGGCCACCGGCGTGGACGGTGTGCGAGCCGA

CAGGGACTACACCATTCTGCTCTCGGACTGGACCGACGAGGACCCCATGCGAGTCCTGTCGAAACTGAAG

GTTCAGAGCGACTACTACAACTACATCAAGCCCACCGTCTTCGATTTTTTCCGCGACGTTTCAAACGACG

GTGTGAAGAGCGCTTTCGAGAAGCGCAAGATGTGGAACGAGATGCGGATGAATCCGACCGACCTGGCGGA

TCTTTCCGGTGCCACACTGACCTATCTGACCAATGGCGTTACCCCCGCTGGCAATTGGACTGGCTTGTTC

AAGCCCGGTGAGAAGGTACGCCTGCGGTTCATCAATGGGTCCGGGAATACGTTCTATGACGTACGTATTC

CCGGCCTGAAGCTCAAGGTCATTCAAGTCGACGGCCAGAACCTGGAACCCGTAAGCGTTGACGAGTTCCG

CTTTGGTCCCGGCGAGACCTACGACGTGCTGGTGGAACCGCGAGACGACGCCTATACGATCTTCTCCCAA

TCCATGGATCGCACTGGCTACGCCCGCGGCACACTCGCGGTGCGCGCAGGACTCAACGCCCAGGTGCCGG

CCGTGGACAAGCCCGAGTGGCTCACCATGAGCGACATGATGGGTGGCATGGGTGGCATGGGTGGCATGGA

CCACAGCAAGATGGACATGGGCGGCATGAACCAAGGCAGCATGGCTGGCATGAACCATGGGGCCATGACC

ATGGACCATAGCCAGCACGCGATGGGCGGCATGGCCATGGATGCTTCGCTCAAGGTCCCCAGCACGAAGG

CTCGGCACGCCAAGACCGAATATGGCGCGAACACGGACATGCGCGTGGACATGGCCAGAACGAATCTCGA

CGACCCTGGCATCGGTCTGCGTAACAACGGCCGCCGCGTTCTGACGCTGGCCGATCAACATACCATTGGC

GGCCCCCTCGACAAGCGCGGTCCTGCTCGGGAAGTTGAACTGCACCTCACCGGCAACATGGAACGCTACT

CCTGGTCAATCGATGGTGTTGAGTACGGCAAATCCACACCGGTCCGTTTCAAGTACGGTGAACGACTGCG

CGTGATTCTGCACAACGACACGATGATGACTCACCCGATGCACCTGCACGGCATGTGGAGTGAGCTCGAG

TCGCCTGACGGTTCGTTCCAGGCCCGGCGACATACCATTCCGGTCCAACCTGCGCAGCGGATCAGCTTCC

TGGTAACCGCCGACGCATTGGGCCGCTGGGCATGGCACTGCCACCTTATGTTGCACATGGATGCCGGGAT

GTTCCGCGAAGTACTTGTGGCCTGA

>gi_300693042_Ralstonia solanacearum PSI07 megaplasmid mpPSI07_15

ATGCGCATGAATCATGCATCCGGCCTGCTGCTGCCGAGCCTGCCCCGGCGGCGGTTCGTGCAAGGGCTGG

CGGCCGGCGGGGTGATCGCCGGGCTGGGGCTGGGCGGCATCGCCCCGTCGTCGGCGGCGGCCGGCGCCAC

CGCGCTCGGCACGGCCCCGGTTCTGCGGGGGACCGAATTCGACCTCGTGATCGACGAGACCCCGGTCAAC

TTCACCGGCAAGCCGGCCATGGCCACCACCATCAACGGCATGCTGCCCGGGCCCACGCTGCGCTGGCGCG

AGGGCGACACGGTCACCCTGCGCGTCACCAACCGCCTGCGCGAACCCACCTCGATCCACTGGCACGGCAT

CCTCCTGCCGTTCCAGATGGACGGCGTGCCGGGCATCAGCTTCCACGGCATCGCCCCCGGCGAAACCTTC

ACCTACCGGTTCAAGGTCCGGCAAAGCGGCAGCTATTGGTATCACTCGCACTCCGGCTTCCAGGAGATGA

CCGGCGTGTACGGCGGCCTCATCATCGACCCCGCCGGCGGCGACCCGATCCGCGCCGACCGCGACTACAC

CGTGCTGCTCGCCGACTGGACCGACGAGGACCCGATGCGGATCCTCTCCAGGCTCAAGACCCAGAGCGAC

TACTACAACTACCACCAGCCCACGGTGGTGGATTTCTTCCGCGATGTCGCCAACGACGGCTGGCAGGCTG

CGCTCGACAAGCGCCGCATGTGGAACCAGATGCGGATGAGCCCGACCGATCTGGCGGACCTGTCCGCGGC

AACGCTGACCCACCTCACCAACGGCGTCACGCCGGCGGGCAACTGGACCGGCCTGTTCTCCCCGGGGGAG

ACCGTGCGGCTGCGCTTCATCAACGGCGCCGGCAACACGTTCTATGACGTCCGCATGCCGGGGCTCAAGC

TCAAGGTGGTGCAGGTCGACGGGCAGGACATCGAACCGGTCACGGTCGACGAATTCCGCTTCGGTCCGGG

CGAGACCTGCGACGTGCTGGTGGCGCCCAAGGACGACGCCTATACGATCTTCTGCCAGTCGATGGACCGC

ACCGGCTACGCGCGCGGCACGCTGGCCGTGCGCCCCGGCATGCAGGCCGCGGTGCCGGCCCCGGACAAGC

CCCAGTGGCTCACCATGACCGACATGATGGGAGACATGGGCGGCATGGCGGGCATGTCCGGCATGGACCA

CGGTGGCATGACCATGATGGACGCGGGCGACATCACGCCGATGGCACCGATGGACACCGGCGGCATGACC

ATGATGGACGGCACCGGCGGCACGGACGACCTGCGGGTGCCGGGCAAGAAGGTCCGCCACGCGCGCACCG

AATACGGCCCCGGCACCGACATGCGCGTCGACACGCCGCGCACCAACCTGGACGATCCCGGCATCGGCCT

GCGCGGCAACGGGCGGCGCGTCCTGGTGCTGGCGGACCAGCACACCATCGGCGGCCCGCTCGACCGGCGC

GGGCCGCGGCGCGAGGTCGAGATGCACCTGACCGGCAACATGGAGCGCTACACCTGGTCGATCGACGGCG

TGGAGTATGGCCGCTCGACGCCCATCCACTTCCGCTATGGAGAACGGCTGCGCGTGATCCTGCACAACGA

CACGATGATGACGCACCCCATGCACCTGCACGGCATGTGGAGCGAACTCGAGGCCCCGGACGGGCGCTTC

CTGGCGCGCCGGCACACCCTGCCGGTCCAGCCCGCGCAGCGCATCAGCTTCCTGGTCACGGCGGATGCGC

TGGGCCGCTGGGCATGGCATTGCCACCTGATGCTGCACATGGACGCGGGCATGTTCCGCGAGGCGGTGGT

CTCGTGA

>gi_340785197_Collimonas fungivorans Ter331 chromosome_16

ATGGTGTCACGCAGAAATTTCATGAGCGGCGCAGGCGCCGTCGCCATCGGCGCCAGCCTGGTCAGCCGGG

CCGGTGCGGCGATGCTGCCCGAAGCCGTAACGATGACTGCCGCGGCCACGCAGCCACCGCTTTCTCCACC

CAATGGGCGGCCCTACAATCCGGTGGTCACCCTGAACGGCTGGACCCTGCCCTGGCGCATGAAGAACGGC

GTCAAGGAATTCCACCTGGTGGCGGAACCGGTGCTGCGCGAAATCGCGCCGGGCATGAAGGCCAACCTGT

GGGGTTACAACGGCCAGTCGCCCGGTCCTACCATCGAGGTGGTCGAGGGCGATCGCGTGCGCATCTTCGT

CACCAACAAACTGCCGGAACATACCAGCGTGCACTGGCACGGCCAGCGGCTGCCGAACGGCATGGACGGC

GTCACCGGCCTGACGCAGCCGGGCATCCAGCCGGGCAAGACCTTTGTCTATGAATTCGTCGCAAAACGCC

CCGGCACTTTCATGTATCACCCGCATGCCGATGAAATGACGCAGATGGCGATGGGCATGATGGGCTTCTG

GATCACTCATCCGAAAGACCCCAGCCAGCACCGCGCCGACCGCGATTTCTGTTTTTTGCTCAACGCCTAT

GACATCGATCCCGGCAGCTACACCCCGAAAACCAGCACCATGCTCGATTTCAACCTGTGGACTTTCAACA

GCCGCGCGTTCCCGGGCATAGACCCGATGGTGTGCAGCCAGGGCGACCGCGTGCGCATCCGCGTCGGCAA

CCTGACCATGACCAACCACCCGATCCACCTGCACGGCCACGAATTTGTGGTCGCCGGCACCGACGGCGGC

TGGACGCCGCCGGCATCGCGCTGGCCGGAAGTCACCACCGATATCGCCGTGGGCCAGATGCGCGCCATCG

AATTCGACGCCACCGAACTCGGCGACTGGGCCTTCCATTGCCACAAATCGCACCACACCATGAATGCGAT

GGGCCATGAGGTGCCGACCATGATCGGCGTCGACCAGCGCGACGTCCTTGCAAAAATCAACCAGCTGGTG

CCGGACTACATGGCGATGGGAGAAACCGGCGGCGCCATGGGCGAGATGGAGATGCCGCTGCCGGACAACA

CCTTGCCGATGATGACCGGCAGCGGCCCGTTCGGCGGCATCGAGATGGGCGGCATGTTCACCACGGTGAA

AGTGCGCAAGGGCCTCGCCCGCAACGATTACAAGGACCCTGGCTGGTACAGGCATCCCGCCGGCACCGTT

GCCTATGAATGGAAAGGCAGCGACGCATCCATGCCGGCCGCCGCCCGCGCACCGGACGCCGGCGGCATGG

CCGCCAGGCCGGGCGAAAAGGTCCTGAAGGTGATCAAGCCTGGTTCGCAAGACAAGCACTAA

>gi_410692016_Thiomonas sp. 3As_17

ATGCAACGCAACCTCCTCATCCGACCAGAAGGTGTCCATGTTCAACGGCGGCGCTTCGTGCAAGGGTTCG

CTGCTGGCGGCGTCATGCTGGGGCTGGCCCCGTGGGCGGCGCGCGCGCAAACGGCGCCCACCGCAACCGG

GACGGCCCCAGATCTGCGGGGTCGCGAATTCGATCTGGTGGTGGCCGAGACACCGGTCAATTTCACCGGA

AAACCTCGGGTCGCGACGACGATCAACGGATCGATTCCAGGGCCGACGCTGCGTTGGAAGGAAGGCGACA

CGGTGACGATTCGTGTGACCAACCGTATGAGCCGCTCGACGTCGATCCACTGGCACGGCATGCTCGTGCC

CTTTCAGATGGATGGCGTGCCTGGAATGAGCTTCAAGGGCATCGCGCCCGGCGAGACCTTCGTTTATCAC

TTCAAGGTTCAGCAGGCCGGCACCTACTGGTACCACTCGCACTCGGGCATGCAGGAGCAAACCGGCATGT

ACGGCGCCATCATCATCGAGCCCGCGCATGGCAAGACCATACACGCCGAGACCGATCATGTGGTGCTGTT

GTCCGACTGGATGGACGAAGACCCGATGCGCGTCCTGGCCAAGCTCAAGGACGACAGCGGTTTTGACAAC

TACCATGAGCCGACGGCGGTGGACTTCTTTCGCGATGTCTCCCGCGAGGGACTGCGTGCGGCACTCGACA

AGCGCGCGATGTGGGAGCAGATGCGCATGAGTCCGAACGACCTCTCGGATCTTTCGGGCGCGACGCTCAC

CTACCTGATGAACGGCGTCACCCCGGCGGGCAACTGGTCGGCGCCCATCGTGCCGGGCAAGCCCGCCAGG

CTGCGCTTCATCAACGGCGCGGGCAACACGTTTTACGACGTGCGCGTCCCTGGCCTGAAGCTCACCGTGG

TGGCGGCCGATGGGCAGGATGTGGAACCGGTCAGCGTGGACGAGTTCCGCTTCGGTCCCGGGGAAACCTA

CGACGTGCTGGTGCGCCCGCAAAACTCGGCCTACATCATCTTCGCACAATCGATGGACCGCACTGGTTAT

GCGCGCGGCACGCTGGCTGCACGCCCGGGTCTCGCCGCGCCCGTTCCGCCGCTGGACCCCCGCCAGCAAC

TGACCATGGCCGACATGATGGGCAACGACATGAGCTCGATGCCAGGAATGGACATGGGGGGGATGAGCAT

GGACACGGGCCCCGCCGTGCGACACGCACAGACCGAGTACGGTCCCGGCGTCGACGCCCGCGTCGATCAC

CCACGGACCAATCTGGACGATCCCGGTGTGGGCCTGCGCAACAACGGCAGACGCGTGCTGACTCTTGCTG

ATCTGCACACGATCGGCGGCCCGATCGATCCGCGTGCGCCAGGTCGCACGATCGAGATGCATCTGACGGG

CAACATGGAGCGCTACACCTGGTCGTTCGACGGCGTGCCGTTCGGGGAGTCGACACCGGTGCATTTCCGC

TATGGCGAGCGCCTGCGCATGGTGTTGGTGAACGACACGATGATGACCCACCCGATGCACATGCACGGCA

TGTGGAGCGAACTGGAGAGTCCGGATGGCCAATTCCAGGTGCGCCGCCACACCATCCCGGTCCAGCCGGC

GCAGCAGGTCAGCTATCGCGTCAGCGCCGACGCGCTGGGGCGTTGGGCCTGGCACTGCCATCTGATGATG

CACATGGATGCGGGGATGTTCCGCGAGATTCTGGTGTCGTGA

>gi_30248031_Nitrosomonas europaea ATCC 19718 chromosome_18

ATGACATGCGCTGCATGCGCTGCCCGGATCGAGAAAAATCTCAACAAACTGCCGGGCGTGCATGCAGCTG

TCAATTTTGCCAATGAGAAAGCCCTCATAAAGTTCGATCACGATTCCACCCACCCTGAAGAGCTGGTTCA

ATCCATCGAAAAGGCAGGATTCCAGGTCCCGGAACAGACCGTACAGCTGCAAATCAGCGGCATGACTTGT

GCCGCATGCGCAAATCGTATCGAAACTGTCCTGAATGAGATACCCGGAGTCAGAGCCATATTGAATCCGG

CTGCAGAAATTGCTTATATCAGCTTCAATCCCGCCATTACTTCGGTCGAGCAACTGGTTTCTGCTGTAGA

AAAGGCAGGTTATGGCGCTAATCAGATCAGTGATGACAATTACGTTAAAGAACAATCCCGCAATCAAGCT

GCCTACCGGAAAGAACTCCGTATATTCTGGATCTCTGCCGCCCTGACTGTACCCTTCATGCTGGAAATGA

TCATGATGCTGACGGGTAATCACAACAACCTGCTCCCTTACTGGCTGCAATGGCTGCTTGCAACGCTGAT

CCAGTTTTGGCCCGGCAGGCGTTTCTATATCAGCGCCTGGCGCACACTGCGTGGCGGAGGAGCCAATATG

GATGTACTCATCGCACTGGGTACCAGCATGGCCTATCTCTTCAGCACCGCCGTCATCATTTTGCAACTGG

ACCAGCATGTTTACTTTGAGGCCAGCGCGATGATCATCACGCTCGTGCTTCTGGGCAAGCTGATGGAAAC

CAGAGCAAAAAGAAAAACTTCAGCAGCCATTGAAACACTCATCAAACTTCAGCCCAAAACGGCCAGAGTC

GAACGTGATGGGGAAATTATCGAGATCGATATCAATAGCCTTAAGAATGAGGATATTTTTCTTGTCCGCT

CGGGCGAAAGCCTGCCCGCCGATGGCATCGTCATTGAAGGATCTTCCAGCATAAACGAAGCCATGTTGAC

AGGTGAAAGCCAGCCGGTTACCAAGCAGGTGGGCGCAAAGGTTTATGCCGCTACCCAGAATCAGCATGGT

TTACTCAAATGCCGTGTAACGGGTGTCGGTAAAAATACCCAGCTTGCAGCCATCATTCGTCTGGTTGAGA

TTGCACAGGGCTCTAAAGCACCTATCCAGCGTATGGCAGATACTGTCTCTGGTATTTTTGTACCAATTGT

AATTGGTATCAGCATTCTGACATTGGGACTAACCTGGTGGCTTACAGGCCATTTCGTCATAGCACTCATC

AATGCAGTAGCAGTACTGGTTATTGCTTGCCCTTGCGCACTGGGTCTGGCCACTCCTACTGCAATCATGG

TTGGCACAGGAAGAGGAGCACAAGAAGGAATTCTGGTAAAAAACGCGACCGCACTGGAACTCGCAGAAAA

AATCCAGATGCTTGTTGTAGATAAAACAGGGACGCTGACCGAAGGCCATCCATCAGTCACAGATATTATC

GTGGCCGATGAGGTGAATGAGCACGATTTATTACAGATTGCAGCTTCACTGGAACAAGGCTCTGAACACC

CCCTGGCAAAGGCGGTATCGCAATATGCTTCTTCATCAAAAATCAGATTATTGGCGATAACCAATTTTGC

CTCTGTGACCGGCAGCGGAATCAAAGCGGATATCGACAATGCATCTTACATTCTTGGTTCACCCAAATTT

TTGGCAGAAAAAGGTGCTGTGCTCGATCAGCAGCGTATCACTGCACTCCAGACAGAAGGTAAAACCGTCG

TGGGTGTCGCTATCCAAATTAATGAATCAGTCCGGGTAATCGGCTATCTTGCCATTGCGGATTCACTACG

CGAAACTTCCATCAAAGCAATTCAACGCATCCAGAATCTGGGCATAGATGTCATGATGCTGACAGGTGAC

AACCCGACTACGGCTGCCGCCATCGCAAAACAGGCGGGAATAAAAATTTTTCATGCAGAAGTTTCGCCTC

AGAATAAAGCAGCCGAAATCGAAAAAATCAAGGCAAATGGCCAGTTTGTCGGGATGGTAGGAGATGGAAT

CAATGATGCACCTGCACTGGCAGCCGCGAATGTGAGTTTTGCGATTGGTGCTGGATCAGATATAGCAATT

GAAGCCGCTGACATTACATTGATGCGCAATGATCTGATGAGTGTTGCGGATGCCATCTCACTATCGCGCA

GCACACTGCACAAAATCCGGCAGAATCTGTTTTTTGCATTCTTTTATAATATTTTAGGTATTCCACTGGC

AGCAGCAGGTATGCTAAGCCCCGTAATTGCAGGTGCAGCAATGGCAATGAGTTCGGTATCAGTCATTACC

AATTCATTACTGTTGAAACGCTGGCGTGCAGGCGTTTAA

>gi_330822653_Alicycliphilus denitrificans K601 chromosome_19

ATGCGATTCAAAACCCCTTCCTTTTCCGTCTCCCCAGGCCAGGCAATCCAGCGCCGGCGCTTTCTGAGGG

CGCTGGCGGCGGGTACCGCATTGGGCACGCTCGATGCCGCACTGCCCGCCTGGGCCCGGCAAGACCTGCC

GGTAGCCCATACGGGAACACCGCCCGAGTTGCGTGGGCCCGTTTTCGACCTGACGTTGACCGAGACCCGC

GTCGATTTCGACGGCCGAACGGGCATCGCCACCACCGTCAACGGCAGTCTGCCCGCCCCGACGCTGCGCA

TGCGTGAAGGCGAGACGGTGACGATTCGCGTCACCAACCGCCTGCGCGAGACGGCTTCGATCCACTGGCA

CGGCATCATCCTGCCATTCCAGATGGACGGCGTGCCGGGCGTCAGCTTTGCCGGCATCGCCCCGGGCGAG

ACCTTCACCTACCGCTTCACGCTGGAGCAAAGCGGCACCTACTGGTACCACTCGCACAGCGGCATGCAGG

AGGTGACCGGCCTGTATGGCGCGCTCATCGTCGAGCCGCGCGGGGGCGAAGCCATACGCGCCGAGCGCGA

ACATGTGGTGCTGCTGTCGGACTGGACCGGCAGCGACCCGATGCGCGCCCTGGCCAAACTCAAGGCCCAG

GGCGACGTGTTCAATCAGCATCGTCCGACCTTCGTCAAATTCATGCAGGACGTGCGCGCCAAGGGCATGC

ATGCGGCCATGGCGGAGCGCGACATGTGGAACGCCATGCGCATGAGCCCGAGTGACCTGGCCGACCTGTC

ATCCACCGTGCTCAGCTACCTGGTCAACGGCCGCACGCCGGGCGCCAACTGGACGGCCCTGTTCAATCCC

GGCGAGCGCGTGCGCCTGCGCTGCATCAACGGCAGCGGCAATACGTTCTATGACGTGCGCATTCCGGGCC

TCAAGCTCACCGTGGTGCAGGCCGACGGGCAAGACGTGGAGCCAGTGACGGTGGATGAGTTCCGCTTCGG

CCCCGGCCAGACCTACGACGTGATCGTGCAGCCCAAGGACGATGCCTACACGCTCTTTGCGCAATCGATG

GATCGCACCGGCTACGCGCGCGCCACACTGGCGGTACGCCCGGGCCTGAGCGCGCCGGTGCCCGCGCTGG

ATGCGGTGCAGTGGCTCACCATGGCCGACATGATGGGCGGCATGGCGCAAGGCGGTGGCCACGACATGGC

GGCGATGGGCCACGGCTCCATGGTCGGGATGAACCATGCTGGTATGGCCAGCATGCAGGGCATGGGCGGC

ATGCCCGGCATGGCGTCCAATCCGTTGCAAAAGCCCGGCACCACCGTGCGCCACGCTCGCACCGAGTGGG

GCCCCGGCGCCGACATGCGCGTGGACATGCCGCGCACCAACCTGGATGACCCCGGCGTCGGTCTGCGCGA

CAACGGCCGGCGCGTGCTCACGCTGGCCGATCTCAAGACCGTCGGCGGATCGCTCTATGCCGGCGGGCCC

GAGCGCGAAATCGAGTTGCATCTGACGGGCAACATGGAGCGCTACACCTGGTCGTTCGACGGCCTGGACT

TCACCGACAGCCGCCCCGTGCATTTCCGCTACGGCGAGCGCCTGCGCGTAATCCTGCAAAACGACACCAT

GATGACCCACCCCATGCACCTGCACGGCATGTGGCAGGAGCTGGAAAACGCCTCGGGCGAATTCGTCGCG

CGGCTGCACACCATCCCCGTGCAATCGGCGCAGCGCGTGAGCTTTCTCGTCAGTGCCGACGCACTCGGCC

GCTGGGTCTGGCACTGCCACCTGATGTTCCACATGGAAATGGGCATGTTCCGCGAAGTGGTGGTGGCATG

A

>gi_74316018_Thiobacillus denitrificans ATCC 25259 chromosome_20

ATGAACCCTCATTCTCGATTCTTGCCTGCAACGCCGAATCTCCCGCGCCGGCGGTTTCTCCAGGGACTGG

CGGCCGGCGGCATCTTGCTCGGTGCGCCCTCCTTGATACTGCCGGCCTCGGCACGGGAAGCCGGCACGGC

CGCAGGCAGTGCGCCGGTACTCAGCGGCACCGAATTTGATCTCACGATCGCGGAGACGGCGGTGAACTTC

ACCGGTAAACCGCGTATGGCGACGACGATCAACGGCTCGATTCCCGGGCCTACGCTGCGCCTGCGCGAGG

GCGACACCGTGACCATCCGCGTACACAACCGGCTGACCGAGGACACGTCGATCCACTGGCACGGCATCCT

GCTGCCCTTCGAGATGGACGGCGTGCCGGGCATCAGCTTCAAAGGAATTCCGCCCGGCCAGACCTTTACC

TACCGCTTCAAGGTGAAGCAAACCGGCACCTATTGGTACCACTCGCACTCCGGCATGCAGGAGCAGACCG

GGATGTACGGCACGATCATCATCGACCCCGCGCGTGGTAACTCCGTCGGCGCCGATCGCGATTACGTCGT

GCAATTGTCGGACTGGACCGACGAGCATCCGATGCGGGTGCTCCACAAGCTCAAGGTGCAGGGCGACTAC

TTCAACTTCAATCAGCCGACCGCCGGGGACTTCTTCCGCGACGTGTCGAAGGAAGGGCTCAAGGCCGCGA

TCGACAAGCGCCGGATGTGGAACGAGATGCGCATGAGCCCGACCGATCTGGCCGACGTCTCCGCTCACAC

CTATACCTATCTGATGAACGGCGCGGCGCCGGCCGGCAACTGGACCGGGCTGTTCCGCCCCGGCGGGAAG

GTGCGATTGCGCTTCGTCAACAGTGCAGCGATGACCTTCTTCGACGTGCGCATTCCGGGACTCAAGATGA

CCGTCGTGCAGACCGACGGGCAGGATGTCGAACCGGTCACGGTCGACGAAATCCGCATCGGCGTCGCAGA

GACCTACGACGTGATCGTGAGCCCCAAGGACGAGGCGTATACGATCTTCGCGCAGTCGATGGATCGGACC

GGCTTCGCGCGCGGCACGCTCGCGACCCGTGCGGGACTTGTCGCGGCGGTTCCGTCACCGGACAAGCCCG

TGGCGCTCGAAATGCAGGACATGATGGGCGACATGACCGGCCTCGCGCGGGCCCACCATGCCCGGACCGA
GTATGGCCCCAGTGTCGACATGCGCGTCGACACCCCGCGCACCGACCTGGACGACCCCGGCGTCGGCTTG
CGCGACAACGGCCGGCGCGTGCTGACCTATGACCATCTCCACAGCGTCGGCGGGCCGCTGGACGCGCGGG
GCCCCGGACGCGAGATCGAGCTCCATCTCACCGGCAATATGGAGCGCTACGCCTGGTCCATTGACGGCGT
TGAGTTCGAGAAGTCCACGCCGGTGCATTTCCGTCACAACGAGCGGCTTCGCGTGGTCTTCGTCAACGAT
ACGATGATGACGCACCCGATGCACTTGCACGGGATGTGGAGCGAACTCGAAAGTCCGGAAGGGAAGTTCC
AGGTGCGCAAGCACACGATCGCAGTACAGCCCGCGCAGCGCGTGAGCTTTCTGGTCACCGCCGATGCCCT
AGGGCGTTGGGCATGGCACTGCCATTTGGGGCTGCACATGGACATGGGCATGTTCCGCGAAGTCGTCGTG
GCCTGA
>gi_543961847_Sulfuricella denitrificans skB26_21
ATGCTCGGCCTGATGTCGGTTGTCAAACCAGTCTGGGCAAGCATCGCAGACCCAGTCTCAAGCCGCGTCC
CGGTGCTCACCGGAACCGAGTTTGATCTTGTTATCAGCGAGTCCCCGGTTAATTTCACTGGAAATCCGCG
CATGGCCACCACTGTCAACGGCTCGATCCCGGCACCCACGTTGCGCTGGCGGGAAGGCGAAACAGTGACG
ATCCGTGTGACCAATAAACTGCGCGAAACCACTTCCCTCCACTGGCACGGCATCATTCTACCGTACCAAA
TGGATGGTGTGCCGGGCATCAGCTACAAGGGAATCGCCCCAGGGGAAACCTTCACCTACCGCTTCAAGGT
GGTGCAAAGCGGCACCTATTGGTACCACTCCCATAGCGGTTTTCAGGAACAGACCGGCATGTATGGAGCC
ATCGTCGTTGAACCGGCGGGAGGCGAGAAAATCCGTGCCGATCGCGACTATGTGGTGCAACTGTCCGATT
GGACCGACGAAGACCCGATGCGGATTTTTGCCAAGCTCAAGATGCAGAGCGACTACTATAATTTCAACCA
GCCTACCGTCTCGGATTTCTTTCGCGATGCCTCGCGCAGCGGGTTCAAGACTGCGTTCGAAAAACGCAAG
ATGTGGAACGAGATGCGCATGAGCCCCGCCGATCTGGCGGATATTTCTGGTTACACCTACACCTTCCTCA
TGAACGGCAGCACCCCGGACGGCAACTGGACAGGGTTGTTCCAGCGTGGCGAGCGCGTGCGTTTGCGTTT
TATAAGCAGCGGCACCCAGAGCTTCTTCGATGTGCGTATCCCGGGCCTGAAAATGAAAGTGGTTCAGGTT
GACGGCCAGGATATCGACCCTGTAACGGTGGACGAATTCCGTTGCGGCCCAGGGGAAACCTATGACGTGA
TCGTCGAGCCCAATGACGACGCCTACACGATTTTTGCCCAGTCCATGGATCGCACCGGTTTTGCCAGAGG
GACGCTCGCTGTGCGCGAAGGGCTTACCGCGACCGTGCCCGAACTAGACAAAGCCGAATGGCTAACCATG
ACGGACATGATGGGCGCCATGGGTGGCATGAACCACGGCGGCATGGCCATGGACCACAGTCGGCACGTCA
TGCCGGGGATGGCGATGTCAGGTGGGTTGGCCATACCAAGTAGCCAGGTGCGCCACGCGAAAACTGAATA

TGGTCCCAGCACCGACATGCGGGTCGACATGGCGCGCACCAATCTGGATGACCCCGGCATCGGACTGCGT
GACAATGGGCGCCGAGTATTGACACTGGCCGATCTTCACACGATTGACGGCCCGCTCGACATGCGCGGCC
CCGAGCGCGAGATCGAGCTGCATCTGACCGGCAACATGGAGCGCTACACCTGGTCGCTAGACGGCCTCGA
GTTCGGAAAATCCACTCCCGTGCATTTCCGGCACGGCGAGCGGCTAAGAGTCATCTTGCACAACGACACC
ATGATGACCCACCCCATGCACCTGCACGGCATGTGGAGCGAGCTGGAAAGCCCGGAAGGCCACTTTCAGG
CGCGGCGTCACACGATTCCCGTGCAGCCCGCGCAACGCATCAGTTTCCTCGTTACTGCCGACGCGCCGGG
ACGCTGGGCTTGGCACTGCCACCTGCTCTATCACATGGACGCCGGCATGTTCCGCGAAGTGGTGGTGGCT
TGA
>gi_311103224_Achromobacter xylosoxidans A8 chromosome_22
ATGAGTAGAGCAGTTTCGAATCCGCGCCGCCGGTTTGTGCAGGGCCTGGCCACGGGCGGCGCGCTGGCCG
GCATGGGCCTGTGGAGTCCGTCGTCGTGGGCGCAGGCCGCCCCGGCGCGGCAGAGCGTGTTGTCGGGCAC
CGAGTTCGACCTGGAGATCGGCGAGAGCCCCGCCAACTTCACCGGCGCGCCCCGCATCGCGACCACGGTC
AACGGCATGCTGCCCGCGCCCACGCTGCGCTGGCGCGAGGGCGACCGCGTCACGCTGCGCGTCACCAACC
GCCTGCGCGAGGACACCTCGATCCACTGGCACGGCATCATCCTGCCCACCGGCATGGACGGCGTCCCCGG
CCTGAGCTTTCCCGGCATCGCCCCCGGCGAGACCTTCACCTACCAGTTCGACGTGGGCCAGAGCGGCACG
TACTGGTACCACAGCCATTCCGGCTTCCAGGAACAGACGGGGCTGTATGGCGCCATTGTGATCGATCCCA
AGCGCCCCGACCCGGTGCGGGCCGACCGCGACTATGTCGTCCTGCTGTCCGACTGGACCGACGAAGACCC
CATGAGCGTGTTCCGCAAGCTCAAGGTGATGCCCGACTACTACAACTACATCCAGCCCACCGTCGGCAGC
CTGATTGACGACGCGGAGAAGTCCGGCTGGAAGAACGCGCTGAACGAACGTCTCATGTGGCAGAAGATGC
GCATGAACCAGACCGACCTGGCCGACGTCTCGGGCGCCACCTACACCTTCCTTACCAACGGCAAATCTCC
TGCGGGCAATTGGACCGGCCTGTTCCGTCCGGGCGAGCGCATCCGCCTGCGCTTCATCAACGGGTCGGCC
ATGACCTACTTCGATGTGCGCATCCCCGGCCTGAAGATGACGGTGGTGGCCGCCGACGGCCTGGCCGTGC
GGCCCGTGGAGGTGGAAGAGTTCCGCATCGCCGTGGCCGAGACCTACGACGTCATCGTCGAACCGCAGGA
CGAGCGCGCCTACACCATCTTTTCGCAGGCCATGGACCGTTCCGGCTATGCCCGCGGCACGCTGGCGCCG
CGCGAAGGCATGCAGGCCGAGGTGCCGGCGCTGGACCCGGTGCAAGTGCTGACCATGATGGACATGGGTA
TGGCGCACGACATGCCCGGCATGGACATGGGCGGGGGGCAGGCAGGGGGCATGGACCACGGCGCTCAAGG
CGCGATGAACCATGGCGCGCAGGGCGGCATGAGTCAAGGCGCCATGGGTGGGATGAACCACGCCGGTATG

GCTGGAATGGCGGGCATGAATCACGGCGGCATGGACCATGGCGCCATGTCCGGCAGCCCAGGCGCCAGCG

AAGGCGGCATGGTCGAGGTCAAGCATCCCTACCCCGCCGAGCGCAGTCCCGCCAACTCCATGCTGCCCGA

CATCGTATCGACCCGCCTGGATGATCCCGGCGTGGGCCTGCGCAACAACGGCCGCCGCGTGCTGACCTAT

GCCGACCTGCACTCGATGGTCGAACCCGCGGACAACCGGCAGCCGACCCGAGAAATCGAGCTGCACCTGA

CCGGCAACATGGACCGCTACATGTGGTCGTTCAACGGACTGAAGTTCACCGAGGCCAAGCCCATCGTGCT

CAAGTACGGCGAGCGCGTGCGCTTCGTGCTGGTCAACGACACCATGATGACGCACCCCATCCACCTGCAC

GGCTTGTGGAGCGACCTCGAGTCCGCCGACGGCAATTTCCAGGTGCGCAAGCACACGATCAGCCTGAACC

CGGCGCAGCGCCTCAGCTACCGCGTCAGCGCGGACGCGCGCGGCAACTGGGCCTATCACTGCCACTTGCT

GTTCCACATGGAGGCCGGCATGTTCCGCGCGGTCGTCGTGGAATAG

>gi_388476123_Escherichia coli str. K-12_23

ATGTCACAAACTATCGACCTGACCCTGGACGGCCTGTCCTGCGGTCACTGCGTTAAACGCGTGAAAGAAA

GTCTTGAACAGCGTCCGGATGTTGAGCAGGCGGATGTGTCTATCACTGAAGCGCACGTTACCGGGACTGC

CAGTGCAGAACAGCTGATCGAAACCATCAAACAAGCGGGTTATGACGCATCTGTAAGCCACCCAAAGGCT

AAACCGCTGGCGGAGTCATCAATCCCGTCGGAAGCACTGACAGCGGTTTCTGAGGCGCTTCCGGCAGCGA

CCGCCGATGACGATGACAGCCAGCAGTTGCTGCTGAGCGGCATGAGCTGCGCCAGCTGTGTCACCCGCGT

ACAAAATGCGCTGCAAAGCGTACCGGGCGTCACTCAGGCACGGGTAAACCTGGCGGAGCGTACTGCGCTG

GTGATGGGCAGTGCCTCCCCACAAGATTTAGTGCAGGCGGTGGAAAAAGCGGGCTACGGCGCGGAAGCGA

TTGAAGATGACGCTAAACGCCGCGAGCGCCAGCAAGAAACCGCCGTCGCTACGATGAAGCGCTTCCGCTG

GCAGGCAATTGTCGCACTGGCGGTGGGTATCCCGGTGATGGTCTGGGGGATGATCGGCGATAACATGATG

GTCACCGCTGACAACCGCAGCCTGTGGTTGGTTATCGGCCTGATAACCCTGGCAGTGATGGTTTTCGCCG

GCGGCCATTTTTACCGCAGTGCATGGAAAAGCCTGCTGAACGGTGCGGCGACGATGGATACGCTGGTGGC

GCTGGGTACTGGCGTGGCGTGGCTCTATTCGATGAGCGTCAACCTGTGGCCGCAGTGGTTCCCGATGGAA

GCGCGACATCTTTATTACGAAGCCAGCGCGATGATTATCGGTCTGATCAATCTCGGCCATATGCTGGAAG

CGCGCGCACGCCAGCGTTCTTCTAAGGCGCTGGAAAAGTTACTCGATTTAACCCCGCCGACGGCACGCCT

GGTTACTGACGAAGGTGAAAAAAGCGTGCCTCTGGCAGAAGTGCAGCCAGGTATGTTGCTGCGCCTGACG

ACCGGCGATCGCGTGCCGGTAGATGGCGAGATTACCCAGGGCGAAGCATGGCTGGATGAAGCGATGCTGA

CGGGCGAACCAATCCCGCAGCAAAAAGGCGAAGGCGATAGCGTCCATGCCGGGACAGTGGTACAGGACGG

CAGTGTGCTGTTTCGTGCCAGTGCGGTTGGCAGCCATACTACGCTGTCACGAATCATTCGCATGGTGCGC

CAGGCCCAGAGCAGCAAGCCAGAAATCGGTCAGCTGGCGGATAAAATCTCAGCCGTATTTGTGCCGGTAG

TGGTGGTTATTGCGCTTGTCAGTGCGGCAATCTGGTATTTCTTTGGTCCGGCACCGCAGATTGTCTATAC

CCTGGTGATTGCCACCACGGTACTGATTATTGCCTGTCCGTGTGCGCTGGGGCTGGCGACGCCGATGTCG

ATTATTTCCGGCGTCGGGCGGGCGGCTGAGTTTGGCGTGCTGGTGCGGGACGCTGACGCGCTGCAACGCG

CCAGTACACTCGACACTGTAGTGTTCGATAAAACCGGGACGCTGACTGAAGGGAAGCCGCAGGTTGTCGC

AGTGAAAACATTTGCTGATGTTGATGAAGCGCAGGCATTGCGTCTGGCGGCGGCACTGGAGCAAGGTTCC

AGCCATCCGCTGGCACGAGCGATCCTCGATAAAGCAGGTGATATGCAGCTACCGCAGGTCAACGGTTTCC

GCACATTGCGCGGGCTGGGCGTGAGCGGTGAAGCTGAAGGTCATGCGTTATTGCTGGGCAATCAGGCGCT

GTTAAATGAGCAACAGGTTGGTACCAAAGCTATCGAAGCGGAGATTACTGCTCAGGCATCGCAAGGGGCA

ACGCCTGTGCTGCTGGCGGTTGACGGGAAAGCGGTAGCCCTGCTGGCAGTACGCGATCCGTTGCGTAGTG

ATAGCGTGGCGGCGCTGCAACGCCTGCATAAAGCGGGATATCGTCTGGTGATGTTGACCGGGGATAACCC

AACCACCGCCAATGCGATCGCCAAAGAAGCAGGGATTGATGAGGTGATCGCCGGGGTGCTGCCGGACGGT

AAAGCCGAAGCGATCAAACATCTGCAAAGTGAAGGACGTCAGGTGGCAATGGTGGGCGACGGCATTAACG

ACGCGCCAGCGCTGGCTCAGGCGGATGTCGGCATTGCGATGGGTGGCGGCAGTGATGTTGCCATTGAAAC

CGCGGCGATTACCCTGATGCGCCATAGCCTGATGGGCGTTGCGGATGCGCTCGCTATTTCCCGCGCAACG

CTGCACAACATGAAGCAGAACCTGCTCGGTGCGTTTATCTACAACAGTATCGGTATTCCGGTCGCCGCCG

GTATTTTGTGGCCGTTCACTGGAACACTGCTTAACCCGGTAGTTGCCGGAGCGGCAATGGCGCTCTCGTC

GATTACCGTAGTGAGTAACGCCAACCGGTTGCTGCGGTTTAAACCGAAGGAATAA

>gi_38347830_Serratia marcescens plasmid R478_24

ATGCTGTTGAAAACGTCTCGACGAACTTTCCTGAAGGGGTTAACCCTCTCTGGCGTAGCCGGAAGTCTTG

GCGTATGGAGTTTCAATGCGCGTTCCAGTCTGAGCCTGCCAGTTGCCGCATCCCTGCAGGGTACTCAGTT

TGACCTGACCATTGGTGAAACGGCCGTCAATATCACGGGCAGTGAGCGTCAGGCCAAAACAATCAATGGA

GGCCTGCCGGGGCCCGTTCTTCGCTGGAAAGAAGGTGACACCATTACCCTGAAGGTCAAAAACCGTCTTA

ATGAACAGACGTCCATTCACTGGCACGGCATTATTCTTCCGGCCAATATGGATGGTGTTCCGGGGCTGAG

TTTTATGGGCATAGAGCCTGATGATACCTACGTTTACACCTTTAAGGTTAAGCAGAACGGGACTTACTGG

TACCACAGCCATTCCGGTCTGCAGGAACAGGAGGGGGTATACGGTGCCATTATCATCGATGCCGGGGAGC

CAGAACCGTTTACTTACGATCGTGAGCATGTGGTCATGTTGTCTGACTGGACCGATGAAAATCCTCACAG

CCTGCTGAAAAAATTAAAAAAACAGTCGGATTACTACAATTTCAATAAACCAACCGTTGGCTCTTTTTTC

CGCGACGTGAATACCAGGGGGCTGTCAGCCACCATTGCCGATCGGAAAATGTGGGCTGAAATGAAAATGA

ATCCGACTGACCTCGCGGATGTCAGTGGCTACACCTACACCTATCTCATGAACGGGCAGGCCCCGCTGAA

AAACTGGACCGGACTGTTCCGTCCCGGTGAAAAGATACGCTTACGGTTTATCAACGGCTCGGCAATGACC

TATTTCGATATCCGTATCCCCGGGCTGAAAATGACGGTCGTGGCTGCAGATGGCCAGTATGTAAACCCGG

TTACCGTTGACGAATTCAGGATTGCCGTTGCCGAAACCTATGATGTCATTGTGGAGCCTCAGGGTGAGGC

CTATACCATCTTCGCACAATCCATGGACAGGACCGGTTACGCTCGAGGGACACTGGCCACGAGAGAGGGG

TTAAGTGCTGCCGTTCCCCCCCTCGATCCCCGTCCTCTGTTGACCATGGAAGATATGGGTATGGGGGGAA

TGGGACATGATATGGCAGGAATGGACCACAGCCAGATGGGAGGCATGGATAACAGCGGAGAGATGATGTC

TATGGACGGTGCTGACCTTCCGGATAGCGGGACATCCTCCGCGCCCATGGATCACAGCAGCATGGCCGGT

ATGGATCATTCCCGGATGGCCGGAATGCCGGGTATGCAAAGTCATCCTGCGTCAGAAACGGATAACCCAC

TGGTTGATATGCAGGCGATGAGCGTCTCTCCGAAATTAAATGATCCGGGTATTGGTCTTCGAAATAACGG

AAGAAAGGTTCTCACGTACGCGGATTTGAAAAGCCGCTTTGAGGATCCTGACGGACGTGAACCTGGCCGT

ACCATAGAACTGCATTTAACCGGCCACATGGAAAAGTTTGCCTGGTCATTTAACGGAATCAAGTTTTCAG

ATGCCGCACCGGTGCTGCTGAAATACGGTGAGCGGCTCAGGATCACGCTGATCAACGATACCATGATGAC

TCACCCCATTCACCTGCATGGTATGTGGAGCGATCTGGAAGATGAAAACGGTAATTTCATGGTTCGTAAA

CACACAATAGATGTTCCCCCTGGTACAAAACGCAGTTACAGAGTGACAGCAGATGCGCTTGGCCGCTGGG

CGTATCACTGCCATTTGCTCTATCACATGGAAATGGGAATGTTTCGTGAAGTCCGGGTGGAGGAATGA

>gi_21240774_Xanthomonas axonopodis pv. citri str. 306 chromosome_25

ATGTCTTTCGATCCCCCGTTTTCCCACGGCTCAACCGCCAGCGGCCTGAGCCGGCGCCGCTTCGTCCAGG

GCCTGGCCCTGGGCGGCATCGCCGCCAGCGGCCTGTGGCGCAACGATGCGCGCGCCGCGGCCCAGGCGAA

CACGCCCGTGCTGCGCGGAAGCAGCCAGTCGCTGCAGATCGGCCGCCTGCCGGTCAACTTCACCGGGCGC

CCCCGCTCCGCAATCACCGTCAACCAGAGCCTGCCGGCACCCACCTTGCGCTGGCGCGAGGGCGACACGG

TGAGCGTGCGCGTGCGCAACGCGCTGACCGACCAACCCACCTCGGTGCATTGGCATGGCCTGTTGCTGCC

GGCCAACATGGACGGGGTGCCGGGCATGAGTTTCGACGGCATCGCGCCGGGCCAGGAGTATCACTACCGC

TTTGCGCTGCGCCAGTCCGGCACCTACTGGTACCACAGCCATTCGATGTTTCAGGAGCAGTCAGGGCTGT

ATGGCGCCATCGTCATCGACCCGCTGGTGCCGCCGCCGTACCGGCACGATCGCGAACATGTGGTGCTGCT

CTCGGACTGGACCGATCTGGACCCGGCCGCCCTGTTCCGTCGTTTGAAACAGATGCCGAGCCACGACAAC

TACGCGCAGCGCACCGTCGGCGATTTTCTACGCGATGCGCGCGACGACGGCCTGCGCGCCACGCTGGCCG

ACCGCGGCATGTGGGGGCGCATGCGCATGACGCCCACCGACCTGTCCGACGTCAACGCCAACACCTACAC

CTACCTGCTCAACGGCGTGGCACCGGCCGGCAATTGGACCGGCTTGTTCAAACCCGGCGAGAAGGTGCTG

CTGCGTTTCATCAATGGCTCGTCGATGACCTACTTCGACATCCGTATCCCCGGCCTGCGCATGACCGTGG

TGGCCGCCGACGGGCAATACGTGCATCCGGTCAGCGTGGACGAACTGCGCATCGCCGCGGCCGAAACCTT

CGACGTGATTGTCGAACCGCTCGGCCAGGATGCGTTTACGTTATTCGCGCAGGACATGGGCCGCACCGGC

TTCGCCTGCGGCACGCTGGCAGTGCAGCACGGCTTGCAGGCGCCGATTCCTGCGCTGGATCCGCGCGCCA

TCCTGACCATGCAGGACATGGGGCATGGCGATGGCATGGATCACGCGCTGCCCGCAATGCACGGCGCGCC

CGGGATGCTGGCGGCGCATGGGATGCACACCATGCACTCGCATGACGATGCCAAGGCACACGACAAGGCG

CCGCATCATTCCGCCAGCGAAACCGGCAACCCGCTGATCGACATGCGCAGCAATGCCACTGCACCGCGCC

TGGACGACCCCGGCGTGGGCCTGCGCGACAACGGCCGCCGCGTGCTGTGCTACGCCGATCTGCATAGCGT

GTTCGACGACCCGGATGGGCGCGAACCGGGCCGTGACATCGAGCTGCATCTGACCGGGCATATGGAAAAA

TTCGCATGGTCCTTCGATGGCATCGCGTTCGCTTCCGCGCAACCCTTGCAGCTGCAATACGGCGAGCGCC

TGCGCATCGTGCTGGTCAACGACACCATGATGCAGCACCCCATCCATCTACATGGCATGTGGAGCGACCT

GGAAGATGCGCACGGCAACTTCCAGGTACGCAAGCACACCATCGACATGCCGCCCGGCACCCGCCGCACC

TACCGCGTGCGTGCCGATGCGCTCGGCCGCTGGGCGTATCACTGCCATCTGCTGTACCACATGGAAGCGG

GCATGATGCGCGAAGTGCGGGTGGAAGCATGA

>gi_16763390_Salmonella enterica str. LT2 chromosome-26

ATGTCTCAAACTATCGACCTGACCCTGGACGGTTTGTCCTGCGGTCATTGCGTTAAACGCGTGAAAGAAA

GTCTTGAACAACGTCCGGATGTTGAGCTGGCGGACGTCACCGTAACCGAAGCGCACGTTACCGGTACCGC

CAGTGCGGACGCGTTAATTGAAACCATCAAACAAGCCGGGTATGGCGCGACGTTAAGCCACCCAAAGGCT

AAACCGCTGACGGAGTCATCAATCCCGTCGGAAGCACTGGCAGCGGTTCCTCATGAGCTTCCGGTAGCGA

CGGCCGATGAAGAAAGCCAACAGTTGCTGTTAAGCGGCATGAGCTGCGCCAGTTGTGTCACCCGCGTACA

ACATGCGCTACAAAGCGTACCGGGCGTCACGCAGGCACGGGTAAACCTGGCGGAACGCACTGCGCTGGTG

ATGGGCAGCGCGTCCGCCGCTGATTTAGTGCAGGCGGTGGAAAAAGCCGGCTACGGCGCAGAAGCGATTG

AAGATGACATCAAACGCCGCGAGCGCCAGCAGGAAACCGCCATCGCCACCATGAAACGTTTTCGTTGGCA

GGCAATCGTCGCCCTTGCGGTCGGTATACCGGTCATGGTCTGGGGGATGATCGGCGATAACATGATGGTG

ACCGACGACAACCGCAGCCTGTGGCTGGCTATTGGCCTGATCACGCTTGCGGTGATGGTCTTTGCGGGCG

GTCACTTTTACCGTAACGCGTGGAAAAGCCTGCTGAACGGCACGGCGACGATGGATACGCTGGTGGCGCT

CGGCACCGGGGTTGCCTGGCTCTACTCCATGAGCGTCAACCTGTGGCCGCAGTGGTTCCCGATGGAAGCG

CGGCACCTCTATTACGAAGCCAGCGCGATGATTATCGGTTTGATTAATCTCGGACACATGCTGGAAGCGC

GCGCCCGTCAGCGTTCCTCAAAAGCGCTGGAAAAATTACTCGATCTCACCCCGCCCACCGCACGCGTCGT

GACAGAAGACGGCGAAAAAAGCGTCCCGCTGGCGGACGTTCAGCCAGGGATGCTGCTGCGGCTCACTACC

GGCGATCGCGTGCCGGTGGATGGCGAAATTACCCAAGGCGAAGCGTGGCTTGATGAAGCCATGCTGACCG

GCGAACCTATTCCGCAGCAAAAAGGCGAAGGCGACAGCGTTCATGCCGGCACCGTCGTCCAGGACGGCAG

CGTCCTGTTCCGCGCCAGCGCGGTGGGCAGCCATACCACGCTGTCACGCATTATTCGCATGGTACGCCAG

GCGCAAAGCAGTAAACCGGAAATTGGCCAACTGGCCGATAAAATCTCCGCGGTATTCGTTCCCGTCGTCG

TGGCCATTGCGCTGTTCAGCGCCGCTATCTGGTATTTCTTCGGGCCGGCGCCGCAAATTGTTTATACGCT

GGTCATCGCCACCACCGTACTGATTATCGCCTGCCCTTGCGCATTAGGCCTGGCGACGCCAATGTCGATT

ATTTCCGGCGTAGGCCGCGCTGCCGAATTCGGCGTGCTGGTACGCGACGCCGATGCCCTGCAACGCGCCA

GTACGTTGGATACCCTCGTTTTTGATAAAACCGGGACGCTGACGGAAGGCAAGCCGCAAGTGGTGGCGAT

AAAAACCTTTAACGGCGTTGAGGAAGCTCAGGCATTACGTCTGGCTGCCGCGCTCGAACAAGGCTCCAGC

CACCCGCTGGCCCATGCGATTCTGGAAAAAGCCGGCGATGATAAACTGCCGCAGGTGAACGGTTTCAGAA

CGCTGCGCGGACTGGGCGTCAGCGGTGAAGCGGAAGGTCATCAACTGCTTCTGGGGAACCAGGCGTTGCT

GAACGAACAGCACGTCGCCACGGATGACATGACGGCGGAAATCACCGCGCAGGCCTCGCAAGGCTCGACT

CCGGTCCTGCTGGCGATTGACGGTAAAGCGGCGGCGTTACTGGCGGTACGCGATCCATTGCGCAGCGATA

GTATTGCCGCGCTTGAACGCCTGCACAACGCCGGATACCGTCTGGTAATGCTCACCGGGGATAACCCAAC

GACGGCTAACGCGATTGCCAAAGAAGCGGGTATTGACGAAGTCATTGCGGGCGTTCTGCCGGATGGCAAA

GCCGATGCGATTAAACGCCTGCAAAGCCAGGGTCGTCAGGTTGCGATGGTCGGCGATGGTATCAACGATG

CGCCCGCGCTGGCGCAGGCGGACGTCGGTATCGCGATGGGCGGCGGCAGCGATGTGGCGATTGAAACCGC

GGCGATTACGCTGATGCGCCATAGTCTGATGGGCGTGGCGGATGCGCTGGCCATTTCGCGCGCGACGTTG

CGCAATATGAAACAAAACCTGCTTGGCGCATTTATCTACAACAGCATTGGTATCCCGGTGGCCGCCGGTA

TTCTGTGGCCGTTTACCGGTACCTTGTTAAACCCGGTAGTAGCAGGCGCGGCAATGGCGCTGTCTTCCAT

TACCGTCGTCAGCAATGCCAACCGGCTATTGCGTTTTAAACCGAAAGCGTAA

>gi_71064581_Psychrobacter arcticus 273-4 chromosome_27

ATGCATAAACAGAACATACTGAACCGCCGACGTTTTTAACCGGATCCTCTGCTGTTCTTGGCGCCTCCT

TGCTGCCGACCATTGCCAGCAGCGCGTTGGGACATTCGAGTCGCTCGGGCAATCAGGGCGCTACTATCAA

TAGTGATCAAAACACTCATAAAGTGCCTGTATTGACCGGTAAAGAGTTTGATTTATACGTCAGGAAACAG

TCCGCTATTGTTAACGGTAAAAAGAGCATGGCGACGCTCATCAATGATTCACTGCCAGCACCGACGCTAA

AGATGCGTGAAGGCGATACGGTGGTGATACGTGTTCATAATCAGATGGATGAATCAACCTCCATTCATTG

GCATGGATTGCTGGTACCGTTTGAGATGGATGGGGTGCCGGGCATTAGCTTTGATGGTATTCCTGCGAAC

AGCACCTTTACTTATAAATTTGAGCTCAAACAATCAGGCACCTATTGGTATCACTCACACAGTGGGTTCC

AAGAACAAACCGGTATGCTGGGTGCTATTGTGATTGAACCTAAAGGGCGTGAGCGTCATCCTATCGACGA

AGACCACGTGATCGTGCTTAGTGATTGGACCAGCCGCAACCCGCATAATCTTTTGAAGCTGCTCAAACAG

CGCTCCGACTTTGATAATTATCATCTGCCAGACTTCAAGAAGCTACTGTCTGATATTGCCGAAACAGATA

TGAAAACCGCGTTTGATAAGCGCAAAATGTGGAATCAGATGCGCATGATGCCCACTGATTTTACTGACTT

GTCGGGCGAAAAAACCTTTACCTACCTTATCAATGGCAAAACAACCGCGGCTAATTGGGCACAAATAGTC

AAAGCAGGACAGCGCGTTAAGCTACGCTTTATCAATGCCTCAGCGCAAACCATCTTTGATGTGCGCATAC

CTGGTCTCAAAATGACTGTGGTGGCAACCGATGGCATTGATGTCGCACCAGTTTCGATTGATGATTTTCG

CATTGGCGTGGCTGAGATTTATGATGTCATTGTCACCCCGACTAAAGATGCGCATACCATATTCGCCCAA

AACATAGACCGTTCAGGTTATGTCGCAGCAACGCTTGCCACCAAAGAAGGCGCTCGTGCGGCAACGCCTG

CCATGGACAAGATTGAATGGCTCACAATGGCAGATATGATGGGCGCTATGGGTGCTGATGGTTATAAAGC

CAAGCATGCCAAAACAGAGTATGATTTTAAGAGCGATATGCGTGTGGACAGTCCACGTATGAACCTTGAT

GATCCCGGTATCAACCTGCGCAAGATTAATAGAGAGGTGCTGAATTATAGCCAGCTGCGCTCTGTCGATG

AGGCAATATTTGCGGAGCAGCGCCCGCCTACCCGAGAGATCGAGCTACATTTAACCGGTAACATGGAACG

CTATATTTGGGCATTTGATGGGGTTAAGTTCAGTGAGGCGACACCGGTCAATATTAAGCCGGGTGAGCGC

GTGCGTATTACCTTAGTCAATGACACGATGATGAATCATCCGATGCATTTACATGGCATGTGGAGCGATT

TGCGCATGCCTAACGGCGAGTTTCAAGTACGCAAGCATACCATTATGGTGCAACCAGCACAAAAAATCAG

CTTTGATGTCACAGGGGAAGCTGGTCGATGGGCATGGCATTGTCATCTGCTCTATCACATGGAAGCCGGT

ATGTTTAGAGAAGTGGCGGTCATTTAA

>gi_77361923_Pseudoalteromonas haloplanktis TAC125 chromosome_28

ATGGGTTTTAAAAAGTCATTGCAGCACATTTCAAAGCCACGTAGGCGTTTTGTTCAAGGATTAATTGCAG

GCGGTGTGCTGGCAGCTTTTCCAACTGTTTTACATGCAGCATCATCTTTAGCGGCTTCAACATTAACAGG

TACTGTGCCTGAGTTGAGCGGTAAAGTTATTGACCTGGTTATTGATGAGTCCCCAGTAAACTTTACCGGT

GTAGTGCGTATGGCAACCACCATTAATGGCTCTATTCCCGCGCCAACATTACGCCTTAAAGAAGGTGACG

ATGTGACCATTCGCGTTACCAACAATTTAGCGGTACCTAGCTCTATTCACTGGCACGGAATTATTTTACC

GTATCAAATGGATGGTGTACCGGGCATTAGCTTTAAAGGCATTATGCCTGGCGAAACGTTTGTTTATAAA

TTTAAGTTACAACAAAGCGGTACTTATTGGTATCACTCACACAGTGGCTTTCAAGAAATGACCGGTATGT

ATGGGGCGTTAATTATTGAGCCTCGTGAGCAAGATGTTATCAGCGCAGATAATGAACATATTATTCAACT

CTCTGATTGGACTGATGATGATCCTATGGAGTTATTCCGTAAATTAAAAATTCAGGGGGATGTGTTTAAC

TTTAATCAGCCCACTGTACCTGAGTTTTTTGATGACATAGCAACAAGCGGCGTTGCTAATGCACTTCAAC

GACGTGAAATGTGGAACCAGATGCGAATGAGTCCAACCGATTTAGCTGATTTATCGGCTTCGGCAATGAC

CTATTTAATGAATGGTACTGCGCCAATGGCTAATTGGCGTGGGCTGTTTAAAGCGGGCGAAAAAGTACGT

TTAAGATTTATTAATGGCTCAAGTAACACCTTTTTTGATGTACGTATCCCAGAGTTAAAATTAACTGTGG

TACAAGCAGATGGACAAAATGTTGAACCCGTTACCGTAGATGAGTTTCGCTTTGGTCCAGGTGAAACCTA

CGATGTGGTTGTTGAGCCTAAAAATGACGCATACACAATTTTTGCACAAAGCATGGACCGCTCAGGTTAT

GCCAAAGGGACTTTGTCTGTCGCTGCTAATATTGATGCGCCAGTGCCTGCACTTGACCCAGTTGAGTGGT

TAGCAATGCGGGATATGATGGGCAATATGGATCACTCTGCTATGCCTGGAATGGATCACAGCGCTATGGG

CCACGCTAGCATGGACAAAACCAGTATGGATCAGGGGGCAATGGATCATAGCACAATGGACCACGGTGCA

ATGGCAATGGACCATAGTAAGCATAATATGGGTAAAAACCCACTGGCTGTTCCAAGTCAGAAAGTTCGCC

ACGCTAAAACAGAATATGGCGCCTCAGTTGATATGCGCGTTGATACGCCACGCACAAACTTAGACGATCC

TGGTATTGGGCTGCGCAATAACGGCCGTCGTGTTTTAACACTTGCAGATTTACGCTCTCTTGATGGCATA

GTTGACCACCAAGCGCCTGAAGCCGAAATTGAGCTGCACTTAACCGGCAACATGGAACGCTACAGCTGGT

CTTTTGATGGCTTAGAGTTTGGTAAAAGTACCCCTGTGCATATGAAGCATAATCAGCGGGTACGAGTTAT

TTTACAAAACGATACCATGATGACACACCCTATGCATTTACATGGTATGTGGAGCGACTTGGAGAACGAT

CAAGGTGATGTGTTAGTTCGCCGCCACACCATAATGGTACAGCCCGCCCAAAGAATTAGCTTTTTAACTA

CGCCACATGATGTTGGTCGTTGGGCTTGGCATTGTCATTTACTGTTTCATATGGATGCAGGTATGTTTAG

AGAGGTGGTTGTATCATGA

>gi_28867243_Pseudomonas syringae pv. tomato str. DC3000 chromosome_29

ATGCCCACCCGAACATCAAGACGCACCTTCGTGAAAGGGCTTGCCGCCAGCAGTATTCTGAGTGGGCTGG

GGCTCTGGCGCAGCCCGGCGTGGGCACTCCCCAATCCGGGCCAGCCTGACGGGTTGTCCGGCACCGAGTT

CGACCTGACCATCGGCGAAACCCAGGTCAATATCACCGGCAATGCCAGAACCGCGATGACTATCAACGGC

GGCATTCCAGGCCCATTGTTGCGCTGGCGCGAGGGCGACACTGTCACCCTGCGGGTAAAAAACCGCCTCG

ACGAAACCACCTCCATTCACTGGCACGGCATCATCCTGCCGGCCAACATGGACGGCGTGCCGGGGCTGAG

CTTTGATGGCATTGCGCCCGACGGCATGTATGTCTATCGCTTCAAAGTCAGGCAGCACGGCACGTATTGG

TATCACAGCCATTCAGGTTTTCAGGAGCAGTCGGGCGTCTATGGGCCACTGGTGATCGACGCCAAAGAAC

CCGAGCCGTTCACCTACGAGCGCGAGCACGTGGTCATGCTCACCGACTGGGCAGACGAAGACCCGGCCCG

GGTGATGAAAAAGCTCAAGAAACAGTCGGACTACTACAACAATAACAAGCGCACCGTCGGCGACTTCATC

AACGACGTCGGCGAAAAAGGCTGGAGCGCGACCACCGCCGAGCGCTGGATGTGGGCGCAGATGAAGATGA

ACCCGACTGATCTGGCGGACGTCAGCGGCGCAACCTACACCTACCTGATGAACGGCCAGGCGCCAAACAT

GAACTGGACCGGGCTGTTCAAACCCGGCGAGCAGATTCGCCTGCGCTTTATCAACGGCTCGTCGATGACC

TATTTCGATGTGCGCATTCCCGGCCTGAAGATGACCGTGGTCGCCAGCGACGGCCTGCACATCAAACCGG

TGGTTGTCGATGAACTGCGGATCGCGGTGGCAGAGACATTCGACGTGATCGTCGAGCCGGCTGACGGTGC

CTACACGCTGTTCGCCCAATCGATGGACCGCACCGGCTTTGCCCGAGGCACTTTAACCAGCAGGCCCGGC

ATGCAGGCCGAGGTCCCGCCTCTCGACCCGCGTCCGCTGCTGAGCATGGACGACATGGGCATGGCGGGCA

TGGATCACGGCAGCATGAACCACAGCGCCAAGCCGGCAATGGACGGTATGGACCACAGCAAGATGGACCA

TGACTCGATGCCCGGCATGGACCACGGCACGATGCCGATGCAAGAAGCACCGGTCATGCAGTCGCACCCG

GACAGCGAAAGGAATAATCCGCTGGTCGATATGCAGGCCATGAGCACGTCGGCAAAACTCAATGATCCGG

GCATCGGCCTTCGGGACAACGGCCGTAAGGTCCTGACCTACGCCGACCTGCGCAGCACCTTCGAGGACCC

GGACGGCCGTGAGCCGAGCCGGACAATCGAACTGCACCTGACCGGGCACATGGAAAAATTCGCCTGGTCG

TTCGATGGCGTGAAGTTCTCCGACGCCAAGCCACTGATGCTCAAGTACGGCGAGCGGGTGCGTATCGTGC

TGGTCAACGACACGATGATGACTCACCCCATTCACCTGCACGGCATGTGGAGCGATCTGGAAGACGAAAA

CGGTCAGTTCATGGTGCGCAAACACACCATCGACATGCCCCCGGGGTCACGCCGCAGCTACCGCGTCACC

GCTGATGCTCTGGGCCGTTGGGCGTATCACTGTCATATGCTGTACCACATGGAAATGGGTATGTTCCGCG

AAGTGCGGGTGGAAGAATGA

>gi_26986745_Pseudomonas putida KT2440 chromosome_30

ATGCAAAGCAAAACCACGAGACGATCTTTCGTCAAAGGCCTGGCCGCTACTGGATTACTGGGTGGACTGG

GCATGTGGCGCGCGCCGGTCTGGGCTGTGACCAGCCCTGGCCAACCGAATGTTCTGACAGGCACCGATTT

TGATCTCTACATCGGTGAGTTGCCGGTCAACATTACCGGCACTGTACGTACAGCGATGGCAATCAACGGC

TCCATTCCCGGGCCCATCCTGCGCTGGCGCGAGGGCGATACCGTCACGCTGCGTGTGCGCAACCGCTTGC

AGCAGGACACCTCCATCCACTGGCACGGCATCATCCTGCCGGCGAACATGGACGGCGTGCCGGGCCTGAG

CTTCCATGGCATCGCCCCAGACGGCATGTATGAGTACAAGTTCAAGGTCCAGCAGAACGGCACCTACTGG

TACCACAGCCACTCCGGTTTCCAGGAGCAGGTCGGGGTGTATGGTGCCCTGGTCATCGATGCGAAAGAAC

CTGAGCCGTTCACCTATGACCGGGACTACGTGGTCATGCTCAGCGACTGGACGGATGAAGACCCTGCACG

GGTGCTGTCCAAGCTCAAGAAGCAGTCCGACTACTACAACTACCACAAGCGCACGGTGGGCGATTTCGTC

AACGATGTGAGCGAAATGGGCTGGTCCGCCGCGGTAGCCGACCGCAAGATGTGGGCCGAGATGAAAATGA

GCCCCACCGATCTCGCTGATGTGAGCGGCTACACCTACACCTACCTGATGAACGGCCAGGCCCCGGACGG

TAACTGGACGGGCGTGTTCAAGCCGGGCGAGAAAATCCGCCTGCGCTTCATCAATGGCTCGGCCATGACG

TATTTCGATGTGCGAATTCCCGGCCTGAAGATGACGGTTGTCGCGGCGGATGGGCAGCATGTCAAACGCG

TTGCGGTCGACGAGTTCCGCATCGCCGTTGCCGAAACCTACGACGTCATTGTTGAGCCGGAAGACGAGCA

GGCTTACACCATTTTTGCTCAATCCATGGATCGCACAGGCTACTCCAGAGGCACTTTAGCCGTTCGCGAA

GGTATGCAAGCTGCAGTTCCGGCAGTTGATCCGCGCCCTCTCATCTCAATGAGTGATATGGGTATGGATC

ACGGCAGCATGGCCGGCATGGACCACGGCAACATGGCCGGTATGGACCACAGCAAAATGGCTGGGATGGA

CCACGGCAACATGGCCGGCATGGACCACAGCAAAATGGCTGGGATGGACCACGGCAACATGACCGGCATG

GACCACAGCAAAATGGCTGGGATGGACCATGGCAGCATGGCCGGCATGGATCACAGCAAAATGGCTGAGA

TGGACCACGGCGGCATGGCCGGCATGGACCACAGCAAGATGGCTGGGATGGACCAAGGCGGCATGGCCGA

TATGGACCATAGCAAGATGGCTGGGATGGACCAAGGCGGCATGGCCGATATGGACCACAGCAGCATGGAA

GGTATGGGTGGCGCCATGCAGTCTCACCCAGCCTCAGAGACTAACAACCCCTTGGTCGACATGCAGACTA

TGAGTCCAACGCCTAAGTTGGACGATCCGGGAATCGGCTTGCGCAACAATGGACGTCGTGTCCTGACCTA

TGCGGATCTGCGAAGCACGTTCATTGATCCAGATGGACGTGAACCAGGGCGCACCATCGAGCTGCATCTC

ACAGGCCATATGGAGAAGTTCGCCTGGTCATTTGACGGCGTCAAATTTTCCGATGCTGAACCCCTACGCC

TCAAATATGGCGAGCGTCTGCGAATCACTTTGGTCAACGACACCATGATGACTCACCCCATCCACCTCCA

CGGAATGTGGAGCGATCTGGAGGATGAGGACGGTAATTTCATGGTTCGAAAACACACCATCGACATGCCT

CCAGGCTCAAAACGAAGCTACCGAGTAACCGCGGATGCTTTGGGCCGTTGGGCCTACCACTGCCACCTAC

TGTTCCATATGGAAATGGGCATGTTCCGAGAAGTCCGCGTGGACGAATGA

>gi_28197945_Xylella fastidiosa Temecula1 chromosome_31

ATGGGAGTCTCTATGAACACTGTTACCTTCCCTTCCCAGGGAGGCATTGACCGGCGTCGCTTCCTAAAAG

GCTTGGCGTTGGGAGGCGTCGTCGCCGGTACCGGCTTATGGCGTCTTCCTGTCCATGCAGCCAACCCTGA

GTTGCCGCCACTGCAGCGGCGTACCACTCCCCTAGAGCTGCGTATCGGACAGTCCAGCGTTAATTTCACC

GGACGTATGCGTCCAGCCATCACCGTTAATAACAGCTTGCCTGGACCCGTATTGCGTTGGCGCCAAGGCG

ATACGGTCCAAATACATGTCACCAATACCTTGCCCGATGTGATGACCTCAATTCATTGGCACGGCATCGT

TCTGCCCTCCAACATGGATGGCGTACCGGGCATGAGTTTCGACGGTATTGCACCAGGCGAACACTATCTA

TACCGCTTTCAGTTACACCAGTCTGGGACCTACTGGTACCACAGCCACGCCATGTTTCAAGAACAGGCCG

GCCTGTATGGAGCGTTGATTATTGATCCACTGGAGCCGCCTCCCTACCGCGCCGACCGCGAGCACATCCT

CCTATTCTCAGATTGGACCGACCTGGACCCTGCCGCACTGTTTCGCAGGCTCAAAAAAATGTCCTCTTAC

GACAACACCTACCAACGCACCGTCCGCGATTTCTTCCATGACATACACCGTGATGGCCTGCGCACCACAC

TGGCCGATCGCCGCATGTGGGGCAGAATGCGGATGACCCCGACAGACCTGTCCGATGTCAATGCCCATAC

CTATACCTACCTGCTGAACGGCACCACGCCAGCCGGCCACTGGACTGGATTGTTCCGCCCCGGAGAAAAA

GTGCTATTACGGCTGATTAATGGTTCGGCGATGACCTATTTTGACGTCCGTATTCCAGGCCTGAAACTGA

CCGTTGTCGCCGTGGATGGCCAATACGTCCATCCGGTGACCGTGGATGAATTACGTATTGCCCTCGCCGA

AACCTATGATGTCCTGATCCAACCCCACGGCCAGGATGCCTTTGCAATCTTTGCCCAGGACATGGGCCGC

ACCGGCTACGCCTGCGGCACCCTCGCCGTGCGTCCCGGCTTACACGCCCCCCTGCCGGCATTGGACCCGC

GTCCTCGCCTGACAATGCAGGACATGGGACACGGGATGACGCATGATCAGCATGGGAGTCGCGTCTCTGA

AATGGCGGACATCCCAGGAATGACCCATCCTGGCAGACTGGGCGATCATGCCAGCCACGCCAACCAGCCT

CCTGCGCAGCCCCCTCATGGTTTACATAGCCTTCCGGCGGACAGAACAGCGCCGCAGCATCCTGCCAGCG

AACAGAACAATCCCTTGGTGGACATGCATGTGGACGCACCACAGCCGCGTTTGAATGATCCCGGCATTGG

CTTGCGTGACAATGGCCGCCACGTCTTGACGTACGCTGATCTGCATAGCCTGTTCGATGATCCGGATGGA

CGTGAACCAGGGCGTGAGATCGAATTACATCTCACCGGCAATATGGAGAAATTCGCCTGGTCGTTTGATG

GCATCCCATTTGCCTCCGCGCAACCCTTACGACTCAACTACGGTGAACGTGTACGCATCGTCTTGGTCAA

TGACACCATGATGGCGCACCCCATTCACCTGCACGGCTTATGGAGTGACTTAGAGGATGCCAACGCCGCG

TTTCAGGTCCGTAAACACACCATCGATATGCCGCCGGGTACACGCCGCAGCTACCGTGTGCGTGCCGATG

CACTGGGGCGCTGGGCCTACCACTGCCACATGCTGTACCACATGGAAAGCGGCATGATGCGTGAAGTACA

GGTGCGCCCATGA

>gi_190572091_Stenotrophomonas maltophilia K279a chromosome_32

ATGTCGCATGATGATTTTCGTGGTCCACGCGGTGGACCGCTGCTGCCTTCGCGGCGGCGATTTGTCCAAG

GCTTGGCCTTGGGAGGCGCAGTCGCAGGATTAGGTTTCTGGCCCAAAGCCAGTTGGGCGCTCAAGGGGCC

GGGACAACCCAACGTACTATCGGGCACTGAGTTTGACCTGACCATTGGCGAGACGCCGATGAACTTCACC

GGCAAGACCCGCACCGCGATCACGGTCAACGGGTCCGTTCCGGCGCCGTTGCTGCGGTGGCGGGAAGGCA

CCACGGTCAACTTGCGCGTCTCCAATGCATTGCCCGCTAACTCCATCCATGGCGCGGACACCTCCATCCA

TTGGCACGGCATCATTTTGCCGGCCAACATGGACGGCGTGCCGGGTCTGAGCTTTGACGGTATCGGACGT

GGTGAGACCTACCACTACCGGTTCACCCTGCATCAGGGCGGAACCTACTGGTACCACAGCCACTCAGGGT

TCCAGGAACAAGCCGGGCTCTATGGCCCGATCGTCATCGACCCATTGGAGCCGGAGCCCTTCAGTTTCGA

TCGCGACTACGTCGTGATGCTGAGCGATTGGACAGACCTGGACCCGACGGCCCTGTTCGATCGTTTGAAG

AAGATGCCGGGCCATGACAATTACTACAAGCGCACGGTCGGCGATTTTGCGCGCGATGTGAAGCGCAACG

GCCTGTCGGCCACGTTGGAAGATCGCAAGATGTGGGGCGTGATGCGGATGACGCCCACGGACCTGTCCGA

CGTCAACGCCAACACCTACACCTACCTGATGAACGGCACGACCTCACTGGGCAACTGGACCGGTTTGTTC

CGCAGTGGCGAGAAGGTGCGCCTGCGTTTCATCAATGGCTCTGCCATGACGTACTTCGATGTGCGTATTC

CGGGGCTGAAGATGACCGTGGTGGCGGCAGATGGCTTGTATGTCCATCCGGTTTCCGTCGACGAGTTCCG

CATCGCGGTAGCAGAAACCTTCGATGTGATCGTGGAGCCCTCCGGGCAGGACGCATTCACCATCTTTGCC

CAAGACTCCGGTCGCACCGGCTACATCAGCGGCACGCTCGCTGTGCGCGAAGGATTACGCGCGCCCGTTC

CGTCTGTGGATCCCCGGCCGCTGCTGACGATGGCAGACATGGGCATGGATCATGGGTCGATGGATATGTC

TGGCGGCAGCAAGGGCATGGAAGGCGGCTGTGGTGCGGCCATGGGCATGCCTGGCATGACCCCACCTGTC

AGCGGTAACGCGACCTCGGCCCATGCAGGCCATGCGATGCCCGCCGCCGGCGATGGTGCCATGGCAGGCA

TGCAGCACGGGGGCATGCAATCACACCCTGCTAGCGAGACCAACAATCCCCTGTTGGACAACCAAGCCAT

GAGCGTGAGTTCGCGCTTGGATGATCCGGGCAATGGCCTGCGCGATAACGGCCGTCATGTGCTGACGTAT

TCCATGCTCAAGAGCACCTTTGAAGACCCTGACGGACGCGACCCCGGTCGCGAGATCGAGCTGCATCTGA

CCGGACACATGGAGAAATTCTCCTGGGGCTTCAACGGTCAGAAGTTTTCCGATGTCGAGCCGCTGCGGCT

GAACTACGGCGAGCGTATGCGCATCGTATTGGTTAACGACACGATGATGACCCATCCCATCCATTTGCAC

GGCATGTGGAGTGACGTGGAGGACGACAACGGCAACTTCATGGTGCGCAAGCACACGGTGGATATGCCGC

CAGGTAGCCGACGCACGTATCGCGTGCGTGCCGATGCGTTGGGCAGCTGGGCGTTCCATTGCCACCTGCT

TTATCACATGGAAGCCGGAATGATGCGCACGGTGAGGGTCGACGAATGA

>gi_188574270_Xanthomonas oryzae pv. oryzae PXO99A chromosome_33

ATGTCTTTCGATTCCCCGTGTTCCGATGCTTCATCCAGCAACGGCCTGAGCCGGCGCCGCTTCGTGCAAG

GCTTGGCCCTGGGCGGCATTGCCGTTGCCGGCGGCCTGTGGCGGCGCGATGCGCGCGCAGCCACTCCGGC

CAACACCCCGGTACTGCGCGGCAGCAGCCAGTCGCTGCAGATCGGCCGCATGCCGGTCGATTTCACCGGC

CGCACCCGTCCCGCGATCACCGTCAACCAGCGCCTGCCGGCACCGATCCTGCGCTGGCGCGAGGGCGACA

CAGTGAGCGTGCGCGTGCGCAACGCGCTGACCGACCAGCCCACCTCGGTGCATTGGCATGGCCTGCTGCT

GCCGGCCAACATGGATGGCGTGCCCGGCATGAGTTTCGACGGCATCGCGCCCGGCCAGGAGTATCACTAC

CGCTTTGCGCTGCGCCAGTCCGGCACCTACTGGTACCACAGCCATTCGATGTTCCAGGAGCAGGCCGGGC

TGTATGGCGCCATCGTCATCGACCCGCTGACGCCGCCGCCGTATCGGCATGATCGCGAGCATGTGGTGCT

GCTCTCGGACTGGACAGATCTCGACCCGGCCGCACTGTTCCGCCGTTTGAAACAGATGCCCAGCCACGAC

AACGATGCGCAACGCACCGTGGGCGATTTTCTACGCGATGCGCGCGACGACGGCCTGCGCGCATCGCTGG

CAGACCGCGGCATGTGGGGGCGCATGCGCATGACGCCCACCGACCTCTCCGACGTCAACGCCAACACCTA

CACCTACCTGCTCAATGGCGTGGCGCCCGCCGGCAACTGGACCGGATTGTTCAAGCCAGGCGAAAAGGTG

TTGCTGCGTTTCATCAACGGCTCGTCGATGACTTACTTCGACATCCGCATCCCCGGTCTGCGCATGACCG

TGGTGGCCGCCGATGGGCAGTACGTGCATCCGGTCAGCGTGGACCAACTGCGCATCGCCGCGGCCGAAAC

CTTCGACGTGATTGTCGAACCGCTCGGCCAGGATGCGTTTACGTTATTCGCTCAGGACATGGGCCGCACC

GGCTTTGCCTGCGGCACACTGGCGGTGCAGCACGGATTGCAGGCGCCGATTCCGGCACTGGATCCGCGCG

CCATTCTGACCATGCAAGACATCGGCCATGGCGATGGAATGGGGCATGGCGACCACGCGATGCACGGCGA

CGGTACGACGCAAGGCGACGCGCATGCAGCACACGCGATGGCAATGCCAATGCCGGAAATGCACGGGCAT

GGGGGTGGCAACACGCAGACCAAGACACCGCATCAACCCGCCAGCGAAGACGGCAACCCGTTGATCGACA

TGCGCAGCAATGCCACCGCGCCGCGCCTGGACGACCCTGGCGTGGGCTTGCGCGACAACGGACGTCGCGT

GCTGTGCTACGCCGATCTGCACAGCGTGTTCGACGACCCGGATGGCCGCGAGCCCGGTCGCGAGATCGAG

CTGCATCTGACCGGGCATATGGAAAAATTCGCCTGGTCATTCGATGGCATCGCATTCGCTTCCGCAGAGC

CCTTGCGCCTGCAGTACGGCGAGCGGCTGCGCATCGTATTGGTCAACGACACCATGATGCAGCACCCCAT

CCATCTGCATGGCATGTGGAGCGATCTGGAAGATGCCGACGGCAACTTCCAGGTGCGCAAGCACACCATC

GACATGCCACCCGGCACCCGCCGCACCTACCGCGTGCTCGCCGACGCGCTCGGTCGCTGGGCGTATCACT

GCCATCTGCTGTACCACATGGAAGCGGGCATGATGCGCGAAGTGCGGGTGGAAGCATGA

>gi_169794206_Acinetobacter baumannii AYE chromosome_34

ATGTCTAGAAAATTAAGTCATGTCCTTTTAATAGGAGTCGGATTGTTTTCGTCGACTTGGGTTATGGCAG

CGGTTAAAGAATATGATTTAACAATTGCTGAACAGACGGTGAATATTACAGGAAAGCCTCTAAAACGAAT

TACTGTAAATGGTAAGTTTGTTGCGCCTCTTCTTGAGTTTGAGGAAGGCGATGATGCCGTTATTCGTGTA

CACAACAAGTTGAAAAACCAAGATTCTTCTATTCATTGGCATGGTTTAATTTTACCTGGCATCATGGATG

GAGTACCGGGGTTCAACCAGTTTGATGGTATTGCACCAAATAAAACCTATGAATATAAATTCAAGGTTCG

TCAGAATGGAACCTATTGGTATCACTCGCATAGTAAAGGTCAAGAGCAAGATGGTCTATATGGCCCTTTA

GTCATTTATCCCAAAAATAAAGTTCCTTTAACGGCCGGAGAAAAAGCTGATAGAGATTACGTAGTCTTAC

TCTCTGATTTTCACAATTCTACTAGTGGTCAGATTATGAGTAATCTCAAGAAAGAAGCTGATTACTACCA

GAATCGAAGAGAAACAGTTTTTGATGTTTTTAGGCAGATTAAAAGAGATGGTTTAAAAGCAACATGGAAA

GATCGTTCCATGTGGAATCAGATGCGAATGCTTAAGACAGATATGTCTGATGTAACAAACTATACGTTTT

TAATGAATGGTAAGACTCCAGAACAAAATTGGACTGGAAACTTTAAAGCTGGAGAAAAAGTACGTCTCCG

GTTTATCAATGCCTCGGCAATGTCTTTGTTCGATGTAAGAATTCCTAATCTGAAAATGACAGTAGTCAGC

GCGGATGGACAGCCAGTCAAACCCGTGCCAGTGGATGAGTTCCGGATAGGAACAGCGGAGACTTACGATG

TTATCGTAGAACCGAAACAGGCAAATTATCAGATTGAAGCTGAATCTATTGACCGAAGTGGTTTTTCTAT

CGGTACATTACATGACGAAAATACTTCACCTGTAAAAAGTATTGTCATGCCGACACCTCGTCCCCGTGCT

TTACTGACAATGGAAGATATGGGAATGAATCATGACATGTCTTCAATGAAAGGTATGGACCATGACATGT

CTTCAATGAAAGGTATGGATCATGACATGTCTTCAATGAAGGGTATGGATCATGACATGTCTTCAATGAA

AGGTATGGATCATGACATGTCTTCAATGAAGGGTATGGATCATGACATGTCTTCGATGAAAGGTATGGAT

CATGACATGTCTTCGATGAAAGGTATGGATCATGACATGTCTTCAATGAAAGGTATGGATCATGACATGT

CTTCAATGAAAGGTATGGACCATGACATGTCTTCGATGAAAGGTATGGATCATGACATGTCTTCGATGAA

AGGTATGGATCATGACATGTCTTCAATGAAAGGTATGGATCATGACATGTCTTCAATGAAAGGTATGGAC

CATGACATGTCTTCGATGAAAGGTATGGACCATGACATGTCTTCAATGAAAGGTATGGATCATGACATGT

CTTCAAAAACAATGAGCTCTTCTGGATCAGATGAAGTGGTTTACGGATGGGCGAATGCCTCAACCCCAGC

TGGACTAAAAGCACTTCAATATAGTGATCTACAATCTTTAACCCCTCAAAAAGATACCCGTGCTCCAGAA

CGTGAAATTGAAATCCGCTTAGGTGGAAATATGGAGCGCTATATTTGGACAATTAATGGTAAGAAGTTTA

ATGAAACTGAACCATTTAAAGTGAAATATGGTGAGCGTATACGCTTGAAATTTGTTAACGATAGTATGAT

GGCTCATCCGATGCATTTACACGGCATGTTTATGCAACTGGAAAATGGCCAAGATCCAAGTAATATGCCG

AATAAACATACGGTTATTGTTCCACCAGGTAAGACAGTTACAACTTTATTAACGGCTGATGAGTTAGGTG

AATGGGCTATTCATTGCCATTTGCTCTATCACATGTCTGCTGGAATGATGAATAAACTTATTGTGGCAAA

TGTCGATAGTAATAGTACTGATTCGAAAGATGTTGTTGCTCAACCTAATACTAATGATAAGGGGGTAAAT

CAACATGCGCACCACTAA

>gi_16120353_Yersinia pestis CO92 chromosome_35

ATGTTGCAAACCACACTGCTCGCCTTACAAGGACTTTCTTGTATGAATTGTGCTCAACGCGTTAAAGCCG

CGTTGGAAAGCCGTGAGGATGTTCACCATGCTGAAGTGAATGTTCACTACGCTAAAGTGACTGGTGAAGC

CGACACTCATGCACTCATTGAAACGATCAAACAGACCGGTTATCAGGCTACAGAAGCCCAAACGCCGGAC

GTTGAATTACACCTATCAGGATTGAGCTGCGGGCATTGCACTGAGACCGTCCGTAAAGCGCTGGAAGCGG

TTTCGGGGGTTATTTCTGCCGATGTAACCCTCGAGAGTGCCAACGTGTACGGCAAGGCCGATATACAGAC

ATTGATTGCCGCAGTGGAACAGGCTGGCTATCACGCCACACAGCAAGGTATCGACTCCCCAAAAACTGAG

CCGCTGACACACTCAGCCCAATCTCAGCCGGAATCTCTGGCAGCGGCCCCGAATACAGTTCCGGCAACAA

ACGTGGCTTTAGCCACAAGTACGGTTTCTGATACAAATACCGTTTTACCTACAAATACCGCTTTACCTAC

AAATACAACGTCGACAACTTCAACAGCCGATACGGCGTCAGCCACCAGCACGGCACCGGTGATCAATCCG

CTGCCAGTGACCGAATCAGTGGCCCAGCCGGCAGCCTCAGAAGGGGAGAGTGTTCAGCTATTACTGACGG

GGATGAGCTGTGCCAGTTGTGTCTCTAAAGTACAAAATGCATTGCAACGTGTTGATGGGGTTCAGGTGGC

CCGCGTTAATCTGGCTGAGCGCAGTGCATTGGTCACAGGGACCCAAAATAACGAAGCCTTAATCGCTGCG

GTTAAAAATGCCGGTTACGGCGCGGAAATTATTGAAGACGAAGGGGAGCGACGCGAGCGGCAACAGCAAA

TGTCGCAAGCCAGTATGAAGCGCTTCCAATGGCAAGCGGCATTAGGTCTGTTGTTAGGTATCCCATTGAT

GGCCTGGGGGCTGTTTGGCGGTAGCATGACGCTGACGCCAGAAACCCAAACGCCCTGGCTTATCATCGGG

ATTATCACGTTGCTGGTAATGATATTTGCGGGTGGTCACTTCTACCGTAATGCGTGGGTCAGCCTGAAAA

ATGGCAGAGCGACCATGGATACCTTGGTCGCTCTGGGGACAGGTGCGGCCTGGATCTATTCCATCACCGT

CAATATCTGGCCAGATGTTTTCCCTATGGAGGCTCGTCACCTTTATTACGAAGCCAGTGCCATGATCATT

GGTTTGATTAACCTTGGTCATGCGATGGAACAACGTGCCAGACAGCGCTCATCTAATGCGCTCGAACGCC

TGTTAGATTTGGCCCCACCAACGGCAAAGTTAGTGACCGATGACGGCGAGAAGGTGATCCCGCTGGCTGA

TGTCCAACTGGGGATGATCCTACGCCTGACTACTGGCGATCGCGTACCGGTTGACGGCGAGATTGTGCAG

GGCGAAGTGTGGATGGATGAAGCGATGCTGACTGGTGAGCCGATCCCACAACAGAAATCAGTCGGTGATA

TTGTCCACGCGGGGACACAAGTACAAGATGGAACCGTACAATTTCGGGCTAGCGCTATCGGTAGCCAAAC

GACCTTGGCGCGGATTATCAAACTAGTCCGTCAGGCACAAAGCAGTAAACCGGAAATCGGCAAATTGGCA

GACCGTATCTCTGCGGTATTCGTCCCAACGGTGGTGGTCATTGCCATTGTCGCTGGCCTGATTTGGTATT

TCTTCGGCCCACAACCCCAGTTGGTCTATACCCTGGTGGTGGCCACCACGGTGTTAATTATCGCCTGCCC

TTGTGCTTTGGGCTTAGCGACACCGATGTCGATTATCTCTGGCGTCGGCCGGGCCGCTGAATTTGGTGTA

TTGGTTCGAGATGCAGATGCATTGCAGCAAGCCAGTAACCTCGATACGTTAGTGTTCGATAAAACCGGCA

CACTGACTGAGGGGCACCCGCAGGTAGTGGCAATCCATACCTTTAATGGTGTCAGTGAGCAACAGGCTTT

GGGCTGGGCGGCAGCATTGGAAACGGGATCAAATCACCCGCTGGCGCGCGCTATCTTACAACGTGCTGAA

GGGCTGACATTGGCCACCGCCAGCCAATTTCGTACATTGCGCGGTTTGGGGGTCAGTGGTGAAGTTGACG

GCATTCCACTGCTCCTTGGCAATAATCGGCTACTGGAAGAGCAACAGATTGATACCCGCGAGCTGCAATC

ATTGATACAGCAACAAGCGGAAAGTGGCGCAACACCGGTTATTCTGACCGCCAATGGTAAGCCCGCCGCA

CTGTTATCGATCCGTGATCCATTGCGCGAAGACAGTATCGGCGCATTACAGCGGCTGCATCAATTGGGCT

ATAGTCTGGTGATGTTGACCGGTGACAACCCTATTACCGCCAATGCCATTGCGAAAGAAGCCGGTATTGA

TCGTGTGATCGCGGGCGTTCTGCCAGATGGCAAAGCCGATGCTATCAAGCAACTGCAAGCGGCCGGGCAT

AAAGTGGCGATGATTGGTGATGGCATTAACGATGCTCCTGCATTAGCACAAGCGGATGTCGGGATTGCCA

TGGGGGGCGGCAGTGATATCGCGATTGAAACCGCGGCGATTACCCTTATGCGTCACAGTTTGTACGGTGT

TGTCGATGCGGTAGAACTTTCCAAAGCAACATTACGTAACATGAAGCAAAACCTGTTGGGCGCGTTCTTC

TACAATGCGCTGGGCATTCCCATTGCCGCAGGGATTTTATATCCCTTCACCGGTACATTGCTCAGCCCGG

TCGTCGCCGGAGCGGCAATGGCGTTGTCGTCCATTACGGTCGTCAGTAACGCTAACCGTTTACTGCGCTT

TAAGCCTAAACAGTAA

>gi_332139403_Pantoea ananatis LMG 20103 chromosome_36

ATGTCAAACACGATTGTTCTGGCGCTGGACGGATTGTCCTGCGGCCACTGCGTAAAACGCGTCAAAGATG

CGCTCGAACAGCGCGACGATGTCGAGCTGGCTGAGGTTACTCAACAGGAAGCCAGCGTGTCAGGCCGTGC

AGACGCTACCGCGCTGATCGCTACCGTTGAACAAGCGGGCTATCATGCAACATTAAAAGCTGCGGGTTCC

AGCCCAAAGCCTGAGCCGTTGACAGCAGCAGAGCCGCCGCCGGAGGCGCTGACAACGGAGTCTTCTCCTC

TTCCGGCAACTGAAAATCAGCCCGCTCACGTGCTGTTAATCGACGGGATGAGCTGCGCCAGCTGTGTCAG

CCGGGTTGAGAAAGCCCTTGAGCAGGTAAGTGGTGTGCAACAGGCCCGCGTGAATCTGGCCGAACGCAGC

GCGCTGGTGATGGGCAATGTGGCACCCCATCTGCTGGTCGCCGCCGTGGATGCGGCCGGCTATGGGGCGG

AAGTGGTCGAAGACGAACAGCAACGACGTGAAAAGCAGCAGCGCAGCGCCCGACGCGCCATGCGACGTTT

TAGCTGGCAGGCGGGACTTGCACTGCTGTTTGGCGTGCCGTTAATGATCTGGGGCATGCTGGGCGACAAC

ATGATGCTCACCGAGGACAATCATACCACCTGGCTGGTGTTGGGCACGGTTACGCTGTTGGTGATGGTCG

CCACGGGCGGTCACTTCTACCGCAGTGCCTGGCGCAGCCTGCTCAACCGCAGCGCGACAATGGATACCTT

AGTCTCGCTGGGCACCGCCGCCGCCTGGATCTACTCCTTTAGCGTGGTGATTTGGCCGGCGTTCTTCCCG

CTGCAGGCGCGTCATCTCTACTTCGAAGCCAGCGTAATGATTCTCGGCCTGATCAATCTGGGCCATGCGC

TTGAACAACGCGCGCGTCAGCGTTCCTCGCGAGCCCTGGAACACCTGCTCGATCTGACGCCGGAACAGGC

AAGAGTCGTAACGGAACAGGGTGAAACCTCAATGCCGGTTAGCGCGGTACAGCCCGGCATGATCCTGCGG

CTGGTCGCGGGCGATCGCGTGCCGGTCGATGGCGAAGTCCAGGACGGTGAAGCCTGGTGTGATGAGGCCA

TGCTGACCGGTGAGGCGGTGCCGCAAAGCAAAACGCAGGGCGATCGTCTGTTTGCCGGCACCCTGGTGCA

GGACGGGACAGTACGCTTTATTGCCCGCGCGACCGGTGGCAATACGACGCTGGCGCGCATCATTCAGTTA

GTCCGTAAAGCCCAGAGCAGTAAGCCAGATGTGGGTCGTCTGGCCGATCGTATTTCTGCCATGTTTGTGC

CGGTGGTGGTGCTTATCGCCCTGATAAGCGCCGCAGTCTGGTATACCGCCGGTCCGGCGCCTCAGATTGC

CTACGCGCTGGTGGTGGCCACCACAGTGCTGATTATTGCCTGCCCCTGCGCGCTGGGCCTGGCCACGCCC

ATGTCGATTATCGCCGGAACCGGCCGCGCCGCCGAATACGGCGTGCTGGTACGCGATGCCGATGCACTGC

AGCGCGCCAGCCGTATCGATACGCTGGTGTTTGATAAAACCGGCACGCTGACAAGGGGCACACCGCAGGT

GACCGAGGTGATGAGCTGGAACGGCAGCACGCGCGATTCGGTCCTGTCTTCGGCGGCGGCGCTGGAACAG

ACATCGGGTCACCCACTGGCGAAAGCGATCCTGGCGGAAGCGGGTACCGCAGCAACGCCCGCGATTGCGC

AATTTCGTACAATACGCGGCAAAGGCGTAAGCGGCATACTCAATGGCAGACAGCTGTTACTGGGTAATGT

CAGTCTGTTACACGATTACGGCGTGGATTGCCAACCGGCGCGGGCTGAAATTGACAGGCTCACCCGGCAA

GGGGCAACCCCGGTATTACTGGCACAGGATGGACAGCTTACCGGTCTGATTGGCTTGCGGGATACGCTGC

GCCCCGAAGCGAAAGCAGCACTGCAGCGGCTGCATGCAGCGGGTTACCGACTGGTTATGCTGACCGGTGA

TCATGAAAATACGGCCCGGGCTGTTGCGGATGAAGTCGGGATTGATGAGGTGATTGCAGGCGTACTGCCC

GAAGGTAAAGCCGATGCGATAAGCCAACTACAGCAGCAAGGAAGGCAGGTTGCCATGGTGGGCGATGGTA

TCAATGATGCTCCTGCACTGGCGCAGGCTGATGTCGGTATTGCGATGGGCGGCGGCAGCGATGTGGCGGT

AGAAACCGCAGCGATTACGCTCATGCGGGCCGATCTGCATGGCGTGGTGGATGCCTTAAAGATTGCCTCC

GCCACCCTGGGCAATATCCGGCAGAATCTGTTTGGCGCGTTTATTTACAACGTTATCGGTATTCCGCTGG

CGGCAGGCGTGCTCTTTCCTTTTACCGGCCTGTTGCTTAGCCCGGTTTTTGCCGGTGCCGCCATGGCGCT

GTCGTCAATAACCGTGGTGAGCAATGCTAACCGCCTGTTGCGCTATAAGCCACGCGTTTAG

>gi_172087630_Vibrio fischeri ES114 chromosome_37

ATGAGTCAAGAATTCAATCTGCCTTTAGCGGGATTAAATTGTGGTCGTTGTATTGCGAAAATAGAAAAAG

CATTTGCAGACAATCCGCATGTACAGCAATACTCGGTAACTAAAACGGATACAAAGCTAACCACTACTCT

CTCTTATCAAGAGGTGGTTAAATTAATCACAGAGCTTGGATATTCCGTCCCTAAAGCACAACATGTCACT

CTTTATTTGTCTGGATTAAATTGTGGTAAGTGCGTCAATAAAACCAAAGAAGCGTTAAGTGGTTTATCAC

ATTCCAGTATTACGTCTATTTCAACAACACAATTGAAATTAGAAACCACTCACTCGCAACAACATATTAT

CGAACTCATTGAGTCTATTGGCTTTCATGCAAGTAACCAGTCACCTGTATCTGATAATAAAGAGAATCTA

TCTCCTCAGTCTGATAATCCCAAAACAGACAATACCAACTATTCATCAGAAAAAACGACACAATTAATAT

TGTCAGGAATGACATGTGCGAGTTGCGTATCTTCTGTTGAAAAAATCATTGCGCAAAATAAAAACGTACA

CTCTGTGACGGTTAATCTTGCTGAACGCACCGCGCTCGTTCATGGCGATATTGATGTACCAACATTAATC

AAAAGCATTGAAGATGGGGGTTATGGCGCAGAACTAAGCGTAAGTGAAGAACAGCGCAGAGTACGACAAA

AAGAGCAATTTCTAAAAACAACCGCTAAACATAAAAGAAATACTATTTTATCACTAAGCATCGGTGCTCC

GCTCATGTTGTGGGGCGTTTTCGGTGGCAATATGCAAATATCAAATACTCACGACCAAATCGCTTGGGGA

GTAATTGGAGTTATTTGTTTAGGGTTATTACTGACTGCTGGTCGACACTTTTTTGTGAATGCATGGAAAG

CCTTTACTCACCATAGAGCAACAATGGATACCTTAGTTGCATTAGGCACAGGCTCTGCTTGGCTATATTC

CATGGCACTAGTTCTTTTTCCTGAATTCTTCCCAGAACAAGCTCGTCATGTGTATTTTGAAGCAACAGCA

ATGATCTTAGGCTTAATTACACTGGGTAACCTCATTGAAACTAAAGCCAGAAATCGCACGTCAAATGCCA

TTGAAAAACTGATCAACTTACAACCACAAACGGCTACCGTTATTATTGATAGCCAAGAGGTTACCTTGCC

TGTCGCTCAAGTTGTTGAAGGAATGGTAATTAAAATTAAACCCGGTGAAAAAATTCCCGTTGATGGCACT

ATCTTGAGTGGTGAGAGCTATCTTGATGAAGCCATGTTAACTGGAGAGCCAATACCGAGCTTAAAATCCT

TAGGTAATGCCGTACATGCCGGTACCATCAATCAACAAGGAAGCTTTACCTTTAAAGCGACGAAAGTTGG

TGAGCACACCATGCTCGCTCGTATTATTACTATGGTACGAAATGCACAGAGCAGTAAACCTAAATTAGCT

AAATTGGCAGATAAAATATCATCAATCTTTGTTCCGAGCGTTATGATTATCGCGATTCTATCCGCTCTTA

TTTGGTATAACTTTGGTCCAGAGCCAACATCAAGTTACATGTTAGTTGTCGCCACTACAGTTCTAATTAT

TGCTTGTCCTTGCGCCCTTGGATTAGCAACGCCTATGTCGGTAACCGTAGGAATTGGTAAAGCAGCTGAA

TTTGGTGCATTAATTAAAGATGCTGACGTACTTCAAACTGCAAGCACCATCGATACGGTTGTTGTAGATA

AAACAGGAACATTAACGGAAGGGAAACCAAGGGTAAATCACGTCACCCTTTTCAACCAATACGATGAAAA

AGAAGTAATTTCACTCGCAGCAGCAGTCGAAACTCATTCTGAGCATCCATTGGCGTTAGCGGTTGTGAAT

TATTTAAATGACAACCATTGGATACCTCTTACTAGTACAAATTTTACTGCCGAACTGGGCCTAGGTGCAT

CAGCGATAGTAAATGGTAATCTCATATTTATTGGTAACCATCGTTACCTTACTAAGAACAACATACCAAC

TCCTGATGTATCTATTTCACCATCAGCTACGCCTTTATATGTGGCTAATGAGCAAGAGTGTATCGGGGTT

ATTCAGGTTAGTGATGCCATTCGCTTAGATTCCAAAGCTGCTATCAAGCAATTTAACCAACTTGGTATTG

ATGTTGTCATGTTAACTGGCGATCGTAAAGAGACTGCAGAGCACGTAGCAAAAGAACTAGGGATCAATCA

AATCATTGCTGGAGTATTACCGGATGGTAAAGCGCAAGCAATCCAAGAACTGCAAAAACAAGGCAAAAAA

GTGGCCATGATAGGTGATGGTATTAATGATGCCCCAGCACTAGCACAAGCTGAAGTCGGTATTGCAATGG

GCAATGGCAGCGATGTTGCAATTGAAAGTGCACATTTAACGTTAATGAATCACTCTTTATTAACTGCAGT

CAATGCCATTACATTATCCAAAGCAACCATGAAAAATATGAAGCAAAATCTATTTGGTGCATTTATATAC

AATACCATTGGGATCCCTGTTGCGGCAGGCATTTTATTCCCATTTACTGGCATGCTACTAAGTCCAGTCG

TTGCAGGCGCAGCAATGGCACTTTCATCTATAACGGTCGTCACCAATGCAAACCGGTTACGACTTTTTAC

TCCCCAACCTCAACAAAAATAA

>gi_45439865_Yersinia pestis biovar Microtus str. 91001 chromosome_38

ATGTTGCAAACCACACTGCTCGCCTTACAAGGACTTTCTTGTATGAATTGTGCTCAACGCGTTAAAGCCG

CGTTGGAAAGCCGTGAGGATGTTCACCATGCTGAAGTGAATGTTCACTACGCTAAAGTGACTGGTGAAGC

CGACACTCATGCACTCATTGAAACGATCAAACAGACCGGTTATCAGGCTACAGAAGCCCAAACGCCGGAC

GTTGAATTACACCTATCAGGATTGAGCTGCGGGCATTGCACTGAGACCGTCCGTAAAGCGCTGGAAGCGG

TTTCGGGGGTTATTTCTGCCGATGTAACCCTCGAGAGTGCCAACGTGTACGGCAAGGCCGATATACAGAC

ATTGATTGCCGCAGTGGAACAGGCTGGCTATCACGCCACACAGCAAGGTATCGACTCCCCAAAAACTGAG

CCGCTGACACACTCAGCCCAATCTCAGCCGGAATCTCTGGCAGCGGCCCCGAATACAGTTCCGGCAACAA

ACGTGGCTTTAGCCACAAGTACGGTTTCTGATACAAATACCGTTTTACCTACAAATACCGCTTTACCTAC

AAATACAACGTCGACAACTTCAACAGCCGATACGGCGTCAGCCACCAGCACGGCACCGGTGATCAATCCG

CTGCCAGTGACCGAATCAGTGGCCCAGCCGGCAGCCTCAGAAGGGGAGAGTGTTCAGCTATTACTGACGG

GGATGAGCTGTGCCAGTTGTGTCTCTAAAGTACAAAATGCATTGCAACGTGTTGATGGGGTTCAGGTGGC

CCGCGTTAATCTGGCTGAGCGCAGTGCATTGGTCACAGGGACCCAAAATAACGAAGCCTTAATCGCTGCG

GTTAAAAATGCCGGTTACGGCGCGGAAATTATTGAAGACGAAGGGGAGCGACGCGAGCGGCAACAGCAAA

TGTCGCAAGCCAGTATGAAGCGCTTCCAATGGCAAGCGGCATTAGGTCTGTTGTTAGGTATCCCATTGAT

GGCCTGGGGGCTGTTTGGCGGTAGCATGACGCTGACGCCAGAAACCCAAACGCCCTGGCTTATCATCGGG

ATTATCACGTTGCTGGTAATGATATTTGCGGGTGGTCACTTCTACCGTAATGCGTGGGTCAGCCTGAAAA

ATGGCAGTGCGACCATGGATACCTTGGTCGCTCTGGGGACAGGTGCGGCCTGGATCTATTCCATCACCGT

CAATATCTGGCCAGATGTTTTCCCTATGGAGGCTCGTCACCTTTATTACGAAGCCAGTGCCATGATCATT

GGTTTGATTAACCTTGGTCATGCGATGGAACAACGTGCCAGACAGCGCTCATCTAATGCGCTCGAACGCC

TGTTAGATTTGGCCCCACCAACGGCAAAGTTAGTGACCGATGACGGCGAGAAGGTGATCCCGCTGGCTGA

TGTCCAACTGGGGATGATCCTACGCCTGACTACTGGCGATCGCGTACCGGTTGACGGCGAGATTGTGCAG

GGCGAAGTGTGGATGGATGAAGCGATGCTGACTGGTGAGCCGATCCCACAACAGAAATCAGTCGGTGATA

TTGTCCACGCGGGGACACAAGTACAAGATGGAACCGTACAATTTCGGGCTAGCGCTATCGGTAGCCAAAC

GACCTTGGCGCGGATTATCAAACTAGTCCGTCAGGCACAAAGCAGTAAACCGGAAATCGGCAAATTGGCA

GACCGTATCTCTGCGGTATTCGTCCCAACGGTGGTGGTCATTGCCATTGTCGCTGGCCTGATTTGGTATT

TCTTCGGCCCACAACCCCAGTTGGTCTATACCCTGGTGGTGGCCACCACGGTGTTAATTATCGCCTGCCC

TTGTGCTTTGGGCTTAGCGACACCGATGTCGATTATCTCTGGCGTCGGCCGGGCCGCTGAATTTGGTGTA

TTGGTTCGAGATGCAGATGCATTGCAGCAAGCCAGTAACCTCGATACGTTAGTGTTCGATAAAACCGGCA

CACTGACTGAGGGGCACCCGCAGGTAGTGGCAATCCATACCTTTAATGGTGTCAGTGAGCAACAGGCTTT

GGGCTGGGCGGCAGCATTGGAAACGGGATCAAATCACCCGCTGGCGCGCGCTATCTTACAACGTGCTGAA

GGGCTGACATTGGCCACCGCCAGCCAATTTCGTACATTGCGCGGTTTGGGGGTCAGTGGTGAAGTTGACG

GCATTCCACTGCTCCTTGGCAATAATCGGCTACTGGAAGAGCAACAGATTGATACCCGCGAGCTGCAATC

ATTGATACAGCAACAAGCGGAAAGTGGCGCAACACCGGTTATTCTGACCGCCAATGGTAAGCCCGCCGCA

CTGTTATCGATCCGTGATCCATTGCGCGAAGACAGTATCGGCGCATTACAGCGGCTGCATCAATTGGGCT

ATAGTCTGGTGATGTTGACCGGTGACAACCCTATTACCGCCAATGCCATTGCGAAAGAAGCCGGTATTGA

TCGTGTGATCGCGGGCGTTCTGCCAGATGGCAAAGCCGATGCTATCAAGCAACTGCAAGCGGCCGGGCAT

AAAGTGGCGATGATTGGTGATGGCATTAACGATGCTCCTGCATTAGCACAAGCGGATGTCGGGATTGCCA

TGGGGGGCGGCAGTGATATCGCGATTGAAACCGCGGCGATTACCCTTATGCGTCACAGTTTGTACGGTGT

TGTCGATGCGGTAGAACTTTCCAAAGCAACATTACGTAACATGAAGCAAAACCTGTTGGGCGCGTTCTTC

TACAATGCGCTGGGCATTCCCATTGCCGCAGGGATTTTATATCCCTTCACCGGTACATTGCTCAGCCCGG

TCGTCGCCGGAGCGGCAATGGCGTTGTCGTCCATTACGGTCGTCAGTAACGCTAACCGTTTACTGCGCTT

TAAGCCTAAACAGTAA

>gi_156932229_Cronobacter sakazakii ATCC BAA-894 chromosome_39

ATGTCAAACACCATAGAACTGGCCCTGGACGGGCTTTCCTGCGGACACTGCGTAAAACGGGTGAAAGAGA

GCCTGGAGCAGCGCCCGGACGTTGAACGCGCGGATGTCACGATTGAACATGCCAGCGTCACCACTACCGC

GCCTGCGGACGCCCTGATTGAGACGGTGAAACAGGCCGGTTACGACGCGACCCTCGCACACCCAAAGGCT

AACCCGCTGACAGAGTCATCAACCCCGTCGGAAGCACTGACAGCGGCGCAACCTGAGCTTCCGGCAGCGG

ATAACGCCGACGATGACGACAGCCGACAGCTGCTTATCAACGGCATGAGCTGCGCCAGTTGCGTGAGCCG

CGTTCAGAACGCCCTGCAAGGGGTGGCGGGCGTCACGCAGGCACGGGTGAATCTGGCGGAGCGCACGGCG

CTGGTGATGGGCCATGCCTCCGCCGCTGATTTAATCGCGGCGGTTGAAAAAGCGGGCTACGGCGCAGAGG

CTATCGATGACGACGCCGAACGCCGCGAGCGCCAGCAGCAGACCGCGCAGGCGGCCATGAAACGCTTTCG

CTGGCAGGCGGCGCTCGCGCTCGCCGTCGGCATTCCGGTGATGATCTGGGGCATGTTCGGCGACAACATG

ATGGTCACCGACGCCAACCGTTCGCTCTGGCTGGTCATTGGGTTTATCACGCTCGGCGTGATGGTTTTCG

CGGGCGGTCATTTTTATACCAGCGCCTGGAAAAGCCTGCGCAACCGCAGCGCGACAATGGATACGCTGGT

GGCGCTCGGCACCGGCGCGGCCTGGCTCTACTCCATGAGCGTCAACCTCTGGCCGCAGCATTTTCCGATG

GAAGCGCGTCATCTCTATTACGAAGCCAGCGCCATGATTATTGGTCTGATTAATCTTGGTCATATGCTGG

AGGCGCGCGCCCGCCAGCGTTCATCGAAAGCGCTGGAACGCCTGCTGGATCTGACGCCGCCGGTTGCGCG

CGTTGTCACGCCGGAAGGTGAGAAAAGCGTGCCGCTGGCGGAGGTGACGCCGGGCATGACGCTGCGCCTC

ACCACCGGCGATCGCGTCCCTGTGGATGGCGACATTACCGACGGCGAAGGCTGGTTTGACGAAGCGATGC

TGACCGGCGAGCCGGTGCCGCAGCAAAAAGCGGCGGGTGATGCGATCCACGCGGGCACCGTTGTGCAGGA

TGGCAGCGTGCTGTTTCGCGCCGCCGCGGTGGGCAACCACACCACGCTGTCGCGCATTATTCGCATGGTG

CGCCAGGCGCAGAGCAGCAAGCCGGAAATCGGCAAGCTGGCCGACCGCATCTCCGCGGTTTTTGTTCCGG

TCGTGGTGCTGATTGCGCTGTTCAGCGGCGCGGTCTGGTATTTCGCAGGCCCGGCCCCGCAGATCGTCTA

TACGCTGGTGATTGTCACCACGGTGCTGATTATCGCCTGCCCCTGCGCGCTGGGCCTTGCCACGCCGATG

TCGATTATCTCTGGCGTCGGGCGCGCGGCGGAGTTTGGCGTACTGGTGCGTGACGCCGACGCGCTGCAAC

GCGCAAGCGACGTCGACACGCTGGTGTTTGACAAAACCGGCACGTTAACCGAAGGCAAACCGCAGGTGGC

GGCCATCGCCACCACGGGCGACATCACCGAAGCGGACGCGCTGCGCCTCGCCGCCGCGCTGGAGCAAGGC

TCCAGCCACCCGCTCGCGCGCGCCATTATCGAACGTGCCGGCGGCGCAGACCTTCCCGCCGTCAGTGGTT

TTCGCACGCTGCGCGGGCTGGGCGTTAGCGGTGAGGCGCAGGGCAAAACGCTGCTGCTCGGTAATCAGGC

GTTGCTGGAGGAAAACGGTATTCCGACCGACGCGCTGCGCGATGAGGCGCAGCGTCAGGCAAGCCTCGGC

GCCACGCCGGTGCTGCTGGCGGTGGACGGTACGCTGGCGGCTCTCTTCGCCATTCGCGACCCGCTGCGTG

AAGACAGCGCCGATGCACTGGCGCGCCTGCACCGCGAAGGCTACCGGCTGGTGATGCTGACCGGCGACAA

TGCCGTCACCGCGAACGCCATCGCCCGCGAGGCGGGCATTGATGAAGTGATTGCAGGCGTCCTGCCGGAT

GGCAAAGCACAGGCGATTCAGAAGCTTCAGCAGGCGGGCCGCAAAGTGGCGATGATTGGCGATGGCATTA

ACGACGCCCCGGCGCTGGCCCAGGCGGAAGTGGGGATCGCGATGGGCGGCGGCAGCGATGTGGCTATTGA

AACCGCCGCGATTACCCTGATGCGCCACAGCCTGACGGGAGTCGCCGACGCGATGGCGATTTCCAAAGCG

ACGCTTGGCAATATGAAACAGAACCTGCTCGGTGCCTTTATCTATAACGCACTCGGCATTCCCGTGGCGG

CAGGCGTGCTGTGGCCGCTGACCGGCACCCTGCTGAACCCGGTGGTCGCAGGCGCGGCGATGGCGCTCTC

ATCGATTACCGTCGTCAGTAACGCCAACCGGCTGCTGCGGTTTAACCCGAAAAAATAA

>gi_82542618_Shigella boydii Sb227 chromosome_40

ATGTCACAAACTATCGACCTGACCCTGGACGGCCTGTCCTGCGGTCACTGCGTTAAACGCGTGAAAGAAA

GTCTCGAACAGCGCCCGGATGTAGAACAAGCGGATGTGTCTATCACTGAAGCGCACGTTACCGGGACTGC

CAGTGCAGAACAGCTGATCGAAACCATCAAACAAGCGGGTTATGACGCATCTGTAAGCCACCCAAAGGCT

AAACCGCTGGCGGAGTCATCAATCCCGTCGGAAGCACTGACAGCGGTTTCTGAGGCGCTTCCGGCAGCGA

CCGCCGATGACGATGACAGCCAGCAGTTGCTGCTGAGCGGCATGAGCTGCGCCAGCTGTGTCACCCGCGT

ACAAAATGCGCTGCAAAGCGTACCGGGCGTCACTCAGGCACGGGTAAACCTGGCGGAGCGTACTGCGCTG

GTGATGGGCAGTGCCTCCCCACAAGATTTAGTGCAGGCGGTGGAAAAAGCGGGCTACGGCGCGGAAGCGA

TTGAAGATGACGCGAAACGCCGCGAGCGCCAGCAGGAAACCGCCGTCGCTACGATGAAGCGCTTCCGCTG

GCAGGCGATTGTCGCACTGGCGGTGGGTATTCCGGTAATGGTCTGGGGGATGATCGGCGATAACATGATG

GTCACCGCTGACAACCGCAGCCTGTGGTTAGTTATCGGCCTGATAACCCTGGCGGTAATGGTTTTCGCCG

GCGGGCATTTTTACCGCAGCGCGTGGAAAAGCCTGCTGAACGGTGCGGCGACGATGGATACGCTGGTGGC

GCTGGGTACGGGTGTGGCGTGGCTCTATTCGATGAGCGTCAACCTGTGGCCGCAGTGGTTCCCGATGGAA

GCGCGGCATCTTTACTATGAAGCCAGTGCGATGATTATCGGTCTGATTAATCTCGGCCATATGCTGGAAG

CGCGCGCGCGTCAGCGTTCTTCTAAGGCGCTGGAGAAATTACTCGATTTAACCCCACCGACGGCGCGAGT

GGTCACCGCAGAGGGTGAAAAAAGCGTGCCGCTGGCGGAAGTTCAGCCAGGGATGTTACTGCGCCTGACG

ACCGGCGATCGCGTGCCGGTAGATGGCGAGATTACCCAGGGCGAAGCATGGCTGGATGAAGCGATGCTGA

CGGGCGAACCAATCCCGCAGCAAAAAGGCGAAGGCGAAAGCGTCCATGCCGGGACAGTGGTACAGGACGG

TAGCGTGCTATTTCGCGCCAGTGCGGTTGGCAGCCATACCACGCTGTCAAGGATTATTCGCATGGTGCGC

CAGGCCCAGAGCAGCAAGCCAGAAATCGGTCAGCTGGCGGATAAAATCTCAGCCGTATTTGTGCCGGTAG

TAGTGGTGATTGCGCTGATCAGTGCAGCAATCTGGTATTTCTTTGGTCCGGCACCGCAGATTGTCTATAC

CCTGGTGATAGCCACCACGGTGCTGATTATTGCCTGTCCGTGTGCGCTGGGGCTGGCGACGCCAATGTCG

ATTATTTCCGGCGTCGGGCGGGCGGCTGAGTTTGGCGTGCTGGTGCGGGACGCCGACGCGCTGCAACGCG

CCAGTACACTCGATACCGTCGTGTTCGATAAAACCGGGACGCTGACCGAAGGGAAGCCGCAGGTTGTCGC

AGTAAAAACATTTGCTGATGTTGATGAAGCGCAAGCATTGCGTCTGGCGGCGGCGCTGGAGCAAGGTTCC

AGCCATCCGCTGGCACGAGCGATCCTCGATAAAGCAGGCGATATGCAGCTGCCGCAGGTCAACGGTTTCC

GCACATTGCGCGGGCTGGGCGTAAGCGGTGAAGCTGAAGGTCATGCGTTATTGCTGGGTAATCCGGCGCT

GTTAAATGACCAGCAAGTCGATACTAAAGCGATTGAAGCCGATATTTCAGCTCAGGCATCGCAAGGAGCA

ACGCCTGTGCTGCTGGCGATTGACGGGAAAGTAGTAGCCCTGCTGGCTGTACGCGATCCGTTGCGTAGTG

ATAGCGTGGCGGCGCTGCAACGCCTGCATAAAGCGGGATATCGTCTGGTGATGTTGACCGGGGATAACCC

AACCACCGCCAATGCGATCGCCAAAGAAGCAGGGATTGATGAGGTAATCGCCGGGGTGCTGCCAGACGGT

AAAGCCGAAGCGATCAAACGTCTGCAAAGTGAAGGACGTCAGGTGGCAATGGTGGGCGACGGCATTAACG

ACGCGCCAGCGCTGGCTCAGGCGGATGTCGGTATTGCGATGGGTGGCGGCAGTGATGTTGCCATTGAAAC

CGCGGCGATTACCCTGATGCGCCATAGCCTGATGGGTGTTGCAGATGCGCTCGCGATTTCCCGCGCAACG

CTGCGCAATATGAAGCAGAACCTGCTCGGTGCGTTTATCTACAACAGCATTGGTATTCCGGTCGCCGCTG

GTATTTTGTGGCCGTTTACTGGAACACTGCTTAACCCGGTAGTTGCCGGAGCGGCAATGGCGCTCTCGTC

GATTACCGTAGTGAGTAACGCCAACCGGTTGCTGCGGTTTAAACCGAAGGAATAA

>gi_397665626_Legionella pneumophila_41

ATGTTACAAAAGGATTTGCGTTGTCTAATAAGGAATTATTGCTTTATTTTTTCCTTTTTACTGCTTAATT

CTTCTGCTTTGTTTGCCCAGCATGAGCAACATGGTGCCTCAACACAACCGTTACCTTCTCCCAAGACTAC

ATCGTCTCAATCGAAACCTGCACAATACAAAATGAAACAACCTATCGCTAAAAGCCAACCTGTTAGAACA

GTGAAACCTCTCACTATGACAGGTGGAGCAAAGCGTACGGTTAATTTGGTGGTTGCTTATAAAACGGTGA

ATTTTGCAGGCAAACTAAGACGCGCTATTGCTGTTAATGGGCAAATTCCAGCACCGACGTTGCATTTTAA

AGAAGGGGATGATGTCACCATCAATGTGTATAACCATTTGGATGAGGGAACGTCCATTCATTGGCATGGT

CTTTTAGTCCCTTGGCAAATGGATGGGGTGGACGGGGTGAGTCAAAAACCAATTCCTCCGGGAGGCGTGT

TCCATTATCGCTTTAAGCTTCACCAAAGGGGAACCTATTGGTACCACGCCCATGCCAAGGTTCAAGAACA

AGAAGGGTTGTATGGTACTTTTATTATTGATCCACCCAATCCTCCAAGCTATCACTACACCAAAGATTAT

GTGGTGGTCTTATCCGATTGGAGCAACACTCGCGCCGAGCAAGTGCTTGCGAATTTAAAAAAAGACGGCG

ACTATTACGGTCCTCGATTTCCCCTACAACCCTCGCTTATGAAGTTTCTTCATGATTATCGTAAAGCTTC

TCCTGAAGAGCGTAAAAAATTAATCGCTGATTATAAAATGATGCAACAAATGCGCATGAGTATCTATGAT

TTAAGTGACGTGGCTTATGATGCCTATTTATTAAATGGTCACCCAAAATCTCATCCTTGGACTGCCCCTG

TGAAGGTAGGGGATAGGGTGCGACTGCGCTTCATTGGCGCAGGAGCAAGCACCATCTATCGTGTTAAAAT

TCCTGATGCCAAAGTGGAGATGGTGCACATTCAAGGAAATGATGTGAGCCCTTATCCCATTGAGGATTTT

TGGATTGCACCAGGTGAAACCTATGATATCCTGGTGACCATTCAAAAAAACAAGCCTTACATTATTTATG

CCGAGTCGATTGACACGCTGGGCAAAGCTTATGGGGCCTTAGTGACCCATCCTAATCAAGTCGTCAACTA

TCAGCACGTGACTCCTTTCCCTGAACCACTTCCTGTCACAAGAGAAATGATGGCCAACATGATGATGTCT

ATGAATGGCGGGGGAATGGGTGGAAATCAACAACACGCCTTGATGAACAAAAAAACATCATCAACGGCGA

TGAAACCCTCCATGACCATGCCTTCGCACTCTCAAGCAATGAGTTCGCAAAGTGCTAATCATTCGATGTC

CTCTAAGACTATCCAAAAGAAAATGGACAAGGCATCCAATCATTCCGCTCACACCTCATCAATGAAGTCC

AAATCCTCATCGACCTCGATGAACAAGAGTATGGACATGCCAGGTATGAATCATGGGGCAATGAGTCAGA

ATAAGACGTCTAACTCTGCTCAAATGAAATCTGACTCCTCGTCGACCTCAATGAACAAGAGCATGGCCAT

GCCAGGTATGAATCATGGGGCAATGAGTCAGAGTAAGACGTCTAACTCTGCTCAAATAAAATCTGACTCT

TCGTCGACCTCAATGAACAAGAGCATGGCCATGCCGGACATGAATCATAGCGCAATGGGCCAGAATAAGA

CGTCTAACTCTGCTAATGACACGTCTATGAATAGGAACATGAAGTCTGACAGGCCTATGGAGCAGGGCAT

GTCGATGAATGGCTCCATGAACATGAAGATGCCCATTGAGCCGACCATCATAGGCGATAAAATAGAGCCA

CCTGATTCGGCAAAAGCCACCACCTTAGGAACCAAATACCAAGAGTTAAAAGGGGCAGTGAAAACCAATA

ATCCGAATAAACCAGTCGATGGGATTATTAAAATGGAATTGTTTGGCTACATGGACCGTTATATCTGGTT

TATTAATGGTCTACCGGAATATAAGGCCAAACCGATTTTGATTGAGCCAGGAAAGCGTTATCGGATTATA

TTTACCAATAATTCGATGATGCGTCACCCCATGCATATTCACGGCCATTGGTTTATTTTACGCAATGGTC

ATGGGGCTTACGATCCGTTATTGCATACCATTGAAGTCCCTCCAGGAGCGACTGCCGTTGCCGATTTTGA

TACTGAAGCCAGCGGTCAATGGTTTTTCCATTGCCACCATCTTCTGCACATGACCGCGGGCATGGCGCGC

GTATTTCAATACACCACCATCATTGAGATTGCCAACGGCACTCGAAAGCCAGACAATTACGCCTATCAGC

AAGCCTACATCAATCGGCCGGTCGTACGAGAAGACGAAGTCATGCCACTGGATGCTTCTCTCATCAAACA

CCCTGCAGGACATCATCAAGGCTTCTATCGTTCAAGCTATATCGAGCTGGGCGAAGACCCTTTTCATAAT

GCTCAGGAAATGACGTTTAGAGGCCTCTATGGCCCAGATTACAATAAGCTCCAGTTGTATACTGAGGACG

CTGAAATTTATAAAGGAACCGTTGAAAACGCAGACATCGATGTGTTCTATTGGCATTTGATTAGCCAATT

TTGGGCGGTTAAAGGAGGAGTCAATTATTTTTATCGCCCGGGAGGCCCTTATTGGCAACCTGGTATTGGT

ATTGAAGGGTTAATGCCTTGGTATATTGATACCAACATTAGAACGTATTATCGTGATGGCAGTGTCAAGT

TTGACATTCAGTTAGCACGGGCTAACCAGCTGACCAATAACTTCTTCTTCCTCACAGGAATTCGCAGCAT

TTTGGCCACTCATACCGTCGTCAAAAACGAGATTGGCAATGGCTTAAACCAAATGCGTTATATCCTGAGA

CCTTATTATCGTCTTAAGCCAGGACTTAATATTTTTTCAGAGTATGAGCATGATGTAGAGTATGGTGCTC

TTAAGAGGATTCTTCGAAGTCAGGGAGAGCCTACCACTCAAGATACAATCACGTTAGGAGTTGCTGTTCT

TTTTTAA

>gi_110832861_Alcanivorax borkumensis SK2 chromosome_42

ATGTCGACGCAAGCGCGCACCCAAACTCTGCCCATTCGCGGCGCGACCTGCAAAGGATGCAGCCGCACCA

TCACCGCTGCACTGGAAGCACTCACCGGCGTGGAGCGTGTGACGGTGGATTTGGAGTCCCAGCAGGTGTC

CGTCACCGGCACCGCCTCGCACAGTGCGCTTCAAGAGGCGCTGGTACAAGCCGGCTACGGCGTAAACCCG

GATGATGCTCACCATGAACACAATAATCCCGCCGACCATGACAACACCGCCAGTAAGCCGATCGCCCAGG

AGCCGCAATCAATACAGCTAAATATCAGCGGCGCCACTTGCGCGTCCTGCGTGCGCACCATCGAAAGCGC

CCTGCGTGACACACCCGGGGTCGACAGTGCGGATATGAATTTTGCCGACCGCACCGCTCAGGTGAGCGGC

TCCGCCAGCACCGCCTCCCTCATTAAAGCGGTAGAAGACGCGGGTTATGGTGCCAGCGAAGCCCGCGAGG

AGGATAACGGAGAGCAGGAAGCCAAGGAGCGGGCACATTACCACCAGTTGCTGCGGCACATGGGGCTAGG

GCTTGGGCTGGGCGTGCCACTAATGGCCTGGGGCCTGCTCGGTGGTGACATGATGGTTCGCCCCGGTGAA

ACCAGCCAATGGGTATGGCTGGGTATCGGCGTTGCCACCCTCGCGGTGCTGGCCACCGCCGGCCGACACT

TTTTCGTCGGCGCCTGGAAAGCCTTTCGCAACCATAACGCCAACATGGACACCCTGATCGCACTGGGCAC

CGGTGCCGCCTGGCTGTATTCCATGGTGGTGGTATTACTGCCCGGCGCCCTGCCGGAAGCCGCCCGCCAT

GTGTATTTTGAGGCCAGCGCCATGATTATCGGCCTCATCAATCTTGGCCAGGCACTGGAAGTTCGCGCCC

GCGGCAAGACCAGCGCCGCGGTAAAACGGTTGCTGGATTTGGGCGCCAAAACTGCACGGGTCATCCGCGA

TGGCGAGGAACAGGATATCCCGGTGGAGCAAGTTCAGGCCGGCGATCAATTGCGGGTTCGCCCTGGCGAA

AAAATTGCTGTGGACGGCACCGTGGAAGAAGGCGAAAGCCGCATTGATGAATCCATGCTCACCGGTGAAC

CCATGCCGGTGAATAAAGCCCCTGGCGATAAGGTCAGCGCCGGCACACTCAACGAAGCCGGCACGCTACT

TTATACCGCCACCGCCGTCGGCAAAGACACCGCCCTTTCACACATCGTCGCACTGGTAAAAAAAGCCCAA

GGCAGCAAGCCACCCATCGGGCGACTTGCCGATCAGGTGTCGGCCATTTTTGTCCCGACCATTATGCTGA

TTGCCATCGCGGCGGCACTGGTGTGGTTCAACGTGGGCCCGGAACCGCGGCTAACCCATATGCTGATCAG

CGCCACCACTGTATTGATTATCGCCTGCCCCTGCGCACTGGGCCTGGCCACGCCCATGTCGGTGATGGTG

GGCGTCGGCAAGGCCGCCGAATACGGCATTCTGATTCGCCAGGGCGAAGCGCTACAAACTAGCAGCCAGC

TGGACACCATTGTCCTCGACAAAACCGGCACCATCACCGAAGGCAAACCTGCCGTCACTGATATCGTCTG

CAATGAGGGCCAGCAAGAAGACACCGTGCTAACCCTTGCCGCCAGCGTGGAAAGCGGGTCGGAGCACCCG

CTGGCCACGGCGATTCTCAACGAGAGCAAGCAGCGAGAGCTATCACTGGATAGCGCCAGTGAATTCCAGG

CTTTAAACGGCAAGGGGGTTACCGCCACCATCCAGGGCAAAACCTATCGACTAGGAAACCGCCGCTGGCT

AGAAAGCGAAGGGGTAAACATCAACCTCGATAGCACCTCTCTCACCGACAAGGGCGTCACGCCGCTGTTT

TTGTCTGAAGCGAACACCCTGCTCGGCATCATCGGCGTCGCGGATCGTATCAAGGACGATTCCAAAGCCG

CCATCGCCCGCCTGAAAGATCAGGGATTAAAGGTCATCATGATTACCGGCGACATTGCCGCCAGCGCCAA

CGCCATTGCCGAACAAGCCGGGGTGGATGAAGTGATGGCTGAAGTGCTACCGGAAGACAAGGCGAAAAAA

ATCCAGGCATTGCAGAGTGAAGGGCGCACCGTGGCCATGGTCGGTGATGGCATCAACGACGCCCCCGCTC

TCGCCCAGGCCGATGTGGGCTTTGCTATTGGCACCGGCACCGACGTAGCCATCGAATCCGCGGCCATCAC

CTTGATGGGAGGCTCGCTACACGGCGTTCCCGATGCCATGGCGATTTCTTCTGCCACAGTCCGCAACATT

AAACAAAATCTGTTTGGTGCCTTTGTGTATAACAGCCTGGGCGTACCAGTGGCCGCCGGCATCCTCTATC

CACTCACGGGCAATCTGCTTAGCCCTGTGATCGCCGGGGCCGCGATGTCGCTCAGCTCGGTTACCGTCGT

CACCAACGCCAACCGATTGCGGTTTTTCAAACCCAAACACCATCAGGGAACAAGCGCATGA

>gi_336246508_Enterobacter aerogenes KCTC 2190 chromosome_43

ATGTCTAACACTATCGACCTGACGCTTGATGGCCTCTCCTGCGGCCACTGCGTTAAACGCGTGAAAGAGA

GCCTCGAACAACGTCCCGACGTTGAACAGGCTGATGTAACCGTTACCGAAGCCCACGTCACGGGTAGCGC

CAGCGCTGAAGCCCTGATTGAGACCATCAAACAAGCCGGATACGGCGCGGAGCTCAGCCACCCAAAGGCT

AAACCGCTGGCAGAATCATCAATCCCGTCGGAAGCACTGACAGCGGCCACTCCTGAGCTTCCGGCAGCCC

ATGACGAGGATGACAGTCAACAGCTGCTGATCAACGGCATGAGCTGCGCCAGCTGCGTTTCCCGGGTACA

AAACGCGCTTGCCGCGGTGCCGGGAGTCTCACAGGCGCGGGTAAACCTGGCGGAACGCACGGCGCTGGTG

ATGGGCAGTGCGTCCGCCGCAGAATTAGTGCAGGCGGTGGAAAAAGCCGGCTACGGCGCGGAGGCGATCG

AAGACGATCTGGAACGCCGCGAACGGCAGCAGGAAACTGCCATCGCCACCATGAAACGTTTTCGCTGGCA

GGCGATTGTCGCCCTGCTGGTCGGCGTACCGGTTATGGTCTGGGGCATGATCGGCGATAACATGATGGTC

AGCGACGACAATCGATCGCTGTGGCTGGTCATCGGTCTCGTCACCCTCGCGGTGATGGTCTTCGCCGGCG

GTCACTTCTATCGCAGCGCCTGGAAAAGCCTGAAAAACGGTACCGCCACCATGGATACGCTGGTCGCCCT

GGGAACGGGCGTCGCCTGGCTATATTCAATGAGCGTCAACCTGTGGCCGCAGTGGTTTCCGATGGAAGCG

CGCCACCTGTATTACGAAGCCAGCGCGATGATTATCGGTCTGATTAACCTCGGCCATATGCTGGAGGCCC

GCGCGCGCCAGCGTTCCTCGAAGGCGCTGGAAAAACTGCTCGACCTGACGCCGCCTTCGGCACGCGTTGT

GACGCCTGAAGGGGAAGAAGATCTGCCGCTCACCGAAGTCCAGGCCGGCATGACCCTGCGCCTGACCACT

GGCGATCGCGTTCCGGTTGACGGCGTGATAAGCCAGGGCGAAGCCTGGTTTGATGAAGCGATGCTCACCG

GCGAGCCGGTGCCGCAGCAAAAAGGCGATGGCGACGCTATTCATGCCGGTACCGTGGTGCAGGATGGCAG

CGTACTGTTTACCGCCAGCGCCGTCGGCAGCCAAACCACGCTGGCACGGATTATTCGCATGGTGCGCCAG

GCGCAAAGTAGCAAACCGGAAATTGGCCAACTGGCAGATAAAATCTCTGCGGTATTTGTTCCGGCAGTGG

TAGCCATTGCGCTGATTAGCGCCGCTATCTGGTACTTCTTCGGCCCGGCGCCGCAAATCGTCTATACCCT

GGTCATTGCCACCACGGTACTGATTATCGCCTGTCCATGCGCATTGGGCCTGGCGACGCCGATGTCGATC

ATCTCCGGTGTCGGACGGGCGGCGGAATACGGCGTGCTGGTGCGTGACGCCGATGCTCTGCAGCGCGCCA

GTGAACTCGATACGCTGGTGTTCGATAAAACCGGTACCCTGACCGAAGGCAAGCCGCAGGTGGTGGCCGT

GAAAACCTTCGCCAGCTTTGATGAATCCACTGTCGTGCGGCTTGCCGCCGCGCTCGAGCAGGGTTCAAGC

CATCCGCTGGCGCGGGCGATCCTCGATAAAGCCGCTGACAGCACTTTACCGGAGGTAAGCGGCTTCCGTA

CCCTGCGCGGGCTCGGCGTCAGCGGTGAAGCGGAAGGCCATCAACTGCTGCTCGGCAACCAGGCGCTGCT

TAACGAGCAGCATATCGCTACCGCTGAACTGGAAAGCGAGATGACGGCACAGGCCTCGCGCGGCGCCACG

CCGGTGCTGCTGGCGATCGATGGTCAGGCCGCTGCGCTGTTCGCCATCCGCGACCCGTTACGGGAAGATA

GCATCGACGCGCTGGCGCGCCTGCATCGTCAGGGCTACCGGCTGGTGATGCTGACCGGGGATAACCCTAC

TACCGCCAATGCCATCGCCAAAGAAGCGGGCATTGATGAAGTGATAGCCGGCGTGCTGCCGGACGGCAAA

GCCGATGCCATTAAACGTCTGCAGAGCCAGGGGCACAAAGTGGCGATGGTCGGCGACGGTATTAACGACG

CCCCGGCGCTGGCGCAGGCGGATGTCGGTATCGCCATGGGCGGCGGCAGTGATGTCGCTATTGAAACTGC

GGCGATCACCCTGATGCGCCATAGCCTTAACGGCGTAGCCGATGCTTTAGCGATTTCGAAAGCGACGTTA

CGCAACATGAAACAGAACCTGCTGGGCGCGTTTGTTTATAACTCGCTGGGTATCCCGATTGCCGCCGGGA

TCCTCTGGCCGCTGACCGGTACCCTGCTGAACCCGGTGGTCGCCGGCGCTGCAATGGCGCTGTCGTCAAT

CACCGTGGTGAGTAACGCTAACCGCCTGCTGCGCTTTAAACCGAAAGATTAG

>gi_292897734_Erwinia amylovora ATCC 49946 chromosome_44

ATGTCGCAAGTCACCCTACTGACGCTGGACGGATTGTCCTGCGGGCACTGCGTAAAACGGGTAAAAGAGG

CGTTGGAGCAGCGCGCCGACGTGGAGCAGGCGGAAGTCAGTATCACCGAAGCACGCGTGAGCGGAAGCGC

CAGCGCCGGGGCGCTGATCGCCACGGTTGCCGGGGCGGGCTATCACGCCAGCCTGGCTGAAACGTTCGCG

CGCCCAAAGACTGACCCGCTGACAGACTCAGACATTCAGCCGGAAGCGCTGACAGCGGCCACCAGCGAGC

TTCCGGCGCAACAGAGTGAATGCCTCCAGTTACTGATTGATGGTATGAGCTGCGCCAGCTGCGTAGGCCG

CGTCCGGAATGCGTTGCACAATGTGCCCGGCGTCAGCCAGGCACGCGTCAACCTTGCAGAACGCAGCGCG

CTGGTGCTGGGCTGCGCGTTACCCACCGCATTAATCGAAGCGGTGAGCCGCGCCGGATATCACGCGGAAA

TCGTTGAAGACGAGGCGAAAAGGCGCGAAAAACAGCAGAAAAGCGGGCTGGCGGCGGTAAAACGCTTCCG

CTGGCAAGCAACGCTGGCGTTGGCACTGGGTGTTCCGCTGATGATTTGGGGGATGGCCGGTGACAATATG

ATGCTCACCGCAGCCAATCGCGACGGCTGGCTGGTGGCAGGAGTTATGACCCTGCTGGTGATGATTGTTG

CCGGCGGCCATTTCTACCGCAGCGCCTGGAAAAGCCTGTTAAATCGTAGTGCCACGATGGATACGCTGGT

AGCACTGGGTACTGCAGCCGCCTGGCTCTACTCCATCACGGTAAATCTCTGGCCTGAGCTGTTCCCGATG

GCGGCACGCCATCTCTATTATGAAGCCAGCGCGATGATTATCGGCCTGATCAATCTGGGACATATGCTTG

AGCAGCGCGCCCGTCAGCGCTCATCACGGGCGCTGGAAAGTCTGCTGGATCTCACCCCGCCCACCGCACG

CGTCGTAACCAAAGATGGCGTACAACTTCGCTCACTTAGCGAGGTACAGCCGGGGATGACGCTACGCGTG

ATGACCGGCGATCGTGTGCCGGTTGACGGCACTGTCGTGCAGGGAGAGGCCTGGCTGGATGAAGCCATGC

TCACCGGTGAAGCCATCCCGCAGCACAAAGCACCGGGCGATACCCTGCATGCCGGAACCCTGTTGCAGGA

CGGTAGCGTACTGTTGCGTGCCGGGGCCGTCGGCAAAAACACCACCCTGGCGCGCATCATCCAGCTGGTG

CGTCAGGCCCAGAGTAGCAAGCCGGAAATCGGCCAGCTGGCGGATAAAATTTCTGCGGTCTTTGTCCCGG

TAGTGGTGGCGGTCGCGCTACTGAGCGCTGCCGTTTGGTATCTGTTCGGCCCGGCGCCTCAACTGGTTTA

CACCCTGGTGGTAGCCACTACCGTGCTGATTATTGCCTGCCCCTGTGCGTTGGGCCTCGCCACACCGATG

TCGATTATTGCCGGCGTGGGGCGGGCCGCCGAATCCGGCGTGCTGGTGCGTGATGCCGATGCGCTGCAAC

GCGCAAGCACCCTGGACACGCTGGTTTTTGATAAAACCGGCACGCTGACCGAAGGCACGCCGCGCGTGGT

CGAGGTTATGCTGTTTAACCAGGCCGATCGCCAGACGGTGCTGCAGCAGGCGGCGGCTCTGGAACAGGGT

GCCAGCCATCCGCTGGCGCGCGCTATCGTCGCCCATGCCGGGCTGGCTACGCTGCCGGACGTTGAGCAAT

TTCGTACCATAGCGGGCAAAGGCGTGAGCGGCGTTTTCGACGGTAGCGCTCTGCTGCTGGGTAACGCGGC

GCTGATGGCGCAGCAGCAGATTGCCACCGATGCCGTCTCAATGCAGATGTCACGGCTGGCAGCAAAGGGC

GCCACACCGGTACTGCTGGCGGTGGATGGCCAGATCGTCGCCCTGTTTGCCATTCGCGATGCGCTGCGCA

AAGAGAGCGTCAGCGCGTTGCAACGCCTGCACCAGCAGGGTTACCGCCTGGTGATGCTGACCGGTGATAA

TGAAATAACCGCCCATGCCATCGCCCGCGAGGCAGGAATCGAGCGCGTGATTGCCGGGGTGTTGCCTGAG

GGCAAAGCCGCAGCTATCAGCGCCCTGCAACGGCAGGGGCACCGGGTAGCGATGATTGGCGATGGCATCA

ACGATGCCCCGGCGCTGGCCCAGGCCGATGTGGGGATTGCCCTCGGCGGCGGCAGCGATGTGGCGATTGA

AACGGCAGCGATGACGCTGATGCGCCCGGACCTCAATGTTCTGGTGGATGGTTTAGCCATCGCCAAAGCC

ACGCTGCGCAATATGAAACAGAATCTGCTGGGTGCTTTTATTTATAATGCCTGCGGCATTCCGCTTGCTG

CCGGCGTGCTCTATCCGCTGACCGGAACGCTGCTCAGCCCGGTCGTCGCGGCGGCGGCGATGGCGCTGTC

TTCAATAACCGTTGTGAGCAATGCCAATCGCCTGATCCGCTATCGCCCACCGACGTGA

>gi_283783779_Citrobacter rodentium ICC168 chromosome_45

ATGTCGCAAACCATCGACCTGACCCTGGACGGCCTGTCCTGCGGTCATTGCGTGAAACGCGTAAAAGAGA

GTCTGGAGCAGCGCCCGGACGTTGAGCAGGCGGAAGTGTCTATTACGCAAGCCCATGTCACCGGCACCGC

CAGCGCCGACGCGCTTATCGAAACCATTAAACAGGCCGGTTACGGCGCAGAGTTAAGCCACCCAAAGGCT

AAACCGCTGGCGGAGTCATCAATCCCGTCGGAAGCGCTGGCAGCGGTCCCTGATGAGCTTCCGGCAGCCG

CGGCTGAAGAAGATAGCCAACAGCTGCTGCTCAGCGGCATGAGCTGCGCCAGCTGTGTCACCCGGGTACA

AAACGCGCTGCAAAGCGTACCGGGCGTCACCCAGGCACGGGTAAACCTTGCGGAACGCACTGCGCTGGTG

ATGGGCAGCGCGTCCGCAACCGATTTAGTGCAGGCGGTGGAAAACGCGGGCTACGGCGCCGAGGCGATTG

AAGATGACGTTAAACGCCGCGAACGCCAGCAGGAAACGGCTATCGCCACCATGAAACGCTTCCGCTGGCA

GGCTATCGTCGCCCTGCTGCTCGGGGTGCCGGTGATGGTCTGGGGCATGATCGGCGACAACATGATGGTC

ACCGCAGACAACCGCAGCCTGTGGCTGGCGATTGGTCTGGCGACCCTCGCGGTGATGGTGTTCGCTGGCG

GCCATTTCTACCGCAGCGCGTGGAAAAGCCTGATGAACGGTACCGCTACTATGGATACGCTGGTGGCGCT

CGGTACCGGCGTAGCCTGGCTCTACTCGATGAGCGTCAACCTGTGGCCGCAGTGGTTCCCGATGGAAGCG

CGCCATCTCTATTACGAAGCCAGCGCGATGATTATCGGCCTGATCAACCTCGGACATATGCTGGAAGCGC

GCGCGCGTCAGCGTTCATCGAAGGCGCTGGAAAAATTACTCGATCTCACGCCACCGACGGCGCGGGTGGT

GACCGACAGCGGCGATAAGAGCGTACCGCTGGCAGAAGTCCAGCCGGGAATGCTGCTGCGCCTGACCACC

GGTGACCGCGTGCCGGTTGACGGTGAAATTACCCAGGGTGAAGCGTGGCTGGATGAAGCGATGCTCACCG

GCGAGCCGCTCCCGCAGCAGAAAGGCGCAGGCGACAGCGTGCATGCCGGGACGGTTGTCCAGGACGGCAG

CGTACTGTTTCGCGCCAGCGCGGTCGGCAGCCATACGACGCTGTCGCGCATTATCCGTATGGTGCGCCAG

GCGCAGAGCAGCAAGCCGCAGATCGGCCAACTGGCGGATAAAATCTCTGCGGTATTTGTCCCGGTCGTCG

TAGTCATTGCGCTGGTGAGCGCCGGGATCTGGTATTTCTTTGGGCCTGCGCCGCAAATTGTCTATACCCT

GGTTATCGCCACAACGGTGCTGATTATCGCCTGTCCGTGCGCGCTGGGACTGGCGACGCCGATGTCGATT

ATCTCCGGCGTAGGCCGCGCGGCGGAGTTCGGCGTGCTGGTACGTGACGCCGATGCCCTGCAGCGCGCCA

GTACGCTGGATACCGTGGTGTTCGACAAAACCGGGACGCTGACCGAAGGCAAACCGCAGGTCGTGGCGAT

CAAGACATTCGCCGGAGTAGATGAAGCGCAGGCGCTGCGTCTGGCGGCGGCCCTTGAGCAAGGTTCCAGC

CACCCGCTGGCGCGGGCGATACTCGACAAAGCGGGCGATATTACGCTGCCTCAGGTCAACGGCTTCCGTA

CCCTGCGCGGGCTGGGCGTCAGCGGCGAAGCCGAAGGCCACACGCTGCTGCTGGGTAATCAGGCGCTGTT

GAACGAGCAACAGGTTGCTACAACGGAAATGGAGGCAGAGATTGCCGCCCAGGCATCACAGGGCGCCACG

CCGGTGCTGCTGGCAATTGACGGCACCGCGGCGGCGTTGCTGGCGGTGCGCGATCCGCTGCGCAGTGACA

GCGTGGCGGCGCTCCAGCGCCTGCACCGCGCCGGATACCGGCTGGTTATGTTGACCGGCGATAACCCGAC

GACGGCAAACGCCATCGCTAAAGAGGCCGGCATCGATGAGGTGATCGCCGGGGTGCTGCCGGACGGTAAA

GCCGATGCCATTCAGCGTCTGCAAAGCCAGGGACGCCAGGTGGCGATGGTAGGTGACGGCATCAACGATG

CTCCGGCGCTGGCGCAGGCTGACGTCGGCATCGCAATGGGCGGCGGCAGCGACGTGGCGATTGAAACCGC

GGCGATCACCCTGATGCGCCACAGTCTGCTGGGCGTTGCAGACGCGCTATCCCTTTCCCGCGCGACGCTG

CGCAATATGAAACAGAACCTGCTCGGCGCGTTTATCTATAACAGCATCGGTATTCCGGTTGCCGCGGGGA

TCCTGTGGCCGTTCACCGGCACCCTGCTGAATCCGGTCGTTGCTGGCGCCGCGATGGCGCTTTCATCCAT

CACCGTGGTCAGCAACGCCAACCGGCTGCTGCGCTTTAAACCGAAAGAGTAA

>gi_325295716_Cronobacter turicensis z3032 chromosome_46

CTGAGGAGACAACGTATGTCAAACACCATAGAACTGGCCCTGGACGGGCTTTCCTGCGGACACTGCGTAA

AACGGGTGAAAGAGAGCCTGGAACAGCGCCCCGACGTTGAACGCGCTGACGTCACTATCGAACATGCCAG

CGTCACCACCACCGCGCCTGCGGACGCCCTGATTGAGACGGTGAAACAGGCCGGCTACGACGCGACGCTC

GCACACCCAAAGGCTAACCCGCTGACAGAGTCATCAACCCCGTCGGAAGCACTGACAGCGGCGCAACCTG

AGCTTCCGGCAGCGGATATCGCCGACGATGACGACAGCCGACAGCTGCTTATCAACGGCATGAGCTGCGC

CAGTTGCGTGAGCCGCGTTCAGAGCGCCCTGCAGGGGGTCGAGGGCGTCACGCAGGCACGGGTCAATCTC

GCGGAGCGCACGGCGCTGGTGATGGGCCACGCCTCCGCCGCTGACTTAATTGCAGCGGTTGAAAAAGCGG

GCTACGGCGCCGAAGCCATCGATGACGACGCCGAACGCCGCGAACGCCAGCAACAGACCGCGCAGGCGGC

CATGAAACGCTTTCGCTGGCAGGCGGCGCTCGCACTCGCCGTGGGTATTCCGGTAATGATCTGGGGCATG

TTCGGCGACAACATGATGGTCACCGACGCAAACCGTTCGCTCTGGCTGGTCATTGGCCTTATCACGCTCG

GCGTGATGATTTTCGCGGGCGGCCACTTCTACCAGAGCGCCTGGAAAAGCCTGCGCAACCGCAGCGCGAC

CATGGATACGCTGGTGGCGCTCGGCACCGGCGCGGCATGGCTCTACTCCATGAGCGTCAACCTCTGGCCG

CAACACTTCCCGATGGAAGCGCGCCACCTCTATTACGAAGCCAGCGCCATGATCATCGGCCTGATTAACC

TCGGCCATATGCTGGAGGCGCGCGCCCGCCAGCGTTCGTCGAAGGCGCTGGAACGCCTGCTGGATCTGAC

GCCGCCAGTCGCGCGCGTCGTCACGCCGGAAGGCGAGAAAAAATTACCGCTGGCGGAGGTGACGCCGGGC

ATGACGCTGCGCCTCACAACCGGCGATCGCGTGCCGGTGGATGGCGACATTACCGATGGCGAAGGCTGGT

TTGACGAAGCGATGCTAACCGGCGAACCGGTGCCGCAGCAAAAAGCGGCAGGTGACACCATTCACGCGGG

CACCGTCGTGCAGGACGGCAGCGTGCTGTTCCGCGCCGCCGCGGTGGGCAACCACACCACGCTGTCGCGC

ATTATCCGCATGGTGCGCCAGGCGCAGAGCAGCAAGCCGGAAATCGGCAAGCTGGCCGACCGCATCTCCG

CCGTGTTCGTCCCGGTCGTGGTGCTGATTGCGCTGTTCAGCGGCGCGGTCTGGTATTTCGCCGGCCCGGC

CCCGCAGATCGTTTATACGCTGGTGATTGTCACTACGGTGCTGATCATCGCCTGCCCCTGCGCGCTGGGG

CTCGCCACGCCGATGTCGATTATCTCAGGCGTCGGGCGCGCGGCGGAATTCGGCGTGCTGGTGCGTGACG

CCGACGCGCTGCAACGGGCAAGCGACGTAGACACGCTGGTGTTCGACAAAACCGGTACCCTGACCGAAGG

TAAACCGCAGGTGGCGGCCATCGCCACCACGGGCGACATCACCGAAGCGGACGCGCTGCGCCTCGCCGCC

GCGCTGGAGCAAGGCTCCAGCCACCCGCTGGCCCGCGCCATCATCGAGCGCGCCGAAGGGGCTTCGCTGC

CCGCCGTCAGTGGCTTTCGAACGCTGCGCGGTCTTGGCGTTAGCGGCGAGGCGCAGGGGAAAACGCTGCT

GCTCGGCAATCAGGCGTTGCTGGAAGAAAACGGCATCGCGACCGACGCGCTGCGCGATGAGGCGCAGCGT

GAGGCGAGTCTCGGCGCCACGCCGGTGCTGCTGGCGGTGAACGGTACGCTGGCGGCCCTCTTCGCCATTC

GCGATCCGCTGCGTGCAGACAGCGCCGACGCCCTGGCGCGTCTGCACCGCGAAGGCTACCGCCTGGTGAT

GCTGACTGGCGATAACGCCGTCACCGCAAACGCCATTGCCCGCGAGGCGGGTATTGACGAGGTGATCGCA

GGCGTGCTGCCGGATGGTAAAGCGCAGGCGATTCAGAAGCTCCAGCAGGCAGGCCGTAAAGTGGCGATGA

TTGGCGATGGCATTAACGACGCCCCGGCGCTGGCCCAGGCGGAAGTGGGGATCGCGATGGGCGGCGGCAG

CGATGTGGCCATTGAAACCGCCGCGATCACCCTGATGCGTCATAGCCTGACGGGGGTGGCCGACGCGATG

GCGATTTCCAAAGCGACGCTTGGCAATATGAAGCAGAACCTGCTGGGCGCTTTTATTTATAACGCGCTCG

GCATTCCCATCGCGGCAGGCGTCCTCTGGCCGCTGACCGGCACCCTGCTGAACCCGGTGGTCGCAGGCGC

CGCGATGGCGCTCTCGTCGATTACCGTCGTCAGTAACGCCAACCGGCTGCTGCGGTTTAACCCGAAAAAA

TAA

>gi_209693634_Aliivibrio salmonicida LFI1238 chromosome_47

ATGAGTCAGAAACTTACCCTACCCCTAGCGGGATTAAATTGTGGTCGTTGTATCGCAAAAGTGGAAAAAG

CACTTTCAGAGATCCCGAAGATATCTCATTTCTCGGTAACCAAAACAGAATTAACATTAACGACGACACT

CTCTTATGAAGAAATCGCAGAGAATATTACGCAATTAGGCTACTCCATTCCTGAACCCGAACTCATCACT

TTGTACCTAGCAGGATTAAAGTGTGGTAAATGCATAAATAAAACCACAGACACGTTAAGTACCTTGTCGC

ATACCTCTATTGAGTCTATTTCAAAAGATAAAATCACGCTAAACACCGTGCACTCTCAAGAAAGCATCAT

TGAATTAATTGAATCCATCGGTTTTCAAGCAAGCTCTGCCCCCTTCATTCCAGATAGTCCCTTACCTAAA

GAAGAGGTCATTACTCTGAATAAAGAAACACCGACAGATCAAAGCAAAACTGAAAAACAAAAAATAGAAA

AAACAACACAATTAATTTTATCGGGAATGACTTGCGCTAGCTGTGTCGCCTCTGTAGAAAAAATAATTTC

AGCCGTTGCTAATGTTCAATCTGTCAGTGTTAATTTAGCGGAACGTACCGCTTTAATTTATGGTGATATC

AATGCACCTGAACTGATCAAAGCAATAGAAAAAGGCGGTTATGGTGCAGAGATCAGTTTAAGCGAAGAAC

AACGCCGTAAAAGCCAAAAAGAACAATACCTAAAAACCACCGTAGCACATAAAAGAAATACCATTATGTC

GCTCGCAATAGGCGCGCCATTGATGCTTTGGGGGGTATTTGGTGGCAACATGCAAATAACAACCGTTAAC

GACCAACTTGCTTGGGGACTCATTGGGCTTATTTGTTTAGGTTTATTACTTACCGCTGGGAAACATTTTT

TTGTTAATGCCTGGAAAGCATTTACTCATCACAGAGCGACAATGGATACGTTAGTGTCTCTTGGTACTGG

TTCAGCTTGGCTCTACTCTATGGCACTGGTGATCTATCCTGCATTTTTCCCAGAGCAAGCTCGTCATGTT

TATTTTGAAGCCACCGCAATGATCTTAGGATTAATTACGCTCGGAAATCTAATCGAAACCAAAGCTCGTA

ATCGCACATCAAATGCCATCGAAAAGCTGCTCAATCTTCAGCCTCAAACCGCTACGGTTATCCAAGATGG

AAAAGAAGTAGAGTTGCCAGTGGCACTCGTAACGAAAGGGATGATCATTAAAATTAAACCGGGAGAGAAA

ATCCCAGTTGATGGTTCGATCATCAGCGGTGAAAGCTATTTAGATGAAGCAATGCTTACTGGAGAGCCTC

TTCCTTGTTTAAAAATAAAAGACGATGTTGTGCATGCAGGAACCATCAATCAACAGGGTAGCTTTACTTT

CACAGCAACGAATGTCGGTGAACATACTATGCTTGCTCGGATCATTGCCATGGTACGTAACGCGCAAAGT

AGCAAACCAAAACTGGCTAAGCTCGCGGATAAAATCTCATCCGTATTTGTCCCAAGCGTTATGATCATCG

CCATTTTTTCGGCTTTACTTTGGTATAACTTTGGTCCAGAACCAACATCGAGCTACATGCTTGTTGTTGC

TACAACGGTACTCATCATTGCCTGCCCTTGTGCTTTAGGTCTTGCAACACCAATGTCAGTGACTGTTGGT

GTCGGTAAAGCCGCTGAGTTTGGCGCATTAATAAAAGACGCTGACGTTTTACAAACAGCAAGTCATATCG

ATACCGTGGTGGTTGATAAAACGGGCACATTAACAGAAGGAAAACCCACGGTCAGTAAAGTGACAAGCTT

TGCGGCTTACAATGAAAAAGACATCATTTCATTCGCAGCAGCAATCGAAAGTCACTCTGAACACCCACTC

GCTCATGCAGTAATGACATATCTCAATCAGCATTCATGGGATTTAGTCACCAGTACACAGTTTAATGCTC

AATTAGGCCTAGGAGCTTCTGCAAACATTGCTGGCGAGACTGTGTTCATAGGAAATACCTCTTATTTATT

AAACAATGAGATTGCCACTCCTGACGTTGAATTATCTCCTGCATCAACGCCTCTCTTTGTGGCGATAAAT

AAAGAATGTATTGGTGTTATTGAAGTAAGTGATGCAATTCGACCCGATTCAAAACTTGCTGTAGCCCAAT

TTACTCAACTCGGTATTAATGTCGTAATGCTCACAGGTGATCGCAAAGAAACTGCTAATTATGTGGCTCA

ACAACTCGGAATAACTCATGTTGTTTCGGGGGTATTACCCGATGGGAAAGCTCAGACAATAAAAGACTTA

CAAGCTCAAGGAAAGAAAGTAGCTATGATTGGGGATGGCATTAATGATGCCCCAGCGTTAGCTCAGGCAG

AAGTAGGGATTGCGATGGGCAATGGCAGTGATGTCGCGATTGAAAGCGCTCAATTGACCTTAATACGACA

CTCACTGCTTACCGCCGTTAATGCGATTGAGCTTTCAAGAGCAACCATTACAAATATGAAACAGAACTTA

TTCGGTGCCTTTATTTATAATGCGATAGGCATTCCTATTGCTGCCGGTATTCTTTTTTCATTTACTGGTA

TGTTACTCAGTCCTGTTGTAGCAGGAGCAGCGATGGCACTTTCTTCTATTACCGTTGTATCCAATGCAAA

TAGATTACGACTTTTTAAATCTCAGTTTTCAAACAAACATACTGACTAG

>gi_400756305_Geobacter sulfurreducens PCA chromosome_48

ATGCGTAAGGTCTTGTTTCCCATCACCGGCATGAGCTGCGCCGGGTGCGCGGCCCGGATCGAGAAGGAGC

TGGGCGGCGTGGCGGGCATTGCCTCGGCCGTGGTCAACTTCGCCACCGCCGAGTTGTCCGTGGAGTTCGA

CGAACGGGTGATCGACGAGGATGCCGTGGTGGCGCGGGTGGAAGCCCTGGGCTACGGAGTGGTGCGCACG

GCGGCCGGGGAGCTGCGTTTCGGGGTGCGGGGGCTGCACTGCGCCTCCTGCGTGGCGAACCTGGAGAAAA

AGCTCCTGGCCGACCCGGCCGTGTCCGCCGCCGTGGTGAACCTGGCCCAGGAGGAGGCCCTGGTCCGGTT

CGATCCCTCCCGCCTGGGCAAGGCGGACATCTTCGCCCTGGTGGTGGCCGCGGGGTACACCCCCGTGGAG

CCCGAAGCGGAAGGGGGGGAGGCCGCCGCGGAGTTGAAGGGCCAGCGCAACTGGTTCATTGCCAGTCTTC

TGCTCTCGCTCCCCATCATGGCCACCATGACCCTCCACGACAACCGGGCCGTGGGCTGGATGAACCTGGT

CCTGGCCAGCGCGGTCCAGTTTTCGGCCGGCCTCACCTTCTACCGGGGGAGCTGGTTCGCCCTGAAGAAC

CGCAGCGCCAACATGGACGTGCTGGTTGCCCTGGGCACCTCGGCCGCCTATTTCTACTCCCTCTTCGCTT

TTTTCGGGGCCTTCGGCGAGCATGGCGGCCACGTGTTCTTTGAGACCTCGGCCATGCTCATCGCCTTCAT

CCGCCTGGGCAAGTACCTGGAAGCCCGGGCCCGGGGCAAGGCGGGGGAAGCCCTGAAGAAGCTCCTGCGG

CTCCAGGCGGACAAGGCGCGGCTGGTGACCGGCGACCAGGAGCGGGAAGTCCCCGCGTCGGCGGTGCGGG

TGGGCGATCTGGTCCGGGTCCGGCCGGGCGAGACGATCCCCGTGGACGGGGAGGTGGTGGAGGGAACCTC

GTCGGTGGACGAGTCCATGGTGACCGGCGAGTCGATCCCGGCGGACAAGGGGCCGGGGGCCGCGGTCACC

GGGGCCACGGTGAACCGCAGCGGCGTGCTCCTGGTGCGGGCCACCCGCATCGGCGAGGAAACGCTTCTCT

CCCAGATCGTGCGGATGGTGCGGGAGGCCCAGGCGGACAAGGCGCCGATCCAGCGGTTCGCCGACCGGGT

GTCCGGGGTCTTCGTGCCGGTGGTGATCGCCCTGTCGGCCCTCACGTTCGCCCTCTGGTTCTGGGGGCTC

CACCAGGAGTTCCTCTTTGCCTTCAAGCTGGCCATCTCCGTGGTGGTCATCGCCTGCCCCTGCGCCATGG

GGCTCGCCACCCCCACCGCCATCATGGTGGGGAGCGGGGTGGGGCTGTCGCGGGGGATTCTGGTCAAGCG

GGGCTCGGTGCTGGAGAACATCTCCCGGGTCCAGGCGATCCTGCTGGACAAGACCGGCACCCTCACCCGA

GGCGAACCCTCCCTCACCGACCTGGTGCCGGCGCCGGGGGTGACGGAGGAGCGGCTGCTGGCGGTGCTGG

CCGCGGCCGAATCCCGCTCGAACCATCCCCTGGCCCAGGCGGCGGTAAGCGGCGCCGCCGGACGGGGCGT

GGTGCCGGCGCCGGTGGACGGCTACCGGGAGACCGAAGGGGGAGGCGTGGCCTGCGTCCTGGACGGTGAA

CCGGTAACGGCCGGCAGCGCCCGGTTCCTGGTCGGGGCGGGCATCGACACTTCGCCCCTGGAGCAGGCCG

CATCCCGGTTGGCCGGGGAGGGGAAATCGCTCATCCTCGTGGCCGAAGCGGGACGCCTGCTGGGGGTGGC

GGCCCTGGCCGACCGGCTCAAGGAGAGCTCGCCCCGGGCCGTGGCCGAGCTGAAGCGAATGGGGATAGCC

ACCTGCATGATCACCGGCGACCACCGGGAGGTGGCGGCCGCCGTGGCCCGGGAGGCGGGGGTTGACAGCT

TCGAGGCCGAAGTCCTGCCGGGCCGCAAGGAGGAAATCGTGCGGGAGTACCAGGCAAAGGGGTACTTCAC

TGCCATGGTGGGCGACGGGATCAACGATGCCCCGGCCCTGGCCCGGGCCGACGTGGGGATCGCCATCGGC

GGCGGCACCGACGTGGCCAAGGAGACCGGCGACGTGATCCTGGTGCGGGACGACCTCATGGATGCCGTGC

GCGCCATCCGCCTGGGGCGGGCGACCCTGGCCAAGGTGAAGCAGAATCTCTTCTGGGCCCTGTTCTACAA

CATCCTCGGCATCCCCGTGGCCGCCGGGGTCCTCTACTATCCGCTGGGGATCACGCTCCGGCCCGAATTC

GCCGGGCTTGCCATGGCCTTTTCCTCGGTGTCGGTGGTGACCAACTCGATCCTGCTCCGCCGGGCGGGAA

AGAAGCTGGGCTAA

>gi_224367124_Desulfobacterium autotrophicum HRM2 chromosome_49

ATGGTTTGTGTTAAGAATATTCACGTATCTGGAATGAGCTGCGGACATTGCGTCAACAGCGTAACCCAGA

CCCTGGAAGCCATACAGGGCGTAAGTTCGGCAAGGGTCTCCCTTGAAGATTCAACGGCTGAAGTGACCTT

TGACCAGGAGGCCGTTGACCTTGAAACTTTAAAGGCCGCCATCAGGGAAAAGGGGTTTGGCACTGAAGCA

CCCTCGCCAATCAATAACAAGCCGTTTACCATTATTGCCCCGAAAAACGTTGAACCGCCCTTTGCCCGGC

AAAGACAGCCGGTTGAAACAAGCTTTAAAATTGAGGGTATGTCCTGTGCCAACTGTTCCAACACCATAGA

AAAAACCCTTAAAAAATCCAAGGGCATAGAAAAGGTCTCGATCAATTTCTCCATGGAAAAGGGATTTGTA

ACCTATGACCCAAGCCTGGTGGATGAGCAGGGCATCTTTGAAATCGTCCAGCGGGCAGGTTATACTGCCT

TCAGCCAGGAAGAAAAAAGAACAGAGGATTCTCCCATTGCCCAGAAAGAGCGATTCCGGTTTATTTTTGC

AGCAAGCCTGACCATCCCCCTGGTCTTTCTCATGTACACCATGCCCTTTGGCCATGCCAACACCAACTAT

TCCATGGGCCTGCTTGCCACCCTGGTAATGGTTGTGTCTGGCCGCACCTTTTACGAGGGTGCCTTTCATT

CCCTGAAAAACCGTTCGGCAAACATGGATGTCCTCATCGCCCTTGGCATCTCTGCTGCCTATTTTTACAG

CCTTTTTTCTTTGCTCGTCCTTGACCCCGCGGCCCATACCTTTTTTGACAGTGCAGCCATGCTCATCACC

TTTATCCTGCTGGGCAAAATGATCGAGGCCAAGGCCAAGGGTCAAACAGGCGAGGCCCTTGAAAAACTCA

TCTCCCTTGGGGCTGACAGGGCAAGGATCCTAGTCAACGGCAGCGAACACATGGTCAAGGCGTCCGAGAT

AAAAAAAGGCGATTTTTTTTCCGTCCGGCCCGGAGAAACCATTCCGGTTGACGGTGAAATCGTCGAGGGA

ACCACCACCATTGACGAATCCATGATCACTGGCGAAGCCATTCCTGCGGACAAGATCAAGGGCGACCCGG

TAACCGGGGCCACCATCAACCAGACAGGTGCCATCACAGCCAGGGCCGTTCGAGTGGGCAAAGAAACCAT

GCTCGCAAGGATTGTCAAGATGGTTGAGGAGGCCCAGGCAGACAAGGCCCCGATCCAGAGACTTGCCGAC

ACCGTGTCCAACTACTTTGTCCCGGTGGTGGTCATTGCCGCCATCCTCACCTTTATTGTCTGGAACTTTG

TTGTTGAATATCCGCCGCCCTTGGGTATCACCCGGTTTCTCTTTGCCTTTCAGCTGATGATCGCGGTACT

GGTTGTGGCCTGCCCCTGCGCCCTTGGCCTTGCCACCCCCACGGCCATAATGGTGGCAAGCGGTGTGGGT

CTTAAAAGGGGAATCCTTTTTAAACGGGCCTCAGTGCTTGAAAATATCTCCCAGCTGGATGTGATTCTGT

TTGACAAAACCGGCACCCTCACAAAGGGCAGCCCCAGGGTCTCGGCCATTTACCCCATGGACCATGGATC

TGAACACCAGGTGCTTAAAATAGCGGCATCCCTGGGGGCCGCATCAATCCATCCCCTGGCCCACGCAATT

GTTAAAAAGGCCGAACAGGCGGGTGTCTCCATGGAAAAAATCTCAAATCAACGGGAAATCCCCGGACACG

GCTCAACGGGCGAACTTGACGGCAGACCGGTCATGCTTGGCAACCTCAAACTGCAACCCCAGGGAAGCTT

TCTTTCAGAAGAGGCCGCAGCAGCCGGTCAGCAGATTTCAGATACCGGAGATTCTTCGGTGTATGTATGG

CAGGACAATACCATGAAGGGCATCATCTCCCTGGCTGATGAAATCAAGGCCGATTCAAAAGAGGCCATGG

CAAGACTCCACGCCCTTGGCATCAAAACAGCCCTTGTCTCTGGGGACAATAAAAAGGCGGCAAAGCGTGT

GGCTCAGATCGTCGGAATTGATGAAGTCGAAGCCGAGGTGCTACCCGGTGACAAGAGTCGGATCGTCAAA

AAATGGCAGGAACGGGGTTACCGGGTCGCCATGGCAGGAGACGGCATCAACGATGCCCCGGCCCTTGCCC

AGGCAGACATCGGCATTGCCGTGGGTTCAGGAACGGACATTGCCAAGGAGACCGGGGACGTAATCTTGGT

CAACAACTCCCTTTTAGACGTTGAACGGGCCATTGGGCTCGGACGAAAGACCCTCAAGACCATACGCATC

AATTTCTTCTGGGCCTTTTTCTACAACCTGCTCATGATACCGGTTGCAGCCGGCCTTGCCTACCCTGCCC

TGGGGCTGGTTCTCAAACCTGAATGGGCCTGCATTGCCATGTGGCTGTCTTCACTTACGGTTGTGGGAAA

TTCCCTGCTCTTGAAAAGATACCGGTTTTAA

>gi_426402010_Bdellovibrio bacteriovorus str. Tiberius chromosome_50

ATGGAAACTCAAGATAACAGCACCGAGTTGCAACTCATTGGCATGTCCTGCGTGAATTGCGCGGGAAAAA

TTGAAAAGACCTTAAACGGCCTGCCGGGCGTGACTGCGACGGTGAATTTTGCGACGGAAAAAGCCCAGGT

GAACCTGGGGGGAAGTCCCCTGACGGTCGAAAACCTGATCAAGACCATTCAGGATTTGGGCTATCAGGCC

TTTGAGGTAAACTCTGAAGTCGACGCACAAAAGATAAAAGAAGAAGCGGCTGCTGAATACCGCAAAGAAC

TGCGTCTTTTTATTATCTCAGCGATTCTGACAGCTCCCTTCCTGGTTGAAATGGCCGTGATGCTGACTGG

GGCGGGGCATGAAATGATTCCGCGCTGGGTCCAGTGGATTTTGGCGACTCCGGTTCAGTTCTGGATCGGG

TGGAGATTCTATCGCGGGTCTTATTATGCTCTTAAATCCGGCAGCGCCAATATGGATGTGCTGGTGGCTT

TGGGAACGACGATGGCTTACGGTTTAAGTGCGGTGATGACGGCCATGAACTGGCATCATCATCACGTGTA

TTTCGAGGCCAGTACTGCGGTCATTACTTTGATTCTGCTGGGTAAGTTGATGGAAAGCCGGGCCAAGGGC

AAAACTTCCGAGGCGGTGGAAGGTCTGCTTCGCCTGCAGCCGAAAAGAGCTTTTGTAGAAAGAAACGGTG

AAGTCATCTCTTTGGATATCAGTGAACTTGTGTCCGGGGATGTTGTGGTGGTGAAAAACGGTGAGTCCAT

TCCGGTGGATGGCCTGGTGGTGCAGGGGACTTCCAGCGTGGATGAGTCCATGCTGACCGGCGAAAGCATG

CCGGTGCAAAAGGGTGTTGACGATCGCGTCTATGCTGCGACCTTAAATCAAGATGGCAGTTTGCGTATCA

AGGCGACGGGGGTGGGCAGCAAAACACAGTTGGCGCAAATCATTAAAATCGTAACGACGGCTCAAGGGTC

CAAAGCACCGATTCAGCGTTTGGCTGATAAAATCTCGGCGGTCTTTGTTCCAATTGTGGTCGGGATCAGT

GTTTTGACTTTCTTCGGCACGTGGCTGTGGACAGGGGATTTGACTCTGGCTTTCATCTCATCAGTGTCGG

TGCTGGTCATTGCTTGTCCGTGTGCTTTGGGGCTGGCAACGCCCACCGCGGTGGTTGTGGGGATTGGCAA

GGGAGCGCAAGCAGGAGTTTTATTCCGGGATGCCAAGGCTTTGGAGCTTGCTGAAAAAATTGATGTTCTG

GTTTTGGATAAAACCGGCACCATCACCGAAGGAAAGCCCGTGGTAACTGAAGTTCATGTGACCTCGGGCC

AGGATAAGAAAGAGGTTCTGACGTTTGCGGCAAGTCTTGAGGCAGGCTCTTCCCATCCGTTGGCGCATGC

AATTTTGGAAGAAGCCAAAAAAGAAGGTTTGCCTTTGGCTGCGGTTGAAAGTTTCAAGAGTGTGATGGGG

CAAGGGGTGGAAGGTCGTATTCAGGGCCGCTCACTGAAGCTGGGTAAAAGCGCCTGGGTGGCTCCGGATT

TTTCTTTGAATCAGGAACTGTTGAGGGACCTTGAATCCAAAGGTCAGACACTGATGGTGCTGGCTGATGA

GACTCGCGTGCTGGGTTATATTGCGGTGGCCGATCGTATTCGTGAAAGCTCCAAAGAAGCCATTGCCGAA

ATTCAAAAGCGCGGCGTGAAAGTAATGATGCTGACCGGGGATAACGAAGGCACGGCCAAAGAGATATCCC

GTCAGGCTGGAATCACAGAATACAAACACAGTGTGAAGCCAGCGGATAAAGCCAATGTCATCGTCACCTT

GAAGCGCAAAAAACTGCGTGTGGCGATGGTGGGTGACGGTATCAACGATGCCCCGGCATTGGCGATGGCG

GATGTCAGTTTCTCAATGTCCTCGGGAACAGATATCGCCATTGAAACGGCGGATGTGACCTTGATGAAAA

ATGATCTGAAGTCCGTGGCCCAGGCCATTGAACTGTCGCATCTGACGCTGAAAAAAATCCGCCAGAATCT

GTTCTTTGCATTCATCTATAACGTCCTGGGAATTCCGCTGGCAGCACTGGGAATGCTAAACCCTGTCATT

GCGGGTGCCGCCATGGCGATGAGTTCAGTTTCAGTCGTCAGCAACTCGCTGCTATTGAAAAGAAAGAAAA

TCTAA

>gi_383452024_Corallococcus coralloides DSM 2259 chromosome_51

ATGAGCACCATGAAGCCCACTGACGAACCCCAGGGCCCCTCCCAGGAAACGCAAGCCGAAGCTCCCTCGC

TCACGCGCCGCGGTCTGCTCGCGCGCACGGGCGCGACGCTGGCGACGGGCGCCCTGCTGATGCACGGCCG

CGCCGCCCAGGCGCAATCCAGCGTGCCCGGCGCGAAGGCCCCGCGTGAAGGGTCCGCGGCGGACACGGGA

GGCAAGGTCACCCGGCAATCGCATCTGCCTCCTGGGAAGGAGGGCCGCGACTACCGGCCTGTCGTCGTGC

CCAACGGCGCGAAGCTGCCGTGGAAGGACGTGGGCGGCGTGAAGGTGTTCCACCTGGTGGCCCAGGAGGT

GGAGCACGAGTTCGCGCCCGGCCTCAAGGCCACGTGCTGGGGTTACAACGGCCACGTGCACGGGCCCACC

ATCGAGGTGGTGGAGGGCGACCGCGTGCGCTTCTACGTCACGAACCGGCTGCCTGCGTCCACCACGGTGC

ACTGGCACGGCGTCCTTCTCCCCAGCGGCATGGACGGCGTGGGCGGGCTGAGCCAGAAGGCCATCGCCCC

GGGAGAGACCTACCGCTACGAGTTCACGGTGCGTCAGTCGGGGACGTGCATGTATCACTCGCACCACGAC

GAGATGACGCAGATGGCGCTGGGCATGGTGGGCCTGTTCATCATCCACCCGCGCAGGCCGGTGGGTCCGC

GCATCGACCGGGACTTCGCCATCATGCTGCACGAGTGGCGCATCGACCCGGGGACGATGCGTCCGGACCC

CAACGAGATGACGGACTTCAACATCCTGACGATGAACGCGAAGGCGTTCCCGGGCACGGAGCCGCTGGTG

GTGCGCAAGGGCGAGCGGGTGCGGATCCGCTTCGGCAACCTGAGCGCAATGGACCACCACCCCATCCACC

TGCACGGCTTCCAGTTCCGCATCACGGAGACGGACGGAGGCCGCATCCAGGAGAGCGCGCAGTTGCCGGA

GACGACGGTGCTGGTGCCTACCGGGAGCACGCGCACCATCGAGTTCGTGGCGGACGAACCCGGTGACTGG

GCCATGCACTGCCACATGACCCACCACGTGATGAACCAGATGGGCCACGACATTCCGAACATGATCGGCG

TGAAGCCCGGAGGTCTGGACGCGAAGGTGCGGCCCCTGCTGCCCGGCTACATGACCATGGGGCAGATGGG

CATGGGAGACATGGCGGGCATGAGCCACATGCCCATGCCCGCGAACTCCATCCCCATGGTGGGCGGCAAG

GGCCCCTACGACGAGATCACCATGGGAGGCATGTTCACGATCCTCAAGGTGAGAGATCGCCTGGAGGGAT

ACGCCGATCCCGGCTGGTACACGCCGCCGCAGGGGACGCTGGCCCGGCTCGCCCAGTTGGATGAGCTGCG

CCGGGACGGCATCGACGTCTAG

>gi_94986445_Lawsonia intracellularis PHE/MN1-00 chromosome_52

ATGTTGCATAGCAACCTAATACTGTTGGAGCTAACTGTGTCTAAACAACAGACATTGAACTTTATTGTCA

AAGGCATGAGTTGTACCTCATGTTCTTCAAGGCTTGAGCGTACATTAGGTAATATTCCTGAAATACAATC

TGTTACTGTAAGCCTTAATAATTCAATGGCAACAGTTATTCCAATCCCTGAAGCTAATATAAAAAAGCTT

ATTCAAGAAGTCATTTCTGAAACAGAAAAAACTGGTTTTATTGCTAACTACTTAGCAGAAGATCTTGCTG

AACAAAAAACATGGATTCTTCAACAAGAATCCATCTCTCATTACTTAGAGGAACTCAAACAACGCCTTAT

TATAGAATTCTTTTTTACTATACTAGTTCTCTTCATTTCAATGGGACCAATGTGGGGAGTTCCTGTCCCT

GTATTTGTTAATCCTAAAACATATCCTCTAAATAATGCTATTGCACAACTTTTATTAACTATTCCTGTAA

TTTGGTCAGGTAGATATTTTTACCTTATAGGATTCTCAAGGCTCTGGAAAATGAGTCCTAATATGGATAC

ACTTGTTGCTCTTAGTACTAGCGCTGCTATAATCTATAGTATATGGTCCACAATAGAAATAAGCTTTGGA

AACTATTATACTGAACCTAGTCTCTATTATGAAACAGCAGTTGTCCTTATTACACTCATTTCTTTAGGAA

AATATTTTGAAATGCTCTCACGCTTCAGGATGTCAGATGCAATAAGCCGATTAATGAACTTAGTTCCAGA

AACAGCATTACGCCTTTCTTCTCTATCTGATCAAGAAGAATCTTTTGAGGAAGTGCCTCTTAGCAATATA

AAAGTAGGAGATATTGTTCAAGTGCGTCCTGGCTCAAAAGTTCCTGTAGATGGGAAAATATTAAAAGGCT

CTTCAAGCATTAATACTTCAATGCTTACAGGTGAATCTATGCCTGTTTTTGTAACTGTAGGAGACAAAGT

TATTGGTGGTACTATAAACCTTTCAGGCATGTTTCTTATGCATGCAGAAAAAGTTGGACTTAATACAGCG

TTAGCACACATTATACGCCTAGTTCGTGAAGCACAAGGTTCTAAAGCACCAATATCTCGTATAACAGACC

AAATAAGTCTTATCTTTGTACCAGTAGTTATAGCTATAGCTTTAGTAAGTGGACTTACATGGTTGTTACT

TGGCGATACATCTATTGCAGATTCTATCCGTATTGTTATGACTGTACTTGTAATATCTTGCCCTTGTGCT

TTAGGATTAGCAACTCCTATGTCAATTATGATTGCCACAGGAAGAGGGGCACAATTAGGACTTCTTATTA

AATCTGGTGCAGCACTTGAATATGCAGCAGATCTTGATACTATTGTTTTTGATAAAACAGGAACACTTAC

AGAAGGAAAACCACACCTTATTCATACAGTTCCCCTAAAAACAATGCCAGATAATGAACTTCTTCGTATA

ACAGCCTCATTAGAAGCTGTATCAGAACATCCTTTAGCAGATGCTATTGTGAGAGCTGCACATAAACATG

GCCTTGTACTTTACCCTGTTATAAACTTTATGTCCTATCCTGGTCGAGGAGTACGTGGAGAACTTCAAGT

GAAGACAGAATCTATAACTGTTTTAGTTGGTAACAAAACATTCCTTGAAGAACATCATATTTCTTTAGAC

AATAAAGTAGGAATAACAACCCTTGAAGAATTTGCAGGACAAAGCTTTATATCTTTACTTGTAGCTATAA

ATGGACAGTTTTCAGGGATAATTGCAGTAACTGATCCTATTCGAGAAGAAAGTAATAGTGTAACAGAACA
ATTACAGCATATGGGAATCCGTACAATTATGCTTACAGGTGATAGTAAACAAACAGCTCAAATAGTTGCT
AATCATTTACACCTGAATGAAGTTATTGCTGATGTAATGCCAGAAGGGAAAGCTTATGCTATCCAAGAAC
TTAAAAATAAAGGATTTCGTGTTGGAATGGTTGGTGATGGTATTAATGATGCACCAGCTCTTGCTAAGGC
TGATGTAGGAATAGCCATAGGAAGTGGAATAGATATAGCAATTGAAACAAGTGATATTGTTCTTCTTAAT
TCTTTATCAACAAATCATCCACTATCTGGCTTAGTTTCGGCACTCTTATTGAGCCGTGCTACTCTTAGGA
ATATTCGACAAAATCTTTTTTGGGCATTTGGTTATAATATAATATGTATACCAATCGCTGCAGGTATATT
AAAACTCTTTGGTGGGCCTTTACTTTCACCTATGCTAGCTGGTCTTGCAATGGCACTTTCATCTATATCT
GTCGTCCTTAATGCATTACGACTGAAAAAATTTACACCATCTTAA
>gi_153002879_Anaeromyxobacter sp. Fw109-5 chromosome_53
ATGAGCGAGGCTCCCGGAGCGCACGAGTCGAACCCGCGCGGCGGGATCACGCGGCGGCGCTTCGTCCAGT
GCGTCGCCCAGGCCGGGGCCGCGGTGAGCCTCGGCCTCGCGCCGGGCCGCGAGGCGCGGGCCCGGCCCGC
GGGAGCCACGGGAGCGCCCCCGGTGCTCGACGCGAGCGCCGGTCCCATCGACCTCCGCGTCGAGCGCACG
CGCGTCGAGATCGGCGGACGGCGTGGCGAGGCGGTCACCCTGAACGGCGTGCTCCCCGCGCCGCTCCTGC
GCTTCCAGGAGGGGCGCGAGGCGGTGATCCGCGTCCTCAACACGCTCGACGAGGACACCTCGATCCACTG
GCACGGCGTCCTCGTCCCGAACGCGATGGACGGCGTTCCCCAGGTGAACTTCCCCGGGATCCGGCCGGGC
GAGACGTTCGTGTACCGCTTTCCCGTTCGGCAGTACGGCACGTACTGGTATCACGCTCACTCCGCGTTCC
AGGAGCAGCTCGGCCACTACGGGCCGCTCGTCATCGAGCCGCGGAGGGGATCGCCGTTCACCTGGGATCG
CGAGCACGTGGTGCTGCTCTCGGACTGGACGTTCGAGGACCCGCACCGGATCTTCGCGAAGCTCAAGAAG
CAGCCCGACTACTACAACTTCCAGAAGCGCACGGTCGGCGACTTCTTCCGCGACCTCGGCAGGAAGGGCC
TCGGGGCGACCCTCCGCGATCGGGCCGAGTGGGGCCGCATGAACATGAACCCGACGGACCTCGCGGACGT
CACCGGGGCGACGTACACGTACCTCGTGAACGGGATCGCTCCGGACGGCGGCTGGACCGCCTCGTTCCGG
CCCGGCGAGCGCGTCCTGCTCCGGTTCATCAACGCCTCGGCGAGCACGTTCTTCGACGTCCGGGTCCCCG
GCCTCGCGATGAAGGTCGTCGAGGTGAGCGGCCAGTGGGTCGAGCCGGTCGACACCGAGGAGATCCGGAT
CGCCATCGCCGAGACGTACGCGGTGCTGGTGGAGCCGAGCGAGGACCGCGCCTACGCGATCTTCGCCGAG
GCGCTCGACCGGAGCGGGTTCGCCCAGGCGACGCTGGCGCCGCGCGCCGGGTTGCGCCCCCCCACGCCGG
AGCGCCGGAGGCGCGCGCTGCTGACCATGGCCGACATGGGCATGGCCCACGGCGCGATGGGCGGCATGGA

GGGGATGCAGGAGGGCGGCCGCGGACACGGGGGAGAGCGAGCAGGTGGCGCCGCGCACGCGGGGCACGC
C

GCCGCGGCGTCCGCGGGGCCGAGGCCGCCGGGCGCGCTCCCGGAGCCGAGGCCGCACGGGCCCGATTCCC

ACGGCCCCGGCAACGCCGCGATCCCGATGATGACTCGCAGCCGGCTCGAGGAGCCGGGCATCGGGCTCGG

CGACGACGGCCGCGCCGTCCTCACCTACGCGGCGCTCCGCGCGCGCGAGCCACGACCCGACTTCAGGGCG

CCCGACCGGGAGATCGAGCTCCACCTCACCGGCAACATGGAGCGGTTCATGTGGTCGATCGACGGCGTGA

AGTACTCGGACGCCGAGCCGATCCGGGTGCGCCCCGGCGAGCGCATCCGGCTCACGCTCGTGAACGACAC

GATGATGAACCACCCCATGCACCTGCACGGGATGTGGATGGAGCTCGAGAATGGGCACGGCGAGCGGATC

CCGCGCGTACACACCGTGAACGTCAAGCCAGCGGAGCGCATGTCGCTGTTGTTCACGGCCGACGCCCCCG

GCCCGTGGGCGTTCCACTGCCACGTCCTCTACCACATGGAGGCGGGCATGTTCCGCGTGGTCCAGGTCTC

CGAGCGCGGAGCGACGGAGCCCGCGCGGCGGGAGGAGCGCGGCGCGGAGGAGCCCGGCCATGATCATGG
A

TGA

>gi_162448269_Sorangium cellulosum chromosome_54

ATGGACCGACGTGCTTTCATCCACGCGGGCGGTCTGGCCGCCGGCGCCGCCATCCTCACGCAAGGGCTCG

CCCATGCGCAGCAGCGCCCTGGCGCGCCGCCCGCCGGGGGCGCCGCTGCGCCCCCGCCTCGCCGGACCGC

GGCGCCCGGGGGGCAGCCCGCCGTGGTGACGCCGAACGGCAGCACGCTCCCGCTGCGCGTGGTGGACGGC

GTGAAGGTCGGCCACCTCGTCGCCGGCCCCCTCGAGCACGAGATCGCCCCCGGCCTGAAGAGCGAGGTGT

GGGGCTACAACGGGAGCACGCCCGGCCCGACCATCGAGGCGGTCGAGGGCGATCGCCTGCGCATCTACGT

GACGAACTCCCTGCCGGAGGCCACCACGGTCCACTGGCACGGCCTCGTGCTGCCGAACGGCATGGATGGA

GTCGCCGGGCTCACCCAGCGCCCGATCGAGCCCGGCGAGACGTATGTTTACGAGTTCGTCGTGCGGTATC

CGGGGACGTACATGTACCACTCGCATTTCGACGAGATGACCCAGATCGCGCTGGGCGCGGTGGGGATGTT

CGTCGTGCACCCGAGGAGGCCCAGGGGGCCGCGTGTGGACCGGGATTTCGTGCTGATGACGCACGAGTGG

AAGATCAACGCGGGGGCGCGCCGGCCCGACCCGAACGCGATGAGCGACTTCAACGTCCTCACGTTCAACG

GCAAGGCGTTCCCCGCGACGGCGCCGCTCGTCGTCGGCCGCGGCGAGCGCGTGCGCGTGCGCCTCGGGAA

CCTCGGCCCGATGGATCACCACCCGATCCACCTGCACGGCCTGTCGTTCCAGGTCACGGCGACGGACGGC

GGGTTCGTCCCCGAGTCGGCCCAGTACCCCGAGACGACCGTCCTGGTCCCGGTCGGCAGCACGCGGGTCA

TCGAGTTCGTCCCCGAGGAGCCGGGCGACTGGGCGATGCACTGCCACATGACCCATCACACGATGATGCA

GATGGGCCATGGCCTGCCGAACATGGTCGGCGCCGAGACCCGCGCGCTCGATCGGCGGATGCGCCGCGTG

ATGCCCGGCTACATGACGATGGGGACCACGGGGATGGGCGGCATGGGCGAGATGCCCATGCCGATCCCCG

CGAACAGCCTGCCGATGCGCGGCGGGGCCGGGCCGTTCTCGTACATCGACATGGGGGGCATGTTCACCGT

CCTCAAGGTCCGCGACGATCCGGACAAGGCGGATCCGGCCGGGTGGTACGTGCACCCGGCGGGCACCGTG

GCCGGACCGGCGAGCGCCGAGCGGATGCGGGCGGACGGGATTGACCCCGCTTCTGGAGGGAAGCCGGGCG

GGTGA

>gi_15611071_Helicobacter pylori J99 chromosome_55

ATGAAAGAATCTTTTTACATAGAGGGAATGACTTGCACGGCGTGTTCTAGCGGGATTGAACGCTCTTTAG

GACGTAAAAGTTTTGTGAAAAAAATAGAAGTGAGCCTTTTAAATAAGAGCGCTAACATTGAATTTAACGA

AAATGAAACCAATTTAGACGAAATTTTTAAACTCATTGAAAAACTGGGTTATAGCCCTAAAAAAACTCTA

GCAGAAGAAAAAAAAGAATTTTTTAGCCCTAATGTTAAATTAGCGTTGGCGGTTATTTTCACGCTTTTTG

TGGTGTATCTTTCTATGGGGGCGATGCTTAGCCCTAGCCTTTTACCTGAAAGCTTGCTTACGATTAACCA

TCATAGTAATTTTTTAAACGCTTGCTTACAGCTTATAGGCGCGCTCATTGTCATGCATTTGGGGAGGGAT

TTTTACATTCAAGGGTTTAAAGCCTTATGGCACAGACAACCCAACATGAGCAGCCTTATCGCCATAGGCA

CAAGTGCTGCCTTAATTTCAAGCCTGTGGCAATTGTATTTGGTTTATACCAATCATTATACCGATCAGTG

GTCTTATGGGCATTATTATTTTGAAAGCGTGTGCGTGATTTTAATGTTTGTGATGGTGGGCAAACGCATT

GAAAACGTTTCTAAAGATAAAGCCTTAGACGCCATGCAAGCTTTGATGAAAAACGCCCCAAAAACCGCCC

TTAAAATGCACAATAACCAACAGATTGAAGTTTTAGTGGATAGCATTGTGGTGGGGGATATTCTAAAAGT

CCTCCCTGGAAGTGCGATTGCGGTGGATGGCGAAATCATAGAGGGCGAAGGGGAATTAGATGAGAGCATG

TTAAGCGGCGAAGCGTTGCCAGTTTATAAAAAAGTCGGCGATAAAGTCTTTTCAGGGACGCTCAATAGCA

ACACGAGTTTTTTAATGAAAGCCACGCAAGATAACAAAAACAGCACCTTGTCTCAAATTATAGAAATGAT

CCATAACGCCCAAAGCTCAAAGGCAGAGATTTCTCGCTTAGCGGATAAGGTTTCAAGTGTATTTGTGCCA

AGCGTGATCGCTATCGCTGTTTTAGCGTTTGTGGTGTGGCTCATCATCGCGCCTAAGCCTGATTTTTGGT

GGAATTTTCGCACCGCTTTAGAAGTGTTTGTATCGGTTTTGGTGATTTCTTGCCCTTGCGCTTTGGGGTT

GGCTACGCCTATGAGTATTTTAGTGGCGAACCAAAAAGCGAGTTCTTTAGGCTTATTTTTTAAAGACGCT

AAAAGTTTAGAAAAAGCAAGGCTAGTCAATACGATCGTTTTTGATAAAACCGGCACGCTCACTAACGGCA

AGCCTGTCGTTAAAAGCATCCATTCTAACATAGAATTATTAGAGCTATTGAGTTTAGCGAGCAGCATTGA

AAAGAGCAGCGAACATGTCATTGCTAAGGGGATTGTAGAATACGCTAAAGAGCGTAATGCCCCCTTAAAA

GACATTAGCGAAGTTAAAGTGAAAACGGGCTTTGGCATCAGCGCTAAAGTAGATTATCAAGGCGCTAAAG

AAATCATCAAAGTGGGCAACAGCGAATTTTTTAACCCTATTAACACGCTAGAAATTAAAGAAAACGGGAT

TTTAGTGTTTGTGGGTAGAGCGATCAATGAAAAAGAAGACGAGCTTTTAGGGGCGTTTGTTTTAGAGGAT

TTGCCCAAAAAAGGCGTGAAAGAGCATATCGCTCAAATCAAAAATTTAGGCATTAATACTTTTCTTTTGA

GTGGGGACAATAGGGAAAATGTCAAAAAATGCGCGCTTGAATTAGGGATAGATGGTTATATCAGTAACGC

TAAACCGCAAGACAAGCTCAATAAGATCAAAGAGCTTAAGGAAAAAGGGCGGATCGTGATGATGGTGGGC

GATGGCTTGAATGACGCCCCTAGCCTTGCTATGAGCGATGTGGCGGTGGTGATGGCTAAAGGGAGCGATG

TGAGCGTGCAAGCGGCGGATATTGTGAGCTTTAATAACGATATTAAATCGGTTTATAGCGCGATTAAATT

AAGCCAGGCGACCATTAAAAATATCAAAGAAAATTTGTTTTGGGCTTTTTGTTATAATAGCGTGTTTATC

CCTTTAGCTTGTGGGGTTCTTTATAAGGCTAATATCATGTTAAGCCCGGCGATTGCGGGTTTAGCGATGA

GCTTAAGCTCTGTGAGTGTGGTCTTAAACTCCCAAAGGCTAAGGAATTTTAAAATTAAGGATCATTGA

>gi_154173617_Campylobacter curvus 525.92 chromosome_56

ATGCCAGCAGATTTGAACGACTATTTCAATAAGCGCAAACCAAGCGGCAATGACGGCGACAAAGAGCCTA

AGATAAATTTCAAAGCTCCGAAATTGCCAAGCGGCTTTGGTAAATTCGGCACATTAGCTTATATCATAAT

AGCCATTATCGCCGTCTTGGTCATAACTCAGCCCTTTGTCGTGATAAATTCAGGCGAGGTAGGCATCAAG

GCCACCGCAGGAAAATACGAACCAAACCCGCTTCAGCCGGGATTTCACTTTTTCGTGCCGTTCATCCAAA

AGGTCATCGTGGTCGATACCAGAGTGCGACTCATCAACTACACATCAGGCGAGGATATGGGCGAGTCGGT

GCAAAAGTCATTTCAGGGCTCTGGTGCGGGCATAATCCGCAAAAATTCCATCTCAGTGTTAGATGCCAGG

AATTTACCGGTCAGCATCGACATCACCGTGCAATACCGCCTAAATCCGGAAAACGCACCGCAGACTATCG

CGTCTTGGGGCCTTAGCTGGGAGAGCAAGATCGTAGATCCCGTAGTGCGTGATGTCGTGCGAAGTATCGC

GGGTAAATACACGGCCGAGGAGCTTCCGACCAAGCGTAACGAGATAGCCACTCAGATAGATGACAGCATA

AGAAAAGATATCGACGCTCAGCCAAACCGCCCGGTAGAGCTCTTGGCCGTTCAGCTACGCGAGATCATCT

TGCCTGAAAAGGTAAAAGAGCAGATCGAGCGCGTACAGATCGCAAAACAAGAAGCCGAGAGGACCAAATA

CGAGGTCGAGCGCGCAAATCAAGAGGCCCTTAAAAAGGCCGCCCTTGCAGAGGGAACGGCAAAGGCCGCC

ATAATCGAAGCCAAAGGTAAAGCCGACGCCGTAAAGATCGAGGCAGACGCGCAGGCTTATGCCAACAAAG

AGGTCGCTAAGAGCCTGGATGAAAATCTACTCAGCCTAAAACAGATCGAAACACAGGGTAAATTCAACGA

AGCTCTTCGCGACAACACAGATGCTAAAATTTTCCTAACGCCAGGCGGTGCGGTGCCAAACATTTGGGTC

GATACCAAAGACAAAGCAAAGCAAAGCTCCGCTACGCAAAGATAA

>gi_526459392_Arcobacter butzleri_57

ATGAAATCACAAAAATTTGACATAAAAGGTATGACTTGCTCCGCTTGTTCTACTGCTGTTGATAGAAATG

TAAAAAAATTAGAAGGAATAAATGAAGTTAATGTCAACTTACTAAATAATAGTATGATTGTAAAATATGA

TGAAAATATACTTAATAATGAAACAATTATCAAAAAAGTTCAAGATGCAGGTTATGAAGCATTTTTAGTT

GAAAATGGTAAAAAAAATCAAAAAAATTTAACAGAAGAAAATCTTGGTAAAATAGAAACTAATGAATTGA

AAAATAGATTAATTATATCTTTTATTTTTGCGATACCATTATTTTATATATCAATGGGACATATGCTAAA

TTGGTATCTTCCTCATTTATTTCATGGATATTCAAATGCTATAACTTTTGCTTTTACTCAATTTTTATTA

GCTTTACCAATAGTTTTTATAAATATAAAATACTATAAAGTAGGATTTAAAACCTTATATAAAGGTTCTC

CTAATATGGATTCTTTGATAGCTATTGGAACAAGTGCAGCTATGATTTATGGAGTATTTTCAATCTATAA

AATAGGTTATGGTTTAGGAAATAATGATATAGATATGGTGATACAATATTCTCATGATTTATATTTTGAA

TCAGCAGCTATTGTTTTAACTTTAATAACACTTGGAAAGTTTTTAGAAGCAAGAGCAAAAGAAAATACTA

GCGAAGCTATAAACAAACTTATAAATTTAACTCCCAAAACAGCTTTAGTTTTAAGAAATAATCAAGAGAT

TGAAATTCCTATTGATGAACTCATTTTAAAAGATATAGTTATTGTAAAACCTGGAAATATCGTTCCAACA

GATGGTGTTATTATCTTTGGAAATAGTTCTATTGATGAGTCGATGTTAACAGGTGAGAGTTTACCAATTT

CTAAAAAAGTTGGAGATAAAGTAATAGGTGCAAGTATAAATAAATCTGGTTCTTTCAAATTTGAAGTTAC

AAAACTGGGAGAAGATACAGTTTTATCACAAATTATAAAATTGATTGAAGAAGCAAGTTCTTCAAAAGCT

CCTATTTCAAAACTTGCAGATAGAATAAGTGCGATTTTTGTACCAACAGTTATAGTTATATCAATACTTG

CAACTGTTACATGGTTGTTTTTGGGATATTCATTTGAATTTGCATTATCTATTGGTATTGCTATTTTAGT

TATTTCTTGCCCTTGTGCTTTAGGACTTGCCACACCAACAGCTATTATGGTTGGAACTGGAAAAGGTGCT

CAAAATGGAATATTAATTAAAAGTGCAGAATCTTTAGAGATAGCTCACACTATAAATACAGTTGTTATTG

ATAAAACAGGAACTATAACTGAAGGAAAAACTCAAGTAACTGATATTTTTACAAGTGAAAAGATTACTCA

AGATAAATTACTTCAATTATGTGCAACAATTGAAAAAAACTCAGAACACCCTTTAGCTGATGCAATATTA

AAAAAAGCACAAGAAAAAGAAATTGAACTTTTAAATGCAACTGATTTTGAAGCACTTAATGGTTTAGGAA

TAAAAGCAAAAGTTGAAGATAGAGTTTTTTATATTGGAAATAAGAAATTATTAGATAGTAAAAATATCTC

TTTAGATTTATTTTATGAAAAGAGTGAAAAGCTGGCAAATGAAGGTAAAACACCAATATTTATTGCAGAC

GAAAGTGAAGTTTTAGGATTGATTGCTATTTCTGATGTTGTAAAACCAACAAGCAAAGATGCTATTTTAG

AGTTTGAAAAAATGGGCTTAGAAGTAATAATGCTAACAGGTGATAATTATAAAACAGCAAATGCAATAGC

AAAACAAATAAATATAAATAATGTAATAGCAGAAGTTTTACCTCAAGATAAAGAAAAAGAGATACAAAAG

CTTCAAAGTTTAGGTAAAAAAGTTGCAATGATTGGTGATGGAATAAATGATGCACCAGCTTTAGTTCGAG

CAGATGTAGGGATCGCAATAGGAGCTGGAACTGATATAGCAATAGAATCTGCAAATATTGTACTTGTAAA

AAGTGATTTATTAGATGCCGTAAAAGCAATTCAATTAAGTAATGCAGTTATAAAAAATATAAAACAAAAT

CTATTTTGGGCATTTTTTTATAATATAATAGGTATTCCTTTAGCTGCAGGAGTGTTTTATACAGTGCTTG

GTTGGAAATTAAGTCCAATGTTTGCAGGTGCAGCTATGAGTTTAAGTAGTGTAACGGTTGTTTTAAATGC

TTTAAGATTAAAGTTATTTGAACCAAGAATTAGTAAAAATTTATTAGAAAAAAACAATATTTCAAAAGGA

GATAAAATGGAAAAAATTTTAAAAGTTGATGGAATGACTTGCGGACATTGTAAAGCAAGAGTTGAAAAAG

TGGTTAGTGCAATTGATGGTGTTGATAGTGTTGAAGTTGATTTAGCTTCTAAAAATGTTACAGTTAAAAT

GTCAAAAGATATATCAGAGCAAACTTTATCTGATGTAATAGTAGATGCTGGATACGAAGTTATCAAATAA

>gi_152991597_Sulfurovum sp. NBC37-1 chromosome_58

GTGCAGAGGAGAACATTTATAAAGGGAGTGGCTGCAAGCTCTTTCATGGGTATTGCAGCAACGGACCTGC

AGGCGGAATCGCCCAAAAGATCGTCAGACAAGAGGCGTACACTGACGGGCAAAGAGTTTTTCTTGATAT

TGACTATACACCTGTCAACATTACCGGAAAGAGATCGGTCGCGACGACTATCAACGGTCAGATACCCGGT

CCGACTCTGGTGTGGCAGGAGGGCGATACCGTTACGATCCATGTGACAAACCACCTGAAAAAATCCTCTT

CCATACACTGGCACGGCATCATACTGCCCTATCAGATGGACGGTGTACCGGGTATCAGTTACAAAGGGAT

CGCGCCGGGCGAAACATTCACCTATCGTTTCAAGGTACGGCAGCACGGTACCTACTGGTACCACAGCCAT

TCGGGTTACCAGGAGCAGACGGGGATGTACGGCGCCATCGTCATCAAGCCGCGGAAGAGAGAACCCTTTC

GTTATGACAAAGACTATGTGGTCCTTCTCTCGGACTGGAGCGACGAAAAACCGACAAGCATCTACAGAAA

GCTCAAACTTTCAAGCGATTACTACAACTTCAACCGTCGAACTGTAGGCGACTTCTTCTCTGAAGTGAAG

AAAAAGGGCTTTGGCAAGGCATTCGATGACCGAAAGATGTGGAATCAGATGCGTATGAGCGACAGGGATC

TTTCTGATGTCACAGGGTATACCTATACCTACCTGATGAACGGGCAGAATCCTGCCACGCAGTTCAAAGC

GCTGTTCAAAAAAGGCGAGAAGATCCGTCTGCGTTTCATCAACGGTGCGGCAATGAGCTTTTTCGATGTA

CGGATACCCGGGCTGAAGATGACCGTGGTCGCAGCGGACGGGAACCACATCAAGCCCGTCAGGGTCGATG

AGTTCCGCATAGGGGTGGCCGAGACCTACGATGTCATTGTCGAGCCGACATCGAACAGAGCCTATGCCAT

TTTTGCCCAAAGTATCGAACGAAGCGGGTATGCACTCGGTTCACTGACCCCGTCGGCCTCCAAACTGGCA

AGCGCACCGAAAATGGACAAACCTCAAGCGTTGACCATGGCGGATATGGGGATGGGTATGTCCAAAATGG

GCAAGGACAAAGGTATGAAAATGCCGATGAAGAAAAGTATGAAAAAGTACAGATGGAGCGCTATGGAAGC

AGAGAAATATCCGATTACGAAATTGCCTATAGCGACAGGCCCGCAGAGTACGATGAAAGCGATGGCCCCG

AAATACCGTCTTGAAGACCCGGGGGTAGGGCTTCGCAATAACGGACGACGTGTTCTGACCTATGCCGATA

TGAAGAACCGCTATTCGACACGCAGAGCAAAAAAACCGGACAGAGAGATCGTCCTGCATCTTACCGGCAA

TATGGAACGCTATATGTGGTCGATCAACGGGATCAAATATGCCGATGCAAAACCGCTGGTATTTCACTAT

GGTGAACGGCTTCGTATCACCTTTATCAACGATACAATGATGAACCACCCCATGCACCTGCACGGAATGT

GGAGCGACCTCGAAACCGGTGACGACAACCATCTGGTACGCAAGCATACGATCGTCGTACAGCCCGGAGC

GAAGATCAGCTACCGTGTGACCGTCGATGCCAAAGGTGGCTGGGCGTATCACTGCCACCTGCTGTACCAT

ATGGCGGGCATGTTCAGAAAAGTAGAAGTGGTATAG

>gi_152989753_Nitratiruptor sp. SB155-2_59

ATGCAACGCAGAGATTTTTTGCGAAGTAGTGCACTCTTTGGTCTTATCGCTGTCACTCCTGCTCTTGCAA

AACAGGATATCGGAGAGCATCAGCCAACACAACTCCTTGAAGGAAACGAATTTGATCTGCATATTAAAAA

GACGTGGGTACGTTTTGGCGACAAGCCTGTTATAGCAACCACGGTTAACGATATGTTGCCGGCTCCCACG

CTGAAATGGAAAAAGGGTGATTTTGTTACGATTCACGTGACGAACCATCTTGATGAGCCTACCTCTTTGC

ATTGGCACGGTATCATCTTACCCTATACGATGGATGGAGTACCGGGTGTGAGTTATCCTGGGATTATGCC

AGGAGAACGCTTTACGTATCGGTTCAAGGTAGAGCAAAACGGTACCTACTGGTATCACTCTCACAGCGGT

TTTCAAGAACAGACCGGTTTACACGGGGCAATCGTGATTGATGACGATATAGATGCCGATAGAGACTATG

TGGTAGGTTTTCACGACTGGAGTGACGAAGATCCAGATACCATATATAGAAAGCTAAAACTTCAAAGCGG

ATACTACAATTTTCAAAAAAGAACAGTTTTCGATTTTGCAAAAGAGGTACAGCAGTTTGGATTTGCCAAA

GCTTTGCAAAGACGAGCAATGTGGAACAAGATGCGTATGGATGATAGAGATCTTTCTGATGTGACTGCCT

ATACGTATACCTACTGCATCAACGAACATACAAACAAAAATCCAGCCCATTTTTTCTTTAAGCCTGGTGA

GAAGGTTCGTTTAAGACTGATCAACCAATCGGCAATGAGCTATTTTGATGTAAGAATTCCCGGATTGAAG

ATGAAGGTTGTGGCTGCAGATGGCGTGCCCGTTGAACCGGTCGAGGTGGATGATCTTCGCATCGCGGTGG

CTGAAAGTTATGATGTATTGGTTTGTCCAACGCAAGAAGCCTATACCATTTTTGCTCAAAGTATCGATCG

GTATGGGTATGTGTATGGAATCTTGAGCACTTCACCTCAAAAAAGAGCTCCTATACCAGAGTCCTATCCG

TATGAACCTCTCTCTATGGTGGATATGGGTATGGCTATGGATGGGATGAAAATGAATCATATGAAGAAAA

AAATGGCTCATACAACAAAGATTCCAAAGACCCCTTTGCCTATGAAAAAGGGAATTGCTTGGACGATGGA

GGCAAAGGGTGCAAGGTATAGACTTGATGATCCAGGAGTTGGATTAAGAAGCGTTCAAAGAAAAGTTTTA

ACCTACGCTGATCTGCATACGCCAAATATCGAAAACAGATTTCCAGATCGTGAGATCCAATTGCACTTAA

CAGGCAATATGGAGCGCTATATCTGGGCAATAAACGGGATTCCTTACTACGAATCAAAACCTTTGCAGTT

TCGTTATGGTGAAAGACTGCGAATAACGTTTATCAATGATACGATGATGAATCATCCGATGCATCTTCAT

GGTATGTGGAGTGATATCGAAAAAGCAGATGGAAAAATTTTGCGAAAACACACCGTCAATGTGCAGCCGG

GCAGTAAACTGAGTATGCGCATCAATGTGGATGCAACTGGGCGATGGGTTTTTCATTGCCATCTACTGTA

TCACATGGGTGGGATGTTTCGAGAAGTAAAGGTGCTCTCATGA

>gi_255767013_Bacillus subtilis subsp. subtilis str. 168 chromosome_60

TTGAGTGAACAAAAGGAAATCGCGATGCAGGTCTCTGGTATGACCTGTGCGGCATGTGCTGCCAGAATTG

AAAAAGGCCTGAAGCGGATGCCCGGTGTCACGGATGCAAATGTTAATCTGGCAACGGAAACATCAAATGT

AATCTACGATCCTGCTGAAACAGGGACTGCAGCGATTCAGGAAAAAATAGAGAAGCTGGGCTATCACGTC

GTTACAGAAAAAGCTGAATTCGATATTGAGGGCATGACCTGTGCGGCTTGCGCCAATCGGATTGAAAAAC

GGCTTAACAAAATAGAAGGCGTCGCAAATGCACCAGTAAACTTCGCTTTAGAAACAGTTACGGTCGAATA

CAATCCAAAAGAAGCGTCTGTCAGCGATTTGAAAGAAGCGGTAGATAAGCTTGGATATAAGCTAAAGTTG

AAGGGTGAACAGGACAGTGAGGCTGCTGCTAAAAAGAAAGAAGAACGGAAGCAAACGGCGAGACTGATTT

TTTCAGCTGTCCTGTCATTTCCATTGCTTTGGGCGATGGTGAGCCATTTTACATTTACATCATTCATTTG

GGTACCGGATATATTTCTCAATCCGTGGATGCAGTTTGCGCTGGCGACACCAGTCCAGTTTCTGATCGGC

TGGCCGTTTTATGTGGGTGCGTATAAGGCGCTTAGAAATAAAAGCGCCAATATGGATGTATTGGTCGCCC

TCGGTACGACGGCTGCTTACGCTTATAGTCTTTATCTAACTTTTCAAAGCATTGGCTCTCACGGGCATAC

AGACGGCCTTTATTATGAAACAAGCGCGATTTTGCTGACGCTGATTTTATTAGGCAAGTTGTTTGAAACA

AAAGCAAAAGGCCGTTCCTCGGATGCGATTAAAAAGCTGATGAAGCTTCAAGCGAAAACAGCTACTGTTG

TAAGAGACGGACAGGAGCAAATCATCCCGATCGACGAAGTGCTTGTTAATGACATTGTTTACGTCAAACC

TGGCGAACGAATTCCTGTCGACGGAGAAGTGGTTGAAGGCCGTTCAGCGGTTGACGAATCGATGATTACA

GGAGAAAGTCTTCCTGTTGACAAAAAATCCTGGTGACAGCGTGACAGGTTCAACCGTTAACGCAAACGGCT

TTCTGAAAATTAAAGCTGTCAACGTCGGAAAGGATACTGCGCTTTCTCATATCATCAAAATTGTTGAGGA

AGCACAAGGGTCAAAAGCGCCGATCCAGCGTCTCGCTGACCAGATTTCAGGCATTTTTGTACCCATTGTA

TTGGGGATTGCGGTTCTCACTTTTCTCATTTGGTATCTTTGGGCGGCTCCTGGCGATTTTGCCGAAGCGA

TCAGCAAGTTCATTGCGGTTCTTGTCATTGCCTGTCCTTGCGCCCTCGGTCTTGCCACTCCGACGAGCAT

AATGGCTGGATCTGGACGCGCTGCTGAATTCGGGATTTTATTTAAAGGCGGCGAGCATTTAGAAAAAACA

CATCGTCTCGATACAATTGTTCTTGATAAAACCGGAACCGTGACAAACGGGAAGCCGCGTCTGACGGATG

CCATTCCATTCGGCCGTTTTGAAGAAAAAGATTTGCTTCAATTTGCAGCGGCAGCGGAAACGGGATCAGA

GCATCCGCTTGGCGAAGCGATTATTGCTGGCGTGAAAGATAAAGGGCTTGAGATACCGAAACTTACCCGC

TTTGAAGCAAAAGTCGGCGCAGGCATTTTGGCTGAAGCCGGAGGAAAAAGCATTCTCGTCGGCACAAGAA

AATTAATGGAGAGCGAACAGGTTGAGCATGGGGCTCTTCTCGCCCAAATGGAAGAGCTAGAGGCCGAAGG

GAAAACAGTTATGCTTGTATCCATTGATGGAGAAGCAGCGGGGCTAGTAGCAGTTGCAGATACGATTAAG

GATACGTCGCGGAAGGCTGTTGCCCGTCTGAAAGAGTTGGGTCTTGATGTCATCATGATGACAGGAGACA

ATCGCAGAACCGCAGAAGCCATCGCGAAGGAAGCGGGAATTGCGAATATCATTGCGGAAGTTCTTCCTGA

ACAAAAGGCGGCGGAAATCGCCCGCTTGCAAAAAGAAGGCCGCCAGACAGCAATGGTAGGCGACGGAATT

AACGATGCGCCGGCGCTTGCCACAGCTGACATCGGCATGGCGATTGGAACTGGCACGGACATTGCCATGG

AAACAGCAGACATTACACTTATTCGCGGCGATCTGAACAGCATTGCCGACGCAATCCGCATGAGCAGATT

GACAATGAAAAACATTAAACAAAACTTATTCTGGGCGCTTGGCTATAACAGCCTCGGCATCCCAATCGCA

GCACTTGGATTTTTAGCACCGTGGATAGCAGGAGCAGCAATGGCATTCAGCTCCGTTTCCGTTGTTCTGA

ATGCTCTTCGTCTGCAAAAAGTAAAATGA

>gi|29165615:2632362-2634770 Staphylococcus aureus subsp. aureus N315 chromosome, complete genome_61

GTGGCTAATACGAAAAAAACAACATTAGATATCACTGGTATGACTTGTGCCGCATGTTCAAATCGTATCG

AAAAGAAACTGAATAAACTTGATGACGTTAATGCCCAAGTGAATTTAACTACAGAGAAAGCAACTGTTGA

GTATAACCCTGATCAACATGATGTCCAAGAATTTATTAATACGATTCAACATTTAGGTTACGGTGTCACT

GTAGAAACTGTCGAATTAGACATTACAGGTATGACTTGTGCTGCATGCTCAAGCCGTATTGAAAAAGTGT

TAAATAAAATGAACGGCGTTCAAAATGCAACGGTCAATTTAACAACAGAGCAAGCTAAAGTTGACTATTA

TCCTGAAGAAACAGATGCTGATAAACTTGTCACTCGCATTCAAAAATTAGGTTATGACGCGTCTATTAAA

GATAACAATAAAGATCAAACGTCACGCAAAGCTGAAGCGCTACAACATAAATTGATTAAGCTTATCATAT

CAGCAGTATTATCTTTACCACTATTAATGTTAATGTTTGTGCATCTTTTCAATATGCATATACCAGCACT

ATTTACGAATCCATGGTTCCAATTTATTTTAGCTACACCTGTACAATTTATTATTGGATGGCAATTTTAT
GTAGGTGCTTATAAAAACTTAAGAAATGGTGGCGCCAATATGGATGTACTTGTTGCTGTTGGTACAAGTG
CAGCATATTTTTACAGTATTTATGAAATGGTTCGTTGGCTAAATGGCTCAACAACGCAACCGCATTTATA
CTTTGAAACAAGCGCCGTACTACTTACCTTAATCTTATTCGGTAAGTATTTAGAAGCTAGAGCGAAGTCT
CAAACAACCAATGCGCTTGGCGAATTATTAAGTTTACAAGCTAAAGAAGCACGCATTTTAAAAGATGGTA
ATGAAGTGATGATTCCTCTAAATGAAGTACATGTTGGAGATACACTTATCGTTAAACCAGGTGAAAAGAT
ACCTGTTGATGGCAAAATTATTAAAGGTATGACTGCCATCGACGAATCTATGTTAACAGGTGAATCTATC
CCTGTTGAGAAGAATGTTGATGATACTGTAATTGGTTCAACGATGAACAAAAACGGTACTATTACTATGA
CAGCAACAAAAGTTGGCGGGGACACTGCGTTGGCAAATATTATTAAAGTTGTCGAAGAAGCTCAAAGTTC
TAAAGCGCCGATTCAACGATTGGCAGATATTATTTCTGGTTATTTCGTTCCTATCGTTGTTGGTATCGCA
CTATTAATATTTATCGTGTGGATTACTTTAGTTACACCAGGTACATTTGAACCTGCACTTGTTGCGAGTA
TTTCCGTTCTTGTCATTGCTTGTCCATGCGCATTAGGACTTGCTACACCAACTTCTATTATGGTAGGTAC
TGGTCGCGCTGCTGAAAATGGCATTTTATTTAAAGGTGGCGAGTTTGTTGAACGCACACATCAAATTGAT
ACCATCGTTTTAGATAAGACGGGTACCATTACAAATGGTCGTCCAGTCGTGACAGATTATCATGGTGACA
ATCAAACGCTACAACTGCTTGCTACTGCTGAAAAAGATTCTGAACACCCATTGGCAGAAGCCATTGTCAA
TTATGCAAAAGAAAAGCAATTAACATTAACTGAGACAACAACATTTAAAGCAGTACCTGGCCATGGTATT
GAAGCAACGATTGATCATCACCATATATTGGTTGGTAACCGTAAATTAATGGCTGACAATGATATTAGCT
TGCCTAAGCATATTTCTGATGATTTAACACATTATGAACGAGATGGTAAAACTGCTATGCTCATCGCTGT
TAATTATTCATTAACTGGTATCATCGCAGTGGCAGATACTGTCAAAGATCATGCCAAAGATGCTATAAAA
CAATTGCATGATATGGGCATTGAAGTTGCCATGTTAACTGGCGATAATAAAAACACTGCTCAAGCCATTG
CAAAACAAGTAGGCATAGATACTGTTATTGCAGATATTTTACCAGAAGAAAAAGCTGCACAAATTGCGAA
ACTACAGCAACAAGGTAAGAAGGTTGCGATGGTTGGTGACGGTGTAAATGATGCACCTGCATTAGTTAAA
GCTGATATCGGTATCGCCATTGGTACAGGTACAGAAGTTGCCATTGAAGCAGCTGATATTACTATTCTTG
GTGGCGACTTGATGCTTATTCCTAAAGCCATTTATGCAAGTAAAGCAACCATTCGTAATATTCGTCAAAA
TCTATTTTGGGCATTCGGCTATAATATTGCCGGTATCCCTATAGCTGCATTGGGCTTACTTGCGCCATGG
GTTGCTGGTGCTGCAATGGCACTAAGTTCAGTAAGTGTTGTCACAAACGCACTTAGATTGAAAAAGATGC
GATTAGAACCACGCCGTAAAGATGCCTAG

>gi|268318562:1733707-1735632 Lactobacillus johnsonii FI9785 chromosome, complete genome_62

ATGAAGTTATCAAACATTAAGCGATTTTGGATATCGTTTATCCTCTCTATCCCCATGCTTATTCAAATGT

TTGCAATGCCATTTCATTGGATGATGCCAGGATATAACTGGATTGCTCTTATTACAACCACAATCGTTAT

GGCAATCTCTGCACTTCCTTACTGGAAAAGTGCTATTGCTGCTTTCAAAAAACATAGTGCTAATATGAAT

ACCTTAGTAGCTACTGGTACAGCTGTTGCATATTTCTATAGTATCTTTGCCATGATTACCAACCGTCCAG

TTTACTTTGAAAGTGCAGCATTTGTTACGGTCTTCGTCTTATTAGGCGACGCTATGGAAGAAAAGATGCA

TGATAACGCATCAAATGCCTTAGGTAAATTAATGGGTTTACAAGCAAAAGATGCGGAAGTTCAAAGAGAT

GGTAAATTTGTAAAAATTCCACTTGATCAAGTTCAAGTTGGCGACATTATTCGCGTTAAGCCAGGTGAAA

AAATTCCAGTTGATGGTGAGATTCTAGAAGGTGTTACTACATTGGATGAATCAATGGTTACTGGGGAAAG

TATGCCAGTAGTTAAAAAAGTTGGTGACACTGTTGTAGGGTCAACTATTAATAGTAACGGTACAATCACC

TTCAAGGCTACTAAAGTTGGTTCTGACACCATGCTTGCTCAAATTGTTGATTTAGTTAAAAAAGCTCAAA

CTAGTCATGCTCCAATTCAAAGCTTAACGGATAAAATTTCTAATATTTTTGTCCCTGCTGTCATGATTAT

TGCCATTTTAACTTTTATCATTTGGTATTCATTCCTTGGAGCAACCGCTGTTGAAGCAATGCTTTTTGCA

GTTAGTGTGATCGTTATTGCCTGCCCATGTGCTTTAGGACTTGCTACTCCAACTGCTTTAATGGTTGGTA

CTGCTCGTAGCGCTAAAATGGGAGTTTTGATCAAGAACGGTGAAGTTCTTCAAGAAGTTAGTGACTTGAA

TACTGTTGTCTTTGATAAAACAGGTACTATCACTGTTGGTAGACCTGAAGTTACTGATATTGTCGGTGAC

AAAAAACAAGTTTTAAGAATTGCTGCAAGTCTTGAAGAATCATCTGAACACCCGCTTGCTACAGCGATAG

CTAAAAAAGCAGATTCTGAAAAATTACAAATCGAAAAAGTTAGCGACTTTGAAGCAATTGAAGGTAAAGG

CGTTAAGGCTAATTACCATGATCAAACAGCTTTTGTTGGAAGCAATCGATTACTAGCCGACGTCAACATT

TCCCAGGAAATGAATAAGCAAGCCACAAAATTACAGGAAGAAGCTAAAACAGTTGTTTATGTCGGCTTAA

ATGGTGAAATTATTGGCCTAATTGCTATTCAAGATGTTCCTAAACCAAGCTCTAAAGAAGCTATCAGCGA

ACTTAAGAAACGTGGATTAAAGACAGTAATGCTTACCGGTGATAATGAAAAAGTTGCTCAAGCTATCGCT

AACGAAGTTGGCATTGACCAAGTTATTGCTGACGTTCTACCAAATGAAAAAGCTGAACATATTCAAGAGC

TTCAACAAAATGGCGATAAGGTAGCATTTGTCGGGGATGGTATCAATGATGCTCCCGCTCTCTCAACAGC

TGATGTAGGAATTGCGATGGGATCTGGTACAGATATCGCAATTGACTCTGGTGGTATTGTCTTAGTTCAA

AATGATTTAAGAGGTGTTGTTCGCGCTATTGATATTTCAAAGAAGACCTTCAACCGTATCAAGTTAAACC

TTTTCTGGGCGCTTATCTACAATACAATTGGTATCCCAATTGCAGCTGGATTATTCGTTGGCTTAGGCTT

TACTCTAAGTCCGGAACTAGCAGGTTTAGCTATGGCTTTTTCATCTGTTTCAGTTGTTGGTAGTTCATTA

CTTTTGAATAAAGCAAAAATTGCCGGAACTAATTAG

>gi|337744364:c6859801-6857372 Paenibacillus mucilaginosus KNP414 chromosome, complete genome_63

ATGGCTAATGAGCAGCTGCTCGAACAGGAGCAGACCACGTTGAAGATATCGGGAATGACCTGTGCCGCGT

GTGCGGTCCGGATCGAGAAGGGGCTGAACAAGCTCGAGGGCGTATCGAATGCCACCGTCAATTTTGCGAT

GGAGCAGGCCTCGGTCGCTTATGATCCGGCCAAGGTAGGCACGGAGGCGATCGAGCAGAAGATCCGGGCG

CTCGGCTACGATACGGTCAAAGAGAAGCTGGAGCTGCAGATCAGCGGGATGACCTGCGCCGCGTGTGCTG

CCCGTATCGAGAAGGGCCTGAACAAGCTGGACGGGGTCACGAAGGCGACCGTGAACTTTGCCCTCGAGAC

CGCCCAGGTGGAATATATCGCGGGCCAGGTGAGCCCGGCCGAGATGATGAAGAAGGTGGAGAAGCTCGGC

TACAAGGCGGCTCCGAAGCAGGAGCTGCAGGAAGCGGGAGATCACCGTCAGAAGGAGATCCGCGATCAGA

AGGTGAAGCTGCTGGTGTCCGCTGTTCTGTCCTTTCCGCTGCTGTGGGCGATGGTCAGCCACTTCTCCTT

CACCTCCTTCATCTGGCTGCCCGAGATCTTCATGAACCCGTGGTTCCAGCTGGCTCTGGCCACTCCGGTG

CAGTTCATCATCGGCAAGCATTTCTATGTCGGCGCCTACAAGGCGCTTCGCAACGGCAGCGCGAACATGG

ATGTGCTGGTAGCGCTCGGCACGTCGGCGGCTTACTTCTACAGCCTGTACCTGACGGTTCAATCGCTCGG

CGGGCATGGTCACCACGAGGTTCAGCTCTATTATGAAACGAGCTCCGTGCTGATCACCCTGATCCTGCTC

GGCAAGCTGTTCGAGATGCTGGCGAAGGGCCGCTCCTCCGAGGCGATCAAGACGCTGATGGGGCTGCAGG

CGAAGACCGCGCTGGTCATCCGGGACGGCCAGGAGATGAGCATTCCGGTGGAGGATGTGCTCACAGGCGA

GATCCTCATTGTGAAGCCGGGTGAAAAGGTGCCGGTGGACGGGGTTGTCGTGGAAGGCGTATCCTCCGTG

GACGAATCGATGCTGACCGGCGAGAGCCTTCCGGTGGGCAAACAAGCGGGCGACGCCGTCATCGGGGCGA

CGATCAACAAGAACGGCATGCTGAAGATTCAGGCGACGAAGGTCGGTCGGGAAACCGCCCTGGCCCAGAT

TATCAAGGTGGTCGAAGAAGCACAGGGTTCCAAGGCTCCGATCCAGCGGGTGGCCGACGTGATTTCCGGT

ATTTTTGTCCCTATCGTAGTGGGCATTGCCCTCGTTACGTTCCTGGTCTGGTACTTCATCGTCGAGCCAG

GCGACTTTGCGAGCGGCCTCGAGAAGGCCATCGCGGTACTCGTGATTGCGTGCCCATGCGCCCTTGGCTT

GGCGACACCCACGTCGATTATGGCCGGCTCCGGCCGGGCGGCTGAGCTCGGCATCCTCTTCAAGGGGGGC

GAGCACCTTGAGGCCACGCACCGGATCGACGCCATCGTGCTCGACAAGACGGGCACGGTGACCAAAGGCA

AGCCGGAGCTGACGGACGTTCTGGCGGCTGAGGGGATGGACGAAGCGGCCTTCCTCCGCGGTATCGGCGC

GGCGGAGCGCAGCTCGGAGCACCCGCTGGCGGAAGCGATTGTGGCCGGGATCAAGGAGAAGGGGATCGCC

CTGCCTGAAGTCGAGGCGTTCGAAGCGATTCCGGGCTACGGGATCCGGGCTGTCGTGGAGGGCCGGGAGT

GGCTCGTAGGTACGAGGAAGCTGATGGTGCAATACGGTGTGAATGCCGAGACGGCGCTGCCGGCGATGAG

CGGGCTCGAATCGGCAGGCAAAACCGCCATGCTGGCGGCCGTGGACGGTCAGTATGCCGGGATGGTGGCG

GTGGCGGACACGATCAAGGAGACATCCAAGGAAGCGGTCAGCCGCTTGAAGGCGCTCGGCATCGAGGTCA

TCATGATCACAGGAGACAACGAACGGACGGCGAAGGCGATCGCGGCCCAGGTGGGCATCGACCGTGTGCT

GGCCGAAGTGCTGCCGGAAGGCAAGGCGGCCGAGGTGAAGAAGCTGCAGGAGGCGGGCAAGAAGGTAGC
G

ATGGTCGGCGACGGCATCAACGATGCCCCGGCGCTGGCTACGGCCGACATCGGAATGGCGGTCGGCACCG

GGACGGATGTGGCGATGGAAGCCGCGGACGTGACGCTGATGCGCGGCGACCTGAACAGCATTCCGGATGC

GATCGCGATGAGCCGCAGGACGATGGCGAACATCAAGCAGAATCTGTTCTGGGCGCTGGCTTACAACGTG

ATAGGCATTCCTGTGGCCGCAGCGGGCTTTCTGGCCCCATGGCTGGCCGGAGCTGCGATGGCGCTCAGCT

CCGTATCGGTGGTGCTCAACGCGCTGAGACTGCAGCGGGTGAAGCTATAA

>gi|328956382:c2004050-2001603 Carnobacterium sp. 17-4 chromosome, complete genome_64

TTGACTGAGAAATCATTTACCATTGAAGGGATGAGTTGCGCATCATGTGCTCAGACTGTTGAAAAAGCGA

CTAAGAAATTACCTGGAGTCAAAGAAGCCAGTGTGAATTTGGCTACTGAAAAAATGCATATTCAATACGA

TGAATCGATTCTGACAGATAAAGATATCCAAGAAGCCGTAAATCAATCAGGATACAAAGCTGTAACTGAT

AAAAAACAGAAAACCTTTACCATTGAAGGGATGACCTGTGCATCATGTGCTCAAACGATTGAAAAAGCAA

CTGGAAAATTACCAGGAGTAGCAAATGTGAGTGTGAATTTAGCTACTGAAAAAATGAGTATCCAATATGA

TCCAGATCAATTAGGAGTAGCGGATATTACAAATGCCGTAAAAGAATCTGGTTATGAAGCTCATGAAGAA

ATTGAATCAGCCGATGCACTAGACATGGATCGTGAAAAAAAAGCACAACACATAAAAGAGATGTGGCAGC

GATTTTGGATTTCAGCTATTTTTACGGTCCCTTTATTGTACATTTCAATGGGCCATATGTTAGGAATGCC

ACTACCTGAAGTGATTGATCCTATGATGAATGCGGGCACTTTTTCTTTTACTCAATTGATTTTGACTTTA

CCAGTTGTAATTCTTGGAAGAGAATTTTTTAAAGTCGGATTCAAAGCTTTATTCAAAGGTCATCCCAATA

TGGATTCGCTTGTTGCTTTAGGAACGAGTGCGGCCTTTGTATACAGCTTAGCTGCAACTATAGGCATATG

GATGGGAAATGTTGGCTTAGCTATGGAGCTTTATTATGAATCTGCGGCAGTTATTTTGACGTTGATCACG

CTTGGTAAATACTTTGAAGCTCTTTCAAAAGGGAAAACTTCTGAAGCAATTAAAAAACTAATGGGTCTTG

CTCCAAAAAAAGCTAGGCTGATGCGAAATGGACAAGAGGTAGAAGTAGCAGTCGACGAAGTACAAGTAAA

TGATTTAATCATCGTGAAACCAGGTGAAAAAATGCCCGTCGATGGAGTAGTGGTCGAAGGAATGACGTCA

GTTGATGAAGCTATGTTGACTGGAGAAAGTATACCCGTTGAAAAAACAACTGGCGATGCGATAATTGGAG

CAAGTATCAATAAAAATGGAACAATTCAATACAAAGCTACAAAAGTTGGTAAAGATACAGCCTTATCTCA

AATCATTAAGTTGGTTGAAGATGCACAAGGTTCAAAAGCTCCTATTGCGAAACTTGCCGATATTATTTCT

GGATATTTTGTACCAATCGTTATCATACTAGCTATCCTATCAGGAGTAGCTTGGTATCTAGCTGGAGAAT

CAGGGGTATTTGCTCTGACAATCGCAATTTCGGTATTAGTTATTGCCTGCCCTTGTGCGCTAGGATTAGC

GACACCTACCGCTATTATGGTCGGTACAGGAAAAGGAGCCGAATATGGCGTTTTAATTAAAAGTGGCGGA

GCACTAGAAAAAACACATAAAATCGACACGATTATTTTTGATAAAACAGGCACACTAACTGAAGGAAAAC

CTGAAGTAACAGATATTGTAACTGTTAGTGAGTTATCTGAAGAGAAGTTATTGATTCTTGCAGCTTCTGC

TGAAAAAGGCTCAGAACATCCACTTGGAGAAGCAATTGTTCACGGAGCAGAAAATAAGAAATTGCCTCTT

CTTAAAACGCAAACATTCAATGCTTTGCCTGGACATGGAATTGAAGTAACAATAGAGAATCAATCTCTCT

TATTAGGAAATAAAAAATTAATGGTCGACCGCGGTATCTCACTTGATGCAGTAGAGTCAATTTCCGATAA

GTTAGCAAGCGAGGGTAAAACCCCAATGTATATTGCTAAAGATGGTCAAATGGCAGGAATAATTGCCGTT

GCAGATACAGTAAAAGCGAATAGCTTGAATGCCATTGAAAAATTGCATAAGATGGGATTAGAGGTAGCAA

TGATTACTGGTGACAATAAACGGACTGCTGAAGCTATTGCCAAACAAGTAGGAATTGACCGTGTGTTAAG

TGAAGTGTTACCGGAACAAAAAGCAAACGAAGTAAAAAAATTACAAGCCGAAGGAAAAATAGTCGCTATG

GTAGGTGATGGTATCAATGATGCTCCTGCTTTAGCACAAGCTGATATTGGTATTGCCATTGGAACAGGTA

CAGATGTAGCTATGGAATCTGCAGACATTGTACTGATGAGAAGTGATTTAATGGATGTACCAACAGCTGT

GGAATTGAGTAAAGCAACCATCAAAAATATCAAAGAAAACCTATTTTGGGCATTTGGGTACAATGTTTTA

GGAATTCCTATTGCTATGGGACTGCTCCACATTTTTGGAGGACCATTGTTAAACCCGATGTTGGCTGGAG

CAGCAATGAGTTTCAGTTCAGTATCCGTACTGATCAATGCACTGCGTTTAAAGAGATTCAAACCCTAA

>gi|336065242:c1401683-1399227 Erysipelothrix rhusiopathiae str. Fujisawa chromosome, complete genome_65

ATGGATAAAAAGACATATAATATAATTGGGATGATGTGTGCATCCTGTCAAAGCCAAGTCGAAAAAGCAT

TACAAGGTGTTGAGGGGGTTGATCATGTGGAAGTTAACTTGCTTACCAATCAAGCAGTTGTTTCTTTTCA

AAGCGACATTCCCGAAGCAGACCTAATCCAAGCCGTTGAAGATCAAGGTTATGGCCTTGAAATCCCCAAT

CCAATTCAAGAAAAAACGGTGGATCTAAAACTTACTGACATGACCTGTGCATCGTGTGTTGCGAATGTGG

AAGGTGCACTCCAACACTTAGAAGGCGTTATGTCCGCTAGTGTTAATTTAATGACGGAACGTGCACGTGT

GACCTATGACCCTCATAAACTAAAAATGGTGGATATGATCCAAGCCATTGAAAATCAAGGCTATGGTGCT

AGCCGTTTGGATGAAGCTGAAGCGATATCCACGGATTCTCAAAAACACCAAGATCAAAAAGAGAATCGTG

CGCTTTACTTTAGTTTAATCTTAGCTGCAACCATGCTCTATATTACAATGGGTCAAATGTTTACCTATAA

ACTACCCTTACCATCCTTTATCGATCCTGATATTAATCCTTTGAACAATGCGCTCTTACAAATTGTTATT

ACCATCCCCATTGTTTGGTTAAATCGTGATTACTTTAGAAGAGGCTTTAAAACATTATTTAAAGGGCATC

CTAATATGGATTCTTTAGTAGCGATTGGAACCGGTAGTGCAATGCTTTACAGTTTCTATGGATTCTTCAA

AATTTTAAATGGAGAACCTCATTTTGTACATCATCTCTACTTTGAGTCTGCAGCGGTGATTTTAGCGTTG

ATTCGTCTGGGAAAAAACCATGGAGTCTCGCAGTAAAGCGAAGACCACCAGTGCCATTAAAGCATTATTAA

ACCTTAAACCAGAAACGGCACTTTTGATTCGTGAGGATGGTGTTGTGGAAATTGATGCGGATGAAATCCG

AATTGGTGATCATCTGCTGGTAAAACCAGGTACATCCATTCCAATGGATGGTCGAATTTTAGAAGGTGAA

AGTGCGGTCGATGAGTCGATGTTGACTGGAGAAAGTATTCCAGTTGATAAAACGCGCGATGATGATGTTG

TGATGGGGACGATGAATCTTAACGGTCGTCTTGTCATCGAAGTGACCGTTGATGATCAAAATACAAAGCT

TGCACAAATTATTCGTTTGGTTGAAGATGCGCAAAATGAAAAGGCGCCCATCTCTAAAGTAGCGGATAAA

GTAGCGGGTGTGTTTGTTCCGGTGGTAATGGTCATTGCGCTTATTTCTGGAATTTTATGGTTTATCGCTA

CCAAAGATCTAGAACGTGCTCTCACCATTTTCGTAACGGTACTCGTTATTGCTTGTCCATGTGCACTTGG

ACTTGCAACGCCAACGGCCATTATGGTCGGAACCGGAGTGGGCGCACAAAATGGAATTTTTATTAAGTCA

GCAGAAGCATTAGAGGCTGCGGCTCATATTGATACTGTTGTTTTCGATAAAACAGGAACCTTAACACATG

GGAAACCTGTTGTGACGGATATCATCACGAACTTGCCTGAAAATGAATTCTTAACTATTGTGGGTTCACT

TGAAAATGCTTCTGAACATCCCTTAGCACACGCGCTTGTTTCGGAGTTAGAAGAGCGAAATCTAGATATT

TTAGCCATTGATTCATTTAAATCAATCTCAGGAAAAGGTCTTCAAGGAAGTGTTGGTGGAAAATCCATTG

CAATTGGAAATGAAGCGTTGATGGATTCGTTAAATATTTCTACAGATCGTTATGAAGCGGATGTGAAACG

TTTATCTCAAGAAGGTAAAACGGCTATGTATGTTTCTGATCAAACAGCATTAATGGGGATTGTTGCAGTT

GCGGATACCCTTAAAACTGAGTCGATTGAAACCGTAAAAGTATTACAAGATATGAACTATGATGTCATCA

TGCTTACTGGTGATCATCGTGATACGGCACATGCAATTGCCGATCAAATTGGCATCTACCATGTTTTAGC

AGAAGTGATGCCTGAAGAAAAAGCCGCAAAAATTAAAGAACTTCAAAACCAAGGTCAAAATGTCTTGATG

GTTGGAGATGGAATTAATGATGCGGTCGCCTTGGTTCAAGCCGATGTAGGGATTGCAGTCGGTACGGGAA

CGGATGTAGCGATTGAATCTGCGAAAATTGTGCTCATGAAAGACAATTTGAAAGATGTTGTGAATGCACT

GGCCTTAAGTAAAGCAACAATGCGTAATATTAAACAAAACTTATTCTGGGCATTTGCGTATAATGTTGTC

GGAATACCATTTGCAGCGGGTCTTTTTAAAGTCTTATTCCAAGGTCCACTTCTTGATCCAATGATAGCCG

GTGCCGCAATGGCACTAAGTTCCGTAAGTGTTGTTTCAAATGCATTGCGTTTACGTCGCTTTAAGATTAA

AATATAA

>gi|448819523:c2952775-2950850 Lactobacillus plantarum ZJ316, complete genome_66

ATGAGCACGACGAAACGGTTTTGGATTTCATTAATATTTTCCCTGCCACTACTGGCCAATATGATTTTGA

TGCCCTTTGGCTGGATGCTTCCGGGTGGTGATTGGACGCAGTTGATTCTGACGACCATCATTATGGCTGT

TTCGGCTCGGCCGTTCTTGCAAAGTTCGTGGGCGTCCTTTACTAAGCACCATGCCAATATGGATACTTTG

GTGGCGATTGGCACCGCGACGGCTTATTTTTACAGCTTATACGCAATGGCAACGGGGCAAGCAGTCTTCT

TTGAAAGTGCCGCACTTGTGACCACTTTCGTTTTGCTCGGCCAAGTCTTTGAAGAACGAATGCGGCACAA

CGCGTCCAGCGCGGTCGCAAAGCTAGTGGGCTTACAAGCCAAGGAAGCGGAAGTGTTACGCAATCAAGAA

TTTGTAAAAGTACCGTTGGATCAAGTAGTGGTCGGCGACATTATTCGGGTCAAGCCAGGCGAAAAAATTC

CCGTTGATGGTGTAATTACAACTGGCCGCTCAACGGTTGATGAGTCAATGGTGACGGGAGAAAGTATGCC

GGTTCAAAAAACGGTGGCGGACGCTGTCATTGGTTCAACGATTAATAGTAATGGGACCTTTCAGTTCAAA

GCTAGCAAGGTCGGTGACCAGACAATGCTGGCTCAAATCGTTGAGCTCGTTAAAAAAGCACAAAACAGTC

ACGCACCGATTCAAAAGCTGACCGATAAAGTTTCTGATATCTTTGTGCCGATTGTGCTAATTATTGCTAT

CGTTACCTTCTTGACCTGGTATGTTTTCTTGAATGCTAGTCTCGCGAATGCCTTGATTTTTGCGGTCGCT

GTGGTCGTGATTGCCTGCCCGTGTGCGCTAGGTTTAGCTACGCCGACCGCACTAATGGTCGGTACCGGCC

GTGGTGCCAAGATGGGGATCCTGATTAAAAATGGTGAGGTCTTGGAAGCTGTCAATGACGTTAAAACGGT

GATCTTTGATAAGACGGGAACGATTACGGTTGGTCACCCACAAGTGACCGATGTTATCGGCGACGAAGCC

CAAGTACTGCGCGTTGCCACTAGCTTAGAGGCGGCTTCGGAACATCCTTTAGCTGCGGCAATATTAAAAC

GCGGCACGGCCCGTGGTATTCAAGCACCAACCGCCACGAACTTCCAAGCCATCGAGGGTAAAGGTGTGAC

TGCTCAAGTTGAGGGCCAATTAGCGGTGGTCGGTAATCATAAATTATTAACTGATGAGCCGATTAGTGCT

GAATTACAACGCCAAGCCACTGAGCTTCAGGACGCGGCGAAAACGGTCGTGTACGTGGGCTTAAATCACG

AAATCATTGGATTGATTGCCATTCAAGACGTCGCCAAATCGACGTCTGAGGTAGCCATTGCGAGTTTGAA

AGCACGTGGCTTGAAGACGGTCATGTTAACCGGCGATAACGAACGGGTCGCACAAGCTGTGGCGGATCAA

GTCGGTATTGATGAAGTGATTGCTGATGTGCTGCCAGGTGATAAGGCTGATCACGTTCACGCGTTCCAGA

AGGCTGGCAAGGTGGCCTTTGTCGGCGATGGTATTAATGACGCCCCCGCGTTGACGACGGCGGATGTTGG

GATTGCCATGGGTTCCGGAACTGATATTGCAATCGATGCTGGTGGCATCGTGCTCGTTAAGAACGATCTG
CGTGATGTTGACCGGGCGTTGGCATTGAGTAAGAAGACGTTTAACCGTATCAAACTTAATCTGTTCTGGG
CTTTCGTGTACAACGTGCTCGGGATTCCAGTCGCGGCGGGCCTCTTCTTTGCCATTGGGCTGACGCTTAG
CCCCGAATTGGCCGGATTAGCAATGGCCTTTAGTTCATTGTCCGTAGTCACGAGTTCGGTATTGCTAAAT
AAAGCTAAGATCAGTTCGAACGCCCAACCTGCGTAG

>gi|448236310:817551-819947 Geobacillus sp. GHH01, complete genome_67

ATGGGAGAGCCGAAAACCGTGACGCTGAAGGTGACGGGCATGACATGCGCCGCGTGCGCCAATCGGATTG
AGAAAGTATTAAATAAGATGGACGGCGTCAAAGCCCATGTCAATTTGGCCATGGAAAAAGCAACGATTCA
ATACGATCCATCAAAACAAACGATCGCCGATATCGAAACGAAAATTGAGAATTTGGGGTATGGCGTTGCG
ACGGAAAAAGTGACGCTTGATATTGAAGGAATGACATGTGCGGCCTGTGCGACGCGGATTGAAAAAGGGT
TAAATCGGATGGAAGGTGTGACAAGCGCTGCTGTCAACTTGGCGACAAACAGCGCGGTGGTCGAATACAA
GGAGGGTGTTGCATCTGTCGAAGACATTTTAGAGAAAATCAAAAAGCTTGGGTATAAGGGGCAAATTCGA
AACGAAGAACAAGACGATGCTGGCCGGAAAGAAGAGCGGCTGAAACAGAAGCAACGGCAACTCGCCATTT
CCATCATCTTATCGTTGCCGCTGCTTTATACGATGCTGGCTCATATGCCGTTTGATATCGGCTTGCCGAT
GCCGCAGCTCTTGATGAATCCGTGGTTTCAGCTTCTTTTAGCTACACCCGTTCAGTTTTACATCGGCGGT
CCTTTCTATGTCGGGGCGTACCGGGCGTTGCGAAACAAGAGCGCGAACATGGACGTCCTAGTGGCGCTTG
GAACATCGGCCGCGTACGTTTACAGCTTGTACGAAGCTTTTCGGACACTTGGGAATCCTGACTATATGCC
AAGACTGTATTTTGAAACAAGCGCCGTCTTGATTACGCTTGTGCTTGTCGGCAAATACTTTGAGGCTCTT
GCGAAAGGGCGGACAACCGAAGCGATCTCGAAGCTAGTCAGCCTGCAGGCAAAGGAAGCTACTGTCATCC
GTAATGGGGAAGAAATAAAAGTTCCGCTCGAGGAAGTGGTGATCGGCGATACGATCGTTGTCAAGCCAGG
AGAAAAAATTCCGGTAGACGGTATGGTCATCGCCGGAGCATCTTCCGTAGACGAATCGATGATTACCGGT
GAATCGATCCCGGTTGATAAAAAGGAAGGCGACTATGTGATCGGGGCGACGATGAATACGAATGGCGTAC
TGACCATTCGTGCCGAAAAAGTCGGAAAAGATACCGCGCTGGCCAATATCATTAAAATCGTTGAAGAGGC
GCAAGGGTCGAAAGCCCCGATTCAGCGGATGGCGGATACCATTTCCGGCATTTTCGTACCGATTGTTGTC
GGAATTGCGGTTGTTTCCTTTCTAATCTGGTACTTCTTCGTTGCGCCGGGTGATTTGGCCAAAGCTCTTG
AGGTGGCCATCGCTGTTCTTGTCATCGCGTGCCCTTGTGCGCTCGGTTTGGCCACACCGACATCGATTAT
GGTCGGTACAGGAAAAGGGGCAGAACAAGGAATTCTCTTTAAAGGAGGTGAGTACCTAGAGGGAACGCAT

AAAATCAATGCCGTATTACTCGATAAAACAGGAACCGTGACAAAAGGAAAACCGGAAGTCACCGATGTGC

TAGCCTTCCGCGAGGACATGCTCGATTATGCGGTTTCCGCCGAGAGCGCTTCGGAGCATCCGCTGGCTCA

TGCGATTGTTGAATACGGGAAGAAACAAGCGATTTCGATGAAGCCATTGGAGCACTTCTCCGCCATTACC

GGCCACGGAATTGAAGCTGTCATTGACGGAAAAAGCATCCTTATTGGGACGCGAAAATTGATGAAGGAAC

GCTCTGTAGCGATTTCTGTGCATGAAGATAAAATGGTAGAGCTTGAAAAACAAGGGAAAACAGTGATGCT

CGTTGCCATCGATGGACAGCTTGCCGGCATCATTGTTGTCGCGGATACGGTGAAAGAAAGCTCGAAAGAA

GCGATTCAAACATTAAAACAAATGGGCATCGATGTCTATATGGCAACAGGGGACAATCAACGGACAGCGG

AAGCGATCGCCAATGAAGTAGGCATTGAGCATGTGTACGCCGAGGTGCTCCCGGAAAACAAAGCGAACAT

CGTCGAGGAATTGCAAAAGCAAGGAAAACGTGTGGCGATGGTCGGCGATGGGATTAACGACGCTCCAGCT

CTGGCCAAAGCGGATATTGGGATGGCGATTGGCACAGGAGCTGATGTCGCCATCGAGACCGCTGATGTGA

CCTTGGTCGGCGGCGATTTGCGCCATATCCCGAAAGCCATTGAGTTGAGCCGCAAAACGATGAAAAACAT

TCGGCAAAATCTGTTCTGGGCGTTGTTTTATAATACGATTGGCATTCCGGTCGCCGCTTTCGGATTATTG

GAGCCATGGATCGCTGGAGCGGCGATGGCATTCAGCTCTGTGTCGGTTGTCGCCAACGCGCTTCGTTTGA

AACGCGTGAAACTATAA

>gi|508605261:c3180599-3178383 Carnobacterium maltaromaticum LMA28 complete genome_68

ATGGAAAATACACAAAAAAAGCAAACAACGATGGAAACTTTAGCAATTACAGGAATGACCTGTGCGAATT

GTTCACAAACAATTGAAAAAACATTAAATAAAGAATCTGGGATTACATTAGCTAACGTCAATTTAGCAAC

AGAAAAAGCCACAGTTACCTATAATCCTGCAGAAACAACATTACCAAAAATTATTAAAAGTATTGAAGCT

GCAGGGTATGGAGCAATTGTCTACGATGAAGAGCACCAAAAAGAAGTGGCTGCGTTAAAAGAGAAGAAGA

GTCAAAGATTAAAAATTGATTTTATAGTTAGTGCTATTTTAAGTGCTCCAATGGTTATCTCAATGATTTT

TATGTTGGTAGGGTGGCATAATCCGATTACAGGTTTCTTTGAAATTCCGGTTGTTCAGTTTATTCTAGCT

ACACCTGTTCAATTTATTATTGGTTGGCGTTTTTATAAAGGAGCATACAAAGCGATTAAAGCGGCTTCTC

CTAATATGGATGTTTTAGTTGCAATGGGAACAACGGCTGCATATGCTTTGAGTATTTATAATGCTTTTAT

TGCTAACCGTCCCCATGATTTGTATTTTGAAAGTAGTGCTGTGATTATTACGCTGATTCTACTTGGAAAA

TATTTAGAGCACAATGCTAAAAATAGAACAGGATCTGCTATCAAACAATTAATGGCGTTGCAAGCTAAAA

CGGCTGTCGTTATTCGAGATGGTGAAGAAATTACAGTACCTATTGAGTCTGTTCAGTTATATGATTTGAT

TCGAATTAAACCAGGTGAACAGATTCCAGTAGATGGTAAGATTATTGAAGGAACAACTTCGTTAGATGAA

AGTATGCTGACAGGGGAAAGTTTACCGGTAGAAAAAACAGTCGATGATACTGTTTTTGGCGGGACTGTAA

ACACGAATGGAACGTTAGTCCTTCAAGCGACTAAAATTGGAAGCGAGACAGCTTTATCGCAAATTATTCG

AATGGTGGAAGAAGCGCAAGGCAGTAAAGCGCCGATTCAACAAATTGCCGATCGAATTTCAGCAATATTC

GTACCAATTGTGTTAGTCGTTGCTTTAGGTACTCTGATTGTTACCGGTTGGTTAACGGGTGATTGGGAAT

TGGCTTTGATTCATAGTGTGGCTGTTTTAGTTATTGCTTGTCCTTGTGCGCTAGGCTTAGCAACACCAAC

TGCGATTATGGTGGGAACCGGCTTAGGAGCAAAAAATGGAATTTTGATTAAAGGTGGCGAAGCTTTAGAA

GGAGCTGCTAAAATTCAGGCCATTATTTTAGATAAAACTGGAACTATTACTAAAGGAAAACCAGAAGTAA

CTGATTATGAAGTGCTACCAGGTGAAAATTCTGAGGAACTATTAAGTTTTATTATGGCTTTAGAGAAAAA

TTCGGAACATCCTCTGGGTAAAGCTATTTATAACTATGGCAAGAAAAAATTGAATGGTGAAGAGCAGACT

GTTGAGAACTTCAAAGCGTTGGTTGGATCTGGAATTTCTGGTGATATTAAAGGCGAAACTATGTACATGG

GTGCGAAACGTTTAATGGATGAAAATCATAGTGATTATTCGCATTTTTCTGAAAAAATTACAGCATTAGA

AGGTAGCGGGAAAACGGCTATGCTTTTTGCAAAAGGTGATCAATGTGTGGCTATGATTGCAGTGGCAGAT

CAAATTAAAGAAACATCTAAAGAAGCAATTCAAAAATTAAAACAAATGAACGTCGAAGTCTATATGTTGA

CTGGGGACAATCGTCTGACTGCTGAGAGTATCGGAAGCGAAGTTGGAATTGATGCTGCACATATTTTTGC

AGAAGTATTGCCTGCAGATAAGGCAAATCATGTCGTATCTCTTCAAGAAAAAGGTTTATCTGTTGCAATG

GTAGGGGATGGAATTAATGATGCTCCTGCTTTGGCAACTGCAAATGTTGGTTTAGCGATGGGAACAGGAA

CAGATATTGCCATGGAAGCAGCAGATATTACCTTAATGAATGGCGATTTAAATAATATTCCTAAAACGAT

TCATTTATCAAAAGTTACATTGCGAAAAATTAAACAGAATTTATTCTGGGCGTTTATTTACAATACAATT

GGAATACCATTTGCCGCTATTGGCTTATTAAACCCAATCGTTGCTGGAGGCGCAATGGCCTTTAGTTCGG

TTAGTGTATTGTTAAATTCTTTAGCTTTAAATCGTACGAAAGTTTAA

>gi|409131816:c2096979-2094421 Thermacetogenium phaeum DSM 12270 chromosome, complete genome_69

ATGTTACCGGGGGTGAACGTCATGGAAAACGGGGCGGAAAGAGCAGGGCCCGCCCGGGTGACTTTGCCCG

TACGGGGTATGACCTGTGCGGCGTGTGTCGCCAGGGTGGAAAAGACCCTGAAAAACATCCCGGGAGTGGA

GGGAGCCCGGGTGAACCTGGTAGCCGGGAAGGCTGCTGTGGATTACCTTCCGGAGCGGGTGAGCGTGCCT

CAAATGGTCAAGGCCATCCAGGAGATCGGCTACGAAGTACCGGAGGAAGAGGTGCTCTTGACCGTTCGGG

GTATGACCTGCGCTGCCTGTGTGGCCCGGGTGGAAAGAACTCTTAGAGCTCTGCCGGGAGTAACGTCGGT

GGTGGTCAACCTCCCGGCTGAGTCGGCAAAGATCCGGTTCTACCCGGGTGCCGTTGACAAATCCCGCATC

AAGCAGGAGATCAATGCCCTTGGTTACGAGGCTTCGGAAAAGCTGACGGGTCAGGAGGCACTCGACCGGG

AGAAAGAGGCCCGGGAACGGGAGATCAGGTACCAGCGGCGCAATATGTGGATCGCCTGGCCGTTGGCGAT

TCTGGTTATGGTGGGCATGTTCCGGGATATGTGGATTTTCCCGTACTTTGTACCAAAATTCCTGGGCAAC

GTCTATGTGCTTTGGGCCCTGACCACACCGGTAGCCTTCATCCCCGGCTGGCAGTTTTTCGTGCACAGCT

GGAACGGTCTGAAGCGGGGTACCACCGACATGAACCTCCTTTATGCCACAGGTATCGGCGCCGCTTACCT

GATAGCTACAATCAATACCCTGTGGCCCAATGCGGGTTTCGGCGGCCGTGGCGCGACCTTCTTTGAGTCG

GCCGCCCTGCTGACAGCCTTTATCGTGCTCGGGCGCTACCTGGAGGCCCTTACCCGCGGCCGGACTTCCG

AGGCCATCAGGAAGCTGATGAGCCTGCGGGCCAGAACCGCATTGGTTGTCCGGGACGGCCGGGAGATCGA

GATTGCAGCCGATGAGGTAGAGGTGGGGGACATTGTAGTGGTTCGTCCCGGCGAAAGCATCCCGGTGGAC

GGGGAAGTGATCGAGGGTTATTCGGCCGTGGATGAATCGATGATTACCGGCGAAAGCATCCCTGTGGAAA

AACGCCCTGGCGCCCAGGTGATTGGAGCGACCATCAACAAGACCGGCTCTTTCAAGTTCAGGGCTACCCG

GGTGGGGAGCGAGACGACACTCGCCCAGATCATCAAGCTGGTGGAGGATGCCCAGGCGTCCAAGGCACCC

ATTCAAAGGCTCGCCGATTTTGTGGCAGGTCATTTCATTGCCGGGGTCCATGTCCTGGCCCTCCTTGTCT

TCCTGTTCTGGTTCTTTATAGGCTATAACGCTTTCTTCCTGCCCGGCAGCCGCTTCATCCTCTCGCCCTT

TAGCCTGGCCCAGGTGGGCGTCTTCGGGTTCTCCCTGCTGTTGTCGGTAACCACCCTTGTGATTTCCTGC

CCATGTGCTCTCGGGTTGGCCACTCCCAGTGCGGTCATGGCCGGGACGGGCAAGGGCGCCGAAAACGGGA

TCCTCTTTAAAGGCGCCGATGCGGTTGAGGAGAGCAGCAAGCTGAATGCTATAGTCTTTGATAAAACGGG

GACCTTGACCAAAGGGGAGCCTTCCGTCACCGATATTATTGCAGCGGAGGGGTTCGATAAACAAGAGGTT

CTGAGGTTGGCCGCCATGGCCGAAAAACCGTCCGAGCATCCCCTTGGGGAGGCCATTGTCCGCGGAGCCC

GTGACGAAGGCCTTGAGATCGAGGATGTTCGGGACTTTGAAGCCGTTCCGGGCCACGGCGTGCGGGCCGT

GTACCGGGGGCGGGAGATCCTTTTGGGCAACCGCCGTTTGATGCAGCAGAGAAACATCAACATAGGTGAC

CTGGCAGCCCGGATGGAGGAACTGGAAGAGGAAGGCAAGACCGCTATGTTGCTGGCCGTTGACGGTAAGG

CCGCCGGTGTGATCGCCGTGGCCGACACTCTCAAGGAAAGCACAAAGGTGGCGGTAGAGCGCCTGCAGAA

AATGGGCATTCAGGTGGCCATGATTACCGGAGATAACCGCCGCACGGCCGAGGCCATTGCCCGGCAGGTT

GGGATCAAGACCGTGCTGGCCGAGGTGCTGCCCCAGGACAAGGCCGAAGAAGTTAAAAAGCTTCAGGAAA

GAGGGCTCAAGGTGGCCATGGTGGGTGACGGGATCAACGATGCTCCGGCCCTGGCCCAGGCGGACGTGGG

GATTGCCATCGGCTCCGGAACCGATGTGGCAAAGGAGACGGGGGACATTATCCTGATCAGGGATGACCTT

CGCGATGTGGTGGGAGCTATCGAAATCGGCAGAGCGACCATGGGGAAGATCAGGCAGAACCTGGTATGGG

CTTTCCTGTACAACGCCCTGGGGATTCCCATTGCCGCCGGAGTCCTGTATCCCTTCACCGGGCTCATCGT

CAGCCCCGAACTGGCAGCCTTTTTTATGGCCATGAGTTCGGTATCGGTAACGTTAAATACGCTCCTTTTA

AAGCGGTTCCGGCCTTCCCTCCGGGAGAGGCCTGTGTAG

>gi|407475894:c256770-254644 Exiguobacterium antarcticum B7 chromosome, complete genome_70

ATGTCGAAAACGATTGAACTGAATATCGAAGGCATGACCTGTGCTGCCTGTTCCGCACGGATCGAAAAAG

TCTTGAACCGGATGGACGGTGTCGAAGCGACCGTCAACTTACCTCTTGAGACGGCACGGATTGCGGTACC

GGACGAGATGGAAGAACAGGTGATCCTTGATAAAATCAAAAAAGTCGGTTACGGTGCGACACTCAAGACG

ACGCCCCACGAACAGGTTCAAAATCGTCGGATGCTCTATCGGTTTTTCATCGCTGCGTTACTGTCGTTAC

CCTTGTTACTCAGCATGGTGTCGCATATTCCGAACAGTCCGCTTCATCTGCCTTTCCTGATGAATCCGTG

GCTTCAATTTGCACTTGCGACACCTGTCCAGTTCATCATCGGGGCCCCCTTCTACAGCGGTGCCTATAAG

AGTCTCCGCAGCGGCAGCGCCAACATGGATGTTTTGGTCGTACTCGGAACTTCCGCGGCTTATTTCTACT

CGATTGCAGAAATGCTGATCCATCCGCTCATGCCAAATCTCTATTTTGAGACAAGTGCCGTCCTGATTAC

GCTTGTCCTTTTGGGAAAAGTGTTAGAAGATCGGGCCAAACAACAAACGACCGGCGCCATCAAAAGTCTT

CTTTCCTTACAGGCGACCGATGCTGTCGTACTTGAAGACGGCATCGAACGGAAAGTCTCGATGGAGCATG

TCCAAGTCGGGATGCAACTCGTCGTTAAACCCGGACAAAAAATTCCGGTTGATGGCGTGATCGTCTCTGG

AGACGCCTATCTCGATGAATCGATGTTGACCGGTGAACCGCTTCCTGCTCATAAAACTGTTCATGACGCT

GTCATCGGAGGGACGTTAAATACGAATGGTCATCTCCTGATTGCAGCGACCAAAGTCGGTCAAGAGACGA

TGCTTGCGAACATCATCCGGGTCGTCGAACAGGCGCAAACGGAAAAGGCACCGATTCAGCGACAAGCAGA

CCGAATTTCTGGTATCTTCGTTCCAATCGTCGTCGCCATCGCCATCGTGACACTTGCTGTTTGGTGGTGG

ACGACCGGCTCGTTTGCCGAAGCCATCCGGCCGGCGATTGCCGTGCTCGTCATCGCCTGTCCGTGTGCCC

TTGGACTTGCGACACCGACCTCGATCATGGTCGGAACGGGTAAAGGAGCAGAACACGGCGTACTGTTCAA

AGGTGGTGCCCAGCTGGAATCGTTACAACATGTCGATGCCGTCGTCTTTGATAAGACAGGTACGTTGACG

ATTGGCCGACCTGTCGTCGTGCAAACGTTCGGACAGGAACAAGCACTCGATTTTGCGGCAGCCATTGAAA

AAAAATCAGAACATCCCCTTGCCCATGCAGTCACGGAAGAACGAGATGTCGTCTTTGATATTGAAGACTT

CACCGTCGATGCCGGACGTGGTGTCCGCGGAACGATTTTAGGACATCACGTGATGGTCGGTTCAGCACGG

ATGATGCGTGAGCATGAGCTGATTCTCCCCGACTGGACTTCCACTGGTGCGACGTTCGTCTATGTTGCGG

TCGACGGACAGATCGAAGCCGGTTATGCGATTCGGGATGAACTCAAACCAACAACGAAACACGTCATTCA

AGAGCTTCAGCAAACTAAAGCGGTCTATCTGTTGACGGGCGACCGACAAGAAGTCGCCTTGGAGCTCGCA

CATGAACTTGGTATTCCGCAGACACAGGTCTTTGCCGATGTGTTACCGATTGAAAAAGCAGATAAAATCA

AAGCCTTACAAGCAACGGGTCAACGGGTGGCGATGGTCGGCGATGGCATCAATGACGCACCAGCCCTTGC

GACAGCAGATGTTGGGATTGCGCTTGGCAGCGGGACAGATGTCGCACTTGAAGCGGCAGATGTCACCCTG

CTTGGACATGATCTGCAACAAGTCGCAACAGCCATCCGCTTAAGCGAGCAGACGATGAAAAACATTCGTC

AAAATCTGTTTTTTGCACTCGGCTACAATACGATTGGAATTCCGATTGCTTTCCTCGGACTCCTTGCCCC

ATGGGTCGCAGGTGCCGCGATGGCCTTCAGCTCTGTATCTGTCGTCACAAATGCGTTACGCTTAAAACGT

ATTCCATTATCCAAAGGAGGAAAATGA

>gi|392987295:1790883-1793066 Enterococcus hirae ATCC 9790 chromosome, complete genome_71

ATGGCAACGAATACCAAAATGGAAACATTTGTCATTACTGGAATGACATGTGCGAATTGTTCTGCTCGAA

TCGAAAAAGAACTAAATGAACAACCTGGTGTCATGTCTGCTACTGTCAATTTAGCCACTGAAAAGGCAAG

TGTGAAATATACCGACACTACTACAGAAAGATTGATCAAAAGTGTCGAAAATATCGGCTATGGGGCAATT

TTATATGACGAAGCGCATAAACAAAAAATTGCTGAGGAGAAACAAACCTATTTAAGAAAAATGAAGTTTG

ATCTTATCTTTAGTGCGATCTTGACTCTACCCTTGATGTTAGCAATGATTGCCATGATGCTTGGAAGTCA

TGGACCAATTGTGTCGTTCTTCCATCTGTCTCTTGTGCAGTTGCTCTTTGCTTTGCCTGTGCAATTTTAT

GTAGGTTGGCGCTTTTATAAAGGAGCCTATCATGCATTAAAAACTAAAGCGCCGAATATGGATGTCTTAG

TTGCGATTGGAACATCTGCAGCCTTCGCATTAAGCATTTATAATGGTTTTTTCCCTAGCCATTCCCATGA

TCTTTATTTTGAAAGTAGCAGTATGATCATTACCTTGATTTTACTTGGAAAGTATTTAGAACATACAGCA

AAAAGTAAAACAGGCGATGCCATCAAACAGATGATGTCCCTTCAGACAAAAACAGCACAAGTTTTAAGAG

ATGGCAAAGAAGAGACGATTGCAATTGATGAGGTCATGATCGATGACATCTTAGTGATTCGTCCTGGTGA

ACAAGTACCTACAGATGGACGGATCATTGCTGGCACTAGTGCATTGGATGAAAGCATGTTGACAGGAGAA

AGTGTACCTGTTGAGAAAAAAGAAAAAGATATGGTTTTTGGTGGAACGATCAATACCAATGGATTGATCC

AAATACAAGTTTCTCAGATAGGAAAAGATACGGTGTTGGCACAAATCATCCAAATGGTGGAAGATGCTCA

AGGAAGTAAAGCACCGATCCAACAAATTGCTGACAAGATTTCAGGGATTTTCGTACCGATCGTTTTGTTT

TTAGCATTGGTGACACTATTAGTTACAGGATGGCTCACGAAGGATTGGCAGTTAGCATTGCTTCATAGTG

TGTCCGTTTTAGTCATTGCTTGCCCATGTGCGCTTGGTTTAGCAACACCAACTGCCATCATGGTAGGAAC

AGGGGTCGGTGCTCATAATGGGATATTGATCAAAGGTGGCGAAGCGTTAGAAGGAGCCGCTCATCTAAAT

AGTATTATTTTGGATAAAACTGGAACGATTACACAAGGCCGACCAGAAGTAACAGATGTCATCGGTCCCA

AAGAGATCATTTCCCTTTTCTATTCCCTTGAACATGCTTCTGAACATCCTTTAGGAAAAGCTATTGTTGC

TTATGGTGCAAAAGTCGGTGCAAAAACTCAGCCAATCACTGATTTTGTTGCTCATCCTGGTGCAGGAATC

AGTGGAACGATCAACGGTGTTCATTATTTTGCTGGGACTAGAAAGAGACTAGCTGAAATGAACCTTTCAT

TTGATGAATTTCAAGAACAGGCGTTAGAATTAGAACAGGCAGGGAAAACAGTGATGTTTTTAGCCAACGA

AGAACAGGTTCTTGGAATGATTGCCGTTGCTGATCAAATCAAAGAAGATGCAAAACAAGCAATCGAGCAA

CTACAACAAAAAGGTGTCGATGTGTTTATGGTTACGGGAGATAATCAACGAGCCGCTCAAGCAATCGGCA

AACAAGTAGGAATTGATTCCGACCATATCTTTGCAGAAGTTTTACCTGAAGAAAAAGCCAACTATGTAGA

AAAACTACAGAAAGCTGGAAAGAAAGTTGGCATGGTCGGTGATGGAATCAATGATGCCCCAGCGCTAGCT

TTAGCAGATGTTGGGATTGCAATGGGAAGTGGAACCGATATTGCGATGGAAACAGCTGATGTGACATTAA

TGAATAGTCATTTAACTTCTATCAATCAAATGATTTCTTTATCAGCTGCCACATTAAAAAAAATCAAACA

AAATTTGTTTTGGGCATTCATTTATAATACGATCGGGATTCCTTTTGCTGCCTTTGGTTTTTTAAACCCA

ATCATTGCTGGTGGCGCAATGGCCTTTAGTTCAATCAGTGTATTATTGAATTCTTTAAGCTTAAATCGAA

AAACGATCAAATAA

>gi|384133787:390174-392552 Alicyclobacillus acidocaldarius subsp. acidocaldarius Tc-4-1 chromosome, complete genome_72

ATGTCAGAAACCCGCGAATTGACGTTGCCTATCGAAGGCATGACGTGTGCCGCCTGTGCGGCGCGGATCG

AGAAGAACCTGGCGAAGCTGCCGGGCGTGCAGCAGGCGAACGTGAATCTCGCGTCCGAGCGGGCCCGCGT

GGTGCTGACCTCCAACACGCCGTGGACCGAGGTGATCGACAAAATTGAAAAGACCGGGTACTCGGTCCCG

CTGCGCGAGGTGGACCTGCGCATCACCGGCATGACGTGCGCCGCGTGCGCGGCGCGGATCGAGAAGGTGG

TCGGGCGCCTGGACGCGGTGAAAGAGGTAAGCGTCAACCTTGCGTCGGAGAAGGCGCGGGTGGCGTATGT

GCCGGGCGTGATCGAAGTCGAGGATATCATTCGCGCGGTGGAGAAGGCGGGCTACGGCGCGGCGCTTGCG

AGCGAGGTGGAGGCGGAGGAAGAGGCGCGCAAGCGGCGGGCGTATCGGCGCGATCTCGCCACGTTCGCCC

TGTCCGCCCTGCTCACGCTGCCGCTCGTCGTCCAGATGTTTGTCATGCTCGCGGGCGGGCGCCCGTTTCT

TCCGAACTGGGCCGCCTGGCTTCTCGCGACGCCCGTCCAGTTCCACGTGGGATGGCGCTTCTACAAAGGG

GCGTACCACGCGCTGCGCGGCGGCGCGGCCAACATGGACGTACTCGTGGCGCTCGGCACCACGGTGGCGT

ACGTGTTCAGCGCCGTTCTGACGGTGGAGGGGCGCCGGGACGTGTACTTTGATAGCTCCGCCACGGTCGT

GACGCTCATCTTCATGGGCAAGCTGCTCGAGGCGCGGGCGAAGGCGAGATCGAGCGCGGCCATCTCGTCG

CTCGCGAAGATTGGGGCGAAGGTGGCGCACGTGCTGCGGGACGGCGAGGAGGCGGATGTGCCGGTTGAGG

CGCTTGCGGTGGGCGATCTCGTCCGCGTGAAACCGGGCGAGCGGGTGCCTGCGGACGGCGTTGTGGTGGA

AGGATGGACGAGTGTCGACGAGTCGTTTTTGACCGGCGAGCCGCTGCCTGTGACCAAGCGCCCGGGCGAT

GAGGTGGTGGGCGCGTCCCTGAACCAGGCGAATCCCTTCGTGATGCGCGTTGCGAAGGTGGGGAGGGACA

CGGCGCTGGCCCAGGTCATCCGGCTGGTGGAGCGCGCTCAAGGATCGAAGGCGCCGGTGCAGCGCCTGGC

GGATCGCATCTCCGGCGTCTTTGTCCCGGCCGTGCTGGGGGTGGCCCTCGTCACGTTCCTCGTATGGGGC

GCCTTGGGGCACTGGTCGCACGGGCTGTTTGCGGCGATTGCAGTGCTCGTGATTGCCTGCCCGTGCTCGC

TCGGGCTTGCCACGCCGACGGCCATCATGGTGGGCACGGGGCACGGCGCCGAGCAGGGAATCCTCGTGAA

GGGCGGCGAGCACCTGGAGATGGCAGCGCGCGTAGACACTGTCGTGTTTGACAAGACCGGGACGCTGACG

GTGGGGAAGCCCGTCGTGACGGAAGTCTGGGCAGCCGAGGGCGTCCTGGAAGAGGACGTGTTGAGGTTGG

CGGCCGCACTTGAGGCGCAGAGCGAGCACCCGCTCGGACGCGCGGTGGTCGAGGCGGCGAAGGCGCGCGG

CGTGGAGATTCCGGCGGCGAAGGACGTGACGGCCGTGCCGGGGCGCGGCATCGAGGGCGTGGTGGAGGGT

GTGCGCGTGCGGGTGGGCAGCCCGAGCTTCGTGTCCTCCGCTGCGCCGGGCCTGCCGGAGGAAGTGCGCG

GCAAGCTCACGGGGCCGGGCCGCACCGTCGCGGTGGTGGCGCAGGGGAATCGGGTGATAGGTGCCATTGC

CATGGCCGACGAGGTGAAGCCGGACGCGCGCGAGGCGGTGGCTGCGCTTCGGGAGATGGGCGTGGACGTG

TGGATGATGACCGGCGACGCGGAGGAGACCGCGCGCGAAGTGGCGGCGCGCCTCGGCATTGAGCACGTGA

TGGCCGAGGTCATGCCTGGCGACAAGGCGGCGAAGGTGGAGGCGCTACGGAAGTCCGGGCGCGTCGTGGC

CATGGTCGGCGACGGCATCAACGACGCGCCGGCGCTCGCCGCGGCGGACGTGGGCATGGCGATGGGCACG

GGCGCCGATGTGGCGCTCGAGGTTGCGGACGTGGCGCTGATGCGCGGCGACGTGTGGGCCGCGGTGGACG

CGCTGCGCCTCGCCAAGGCGACCATGCGCAAGATCCGGCAGAACCTGTTCTGGGCGCTCGTGTACAACGT

GCTCGGGATTCCGCTCGCTGCCTTAGGCGTTTTGAGCCCCATCATCGCCGGGGCCGCGATGGCCTTAAGC

TCAGTGAGCGTGGTGTCAAACAGCCTGCTCTTGCGGCGCACGCGCCTCGGCTCGCCGTTGCGCCAGTAG

>gi|339626328:c3356477-3354051 Sulfobacillus acidophilus TPY chromosome, complete genome_73

ATGGCGGTGACACCCGAATCTCAGAACGACCAGACCGTAACGGTCGAAATCGGCGGTATGACATGCGCCA

GTTGTGCGCTTAGCATCGAAAAGGCGTTAAACCATCTACCCGGAGTCGGCCGGGCAGAAGTCAATCTGGC

CTTGGAACGAGCCGATATTCGGTTTGATCCGGCGACCGCCCCCATTCCCGAGTTAGTGGACACGATCACC

CAGTTGGGCTATACGGTACGGCAAGATTCGATAACCTTGATGGTGCCCGGGATGGACGAGGAACCGCTTC

GCGAACGGGCCGAACAGGTTGCCCAACGCATTACCGGGGTGGTTGCGGCCCGCGGGAACTTGGCAACCGG

TACGCTCACGGTAGATTTTCTGCGGGGGGTCGTCGAGATCCCGGAGATTATCGCGCGCTTGGCCGACGCG

GGGATTACCGCCCGGGTAGTGGGCAATGACCAAATGGATGCCCGTGCGCTGGAGATGGCCGAATCGCGTC

AGCGGCTGGTCAGTGCGGTCATTTTGACGGTACCGATCTGGCTCGCCATGCTCCATACCATGACCGGTCT

CGGACCGCGGTGGCTCGGGGAGCCGTGGGTATTGGCCGTTCTGGCGACGGCCGTCGAATGGGGACCGGGT

TTTCCGTTTATCAAACGGGCTTATCTCAATTTACGACATAAAAATGCCAATATGGACGTGTTGGTGGCAG

TAGGCACATTGGCGGCGTGGGGTTTGTCGCTCTATGATTTGAAGGTTCACGGGCCGTTATATTTTGATTC

GTCCGCCACCGTCATCACGTTGGTGTTAGTCGGTAAATACTTGGAGTCGGTGGCCAAAGGCCGTACCGGT

CAGGCGCTGACGGAGCTTTTGGCGTTACGCCCGGCATCGGCACGGCGGCAGACCGCGTCCGGCGACTGGG

AATCGGTCGCGGTCGAATTGATTCAGGTCGGGGACCGGGTGCAGTTACGGGCGGGTGACCGGGTGCCGGT

CGATGGTCGTGTGTTGGAAGGTCACAGTGCGGTCGACGAATCCATGCTCACAGGAGAACCCTGGCCTGTA

CCGAAGGGGCCGGGCGATCCGGTTACGGCAGGCACCATGAACGGCTCCGGCACATTGGTAATGGAAGCCC

AGCGGGTTGGACGGGACACGGCCTTGGCGCAAATTGTACGGGTCGTGGAAATGGCGCAAGCGCATAAGGC

GCCGGTGCAGCGCTTGGCCGATCGCATCGCCAACGTCTTTGTCCCTACGGTGATGACGATTGCCCTGTTG

ACGTTTTTAGGGTGGGGACTCGCAACGCACGACTGGCGTGCCAGCGCCTTAATTGCGGTCGCGGTGCTGG

TGGTGGCTTGCCCCTGTGCCCTCGGGTTGGCGACACCCACGGCGGTCATGGTCGGCTCCGGATTGGCGGC

CCGCCAGGGGATTTTGTTTCGCAGCGCGGAGGCGCTCGAGCGGGCGAGCCAGGTGGACATTGTGGCATTT

GACAAAACCGGTACCTTAACGGTGGGGCGTCCGGAAGTCGAAGAGATTCTGACCGCGCCGGGTGTGTCGG

CGGGAGAGCTCATGGCCTGGGCAGCGGCGATTGAAAAGGCATCCAGCCATCCCCTGGCTCGAGCTGTGCT

GAGCTATGCCGAAGACCATCGATTGGCCGTTCCCGAGGGCGAAAGTATTTATACGGAACCGGGCCAAGGA

ATGGTGGGTTATATTGGCAGCCAAGAGATTTTGGTCGGGAATTTAAAGCTTCTCACGGCTTATGGGGTCA

TACCCGACGAAACGGTCATCCGAAAAGTCGATCAGCGGCTTAAATCCGGTGCCAGTGCCATTTGGGTCGC

TCAGGACGGTCAATGGCGGGGAGTCGTGGTGGTGGCCGATACGCTGCGACCGGAGGCGCGGGACACGGTT

AAACGGCTCCAACGGCAGGGGCTCGACGTGGCCCTTTTGACCGGCGATCAAAAAGAGACCGCCCACAGTG

TCGCCCAACGTCTCGGTATTCACCGCGTGGCGGCGGAATTGCTACCGGAAGCCAAGGCCGAGACCGTGCG

GGAATGGCAAGCCGCCGGACACCGCGTATTGATGGTCGGCGACGGCATTAACGATGCGCCCGCTTTGGCC

ACGGCCGACGTCGGGGTAGCCATCGGGACGGGTACCGATGTTGCGATCGAATCGGCGGATATCACCCTTT
TAGGCGATGATTTGTCCGGCGTCGTCCGCATGTTGGTGATTGCCCGCAAAAGCTTAGGTAAAATTCGTCA
AAACCTCTTCTGGGCGATGATTTATAACGTGCTCATGATTCCGTTAGCCGCTTTCGGTTTCTTGTCGCCG
ATGGTGGCCGGCGCGGCGATGGCGTTTAGCTCGGTCTCGGTGGTGTCCAATTCCTTGCTGCTGCGGATGG
CACGACTGCCTCGAACGCGTCAAAGACCGCTGGCCAAACCCTCGTAG
>gi|555077094:c2133897-2131291 Tepidanaerobacter acetatoxydans Re1 complete genome_74
ATGAATGTGGAAAAAACTTATAATGTAACCGGCATGACATGTGCTGCATGCTCGGCTGCCGTTGAACACA
GCGTAAAAAAGGTTTATGGTGTAGAGTCGGTAACAGTAAACCTGCTGACAAACAGCATGCGTGTTAAATA
CGATGATGAAAAGACATCAGATATTGAAATAATAAAAGCCGTAGAAGATGCGGGATATGGTGCATCTTTA
AAAGAGAAAAAGGGAATAAAGCAAAAAGCAGAAACAACTAAAGCTAAGACTTTGGCAGATGCGGAAATAG
AGGATATGAAACTGCGCTTGAAGGTTTCTATTTTATTTATGATACCTCTAATGTATATATCCATGGGACA
TATGCTAAATCTGCCGCTGCCGTCTTTCTTATCCGGCATAGAAAATGCGGTTTCTTTTGCCTTTGCTCAG
TTTTTATTGGTGTTACCGATTGCTTTTGCTAATCGTAAATTTTACACAATAGGTTTTAAAACTTTATTTA
AAGGTCATCCAAATATGGATTCGCTGATAGCTATCGGCTCAGGCTCGGCCCTTATCTATGGTGTATTTGC
CATTTTTCGCATGAGTTATGGTTTGGGTGCCGGCGATTTTGAATTAGTCCACAGATACTATCATGATTTA
TATTTTGAATCAGCAGCCATGATTTTAACCCTTATCACAGTAGGAAAGTTTTTAGAAACTCGAGCCAAGG
GTAAAACCACAGACGCTATTAAGAAATTGATGGACCTTGCGCCAAAAGCCGCCAGGGTAGAAAGGGACGG
CTTGGAAGAAGAAATCCCGGTAGAGTCGGTCAAAGTAGAAGATATTATAGTAATACGTCCTGGAGATTCA
ATCCCGGTGGATGGTATAGTGATTGAAGGGCAATCTGCCGTGGACGAATCCGCATTAACTGGTGAAAGTA
TTCCGGTAGAAAAGCAGCCTGGAGACAGGGTAAGTGCAGGGACTATAAACAGAACAGGTGCTTTTAAATT
TAGGGCTACACAAGTGGGAGAAGATACGACACTTGCTAAAATAATAGCATTGGTGGAAGATGCAAATGCC
ACGAAAGCTCCGATAGCAAAGCTCGCAGACAGGATAAGCGCCGTCTTTGTCCCCACAGTAATCTGTATTG
CTCTTATTGCAAGCATAGCGTGGCTTTTTGCAGGCTACAGTTTTGAATTTGCTTTGTCAATAGGGATATC
GGTTTTAGTTATCTCATGCCCCTGCGCATTGGGACTTGCTACGCCTGTTGCGATAATGGTCGGAACCGGA
AGGGGGGCAGAGCACGGAATATTAATAAAGTCGGCAGAGGCTTTAGAGGCGCTTTGCAGTATAAACACCG
TTGTCATAGATAAAACCGGAACATTGACACAAGGTAAGCCTCAGGTAACAGACATTGTGCTTTGCGGGGC
AGAATCCGAAGAAGAACTTTTGAAAATTGCCCAGGCATTAGAGAAAAATTCGGAACACCCGCTGGCAGAA

GCTATTTTAAGTTATTGCAAAGAAAGAGAAATTCCGCTGATAGAAATTGACGAATTCAGGTCTATTCCAG

GCCGAGGCGTAGAAGGGTACATTAAAGGGATGGCATACTTTGCGGGAAATGAGCAGTTTATCAAAGAAAA

GCAAATTGACATAAACATGGCTGTTGAGCTGTCGGACAACTTTTCAAATGAAGGTAAAACCCCGCTTTAC

TTTGCAAGTGCTCATACATTACTAGGGATTATTGCTGTTGCAGATGTTTTAAAACCTACTAGCCGCCAAG

CGGTTCAGAAATTTAAACAAATGGGCATTAAAGTAGTTATGCTTACAGGAGACAACCCTAAGACGGCAGA

AGCCATACGCCTGCAGTTGGAGATTGATGAAGCAATAGCAGGAGTGCTGCCTCATGAAAAGGATAAAAAG

ATTCAAGAGCTTCAAAGCCGGGGACAAAAGGTTGCTATGATTGGCGACGGAATTAACGATGCACCTGCTT

TAGCAAGAGCCGACGTAGGAATAGCCATAGGTGCAGGAACAGATGTTGCCATTGAATCTGCGGATATCGT

CCTTATAAGAAGCGATTTAATGGATGCGGTGGCGGCTGTAGAACTTTCCAAAGCAACAATAAGAAATATA

AAGCAAAATCTTTTCTGGGCGTTTTTCTACAACACCTTGGGCATTCCCATTGCTGCAGGAATCTTATATC

CTGCCTACCATATAAAGCTTACTCCTATGATTGGTGCGGCAGCTATGAGCATGAGCTCGTTTTTCGTAGT

TACGAATGCGCTGAGGCTTCGAAAATTTAAACCTACTGTACCTAAAACAGAATGTAGCGTTCAGTGCGGC

ATGCAGAAAAATGCATCAATGACCGTTGCTAAGGTAAGTAATTCCTCAAATAATAAAAATAATGAAAGGG

AGATTGAAAAAATGAAAAAAGTTTTGAAAATCAAAGGCATGATGTGTGAACACTGTAAGATGCGTGTTGA

AAAGGCTTTAAATGCAATAGATGGAGTTAACGCAAGTGTTAATCTAGAGGATGGTTCAGCCGAGGTAGCA

TATCTAAAACCGATATCTGATGAAACGTTAAAACATGTTATAGAAGAAGCAGGGTATGAAGTAACATCCA

TTGTGGAGGAGGTATAA

>gi|297625198:2086157-2087692 Propionibacterium freudenreichii subsp. shermanii CIRM-BIA1 chromosome, complete genome_75

ATGAAGGCAACTCGACGCAGCGTGCTGGCGGGATCCATGGCAGTGGCGGGCAGCTGGTTGCTGGCCGCCT

GCTCGGCGAACAGCCCGTCCAACTCGGTCTCGCAGGCCTTCGCCACCCCCAGCGCACTCACCCCGAGCCC

CGGCCAGCACGTGGTCACCAGGACCATCACCGCCTCGGCCTTCACCGCCGACCTGGGTGGGGTCAATGTC

AACACCTGGGGCTACGGCAACACCCTGCCCGGCCCCGAACTGCGCGCCAGCGCCGGCGACCTGCTGCGCA

TCACCCTGGACAACCAGTTGCCGGCCCCCACCACGATCCACTGGCATGGCATCCAGATCCGCAACGCCGC

CGACGGCGTGCCGGGCCTCACCCAGGACGCCGTCACCCAGGGCACCAAGTTCCTGTACGAGTTCGTGGCC

CCCGATCCGGGCACCTATTTCTTCCACTCCCACCAGGGCGTGCAGCTCGACCGCGGCCTGTACGCGCCGC

TCATCATCGATGACCCCGCCGAGCCCGGTGGCTACGACCAGGAATGGGTGGTCACCCTGGACGACTGGGT

GGACGGCACCGGCCGCACCCCCGACCAGGTCTTCGCCGACCTGAAGTCCAGCGGTGGCGACGACTCCTCG

TCGTCGTCGAGCCAGGGCATGGGCGGCATGGGTGGCATGCACGGTTCCGGCATGGGCGGCATGAACGGTT

CCAGCATGGGCGGCATGAACGGTTCCAGCGGCTCCATGATGGGCGACTCGCCCTTCGGCTCGGTGGGCGA

CGTCGCCTACCCGCACTACCTGATCAACGGGGCAATCCCCGCCGCACCGCGCACCTTCAGCGCCAAGCCC

GGCCAGCGGGTGCGCATCCGGCTGATCAATGCCGCCTCCGACACCCTGTTCAAGGTGGCGCTGGGTGGCC

ACACGATGAGCATCACCCACACGGATGGCTATCCCGTGCAGCCCCACGACACCAGGACGCTGTACATCGG

CATGGGCGAGCGCTACGACGTGCTCGTGACACTGGCCGACGGCGTGTTCCCGCTGGTGGCCCAACCCTTC

GGCAAGCAGGGCCAGGCCCTGGCGCTGGTGCGCACCGGCGCCGGCGACGCCCCGTCCGCAGATGTGCACC

CCACCGAGCTGGACGCCGACGCCACCCGAGCCGGCGAGCTGACCCCCACCGACGCGGCACGCCTGCCCGC

GCACGACGTGGACGCCCATGCCGACGTGAAGCTGGCCGGCACGATGAGTCCCTATGTGTGGACGATCAAT

GGTGATGTCTTCGGCAAGAACACGCCCATCGGGGTCAGCGAGGGCCAGCGGGTGCAGCTCGACGTGACGA

ACTCGTCGATGATGAGCCATCCCCTGCACCTGCACGGCCACACCTTCGCGCTGCCCAATGGCGTGCGCAA

GGACACCGTCGTGGTGCCGCCGATGACGTCGACGAGCCTGCAACTGCAGGCCAGCAATCCCGGCGCCTGG

GCCATGCACTGCCACAACATCTACCATGCCGAGGCCGGCATGATGATTGCGCTGAATTACCGCTGA

>gi|308175814:736015-738018 Arthrobacter arilaitensis Re117 chromosome, complete genome_76

GTGAACAGGCTCCAAAAATGGGTCTATGGGAACTGGTCAGTCCCCGTCGTATCCGGCGTGCTCATCATCA

TCTCCTTCGCCATCCAGCGGCTGGCCGGGGGTGTCGCCAACCTCACCGTCAGCCCCCAGTGGTGGCTCGA

CGCCGGGGCCCACGCCACTCACGCCAGCGCGGCTTTCACCCTCGGGGATGTCTTCATGATCGCCGCGGCC

GTGGTTGCCGGCTACGGGATCGTGGCGAAGGCGGTGCGCGCGCTGATCGCCAAGGTCGTCGGCATCGACC

TGCTCGTGTCGGTTGCGGCTATCGGCGCGGTCATCATCGGCAACTTCTGGGAGGCCGCGGCGGTCACGTT

CCTGTTCGCGATCGGCCACGCGCTGGAAGCTGCGACCCTGAACAAGACCCGCTCGGCACTGGCCGAACTC

GTCGCCGTCGCCCCGGATTCCGCGATCGTTGTGCGCGATGGCGAGCAACAGGAGATCCCTGCCGGGCAAG

TGAGAATGGGCGAGATCGTCCTGGTCAAGAACGGTGCGAAGGTCCCCGTGGACGGCCAGGTCGTCTCCGG

CACCGGTGCCATCGACGAGGCCTCCATCACCGGCGAGTCCATCCCGGTGGAGAAAACTAAAGGCGGGCAG

GTCTTCGCCGGCACCGTCTCCCGCGGCGGCTTCCTACAGGTCCTGGCCACTGGCATCGGCGCAGATACCA

CCTTGGCCCGCATCATCCACCGTGTGGAAGAGGCTCAGGACGCCAAGGCCAAGACCCAGGCCTTCATCGA

CCGGTTCTCCACGTGGTACACCCCCGCCGTCATGGTCCTGGCCCTGGTGGCCGGACTTATCAGCGGAGAC

GTGGTGCTGGCCCTGACCCTGTTGGTCATCGGCTGCCCGGGCGCCCTGGTGATCTCCATCCCGGTCGCCA

TCGTGGCGGGCATCGGCCGCGCCGCGCGCAACGGCATCCTCATCAAGGGCGGCGAGTTCCTGGAGACCTC

CGCGAAGATCACGGCGGTCGCCGTGGACAAGACCGGCACCCTCACCGAGGGCAAACCGCAGCTGACCGAC

GTCATCATCCTGGATTCCACGCTCGACCGTACGGAGGTGTTGCGCTGGGCCGCGGCAGCTGAGGCCGGCT

CCGAACACCCACTGGCCCGCCCCATCCTGGAGACCGCCCGCGAGGAGGGCGTTGCCCCGCAGGGCATCCC

CGGCGCCGTCACCCCGGTCCCCGGCAAGGGCATCGTGGCGAACGTCAATGGCCGCCAGGTGCTGATCGGC

AACCCGCCGCTACTGGAGCAGTACGGCATTGCCTCGGGCATCGCCGAGGCTGCGCAGGCCGCCCAGGACC

TGGCCGCCGCCGGGAAAACACCTATGATCGTCGCTGTTGACGGGAGGGTGATCGGCGTCGTCGCCGTCGC

GGACCAGATCCGCCAGGACGCACCGGAGATGGTCGCGCGACTGCACCGGGCCGGCGTCGAGAAGGTCGTC

ATGCTCACCGGTGACACCCGACCGGTGGCCGAGGCCGTCGGCAAAGCCACCGGGATCGACGAGATCCACG

CCTCCCTGCTCCCTGAGGACAAGCTGGACGCTGTCACCGAGCTACAGCGCCAGGGCCACACGATCGCGAT

GGTCGGTGACGGCGTCAATGACGCTCCGGCTCTCGCCACCGCGAACATCGGCGTGGCGATGGGTGCGGCA

GGCTCGGCCGTGGCCGTGGAGACCGCGGACATCGCCCTGATGGGCGACAACCTGCTCAAGCTGCCCGAGG

CCATCGGCCTGGCCAAGCGAACCGTGAACGTGATGAAGCAGAACATCGCGATCGCCCTGATCACCGTGGT

GCTGCTACTGGCCGGAGTCTTCGCCGGCGGAGTGACCATGTCGATCGGCATGCTCGTCCACGAAGCCTCC

GTGCTCGTGGTGATCGCCAACGCGATGCGGCTGCTGCGAAACACCCGAGACTCCACGACGATGCCAAAGA

GTGAACGCACCATCGGGACAGCTGCGGAGCAGGTGGCCGCCTGA

>gi|226303489:4793163-4795424 Rhodococcus erythropolis PR4 DNA, complete genome_77

ATGACGTCACCGACTGCGGTCGACAATGCTGCCAACTCGAGCAGCGTCGAACTCGCGATCACCGGAATGA

CCTGCGCCTCCTGCGCGTCACGAATCGAGAAGAAGCTCAACAAGATCGACGGCGTCACAGCCACGGTCAA

CTACGCGACGGAAAAAGCCAAGGTCACCTTCCCGGACGGGGTACCCGTCGAAGATTTGGTCACCGCCGTC

GAGAATGCCGGTTACGCTGCCGCGCTCCCGAAACCGCCCAGCGCGCCAGACACCGTCGACCCGGATGCGG

AGACCCCGCAGGATCGTGATCTGCGCGAGCTTCGTACCCGCCTGATCATCTCGACTCTGCTCTCCGTGCC

GGTCATCGCGATGGCGATGATTCCTGCGTTGCAGTTCGACAACTGGCAGTGGCTCTCCCTCACGCTGGCC

GCACCGGTCATCGTGTGGGCGGCCCTTCCCTTCCACCGGGCTGCCTGGGTCAACCTGCGTCACGGCGCCG

CAACGATGGACACGCTCATCTCTCTGGGCACCATCGCAGCGTTCGTCTGGTCGCTGTATGCACTGTTCTT

CGGCACCGCGGGCGAAGCGGGAATGAAACACGCGTTCAGACTGACGGTCGACCGGATGGACGGCAGCGCC

AACATCTACCTCGAGGTTGCCGCGGGCGTCATCACGTTCGTGCTCGCCGGACGCTACTTCGAGCACAAGT

CCAAGCGCACGTCCGGCGCCGCACTACGCGCGCTCATGGAACTCGGTGCAAAAGACGTCGCGGTACTGCG

AAACGGCGTCGAAGTACGAATCCCCGCCGAAGAACTGGCAGCGGGCGACTCCTTCATCGTTCGGCCCGGC

GAGAAGATCGCCACCGACGGCATCATCACCGAAGGCCGCTCAGCCGTCGACATGAGCATGTTGACCGGCG

AATCCGTCCCGGAGGAGGTCGGTGTCGGAGACACCGTCGTCGGGGCCACCGTCAACGTCGGTGGAACATT

GACCGTCGAGGCCACCCGAGTCGGGAGCGACACCCAACTCGCCCAGATGGCGGCACTGGTCGAAGACGCA

CAGAACGGCAAGGCCGAAGTTCAGCGCCTCGCCGATCGGATCGCCGGTATCTTCGTACCGGTCGTCATCG

CGATTGCCGTTGCGACACTGGGCTACTGGCTCGGTACCGGCAACCCCATCGAGATGGCGTTCACCGCTGC

GGTCGCCGTGCTGATCATCGCGTGCCCGTGTGCTCTCGGATTGGCAACGCCCACAGCTCTGTTGGTGGGC

ACGGGCCGCGGAGCCCAGATGGGAATCCTCATCAAGGGACCCGAGGTGCTCGAATCGACGCGCGCCGTCG

ACACCGTCGTGCTCGACAAGACGGGAACCGTCACCACCGGTCAGATGACATTGCTGGACACCGTGGTGGG

CGAAGGAACAACCGTCGACGAGGTCCTTCGGCTGGCCGGTGCACTCGAACATCGCTCCGAGCACCCCATC

GCCGCTGCGATCGCGAAGGGTGCGACCGAAGCCGTGGGCGAATTGCTTGCGGTCGAGGATTTCGTGAACG

TCGCCGGTCGCGGCGTGCAGGGAATGGTCGAGGGTCATGCCGTTGTTGCCGGCCGAGACGCGCTGCTCGC

CGACTGGTCGATGCCGTTGCCGGAAAATCTCCGTGACGCGAAAGCTGAAGCAGAGAAGGCCGGGCGCACC

GCAATCGCGGTGGGCTGGGACGGCAAGGCGCACGGCGTCCTCGTAATCGCCGACGCGGTCAAGGACACGA

GCGCCGAAGCAATCGCGCAGTTGAAGGCACTCGGCCTCGAACCGGTTCTGCTGACCGGTGACAACGCCAC

CGTCGCCCGCACGGTGGCGGATCAGGTCGGGATCAGCACCGTCTTCGCCGAGGTCATGCCGGCGGACAAG

GTCGACGCCGTCAAGAAGCTGCAGGCGGAAGGACGCGTCGTAGCCATGGTCGGCGACGGCGTGAACGACG

CGGCGGCCCTGGCTCAGGCCGATCTCGGTCTGGCGATGGGCACGGGTACCGACGTTGCGATCGAGGCCAG

CGACATCACCCTGGTTCGAGGTGACCTGCGGTCGGCAGCCGATGCAATTCGACTGTCCCGCAAGACGCTC

AAGACCATCAAGGGCAACCTGTTCTGGGCATTCGCCTACAACACGGCTGCGATCCCGCTGGCTGCGGCCG

GCCTGCTCAACCCGATGCTCGCTGGTGCCGCAATGGCATTCAGCAGTGTGTTCGTGGTGAGCAACAGCCT

GAGGTTGCGGCGGTTCAAGTAA

>gi|148271178:1187078-1189552 Clavibacter michiganensis subsp. michiganensis NCPPB 382 chromosome, complete genome_78

GTGACCGACCCCGCATCCGGATCCGCGCCCGCTCCGGCCGCCGATGCCGCCCCCGCATCGGCGTCCGACG

GCGGCCCGGTGCTCACGCGCGTCGACCTCGACGTGCAGGGCATGACGTGCGCGTCGTGCGCGATGCGCAT

CGAGCGGAAGCTCGGCCGGATGCCCGGGGTCGAGGCCGCCGTCAACTACGCCACCCATCGGGCTCGCGTG

CAGCTGCCCACGGGCACGAGCGTCGAGGACGCCATCCGCACCATCGAGCGCACGGGGTACCGCGCGTCCG

AGCGGGCCGGGTGGGGAAGCGCGCCGGCGGACGGCGTGTCCGGATCCGCTGCTGCTGCTGCTGCTGCTGC

TGCCGCTGCCGCCGCATCCGCATCCGCGGAGCGCGCCCGTCGCGGTCGCGGCTCCCCCGTCCGCCCG

GACGCGCCCGCCGCCGCCTCGGATCCCGCACCCGCGCCCGCCCCGGAGGATCCGCCCGCCCGCGACGCCG

TCGCCGCCCGCCGCCCCGACGCCGATGAGCTCGCGCTCCGGCAGCGGCTCGTGGTCTCCGCGGCGCTCAC

CGTCCCCGTGTTCCTCATGGCGATGCTCCCCGCGCTCCAGTTCCCGAACTGGCAGTGGCTGTCGCTCGCG

CTCGCCGCGCCCGTCGCGGTCTGGGGCGCGTGGCCGTTCCACCGGTCCGCGGCCGTCAGCGCGCGCCACG

GCGGCGTCGGCATGGACACGCTCGTGAGCATCGGCGTCGCCGCCGCGTTCCTCTGGTCGCTCTACGCGCT

CTTCCTCGGCGACGCGGGCCGACCGGGCATGCGCATGACCTTGAGCCTCGTCACGGAGCCCGGCGGCGGA

TCCGGCGACGTCTACCTCGAGGTCGCCGCGGCCGTCACGGTCTTCCTGCTCGGCGGCCGCTACCTGGAGG

CGCGTGCCGCCCGCGCGTCCGGCGCCGCCCTCGCCGCGCTCCTCGACCTCGCCGCGAAGGACGTCGCGGT

CGTGCGCGACGGCGTCGAGCGGCGGATCCCCATCCGCGAGCTGCGCGTCGGCGAGGAGTTCGTCGTGCGG

CCGGGCGAGCGGATCGCGACCGACGGCGTGGTCGTCGACGGATCCAGCGCGGTCGACCGCTCGCTCCTCA

CGGGCGAGTCGCTGCCCGTCGAGGTCGGCCCCGGCGACGACGTCACGGGCGCGACCCTCAACGCCGGAGG

CCGGCTGCTCGTGCGCGCCACGCGCGTGGGCGAGGAGACCCGTCTCGCCCGCATGGCCGCGCTCGTGGAG

GAGGCGCAGACGGGGAAGGCGCGGATCCAGCGCCTCGCCGACCGGGTCTCGGCCGTGTTCGTGCCCGTCG

TGCTCGTGCTCGCGGTCGGCACGCTCGTCGGCTGGCTGCTGCTCGGCTTCCCGCCCGAGGCCGCGTTCAC

CGCGGCCGTGGCGACCCTGATCATCGCGTGCCCGTGCGCCCTCGGACTCGCGACGCCGACCGCACTCCTC

GTGGGCACGGGCCGCGGCGCCCAGCTCGGGATCCTCATCACCGGCCCCGAGGTCCTGGAGTCGACCCGGC

GCATCGACACCGTGCTGCTCGACAAGACCGGCACCGTCACCACCGGCGTCATGTCGCTCGTGCGGGCCGT

GGCCGCGGCGGGCGTCGACGCCGATGAGCTCGTTCGCGTGGCCGCCGCCCTCGAGGCGCGGTCGGAGCAC

CCGGTGGCGCGGGCCGTCGTCGAGGCGGCGGGTGCCGGATCCGTCCCCGCCGTCAAGGGCTTCGTCGCGA

CCGCGGGACTCGGCGTCCACGGCGTGGTGGACGGCCGCGCGGTCGCGGTCGGCCGGCCGTCGTGGCTCGC

GGAGCAGTGGGCCGCCGAGCCGGACGCGTCGCTCGCCGAGGCCTTGCGCGGGGCCGAGGCGGAAGGATCC

ACCGTGATCGCGGTCGCGTGGGGCGGTGCCGTCCGCGGGATCCTCGCGGTCGCGGACACCGTCAAGCCCA

CGAGCGCCGAGGCCATCCGCCGCATCCGCGCCCTCGGCCTCCGTCCCGTGCTCCTGACCGGCGACACGGC

GGGCGCCGCGCACCGCGTCGCCGCGGAGGTCGGCATCGACGAGGTGATCGCGGGCGTCCTCCCCGAGGGC

AAGCTCGACGCGGTGCGCCGCCTGCAGGCCGAGGGGCGCGTCGTCGCGATGGTCGGCGACGGAGTGAACG

ACGCCGCCGCCCTCGCGCAGGCCGACCTCGGGATCGCGATGGGCACGGGCACGGACGCGGCCATCGAGGC

GGGCGACATCACGATCGTCCGCGGCGACCTCGTGCTCGTCGCCGACGCCGTCCGGCTCGCGCGGCGCACG

CTCGGCACCATCCGCGGCAACCTGTTCTGGGCCTTCGCGTACAACGCGGCCGCGATCCCGGTCGCGATGG

CCGGCCTCCTCAACCCGCTCGTGGCCGGGCTCGCGATGGCGCTGTCGTCGGTGTTCGTCGTCACGAACAG

CCTGCGCCTGCGGTCCTTCCGGTAG

>gi|300781937:c7223833-7221563 Amycolatopsis mediterranei U32 chromosome, complete genome_79

ATGTCTTCCGAGACGACCACACCCGCCGCGCAGAGCGTCGAGCTCGCGGTGCGCGGTATGACGTGCGCCG

CCTGCGCGGCACGGGTGGAACGCACCCTCAACAAGCTCGACGGTGTCCGCGCGACGGTCAACTTCGCGAC

CGAACGCGCGGCCGTCCGCTGCCCGGACGACCTTGATCCGGCCGTGCTCACGAGCGCGGTCGCGAAGGCC

GGCTACACCGCCGAAGTCCGCAAGGGCGCCGCGCACGACGCCCGCCAGGAGGACGCGCACGCCGCACGGG

TCCGTGATCTGCGGCGTCGCCTCGGACAGGCGATGTTGCTGTCGGTGCCGCTGGGGAACCTGTCGATCAC

CTCGGCGTTGGTGCCGTCGCTGCGGTTCCCCTGGTGGGAGCTGCTGTGCCTCGCGCTGGCCACCCCGATC

GTCTTCTGGGCCGCGGCGCCGTTCCACCGGGCGGCGTGGCGGAACCTTCGGCACGGTTCGTCCAGTATGG

ACACTTTGGTGTCATTGGGGGTGTTCGCTTCCTACGGCTGGTCCACGTGGTCGGTGCTGACCGGCGGATC

CGGTGCGGCCGGGTACTGGCTGGGGTTCGGCACGACGGCCGCCGGCGCCGACTCGGTCTACCTCGACGTC

GCCGCCGGGGTCACGACGTTCCTGCTCGCCGGCCGCTACTTCGAGACCCGGGCGCGGCGCAGTGCGGGCG

ATCTGCTGCGGGCGCTGGACGCGCTGGCGGCCAAGGACGTCCGCGTCCTGCGCGACGGCACCGAGGTCAC

GGTTCCGGTGGCCGACCTGGCCCGCGGTGACGAGTTCGTCGTGCGACCGGGGGAGAAGGTCGCGGCGGAC

GGCGAGGTCGTCCTCGGAATGTCCACTGTGGATGTCGCTGCGGTGACCGGTGAGCCCGTTCCGGCGGAAG

TCGCCGTCGGCAGCCGGGTGATCGGCGGGAGCGTGAACCGCAGCGGCCGCCTGGTCGTGCGGGCCACCGA

GGTCGGGGCGAACACGCAGCTGGCGCAGATGAGCGCGCTGGCCGAGCGGGCCCAGGAACGCAAGGCGAAC

GTGCAGCGGCTCGTGGACCGGATCTGCGCGGTGTTCGTGCCGGTCGTGCTGGGTATCGCCGTGGTGACGC

TCACCGGCTGGCTGCTGGCCGGGCAGCCGGTGCGGGCCGGGTTCGGCGCGGCGGTGTCGGTGCTGATCAT

CGCCTGTCCGTGCGCGCTCGGACTGGCCACGCCGACGGCGTTGATGGTCGGCGTCGGCCGCGGCGCGCAG

CTGGGGATCCTCGTCAAGGGCCCGGACGCGCTGGAGGCCAGCCGCCGCGTCGACACCGTGGTCCTGGACA

AGACCGGCACCGTGACCAGCGGGCGGATGACCGTGACCGGCGTCCGGCCCGTGGGCGGGCGGACCGCCGA

GGAGGTGCTGCGCGCCGCCGCGGCGGTCGAGGCGGCGTCCGAGCACCCGATCGCCGTCGCCGTGGTCGCC

CGGATCTCCGGCGAGCTGCCCGAGGTGACGGACTTCCGGGCGCTGCCGGGGCTCGGCGCGGCCGGTGCCG

TCGACGGCGTGCGCGTGTTCGTGGGGCGGGCGCGGCTGCTCACCGAGGAGGGCGTCCCTGTTCCGCCCCA

GGTCGAGGCGGACGTCGCCGCCGCCGAGAGCGAGGGCGTCACGGTCGTGCTCGTCGCCTGCGACGGCGAG

GTGGCCGGGCTGTTGCTGGTGCGCGACGAGGTCAAGCCGTCGGCCGCCGAGGCGGTCCGGGCGCTGCACG

CGATGGGCCTGAAGACGGTGCTGCTGACCGGCGACAACGAGGTGACCGCGCGGGCGGTGGCCGCCGAGGT

CGGCATCGAGGACGTCCGCGCCGGAGTCCTGCCTGCGGAGAAGGCCGAGGTCATCGAGCAACTGCGGGCC

AGTGGCCACCGGGTGGCGATGGTCGGCGACGGCATCAACGACGCCCCGGCGCCGGCGACCGCCGACCTCG

GCCTGGGGATCGCCAGGGGCACCGACATCGCCCTGCGTGCCGCCGGCCTGATCCTCGTGCGCGACGACCT

GCGCGTGGTGCCGGACGCGCTGAGCCTGGCGCACCGCACCCTCGGTACGATCCGCGGCAACCTCGCCTGG

GCGTTCGGCTACAACCTCGCCGCCATCCCGATCGCCGCGCTCGGCCTGCTCAACCCGCTCATCGCCGGCG

CCGCCATGTCGCTGTCGTCCGTGCTCGTGGTGTCCAACAGCCTTCGCCTGCGAAACTTCGCCGGCACCGG

TATCAGTTACGAAATCGAGGAAACCCGATGA

>gi|410864597:c256570-254561 Propionibacterium acidipropionici ATCC 4875 chromosome, complete genome_80

ATGATGTCTGCTATCAGACGCCCGGGGCCGCGACTTGCCGCCGCCGGGATCCTCATCGCGGCCGCCCTGG

TGATGGGCCGCCTCGGCACCGATTGGGCACTGCCCGGCAACATCGCCATGGCTGCCGCCGCCATCATCTC

GACCATCCCGATCGCGGCGGCCGCCCTGCGGGCGCTGCGCTCCAGGACGATCGGGATCGACCTGCTGGTC

ACCATCGCCTCGATCGGGGCCCTGGCGATCGGCAACTACTGGGAGGCGGCCGCCGTCACCTTCCTCTTCG

CATTCGGCCACGTCCTGGAGCAGGGGGCCATGACGCGCACCCGATCGGCCCTCACCGATCTGGTGCGCTC

GGCCCCCCAGGACGCGGTCCTGGTGCGCGACGGACGCACCGAGACCGTGCCCACCCGCACCCTGAGGCCC

GACGACGTCGTACTGGTCGCCGCCGGCAGCTCCGTCCCGGTCGACGGGACGGTGATCTCGGGATCCGGTG

CCGCCGACGAGTCGGCGGTGACCGGGGAATCCATGCCCGCCGAGAAGACGCCGGGCGACCAGGTGCGCTC

CGGCACCACGCTCACCTCGGGGATGGTCCAGGTGCGCGCCGAGAAGGTGGGCTCCCAGACCCTGCTGGCC

CGCATCGTGGCCAGGGTCGAGGAGGCCCAGGACGCCCGCGGACGGGTGCAGACGATGATGGATCGGTTCG

GACGCTGGTACACCCCGGCCATCATCGTGCTTGCGATCGTCGTCGGGCTCATCAGCGGCAGCCTCACGCT

GGCCCTCACCCTGCTGGTGATCGGCTGCCCCGGCGCCCTCGTGATCTCGGTGCCGGTGGCCGTGGTCGCC

GGTATCGGACGCGGGGCCCGCGACGGCATCCTCATCCGCGGCGGGGAGCATCTGGAGACCGCGGCGAAGG

TCGACACCGTCGTCTTCGACAAGACCGGGACGCTGACCACCGGGCATCCCGAACTCACCGACGTCGTGGC

GTACGGGGCCGCAGACGAGGAGGAGGTGCTGCGGTGGGCGGCGCTGGCCGAGATCGGCTCCGGGCACCCG

CTGGCCGGCCCGATCCTCGACGCCGCCCGGAGCCACGGCGTCGCCCCCGAGGGAGTTCCCGGCGAGGCCG

ACGCGATCCCGGGACGCGGCCTGATCGCCGACGTGCTGGACGCCGAGCACCCGCAGACCCGCCATCGGGT

GGTCGTCGGCACAGTGGAACTGGCCGGCCAGGAGCTGGGCTCCGACAAGGAGTCCGACACCGCATGGGCG

GCCCGGACGTCCGCGCGGCTGGCCGGCGGCGGGGCCACGCCGGTCACCGTCGTCCGCGACGGCGTCCTGC

TCGGCGTGCTCGCGATCGCCGACACGATCCGGGCCGACGCCCCCGCCGCACTGGCCCGGCTTCGCCGCAC

CGGCGTCACCCGTCTGGTGATGCTGAGCGGCGACCGGCAGGAGGTCGCAAGCGCGGTGGCCGATCAGCTC

GGCATCGACGACGCCCGTGGCGGGCTGCTGCCCGAGGACAAGCTGGCTGCAGTCCAGGAGCTGCGCGCCG

GCGGAGCGGTCGTGGCCATGGTGGGCGATGGGATCAATGACGCCCCGGCCCTGGCGGCCGCCGACGTCGG

GGTGGCGATGGGGGCGGCCGGCAGCGCCCTGGCGGTGGAGACCTCCGACATCGCGCTGATGGCCGACGAC

CTCACCCGGCTGCCGGAGTCGCTGAGGCTGGCCCGCCGCACCCGCGCGGTGATCCGCCAGAACATCGTCA

TCGCCGTCGCGACGGTGACCCTGCTGCTGGCCGGGGTGCTGCTCGGAGGGGTGACGATGGCGCTGGGGAT

GCTCGTCCACGAGGCGTCTGTGCTCGTGGTCATCGTCAACGCCCTGAGACTGCTGGGACGCCGAGCCCGC

ACCCGCGAGGAGTCCCGCCAGGACCAGGCCGGCAAGCAGGATCAGCCGTGGCAGACTTCAGAGCATGGCG

GATCGAGATCGGGACGCCCAGGGGCGTCCCCGCAACAGCAGGCCGCGTGA

>gi|470176208:c3559318-3556775 Acidimicrobidae bacterium YM16-304 DNA, complete genome_81

GTGTCACCGAGCGGACGTGTTGACCTTCCCCTCGACTGGAACCCGTACGGTGATGGCATGACGACACCGA

TGACCGACCATCGAGTGACGCTCGACCTCGACGTCGAGGGGATGACGTGCGGGTCGTGCGCGGCCCGAGT

GCAGAAGACACTGGCCAAGCAACCGGGAATCGCCGACGCCGAGGTCAACTACGCCACCGGTCGGGCCCGA

ATCACCGCGGATGGCGAGATCGATCTCGACGCCTTGACGGCGGCCGTCGAACGCACCGGCTACACCCTGG

TCGTGCCGCACGGCATCGACGCTCAGCCCGCCGAGTTCGATGTCGGCGGGATGACGTGTGGGTCGTGCGC

GGCGCGTGTGCAGAAGGTCCTCCAGCGACAGCCGGGCGTCGCCGACGCCGAGGTCAACTACGCCACGGGT

CGCGCCGTCGTCACCCCGGCGGCCGGTGATCCGCTCGATGCCGCTGGGCTCGCCGCGGCGGTCCGCAAGG

CCGGCTACGACCTCGTCGCCGTCGACCGTTACGCCCCGTCGTCCGGACCGTCGGCGACGGAACGCGCCGA

CCAGGCCGACGACGACGAGGCCCGTGAGCGTCGGCTGTGGCTCCGGCGAGCGCTCATCGCCGCTCCCGTT

GCGGCATTCATGGTCTCGACCATGCTCTACCACGACCTCGCGATGGAGAACACCTGGCTGCGCTGGCTGC

AGTTTGCGCTGGCCGTGCCGGTGCAGTTCTACGTCGGCTGGCCGATCCTCGTCGGCGCAGCGCAACGGGC

ACGGCACCTCACCGCCAACATGGACACGCTCATCGCGATGGGCACGCTCTCGGCGTTCGTGTTCTCGACG

TACCAGCTGCTGACCGACGGGATGGAGCTCTACTTCGAAGCGGCCGTCGTCATCCTGTTCTTCATCACGC

TCGGCCGATACCTCGAGGCACGCGCCAAGAGCCGGGCAGGTAAAGCGCTTCGAGCGCTCGTCGAGCTCGG

GGCGAAGGAAGCGCGTCTCGTCCGTGATGGTGTGGAGGAGATGGTGCCCGTCGACACGGTTCGCGTCGGC

GACGTGGTCAAGGTGCGTCCGAGCGAGAAGGTCCCCGTCGACGGCGAGGTGGTCGACGGCGCGAGTGCGG

TCGACGAGTCGATGCTCACCGGTGAGTCGCTTCCGGTCGACAAGCGGGTCGGCGACAAGGTGGCCGGCGC

CACGGTCAACACATCTGGCGTGCTCACGATTCGCGCCACGGCGATCGGCGCCGAAACCGCGCTCGCGCAG

ATCGTCGAACTCGTCGAGAACGCCCAGACCGGCAAGTCGGCTGCGCAGCGTCTGGCCGATCGCATCTCCG

CCGTGTTCGTTCCGGTGGTGATGTCGATCTCGCTGCTGACATTCCTCGGCTGGACCGTCATCGGGGGCAG

CGTCAGCGGCGGGGTCTACGCCGCCGTCGCCGTGCTCATCATCGCCTGCCCGTGCGCGCTGGGGCTGGCG

ACGCCGATGGCGATCATGGTCGGCACCGGCCGTGGTGCCCAGCTCGGCATCTTGATCAAGTCGGTCGAGG

TGTTGGAACGAACGCGCCGGATCACCACGGTGGTGTTCGACAAGACCGGCACCCTCACCGAGGGAGCGAT

GTCGGTGACCGACATCGTCACCGCCGACGACACGACCTCGGTCGAACTGCTGGCGAAGGCGGGAGCGGTC

GAGGCCGACAGCGAGCATCCGATCGGCCAGGCGATCGCCGAGGCCGCTCGCGCCGAGCACGGCTCGCTCG

ACGCGGTGACCGCGTTCGAGACCATCGTCGGTCACGGCATCCGTGCCGACGTCGACGGCACCACGGTCTG

GGTCGGGCGTCGCGAGCTGGCGTCGCAGGCATCGCTCGTACTCCCCGACGCGCTCGACGCCGACGCGGCG

GAACTCGAAGCGCAGGGCCGAACGGCGGTGTTCGCCGGTTGGGACGGCCGGGTCCGAGGCGTGATCGCCG

TCGCCGACACGCTCAAGTCCGCAGCCGTCGACACGGTGCAGCAGCTCCACGATCTCGGATTGACCGTCGC

CATGATCACCGGCGACAACTCGCGCACTGCGCAGTCGATCGCGGACGCCGTCGGCATCGATCGGGTGATC

GCCGAAGTGCTGCCCGAAGACAAGCATCGCGAAGTGCAGCGCCTGCAGGAGTCGGGGGAGGTCGTCGCCA

TGGTCGGTGACGGGGTCAACGATGCGCCGGCCCTGGTCCAGGCCGACCTCGGTATCGCCATCGGCACGGG

CACCGATGTGGCGATCGAGTCCAGTGACCTCACGTTGCTCCGCGGCGACGTCGACGGCGTCGTCACCGCC

ATCGAGTTGTCGCGCCGCACCTACCGAACGATCGTCCAGAACCTCGGCTGGGCGTTCGGGTACAACACGA

TCGCCATTCCGCTCGCCGTGGCCGGTCTGCTCAGCCCGATGATCGCCGGTGCCGCCATGGCGTTCTCGTC

GATCAGCGTGGTCGCCAACAGCATCCGCCTTCGCCGCTTCGGCCGCACAAGGCCCAGCACCGCAACGCCC

ACCGAATCCAGGAGCACCTCATGA

>gi|336115651:4625905-4628187 Microlunatus phosphovorus NM-1, complete genome_82

ATGACCGAGTCGGCGGCTGAAGTCCAGCAGCTGGGGTCGAGCTATGAGCTGGAGATCGGCGGCATGACGT

GCGCGTCGTGCGCGAACCGGATCGAACGGAAGCTGAACAAGCTGGACGGTGTGACCGCTTCGGTCAACTA

CGCCACCGAGAAGGCGAAGGTCGAGGTCCCGGCCGGTTACGATCCGGCGCTGCTCATCACCGAGGTCGAG

AAGACCGGCTACACCGCCACCCTGCCAGCCCCCAAGAAGGTCTCGACAACCTCGGCCGGGGCGATCGGCA

CTGGCGAGGATGAGGTCGATCACGAGCTGGTGTCGCTGCGGAACCGGCTGATCGGGTCGGTCGTGCTGGC

GGTGCCCGTCATCGTGCTGGCGATGCTTCCCGCACTGCAGTTCACCTACTGGCAGTGGGCCTCACTCGCC

CTGGCCGCACCGGTGATCGTCTGGGGCGCCTGGCCGTTCCACAAGGCGGCCTGGACCAACCTGCGCCATG

GCGCAGCGACCATGGACACCTTGATCTCGCTGGGCACCACGGCGGCGTTCCTGTGGTCGCTGTATGCGCT

GTTCTTCGGCACTGCCGGCGAACCGGGCATGACCCACCCCTTCGAGTTGACCGTCGCCCCCAGCGACGGT

GCCGCCAACATCTACCTCGAGGTCGCGGCCGGGGTGACCATGTTCATCCTGGCGGGCCGCTATTTCGAGA

AGCGGTCCAAACGGCAGGCGGGGGCCGCCCTGCGGGCCCTGCTGGAACTCGGCGCCAAGGAGGTCGCCGT

CCTGCGGGGCGGCGTGGAGACGCTGATCGGAGTCGAGGACCTGAGAGTGGGCGACGAGTTCATCGTCCGG

CCCGGCGAGAAGATCGCCACCGACGGAGTGATCGTGACCGGTACGTCGGCGGTCGACGCCTCGATGCTGA

CCGGGGAGTCGGTGCCGGTCGAGGTCGGTGTCGGCGACACCGTCACCGGTGCGACGGTCAACGCCGGTGG

CCGGCTGGTGGTCCGCGCCACTCGGGTCGGCGCCGACACCCAGCTGGCGCAGATGGCGAAGCTGGTCGAG

GACGCGCAGTCGGGCAAGGCCGAGGTGCAGCGACTTGCCGACCGGATCTCCGGGGTGTTCGTGCCGATCG

TGATCGCGATTGCGGTTGGCACACTCGGCGTGTGGTTGGGTGCCGGGTTCGGCACGACGGCCGCGTTCAC

GGCCGCGGTGGCGGTGCTGATCATCGCCTGCCCCTGCGCGTTGGGTCTGGCTACGCCGACCGCGCTGCTG

GTCGGCACCGGTCGCGGCGCGCAACTCGGCATCTTGATCAAGGGCCCCGAGGTGCTGGAGTCGACCCGAA

AGATCGACACCGTCGTACTCGACAAGACCGGCACGGTGACCAGCGGCAAGATGACCCTGGTCGAGGTGCA

CACCGCCCCTGACACCGATCGAAGCGAGCTGCTACGGCTTGCCGGAGCGTTGGAGAACGCCTCCGAGCAC

CCGATTGCCAAGGCCATCGTGAAGGGGGCGGTCGAGGAGGTCGGGGCGCTGCCCACACCGGAGGACTTCG

CCAACGTCGAAGGCAAGGGTGTGCAGGGCATCGTCGACGGGCACGCCGTGCTGGTCGGCCGGGAGACGTT

GCTGGCCGAGTGGTCGCAGCAGCTGCCCGACGACCTGGCCGCGGCCAAGGCCGCCGCCGAGAGCGAGGGC

AAGACGGCGGTCGCGGTCGGCTGGGACGGTCAGGCTCGCGGGGTGCTGGTGGTCGCGGACGCCGTCAAAC

CGACCAGTACGCAGGCCGTCGCCCAGCTGAAGGGCCTGGGGCTGACCCCGATCCTGCTGACCGGTGACAA

CGAGACGGTGGCTCGGCAGATCGCCGCCGAGGTCGGCATCGACCGGGTCATCGCCGAGGTGCTGCCCGCA

GGCAAGGTCGAGGTCGTGTCCCGGCTGCAGGCTGAGGGCAAGGTCGTGGCCATGGTCGGCGACGGCGTCA

ACGACGCCCCCGCCCTGGCCCAAGCCGATCTCGGTCTGGCGATGGGCAGCGGCACCGATGTGGCGATCGA

GGCCTCCGACATCACCCTGGTCCGCGGTGATCTGCGCGCGGCCGCCGACGCGATTCGGCTGTCTCGCAGG

ACCCTGGGCACCATCAAGACCAATCTGTTCTGGGCGTTCGCGTACAACGTCGCCGCGATCCCGCTCGCCG

CACTCGGTCTGCTGAACCCGATGCTGGCCGGTGCCGCGATGGCGTTCTCCAGCGTCTTCGTGGTCGGCAA

CAGCCTGCGACTGCGCAGCTTCCGGTCCAACGCCACGAACTGA

>gi|182433793:c5679711-5677288 Streptomyces griseus subsp. griseus NBRC 13350 chromosome, complete genome_83

ATGGCCAGCACAGCAGCAGCCGACTTCCCCACCGCCGAGGCGTCCGAGGCCGAGCTCATGATCGGCGGGA

TGACCTGCGCCTCGTGCGCCGCCCGCGTCGAGAAGAAGCTCAACCGGATGGACGGCGTCACCGCCACGGT

GAACTACGCGACCGAGAAGGCCCGCGTCACCTTCGGGGAAGGCCTGGAGCTGGGGGACCTCGTCGCCACG

GTCGAGAAGACCGGCTACACGGCCCGCCCCGTCGCCCGGCCGGAGCGGAGGGCGCCGACGCCGTCCGAGG

CCACCGCTCCCCCGGCCGCCGCCCTCCCGAGCCCCGCTTCCCCGGCCGCCGTCCCGGCCCCGTACGCCGG

CGAGAGCCGCCGCACGGACCCCGCCACGCCCCTCGACGCCACCCCCGCCGCGGGACCCGGGCCGGACCCG

CGCTCAGCGGACCGGGACGCCGGGGCGGAGCAGGACGCCGCCATCGGCGCGCTCCGCCGGCGCCTGGTCG

TCTCGGCCGTGCTGGCACTGCCCGTGGTCCTGCTCGCCATGGTCCCGGCCCTCCAGTTCGACTCCTGGCA

GTGGCTGAGCCTCGTCCTCGCCGCGCCGGTCGTCGTCTGGGGCGGCCTGCCCTTCCACCGCGCCGCCTGG

ACCAATCTCCGGCACGGTGCGGCCACGATGGACACACTGGTCTCGCTCGGCACGCTCGCCGCGTTCGGCT

GGTCGCTGTGGGCCCTCTTCTTCGGCGACGCGGGCATGCCCGGCATGCGCCACGGCTTCGACCTCACCGT

CGCGCGGGCCGACGCCGGCTCGACGATCTACCTGGAGGTGGCGGCCGGGGTCGTCACCTTCATCCTGCTC

GGCCGCTACCTGGAGGCCCGCGCCAAGCGGAGGTCGGGGGCGGCGCTGCGGGCGCTGATGGGCCTGGGAG

CCAAGGACGTCGCCGTGCTCAGGGGCGGCCGGGAGGTCCGCGTCCCGGCGAGCACGCTCGTGGTCGGGGA

CCGTTTCGTGGTCCGGCCCGGGGAGAAGATCGCGACGGACGGGACCGTCGTCGAGGGCTCCTCCGCCGTG

GACGCCTCCATGCTCACCGGCGAGTCCGTCCCCGTCGAGGTCACCACCGGCGACACCGTCACCGGAGCCA

CCCTCAACGCCGGCGGACGCCTCGTCGTCGAAGCCACCCGCATCGGCGCCGACACCCAACTCGCCCGCAT

GGCCAAACTCGTCGAGGACGCCCAGAACGGCAAGGCCTCCGCACAACGCCTCGCCGACCGCATCTCCGCC

GTCTTCGTCCCCATCGTCATCGCCCTCGCCCTCGCCACCCTCGGCTTCTGGCTCGGCAACGACGCCGGAC

TCACCGCCGCCTTCACCGCCGCCGTCGCCGTCCTCATCATCGCCTGCCCCTGCGCCCTCGGCCTCGCCAC

CCCCACCGCCCTCATGGTCGGCACCGGCCGCGGCGCCCAGCTCGGCATCCTCATCAAGGGCCCCGAAGTC

CTGGAGACCACCCGCCGCGCCGACACCATCGTCCTCGACAAGACCGGCACCGTCACCACCGGCCGCATGA

CCCTCCAGACCACCCACACCACCCACACCACCGACACCACCGAAGTCCTCCGCCTCGCAGGAGCACTGGA

GAACGCCTCCGAACACCCCATCGCCCAAGCCGTCGCCACCGCCGCAGCCGCCACCACCGGCCCCCTCCCC

ACCCCCGAGGACTTCACCAACGTCCCCGGACTCGGCGTCCAGGGCACCGTCAAAGGCCACGCCGTCCTCG

TCGGACGGCCCCGGCTGCTCGCCGACGCGGCGATCCCCCTCCCGCCGGTGCTGGAGGCCGCCCTGGCGGA

GGCCGAGGAGCTCGGCCGTACGGCGGTCGTCGCGGCCTGGGACGGGGAGGCCCGGGGCGTGTTCGCGGTG

GCGGACGCGGTCAAGGACAGCAGCGCGGCGGCCGTGCGCGAGCTGCGCGCCCTGGGGCTGAGGCCCGTGC

TGCTGACCGGCGACAACCGGGCCGTGGCGGAGGCCGTGGCCCGCGAGGTGGGCATCGACGAGGTCCACGC

GGAGGTGCTCCCCGAGGACAAGGTGAACGTCGTGAGGCGGCTTCAGGCCGAGGGCCGGGTCGTGGCCATG

GTCGGCGACGGGGTCAACGACGCGGCCGCCCTCGCCGCGGCGGACCTGGGGCTGGCGATGGGCACCGGGA

CGGATGCGGCGATCGAGGCGAGCGATCTCACACTGGTTCGTGGAGATCTCAAGGTGACGGCTGACGCAAT

CCGCCTCTCCAGGCGTACGCTGGCCACCATCAGGGGCAACCTCTTCTGGGCCTTCGGGTACAACGTCGCG

GCCTTGCCCCTGGCTGCAAGTGGCCTGCTCAACCCTATGATCGCGGGAGCCGCCATGGCGTTTTCGTCCG

TGTTCGTCGTCACGAACAGTCTACGGTTGCGTGCCTTCACGTAA

>gi|379733551:3228347-3230677 Blastococcus saxobsidens DD2, complete genome_84

ATGACCACCGCACCCGCAGGCACCACCACCGACGTCGAGCTGCTCATCGGCGGGATGACCTGCGCCTCCT

GCGCCGCCCGGGTGGAGAAGAAGCTCAACAAGCTCGACGGCGTCACCGCCACCGTCAACTACGCGACCGA

GAAGGCGAAGGTCAGCTACGGCGACGGCGTGAACACCGGGGACCTGATCGCCACGGTCGAGAAGACCGGC

TACTCCGCGCGCCTCCCCGAGCCCCCGCGGCCGGAGATCCCCGCGGAGGCCGAGGCCGACCCCGCCGACG

CCCCGGTGCACGCGCTGCGGACCCGCCTCGTGGTCTCCACCCTGCTGAGCGTCCCGGTGATCCTCATGGC

GATGGTGCCGGCGCTGCAGTTCCAGTACTGGCAGTGGCTGTCGCTGACCCTCGCCGCCCCCGTGGTGGTG

TGGGGCGGCGCGCCGTTCCACCGGGCGGCCTGGACCAACCTGCGCCACGGCGCGGCGACCATGGACACCC

TGGTGTCGCTGGGCACGCTGGCGGCCTTCGGCTGGTCGCTGTACGCGCTGTTCTGGGGCACCGCCGGCGT

CCCCGGCATGACGCACCCGTTCGAGCTGACCATCGCCCGCACCGACGGCAGCGCCAACATCTACCTCGAG

GCCGCGGCCGGGGTGACGACGTTCATCCTGGCCGGCCGCTGGTTCGAGGCGCGGTCCAAGCGCCGGGCCG

GCGCGGCGCTGCGCGCGCTGCTGGAGCTGGGCGCCAAGGAGGTCTCGGTGCTGCGCGGCGGCACCGAGCA
GCGGGTCCCGGTCGGCGAGCTCGGCGTGGGCGAGCTGTTCGTCGTCCGGCCCGGCGAGCGGATCGCCGCC
GACGGCGAGGTGACCGAGGGCTCGTCCGCCGTCGACGCCTCGATGCTCACCGGCGAGTCGGTGCCGGTCG
AGGTCCGCCCCGGCGACCCGGTCACCGGCGGCACGGTCAACGCCGGCGGCCGGCTGGTCGTCCGCGCCAC
CCGCGTGGGCAGCGAGACCCAGCTCGCCCAGATGGCCCGGCTGGTGGAGGACGCGCAGAACGGCAAGGCC
GAGGTGCAGCGGCTCGCCGACCGGATCTCCGGGATCTTCGTGCCGGTCGTGCTCGCGCTGACCGTGGCGA
CCCTCGGCTTCTGGATCGGCACCGGCGCCGGCCTGCCGGCCGCCTTCACCGCCGCGGTGGCCGTGCTGAT
CATCGCCTGCCCCTGCGCGCTCGGGCTGGCCACGCCCACCGCGCTCATGGTGGGCACCGGCCGCGGCGCG
CAGCTCGGCATCCTGATCAAGGGGCCGGAGGTGCTCGAGTCGACCCGACGGGTGGACACGGTGGTCCTGG
ACAAGACCGGCACGGTGACCACCGGGCGCATGACGCTGCTGGACGTCGTCCCCGCCGCCGGGGAGGACGC
CGACCGGGTCCTCCGCCTGGCCGGCGCCCTCGAGGCGGCCTCGGAGCACCCGATCGCCCAGGCCGTGGCC
CGCGCCGCGACCGAGCGCACCGGTGACCTTCCCGCGGTGAGCGACTTCGCCAACGTCGAGGGCCTCGGCG
TGCACGGGGTGGTGGAGGGCTCGGCGGTGGTGGTCGGACGACGGCGTCTGCTGGCCGACTGGTCGCTGGC
CCTTCCCGCCGACCTGGAACGGGCCGTCGCCGAGGCCGAGGACGCCGGCCGGACCGCGATCGCGGTCGGC
TGGGACGGCGCGGCCCGCGGGGTGCTCGTCGTCGCCGACGCGGTCAAGCCGACCTCTGCGACGGCCGTCG
CCCAGCTGCGCGCCCTCGGGCTGACCCCGATCCTGCTCACCGGCGACAACGAGAAGGCCGCCCGCGCCGT
CGCCGCCCAGGTGGGCATCGACGAGGTGATCGCCGAGGTGCTCCCGCAGGACAAGGTGGCCGTCGTGCGG
CGGCTGCAGGACGAGGGCCGCACCGTGGCCATGGTGGGCGACGGCGTGAACGACGCCCCGGCGCTGGCCC
AGGCCGACCTGGGCCTGGCGATGGGGACCGGCACCGACGTCGCCATCCAGGCCAGCGACCTGACCCTGGT
GCGTGGCGACCTCCGGGTGGCCGCGGACGCGATCCGGCTGTCCCGCCGGACGCTGGGCACGATCAAGGGC
AACCTGTTCTGGGCCTTCGCCTACAACGTGGCCGCGCTGCCGCTGGCCGCCGCGGGCCTGCTGAACCCGA
TGATCGCCGGTGCCGCCATGGCGTTCAGCTCGGTGTTCGTCGTCTCCAACAGCCTGCGGCTGCGGGGCTT
CGCACCACTGCCGGAGAACCGCGACGCGACCCTGGCTGTCGCGGGTGGGCACCCGGCCGACGAGCAGGTG
GCGGCGCCGAGCCGGAGCTGA

>gi|506928554:c5344537-5342798 Amycolatopsis orientalis HCCB10007, complete genome_85

ATGGACACCCTGGTCTCGCTGGGTGTGCTTTCGTCGTTCGCGTGGTCGGCTTGGTCCGCCTTCGCCGGCG
GCACCGAACCCGGCTACTGGATCGGCTTCGGCCCGACGGCGGCCGGTGCCGACGCCATCTATCTCGACGT

GGCCGCCGGGGTGACCACCTTCCTGCTGGCCGGGCGGTACTTCGAGAGCCGGTCCCGGCGCAGCGCCGCC

GGACTGCTCGCCGCGCTGGACGCCTTGGCCGCCAAGGACGTCCGGGTGCTGCGGGACGGAGCCGAGCGGA

TGGTCCCGATCGGCGAACTGGCCGTCGGCGACCTGTTCGTGGTCAAGCCGGGGGAGTCCATCGCCGCCGA

CGGCGTCGTCCACAGCGGACTGTCCACTGTGGATGTCAGCGCGGTGACCGGTGAACCCGTGCCCGCGGAG

GTCTCCACCGGGGATCGGGTCATCGGTGCTTCGGTGAACCGGGACGGCAGGCTCGTCGTCCGCGCGACCG

CGGTCGGCACGCATACCCAGCTGGCCCAGATGACCGCGCTCGCGGAACGCGCGCAGGCCCGCAAGGCCGC

GGTGCAACGTCTGGTCGACCGGATCTGCGCGGTGTTCGTCCCCGCCGTGCTCGGCGTGGCCGCGCTGACG

CTCGCGGTCTGGCTGCTCGGCGGGGCACCCGCGCGCGAAGGGTTCGCGGCGGCCGTCGCCGTCCTCATCA

TCGCCTGCCCGTGCGCGCTCGGGCTCGCGACGCCGACCGCGCTGATGGCCGGGGTCGGCCGCGGCGCCCA

GCTCGGCATCCTCATCAAGGGGCCGGACGCGCTCGAGGCGAGCCGCACGGTGGACACCGTCGTACTGGAC

AAGACCGGCACCGTCACCACCGGGAAGATGACCGTCACCGCGTGCCGCCCCGCCGACGGGTTCACCACCG

GCGAGCTGCTGCGGTTCGCCGCGGCCGTGGAGGTCGGTTCGGAGCACGCCATCGCGGCGGCCGTGGTGAC

CGCGGCGCGGGCCGAACTGCCGGACCTGCCCCCGGTCGAACGCTTCGCCGCGCTGCCCGGGCTCGGCGCG

CAGGGCGAGGTCGAGGGGCACGACGTCCTCGTCGGCAACGCGCGGTTGTTCGCCGATCGAGGGATCCCCT

GTGACTCCGCGGCGGCGGACGGCGCGACGGCGATCCTCGTCGCGGTGGACGGCCGGGTCGCCGGGCACCT

GGAAGTCCGGGACGTCGTGAAGCCGTCGGCACCGGCCGCCGTCGAGGCCCTGCACGCGCTCGGCCTGCGG

ACCGTGCTCCTGACCGGTGACAACGAAGCCGCCGCGCGTGTGGTCGCGGAGGAGATCGGCGTCTCCGACG

TGCGCGCCGGGGTGCTGCCGGCGGAGAAGGCCGCGGTCATCGAGGAACTGCGGGCCGGGGGAGCGCGGGT

CGCGATGGTCGGCGACGGGATCAACGACGGTCCGGCGCTGGCCACCGCGGATCTCGGCATGGCGATGGCG

CGGGGCAGCGACATCGCGGTCCGCTCCGCCGACATGGTGCTGGTCCGCGAAGACCTGCGCGTGGTCCCCG

ACGCCATCCGGCTCGCCGACCGCACGCTGCGGATCATCCGGGGGAACTTGGCCTGGGCGTTCGGCTACAA

CGTCGCCGCGATCCCGCTGGCCGCGCTCGGCCTGCTGAACCCGCTGATCGCCGGGGCGGCGATGTCGCTG

TCCTCGGTGCTCGTCGTGTCCAACAGCCTCCGGCTCAAGGACTTCCGACGCGCCGCGTGA

>gi|357409266:c5291699-5290122 Streptomyces pratensis ATCC 33331, complete genome_86

ATGTCTGAAATGCACACTCGCCGCGCCGTGCTCGGCGCGGGTGTCGCCCTGGCCGGAGCAGCGGGACTCG

CCTCGCAGGCCGTCACCCCCTCCCGGGCCCACGCCGCTCCCCGCCGCACGCCCGCCCCCTCCCCCGCGCG

AGCCGCCGACCAGTACGTCACGCCCGACGGTCCCGAGGTGGTGGACGCCGAGGCCCGCCGGGGTGCCGGC

CCGGTGCGGAAGGTCTCGCTGACCGCCACCTCCTCGCGCCTGGAGCTGGGCGGCGGCCTCACCGTCCCCA

GCTGGGCCTACGGCGACCGCCTGCCGGGTGAGGCGATCCGGGTCACCGCGGGCGAGATCCTCGAACTCGC

CCTGGCCAACAACCTGCCGCAGGCCACCACGATGCACTGGCACGGTCTCGCCCTGCGCAACGACATGGAC

GGCGTGCCGGCGCTGACGCAGCAGGCGGTGAAGGCGGGCGCCGACTTCACCTACCGGTTCGCGGTCAGCC

ACCCCGGGACCTACTGGATCCACCCGCACTCGGGCGTGCAGCTCGACCGGGGCCTGTACGCGCCGCTGAT

CGTCGAGGACCCGAAGGAACCGCTCGCGTACGACAAGGAGTGGGTCGTCGTCCTCGACGACTGGCTCGAC

GGGGTGGACGGCTCCACCCCCGACGCGGTCCTCGACGAGCTCACCGAGGGCCGGGGGTCCGCCCATGACG

CCACCGGCCACCAGGCGGCCCGCCGCGCGGCCGACGGCGACGCCCCCTCGCGGCTCCTCGTCGGCGCGAG

CAGCGATCTGCTCGGCGGCCACGCCGGCGACGTCGCCTATCCGCACTACCTGGTCAACGGCCGCAGGGCG

AGCGACCCTTCGGTCTTCGAGGCGGCTCCCGGCGACCGCATCCGGCTGCGCGTCGTCAACGCCGGCGGCG

ACACCGCGTTCCGGCTGGCGCTCGGGGGCCACCGGATGACGGTGACGCACACGGACGGCTTCCCGGTGCG

GCACACCGAGACCGACACCCTGCTGCTGGCGATGGGTGAGCGGTACGACGTGCTGGTGACCGCGGGGGAC

GGGGTGTTCCCGCTGACGGCGCTCGCCGAGGGCAAGGAGGCGTCGGCGCTGGCCGTGCTGAGGACGGGGC

CGGGAGCGATCCCGCCTGCCACGGTGCGGCCCGCGGAGCTGGACGGCGAGCTGGTCACGGCCGACCGGCT

GGCCCCGGACGAGGCGGTGGCACTGCCGGAGCGCGCACCCGACCGGACCGTGCGCATGACGCTCACGGGT

TCCATGGACGCCTACGACTGGGCCTTCGACGGCAAGCCCTACGACCCGGCGGTCCGGCATCCCGTCAAGG

CCGGGGAGCGCGTGCGACTGGTCTTCACCAACCGCACCGCGATGTGGCATCCGATCCATCTGCACGGCCA

CACGTTCGCGATGGCCGGCACGGCGCTCGGCGCCCGCAAGGACACCGCCGCGGTCCTGCCCGGACGCTCG

CTGACGGTCGACTTCGACGCGGACAACCCCGGCCTGTGGATGCTCCACTGCCACAACGTCTACCACGCCG

AATCCGGGATGATGACGGTCCTGGGCTACCTGCGCTGA

>gi|300781937:c7214944-7212716 Amycolatopsis mediterranei U32 chromosome, complete genome_87

GTGATCGGCGGCATGACCTGCGGGTCGTGCGCCGCGCGGATCGAACGCAAGCTCAACCGGCTCGACGGCG

TCGAGGCCAGCGTCAACTACGCCACGGAGAAGGCCCACGTGCGGGTCCCGCCGTCGATGACGACCGACGA

CGTCATCCGCGCGGTGGAGGCGGCCGGCTACACGGCCGCGCCACCCGCACCACCGCGGTCCGCGGAGACG

GCCTCCGAGCCGTTGCCGTCCGACGCGCTGCGCCGCCGCTTGCAGCTGTCAGCGCTGTTCGGTGCCCCGG

TGATCCTGGCGGCCATGGTGCCGGTGCTGCAGTTCCCCGCCTGGCAGTGGGTTTCCCTGGTCCTGGCCAC

GCCCGTGGTGGTGTGGGGCGCGTGGCCCTTGCACCGCGCCGCCTGGACCAACCTCCGCCACGCCACCGCG

ACCATGGACACCCTGGTGTCGATGGGCGTGGCCGCGGCCTACCTCTGGTCGCTCTACGCGTTGTTCTTCG

GCGGGGCCGGGGTCATCGGCATGCGACACGAGTTCGAGTTCACCCTCTACGCCGACCACAACGTCGACGG

GATCTACTTCGAGGTCGCCACCGGCGTCACGCTGTTCATCCTGGCCGGCCGCTACTTCGAGTCCCGGTCG

CGCCGCCGGGCCGGTGCGGCGTTGCGGGCACTGCTCGACCTCGGCGCGAAGTCCGTCGCCGTGCTTCGGG

ACGGCCGCGAAGAGCGGATCCCGATCGACCAGCTGAGGACCGGCGACCTGTTCGTGGTCCGGCCGGGGGA

GAAGATCGCCACCGACGGCGTGGTCGTCGACGGCGCCTCCGCGGTCGACCTCAGCCTGGTCACCGGGGAA

GCCGTGCCGGTCGAAGCCGGTGTCGGCGCCGAGGTGGTCGGCGCGACGGTCAGCATGAGCGGGCGGCTCG

TCGTGCGCGCGACCCGCGTCGGCACCGAGACCCGGCTGGCGCGCATCGCGGCGATGGTCGAACGCGCCCA

GAGCGGCAAGGCCGATGTGCAGCGGCTCGCCGACCGCGTGTCCGCGGTGTTCGTGCCGATCGTGATAGCG

CTTTCCGTGGTGACACTGGGGTTCTGGCTCTTCTCGGGCGCGGGGGCCGGGATGGCGTTCACCGCCGCGG

TCGCCGTGCTGATCATCGCGTGCCCGTGTGCTTTGGGGCTGGCCACACCGACCGCCCTGCTGGTGGGCAC

CGGCCGCGGCGCCCAGCTCGGTGTGCTGGTCAAAGGACCGGAGATGCTGGAGTCGACGCGGCGGGTCGAC

ACCGTCGTGCTCGACAAGACCGGCACGGTGACCACCGGGAAGATGACGCTGGCCGGGGTCAAGACCGTCG

ACGGCGTGCCCCCGGACCTGGCGTTGCAGCTGGCCGGGGCGCTGGAAAGCGCGTCCGAGCACCCGATCGC

GGTCGCGGTCACCGACGCGGCGCGCCGCCGCTTCGGCGACGTCGGGCAGGTGCGGGAGTTCCGGGCCGTC

GCGGGCTACGGCGTGCTCGGCCGGGTGGACGGCCACCGCGTCGTCGTCGGCCGGCCCGAACTGCTGCAGA

AGATGTCGGTCCGGGTGCCGTCGGCGCTGTTCAAGGTGAAGCAGCAGGCCGAACGGGCCGGGAACACCGC

GGTCCTCCTGGCTTGGGACGGTTTCGCGCACGCGGTGCTGGTCGTGGCCGACACGGTGAAGCCGACGTCG

GCTCCGGCGGTCTTCCAGCTGCGCCGGCTCGGCCTCACCCCGGTGCTGCTCACGGGCGACAACGAAGCCG

TGGCCCAGTCGATCGGGCAAGAGGCCGGGATCACCGAAGTGATCGCCGGGGTCTCACCGGAACAGAAGGT

CGAGGTGATCGCGGATCTGAAGGCCCAGGGCCGGGTGGTGGCGATGATCGGCGACGGCGTGAACGACGCA

GCCGCGCTGGCCACCGCGGATCTGGGGCTGGCGATCGGCACCGGCACGGACGCGGCGATCGAGGCGGCGG

ACCTGACGCTGGTCCGCGGCGATCTGATGGCCGCGGTCGACGCGATCCGGTTGTCGCGCCGGACGTTGGC

GACGATCAAGGCGAACCTGTTCTGGGCGTTGGCCTACAACGTGGCCGCGTTGCCGCTGGCGGCGGCGGGG

CTGCTGAACCCGATGATCGCCGGGGCGGCGATGGCGGCGTCGAGCTGCCTCGTGGTCTCGAACAGCCTGC

GCCTCACCAGGTTTCGGGGAACAGCCGCGGCGCAGCCACTGCTCGAACCCCGCTCGTGA

>gi|473832060:1026665-1029094 Clavibacter michiganensis subsp. nebraskensis NCPPB 2581 complete genome_88

GTGACCGACACCGCATCCGGATCCGCGCCCGTCGCGGGCCCCGTCGCCGCCCTGCCCGAGGAGGGCGTCG

TGCTCACGCGCGTCGACCTCGACGTGCAGGGCATGACGTGCGCGTCGTGCGCGATGCGGATCGAGCGGAA

GCTCGGCCGGATGCCCGGGGTCGAGGCGTCCGTCAACTACGCCACCCATCGGGCGCGCGTGCAGCTGCCC

GCGGGCACGAGCGTCGAGGACGCGATCCGCACCATCGAGCGGACGGGCTACCGCGCGTCCGAGCGCGCCG

GGTGGGGGAGCGGGGCGGGAGATGACGCGTCCGCGCCCGTCGCGTCCCGACCGGGGGAGCCCGCGCCCGT

AGCGGTCGCGGCTCCCCGCGTCCGACCCGACGCGCCCGACGCCGGCGGTTCGTATCCCGCATCCGCGCCG

TCGCCCACGCCCGGCCCGGCAGCGCCCGCCGCCCGCCGCCCCGACGCGGACGAGCTCGCGCTCCGGCAGC

GGCTCCTGGTCTCCGCGGTCCTCACCGTCCCCGTCTTCCTCATGGCGATGATCCCCGCGCTCCAGTTCGA

CGACTGGCAGTGGCTGTCCCTCGCGCTCGCCGCGCCCGTCGCCGTCTGGGGCGCGTGGCCGTTCCACCGG

TCCGCCGCCGTCAGCGCGCGCCACGGCGGCGTCGGCATGGACACGCTCGTCAGCATCGGCGTCGCGGCCG

CCTTCCTCTGGTCCCTCTACGCGCTCTTCCTCGGCGACGCGGGCGAGCCCGGCATGCGCATGACCATGAG

CCTCGTCGGCGAGCCCGGCGGCGGATCCGGCGACGTGTACCTCGAGGTCGCCTCGGCCGTCACGGTGTTC

CTCCTCGGCGGCCGCTACCTGGAGGCCCGGGCCGCGCGCGCGTCCGGCGCCGCCCTCGCCGCGCTCCTCG

ACCTCGCCGCCAAGGACGTGGCCGTGGTCCGCGACGGCGTGGAGACGCGGATCCCCATCCGCGACCTGCG

CGTCGGCGCGGAGTTCGTCGTGCGGCCGGGCGAGCGCATCGCCACCGACGGCGTGGTCGTCGACGGGTCC

AGCGCCGTCGACCGCTCCCTGCTCACCGGCGAGTCCCTTCCCGTGGAGGTCGGCCCCGGCGACGACGTCA

CGGGCGCGACCCTCAGCGCCGGCGGACGCCTCGTCGTGCGCGCCACGCGCGTGGGCGAGGAGACCCGGCT

CGCCCGCATGGCCGCGCTCGTGGAGGAGGCGCAGACCGGGAAGGCCCGGATCCAGCGCCTCGCCGACCGC

GTCTCCGCCGTCTTCGTGCCCGTCGTGCTCGTGCTCGCCGTCGGTACGCTCGTCGGCTGGCTGCTGCTCG

GCTTCCCCGCGGAGGCCGCCTTCACCGCCGCCGTGGCGACGCTCATCATCGCCTGCCCGTGCGCCCTCGG

GCTCGCGACGCCGACCGCGCTCCTCGTGGGCACGGGCCGCGGCGCGCAGCTCGGGATCCTCATCACCGGC

CCCGAGGTGCTCGAGTCCACCCGCCGGATCGACACCGTGCTGCTCGACAAGACCGGCACGGTCACGACGG

GCGTCATGTCGCTCGTGTCCGCCGTCCCCGCGGCGGGCGTCGACTCCGACGAGCTCGTGCGCGTCGCCGC

CGCCCTCGAGGCGCGGTCGGAGCACCCGGTCGCGCGCGCCGTCGTCGAGGCCGCGGGCGGCGGATCCCTC

CCTCCCGTGGAGGGCTTCGTCGCCACCGCGGGCCTCGGCGTCCACGGTGTCGTCGACGGACGCGCGGTCG

CGGTCGGGCGCCCCTCGTGGCTCGCGGAGCAGTGGGCCGCCGAGCCCGACGCGGCGCTCGCCCAGGCGTC

GCGGGACGCCGAGGCGGATGGATCCACCGTCGTCGCGGTCGCGTGGGACGGCGCCGTCCGCGGGACCCTC

GCCGTCGCCGACACCGTCAAGCCCACGAGCGCCGAGGCCGTCCGCCGCATGCGTGCGCTGGGCCTCCGTC

CCGTCCTCCTCACGGGCGACGCGGCGGGCGCCGCGCACCGCGTCGCCGCGGAGGTCGGCATCGACGAGGT

CATCGCGGGCGTGCTCCCCGAGGGCAAGCTCGACGCCGTGCGTCGCCTGCAGGCCGAGGGCCGCGTGGTC

GCGATGGTCGGCGACGGCGTGAACGACGCCGCCGCCCTCGCGCAGGCCGACCTCGGGATCGCGATGGGCA

CGGGCACCGACGCGGCCATCGAGGCGGGCGACATCACGATCGTCCGCGGCGACCTCGTGCTCGTGGCCGA

CGCCGTGCGCCTCGCGCGGCGCACGCTCGGCACGATCCGCGGCAACCTGTTCTGGGCCTTCGCCTACAAC

GCGGCCGCGATCCCCGTGGCGATGCTCGGCCTCCTCAACCCGCTCGTCGCGGGCCTGGCCATGGCGCTCT

CGTCGGTGTTCGTCGTCACGAACAGCCTGCGCCTGCGCTCCTTCCGCTAG

>gi|239916571:c1982520-1980955 Micrococcus luteus NCTC 2665 chromosome, complete genome_89

ATGACCTCCTCACTGACCACCCGCCGCACCTTCCTGGGCGCCTCCGTCGGGGCCCTGGCGCTGGCCGGGC

TGGCCGCCTGCGCCGATCCCGCCATCAGCCCCCAACCGTCAGGGCGCATCCTGCCCACCGACCCGCTGGT

CGCCGAGTACGAGAAACGACGCGCCGCCTCGGGCACCACCGTCACCCACCGGCTCTCCGCCGCACCCCTC

GAGACCTCCCTCCAGGGTCGGGGCCTGACCACCTGGGGATACAACGACGGCCTCAACGGGCCGCTGCTGC

GCGCCCAGACCGGGGACCTGCTCAAGGTCAGCGTCGCCAACGACCTGCCCGAGGACACGAGCGTGCACTG

GCACGGGCTGGCCCTGCGCAACGACGCCGACGGAGTCCAAGGAGTGACCCAGGACCCCATCGCCGCCGGA

GAAACCTATACGTACGACTTCCGGCTGTCCCATCCGGGCACGTACTGGTACCACTCCCACTTCGACACCC

AGCGCGAACGCGCCCTGTACGGGGCCCTGATCGTCGAGGACCCCAACGAGCCCCTCAGCTACGACCAGGA

ATGGGTCGTGATCCTGGACGACTGGCTCGACGGGATCACCGGAACACCGCAGGACGTCGTCGAGGAACTC

TCCGCCGGGATGGACATGTCCGGCGACATGGAGGGCATGGCCGGCATGGCGGGCATGGACCACGGGTCCA

TGAGCCAGGACAGCTCCTCGGGCATGCCCATGAAGCACATGCTCATGGGCGCTGAGAGCGACTATCTGGG

CGGGGACGCCGGCGACGTGAAGATCCCCGTGCACCTGTTCAACGGCCGCCCCCCATCCGACCCGGACACC

TTCTCCAGCACCCCGGGCAACCGGATCCGCCTGCGCTTGATCAACGCGGCCGGAGACACCGCTTACCGGG

TCGGGATCCCCGGCCAGGAACTGACCATCACCCACACCGACGGGTTCCCGGTCCAGCCCCGCCAGGCCGA

TGCCGTCGTCCTGGGGATGGGCGAACGCCTGGACGTGCTGATCACCCTCGGCGACCAGGCGGTGCCCGTA

CTGGCCCTGCCCGAGGGCAAGACCGGCCACGCCTACGGCATCATCGCAGCCGGTCCAAAGACCACGCTCT

CCGGTCGGCTCCCGCAGACCCTGGGCGGAACCGTGCTCGATGGCAGCCGACTGAAGGCACACGAATCCGT

CCTGCTCCCGCCCAAGGACCCGACCAGGACCCACGAGGTACGCCTCACCGGGTCGATGATGGAGTACGAC

TGGGGCATCAACGGGCGACGCTTCGACCCGGCCAACCCCTTCGAGGGCGCCTTCGAACTCCGGTTGGACG

AACGCGTGCGGGTCACGATGACCAACGACACCGACATGTGGCACCCCATGCACCTGCACGGCCACACCTT

CCAGCTGGCCGACCATGGCGCCCGCAAGGACACGGTCATCATCAAACCCCGGCAAACCATCGTCTTCGAC

TTCGAGGCGGACAACCCGGGCCAGTGGCTAACCCACTGCCACAACGCCTACCACGCCGAGGCCGGGATGA

TGGGCGTCTTCTCCTACATCCACTGA

>gi|403525101:4634133-4636562 Arthrobacter sp. Rue61a chromosome, complete genome_90

TTGAGTAGCCAGGAGACTTTCAACCAACCCGCACACAGGGTCATCGAGCTGGACATCGAGGGCATGACCT

GCGCCTCGTGTGTCAATCGAGTGGAACGAAAACTGGGAAAACTCGAGGGTGTAGAGGCCAGCGTCAACCT

GCCCCTCGAATCAGCCCACGTCACAGTCCCAGCATCAGTGACGGATCAGCAGATCGTGGACACCGTCAAT

GCCACCGGCTACAAAGCCACCGTCCGCCAAGCCCCGGCGGCCCACGGACACACCTCCACCAAACAAACCC

ACGATCGTGGCGCCGCTGTAACGATGCCACGCGGCGAAGCCGGCGGCGTGCGGCAGCATGCGTCTAAGGG

AGGCACGACCGAGCATGCAGAGCACGGCGCCGACTTCGCCGCCCAAGCCAACCACGGCGAGCACGGCGAG

CACGCTACACACGAAGACCACATGGCTCACGGCCCAGCAGCCTCTACACTGCGCCCCCGCCTGATCGTCG

CGGCCCTCCTGACCATCCCCGTGTTCGCAATCTCCATGATCCCCGCGTTGCAGTTCGCCAACTGGGGATG

GGTTGTAGGTGCGCTTGCCCTACCAGTGGTGAGCTGGGCGGCCTGGCCCTTCCACCGGGCAGCGGCCATC

AACGCACGGCACTTCGCTTCCACCATGGACACGCTCGTTTCCATTGGCGTCATCGCCGCCTACCTCTATT

CGGCGTGGCAACTCTTTGTGGATCCCCGGATGACCGAACACCCCGGCATGGAAAACATGTCCGGTGGTGG

CCTCTACTTTGAGGTCGCTGCGGTGGTCACAACGTTCCTGCTTCTGGGTCGCTACCTTGAAGCCAACGCC

AAGGCCAAAGCGGGCAATGCCCTCAAGGCACTGCTGAATCTGGGCGCCAAGGATGCAACCATCCTGGTGG

ATGGCGTCGAGCAGAAGATTCCCGCGGATCAGCTCCTCGTGGACGACGTCATCGTTGTCCGCCCGGGCGA

AAAGATCGCTACCGACGGCGTGGTCACGGACGGTGCATCCGCCGTCGACGCCTCACTTGTCACGGGCGAA

TCGGTGCCGGTGGAGGTTGGACCGGGCAGCTTGGTGACGGGCGCTACCATCAACACTTCCGGCCGGCTGC

TGGTCCGGGCCACCCGCGTGGGCTCCGACACTACGCTCGCCCAGATGGGGCGTCTGGTCAGTCAGGCGCA

GACGGGCAAGGCGCCCATTGCGCGGCTCGCTGACAGGATCAGCTCTGTCTTCGTCCCGATTGTCCTGGTT

ATCGCCCTGGCCACGTTCCTTCTGTGGCTGTTCTTCTCGGGCGACCTCAACGCAGCCTTCACCGCCGCCG

TCGCCGTCCTGGTCATCGCTTGCCCCTGCGCCCTGGGCCTGGCAACTCCCGTCGGGCTACTGACGGGGAC

GGGCCGCGGAGCTCAGCTGGGCATCCTCATCAAGGGCCCTCAGGTTCTTGAGGACACTCGCCACGTGGAC

ACCATCCTGCTGGACAAGACGGGCACCGTGACCAGTGGCAAGCTCGCTGTGGACCACACAACGGGCCTCA

ACGGCTACTCGCCGGCCACCGTCTTGACGTTGGCCGGAGCGGTTGAGTCGGCATCAGAGCATCCGATCGC

GCACGCAATTACGGCCGCGGCGAAGGACGCCATGCGCGACGCCGGCACTCTCCCTGGTGTTGACGGCTTC

AGTTCCGCTCCCGGCGGCGGCGTCAGGGGGTCCGTTGCAGTTGACGGGGTCTCCAAGATGGTGGTCGTCG

GACGCTCGGGCTGGCTGGAGGAGAACGGCATCCCGCTCGATTCAAGCCAACGGGAAGCCCTCACGGCCGA

AGAAAACGGCGGCGCCACTGCTATCTGGGTTGCGGTGGACGGCAAGGCCGCCGGAATCGTGAGCCTGAGC

GACACCATCAAACCTGGCTCGGCAGCTGCGATACAGAAGCTTAAGGGAATGGGAATTCGGCCCATCCTGC

TGACCGGCGACAATGCTGCAGTTGCCGCCCAGGTGGCTGCCGCCGTCGGGATCTCACCGGACGACGTGTT

CGCCGGCGTACTGCCGGAGGGGAAAGTTGAGGCGGTCAGGAAGCTGCAGGCATCCGGTGCGACGGTTGCC

ATGGCCGGTGACGGCGTGAACGACGCCGCGGCCTTGGCACAGTCGGACCTTGGAATCGCGATGGGATCCG

GCACGGATGTGGCGATAGAAGCCTCGGATTTGACGGTGATGGGCAGTGACCTGGGTCAGCTGGTGCAGGC

GATTGAGCTGTCCCGCAAGACTCTGTCCACCATCAAGACCAACCTGTTCTGGGCGTTCTTCTACAACGCA

ATCGGCATCCCGGTGGCAGCACTCGGGTTCCTGAACCCGATGATCGCCGGCGCAGCAATGGCAGCCAGCT

CGGTGCTGGTAGTAGCGAACTCGCTGAGGCTGCGCTCGTTCGGTAAGTAG

>gi|256823905:c485969-484404 Kytococcus sedentarius DSM 20547 chromosome, complete genome_91

ATGACCTCCTCACTGACCACCCGCCGCACCTTCCTGGGCGCCTCCGTCGGGTCCCTGGCGCTGGCCGGGC

TGGCCGCCTGCGCCGATCCCGCCACCAGCCCCCAACCGTCAGGGCGCATCCTGCCCACCGACCCGCTGGT

CGCCGAGTACGAGAAACGACGCGCCGCCTCGGGCACCACCGTCACCCACCGGCTCTCCGCCGCACCCCTC

GAGACCTCCCTCCAGGGTCGGGGCCTGACCACCTGGGGATACAACGACGGCCTCAACGGGCCGCTGCTGC

GCGCCCAGACCGGGGACCTGCTCAAGGTCAGCGTCGCCAACGACCTGCCCGAGGACACGAGCGTGCACTG

GCACGGGCTGGCCCTGCGCAACGACGCCGACGGAGTCCAGGGGGTGACCCAGGACCCCATCGCCGCCGGA

GGACGCTATACGTACGGCTTCCGGCTGTCCCATCCGGGCACGTACTGGTACCACTCCCACTTCGACACCC

AGCGCGAACGCGCCCTGTACGGGGCCCTGATCGTCGAGGACCCCAACGAGCCCCTCAGCTACGACCAGGA

ATGGGTCGTGATCCTGGACGACTGGCTCGACGGGATCACCGGAACACCGCAGGACGTCGTCGAGGAACTC

TCCGCCGGGATGGACATGTCCGGCGACATGGAGGGCATGGCCGGCATGGCGGGCATGGACCACGGGTCCA

TGAGCCAGGACAGCTCCCCGGGCATGCCCATGAAGCACATGCTCACGGGTGCTGAGAGCGACTATCTGGG

CGGGGACGCCGGCGACGTGAAGATCCCCGTGCACCTGTTCAACGGCCGCCCCCCATCCGACCCGGACACC

TTCTCCAGCACCCCGGGCAACCGGATCCGCCTGCGCTTGATCAACGCGGCCGGAGACACCGCCTACCGGG

TCGGGATCCCCGGCCAGGAACTGACCATCACCCACACCGACGGGTTCCCGGTCCAGCCCCGCCAGGCCGA

TGCCGTCGTTCTGGGGATGGGCGAACGCCTGGACGTGCTGATCACCCTCGGCGACCAGGCGGTGCCCGTA

CTGGCCCTGCCCGAGGGCAAGACCGGCCACGCCTACGGCATCATCGCAGCCGGTCCAAAGACCACGCTCT

CCGGTCGGCTCCCGCAGACCCTGGGCGGAACCGTGCTCGATGGCAGCCGACTGAAGGCACACGAATCCGT

CCTGCTCCCACCCAAGGACCCGACCAGGACCCACGAGGTACGCCTCACCGGATCGATGATGGAGTACGAC

TGGGGCATCAACGGGCGACGCTTCGACCCGGCCAACCCCTTCGAGGGCGCCTTCGAACTCCGGTTGGACG

AACGCGTGCGGGTCACGATGACCAACGACACCGACATGTGGCACCCCATGCACCTGCACGGCCACACCTT

CCAGCTGGCCGACCATGGCGCCCGCAAGGACACGGTCATCATCAAACCCCGGCAAACCATCGTCTTCGAC

TTCGAGGCGGACAACCCGGGCCAGTGGCTAACCCACTGCCACAACGCCTACCACGCCGAGGCCGGGATGA

TGGGCGTCTTCTCCTACATCCACTGA

>gi|148241099:250863-253139 Synechococcus sp. RCC307 chromosome, complete genome92

ATGGCTCCAGAAGCGCTACTTCTTGATATCGAAGGCATGAAGTGCGGCGGCTGCGTCAGCGCTGTGGAGA

AGCGCCTGCGCGCCCAACCCGGGGTTCAGGAGGCCAGCGTCAATTTGCTGAGCCGCTCGGCTTGGGTGGA

ACTCGAGCCCCCTGCAACGGCGGAATCCTTGCTGGAAAGCCTGGCCGCCCTTGGCTTTCCAGGCCATCTG

CGCGATCCAGACGGTGAGCTGGAGCGCCTGCAGCAAGCCCGCCAGCAGCAGAGCTGGTGGAGCTGCTGGC

GACAGCTGGTGGTGGCGCTGGTGCTGCTGTTGATCAGCAGTGCCGGCCACCTCAGCAGTGGCGGTGTGCT

CGCCGATATGCGCCTGCACGCCCTGGTGGCCACAGTCGCTTTGGCCGGCCCGGGCCGGCCGATCTTGGTG

CGCGGTTGGAAAGGCTTGGCCCAGGGCGTTCCTGGCATGGACAGCCTGGTGGGCTTGGGCGTGGGCACGG

CCTATTTGGCCAGCATGGTGGCTTTGATCTGGCCCTCGGTGGGCTGGTCTTGCTTTTTCAATGAGCCGGT

AATGCTGCTCGGCTTTGTGCTGCTGGGCCGCTTTTTGGAAGCCCGCGCCCGCCGCCGCACAGGCGCTGCG

CTGGAGGAGTTGGCGCAATTGCAGCCTGAAACGGCCCTGTTGCTGCTGGGGGATGGCTCCACCCGCAGCA

TCCGCGTGGGTGGGCTGCGGCCCGGCGATCATCTGCGGGTGCTCCCGGGTGATCGTTTACCCGTTGACGG

GGTGGTGATCGATGGCAGCTCATCGCTGGATGAATCGAGCCTCACCGGCGAACCATTGCCCCGCAGCGTG

GCCAGTGGTGATGAATTGGCTGCCGGCAGCCTCAATTTGCAGGCGCCGCTGGATCTCAAGGTGCTCAGGC

CGGGATCGGAATCAGCGCTGGCCCGGGTGATTGCCCTGGTGGAGCAAGCCCAGGCCCGCAAGGCACCGGT

GCAGGCCATGGCGGATCGCTGGGCGGGCCGCTTCACCTGGATCGTGATGGCTTTGGCCCTAGCCACGTTT

CTGTTCTGGTGGCTGCTGGGCAGTGATCTCTTCCCTCAGGTGCTCGATGGCATGGCCCATGGCCCCGGCC

ATCACCGCAGCTTGGGCAGTGGCGCTGAAACTCCCTTCGCCCTGGCCTTGCAGTTGGCCATTGCTGTGCT

GGTGGTGGCTTGCCCGTGCGCGTTGGGGCTGGCCACCCCCACAGCCATCAGCGTGGCCACTGGCCGCGCC

GCCCATTTGGGCCTGCTGTTCCGCGGCGGCGATGTGCTGGAGGTGGCTGCTTCGGTGCGCACGGTGTTGC

TGGATAAAACCGGCACCCTCACTCGCGGCCGGCCCCTGCTGGAAGACCAGCTGGTGATCGCCGATGGCCT

GGATGCTTCGGGTTTGCTGCAACTGGCTGCGTCCTTGGAGCAAACCACCCGCCACCCCTTGGCGTGGGCG

CTGCTGCAGGCCGCTGAAGGCCAGGGCTTGAACTTGCTGAGCTGCAGCAGCAGCACCACCCAGGCCGGCG

ATGGCGTCGAGGGACAGTTGGATTCGCAGCAGCAGCTCTGCCGTTTGGGTCGTTTGGCCTGGCTTGAGCA

CCACGGTGTTCATGTGCCTGCCATGGCCAGTGCATGGCAGCAGCAGCAGGAGCAGGGCGGGGCCACGGTG

CTGGCCTTGGCCCATGGCCAGGAGCTGTTGGGGCTTTTGGCCGTGCGCGATGCCATGCGCCCCGATGCGG

CTGAGGCCCTGGCCCAGTTGCGCAGCGATGGCTTGGCCTTGGCCGTGCTCAGCGGTGATCGTGAGGGCCC

CGTTCGGCAGCTCGGCCAACAGCTTTCTCTGCAGCCGGATCAATTGGCTTGGGGGCTGCGGCCTGAGCAC

AAGCTCCAGCGGTTGCATCAGGCCTCTGGCCCGGTGGCGATGGTGGGTGATGGCCTCAATGACGCCCCAG

CCTTGGCGGCGGCTGATCTCGGCATTGCGGTGGGAACCGGCACCCAGATCGCCCAGGACAGCGCTGATTT

GGTGGTGCTCGGGGAACGCCTGATGGCAGTGCCGCAAGCATTGGCCTTGGCCCGCTCAGCCCAGGCCAAA

ATTCGCCAGAACCTCAGCTGGGCATTCGGTTACAACCTGCTGGTGTTGCCCTTGGCCGCTGGGGTGCTGC

TGCCAGGCTTTGGCCTCTTGCTCACTCCTCCTTTGGCCGCTCTGTTGATGGCACTGAGTTCGATCACGGT

GGTGGCCAATGCTCTGTTGCTGCGCGCTCCGGCATGA

>gi|554634310:2135703-2137964 Gloeobacter kilaueensis JS1 chromosome, complete genome93

ATGCAGATGCAACTGAAGATTCCGGCGATGCACTGCGGCGGTTGTGCCCAGGCGGTGAGACGGGCTGTGC

TGGAACTAGATCCCGGTGCGCAGATAGATGTGGATCTACAAGCGCGCACGGTGCAGATCGAATCGAAGCA

CCCGGAGGAGAACCTGCACCAGGTTCTCGCCGAAGCGGGCTATCCGCCGGAGAAGGCGGGGGAAGCTGAA

GGTTACGGGCAACAGGCCATAGCGGACGACAGCAGCACCGAAGCTGTAGACGAGCAGGAGGCGGTGTACC

GGCGGACGATGGGCAAATTTGCCTTTGCGGCGGCGATTGCGGTGCCGGTGCTGCTCGTGGGACTGGGAGA

TTATGTTCCGGCCCTGGCCAGGCTGCTGATGCCTTACCAGCAGGTGGTCGGCCTGGTTTCGGCGGTCCTC

AGCCTGGCGGTCCTTGCCTGGTCGGGGGCGGAGTTTTTTAAGGGAGCCTGGCACAACTTCCGCAACCACA

ACGCCAATATGGACACGCTGATTGCCCTGGGAACCGGGGCAGCGTGGCTCTACTCGTTCTTTGTCGTCGT

CGCACCGGGGCTTTTTCCGGTGTCGGCGCGCGGCACGTTCTTCGATGTCGCGGTGGTGGTGACTGCCCTG

GTACTGTTGGGTCAGGCGCTCGAAGCGCGGGCCCGTGGCCGGGCCAATGCCGCTTTGGGAGCCCTCCTCG

ATCTGCAGGCCAAGACGGCGCGGGTGCTCCGCGACGGCCAGGAAATGGACCTGCCGGTGGCCCAGGTGCG

GGTGGGCGACATTCTGCTGGTGCGGCCCGGCGAAAAAGTGCCCGTGGACGGAGTGATTGTCCAGGGCGAA

TCGGCGATCGATGAATCGGTCGTCACCGGCGAATCGGTGCCGGTGGACAAAAAATCTGGCGACGGGGTGA

TCGGCTCCAGCCTCAATACGACCGGTGCTTTTCGGATGCGGGCCACCCGCGTCGGTGAGGAGACTACCCT

CGCCCAAATCGTGCGTCTGGTGGGCGAGGCCCAGAGTACGAAGGTACCGATTGCCCGCCTGGTGGACACC

GTGTCGCGCTACTTCGTGCCCGGCGTGGCGATCGTCGCTATCCTCACGTTTTTAGTCTGGTTCAACCTCG

GTGCGCCCCTCAATCTGGCGGTGGTGACGGCGGTGACGGTGCTCGTGATCGCCTGTCCCTGTGCCCTGGG

GCTGGCGGCTCCTATTAGTCTGGTGGCGGGACTGGGCAGAGCCGCCGAAAACGGCGTGCTGGTGCGCAAT

GGCGAGGCACTTGAGACGGCTTCCAAGCTTACGACGATCGTGCTCGACAAGACCGGCACGATCACCCTCG

GCAGACCGGAGTTGACCGATGTGCTGCCTGTGGCCGGGTTCAATCTGGATACATTGCTGGCTCTGGCCGC

CGCCGCTGACCGCAACAGTGAGCATCCGCTCGCTACCGCCATCGTCAAAGGCGCTCAGGCGCGAGGCACC

CAAGCGGCTGAGCCCGAACGCTTCCGGTCCATTCCTGGCCAGGGCGTCGAGGCAACAGTTGATGGGCGAA

CGGTGCTGGTGGGCAATGCCCGGCTGATGGAGGCCCAGAACGTGGCGATGGCAGCCTTCCAGGGCGATGC

GGCGCGGCTGGCCAACTCGGGCAAGACGGCGATGTTCGTGGCGGTGGACGGGCAGGCGGCGGGCATTCTC

GCCGTGGCAGACACGGTGAAACCCGATTCGCTCGCAGCGATCCGGGCTTTCGCTCGGATGGGCATCGAGG

TGGCAATGATCACCGGCGACAACGAGCGCACGGCTCAAGCCATTGCCCGGCAGGTGGGCATTGAGCGGGT

CATAGCCGGGGTGCAGCCCCAGGACAAGGCCCAGCAGGTGCAACGGTTGCAGGGCGAAGGGCGCACCGTC

GGCATGGTCGGTGACGGGGTAAACGACGCTCCGGCTCTGGCCCAGGCGGATGTTGGTTTTGCGATCGGTA

CGGGCACCGACGTTGCCATCGAGGCGGCGGACATCACCCTGGTTGGCGGCTCGTTGCGCTCGGTGGTCTA

CGCCATCCAGATCTCGCGGGCCACCCTGGGCAACATCAAGCAGAACCTCTTCGGCGCTTTTATCTACAAC

AGTCTGGGCATCCCGGTGGCGGCGGGGCTCTTTTATCCGCTGTTTGGCTGGCTGCTCTCGCCCATCCTCG

CCGGGGGAGCGATGGCTCTTTCGAGCATTACCGTCGTACTCAACGCCAGCCGCCTGCGGCTTTTTGCTCC

CAAATCCGTTTCGGAGGCATAA

>gi|257057919:2512523-2514328 Cyanothece sp. PCC 8802 chromosome, complete genome94

ATGAAAAATCTATTAAATAGACGCAACTTTTTAAGGTTAACTACTGGACTAGGTTTAGCCATTGGATTCG

ATTATTTACTTCCCAGTTACGGCAAAACTGTCAAGCAAATAAAAGCCTCTCAAGATGAGATAAAATATGC

TGATGTTATTGACTTAACGATTCAGGAAACTAGCCTCAAAATTGGCAATAAAACTGCCACTGGAGTAACA

GTTAATAATACCATTCCTGCTCCTTTACTGCGATTAAAAGAAGGACAAATCGCTAAAATCAACGTTACCA

ATAACTTAGATCAAGACACTTCTATTCATTGGCACGGTATCATCTTACCGTCGAAAATGGATGGAGTTCC

TGGGGTGAGTTTTGCGGGAATAAAACCCGGTCAAACCTTTACCTACGAATTTCCTGTTATTCAGAGTGGA

ACTTATTGGTATCACAGTCATAGTGGCCTTCAAGAACAACGAGGTCACTATGGTGCAATAATTATTGATC

CCTTAGAACCTGACCCCTTTGAATACGACAAAGATTATGTAGTCATTTTATCTGATTGGACTGATGAAGA

CCCCCATGATGTATTAGGTAATCTCAAAAAAATGAGTGCCTATTACAATAATCAAAGACGCACCATTGAG

CATTTATTTGAAGATTCTATGTGGAGAGAAATGCGAATGGACCCCACAGATATTGCTGATGTTACGGGTG

CAACCTATCGCTTTTTAATGAACGGGTTAGTTGCTGAAGATAATTGGACGGCGATTTTTCAACCCGGAGA

AAAAGTTAGACTCAGATTTATTAATGCTGCGGCCATGACTTTTTTTGATGTGCGTATTCCTGGGTTAAGA

ATGACAGTTGTACAAGCAGATGGACAGAACATTCAACCTGTTACCGTAGATGAATTTCGTATTGGAGTTT

CAGAAACCTATGATGTGATTGTACAACCCAAAAAAGAGCAAGCTTATACTATTTTTGCAGAAACTATGGA

TCGTAGTGGGTATGCAAGTGGAACTTTAGCGATTAATCAAGGAATGAGTGCTCCTATTCCTAAACTTCGA

GAACGTCCTGTCCGTTCCATGAAAGATATGGGAATGGATCATGATATGTCAGGACATAATGATCATGGTT

CTATGAGTCAAAATAGTTCCGCAACCGTTGATCATTCTACGATGAATCATGATACTTCAGGACATAGTGA

TCACGGTTCCATGAATCATAATACCTCCGAAGATAAAGATCAGTTAACCATGGAACATGATATGTTCAAT

GATGAAGCAGTTCCCAATACTTCCCAAGATTTCGGAGTAGCTAATGCTGGTAGTCCTATGATGGTACAAA

ATCGCTTAAATGAACCAGGAATCGGGTTAGAAAATACGGGAACTCGTGTTTTAGTTTATAGCGATTTGCG

TAATATTAAACCCGGATATGACCAACGCAAACCTGAACGAGAAATAGAAATCCATTTAACCGGAAATATG

GAACGGTATATGTGGTCATTTAATGGCAAAAAATACTCAGAAGAAAAGGAAATTACCTTCTACAAAGGGG

AAAGATTACGGTTAACATTTATCAATCAAACGATGATGGAACATCCCATACACTTACATGGGATGTGGAT

GGAATTAGATAACGGTTCAGGGAACTATAAACCCCGTAAACATACCGTTGTTGTCAAACCTGCTGAAAAA

ATGTCTGTTGAAGTGAACGTTGATGTTACTGGTAAATGGCCGTTACATTGTCATCTGCTTTATCACATGA

AAGTTGGAATGTTTCGGACTGTTGCCATTGCTGATCGTCCTGTTGAGGCTAGTTAA

>gi|434408264:c276672-274756 Stanieria cyanosphaera PCC 7437 plasmid pSTA7437.01, complete sequence95

ATGAACAATAAATCTAGTACATTCAATAGACGAAACTTTATCCGTTTCACTGCTGGAATGAGCGTAGCAC

TGGGACTCGATAGCCTTCTCCCCGCATATGTCAAACCAGTCGTTGCTGCAAACAAGTCAAAAACTAAATT

TGAATATTCCGATCCCATTGACTTAAAAATTCAAAAAACCAAACTCCCAATTGCAGGAAAAAGTGCCTCT

TCTTTGACAGTTAACGGCTCGATACCCGGACCTTTGGTACGTCTTAAGGAAGGTCAGACAGCAACGATTC

AGGTTACAAATCAGTTAGAGGAAGATACATCAATTCACTGGCATGGCATTATTCTTCCTCCCAATATGGA

TGGAGTACCTGGTGTAAGTTTTGCAGGGATTAAACCAGGAGAAACTTTTACCTATCAATTCCCCGTCACC

CAAAGTGGCACTTATTGGTATCACAGTCACAGTGGAATGCAAGAACAACTGGGGCATTTTGGTCCGATTA

TTGTTGACCCAAATGAACCCGAACCTTTTGAGTATAACCGAGATTATGTCGTCATGCTTTCCGATTGGAC

TTTTGAAAATCCCCACCATATTCTTGCCAATCTCAAAAAAATGCCTGCTTACTACAATTATCAACGAAGA

ACCATCGCTAATTTATCCGAAGATTGGGCATGGAAGGGGATGCGAATGGATCCTACCGATATCGCCGATG

TTACTGGAGCAACCTACACCTATTTAATGAACGGTTTGGCACCAGATTCCAACTGGACTGGACTCTTTCA

ACCTGGTAATAAAGTACGACTTCGCTTTATTAACGCTGCTGCAATGACTTTCTTCGATGTTCGCATTCCT

GGACTCAAAATGACTGTCGTGCAAGCAGACGGTCAAAATGTACAACCAGTAGCTGTCGATGAATTCCGTA

TTGGTGTTGCCGAAACTTATGATGTGATTGTAGAGCCTCAACAAGAGCAAGCTTATACTATTTTTGCAGA

AACGATGGATCGCAGTGGCTATGCTCGTGGCACATTAGCAATTCGTGAGGGACTAAGCGCGTCTCTACCC

GAACGTCGCGAACGTCCATTGCGGAGTATGGCAGATATGGGAATGGATCACGGATCATCAGGTATGAATC

ACGATATGTCAAGCATGAATGGTAGTTCTACTATGAATCACGATATGTCAAGCATGAATGGTAATTCCAA

CATGAATCACGATATGTCAAGCATGAATGGTAATTCCAACATGAATCACGATATGTCAAGCATGAATGGT

AATTCCAACATGAATCACGATATGTCGAGCATGAATGGTAATTCCAACATGAACCACGATATGTCGAGCA

TGAATGGTAGTTCTACTATGAATCCCGATATGTCAGGTAAGGATACAGGAGTTCCTCACGGTTCTGACGA

TCATGGTTTAGGAAATGCAGCAGTGCCAATGATGGTAAAAAGTCGGTTAAATGAACCTGGTGTTGGTTTA

GAAAATACGGGAACTCGCGTCTTAGTTTATACTGATTTGCGAAGTTTAAAGCCAGGTTCTGACCAAAGAA

AACCAGAACGAGAACTAGAATTACATCTTACTGGCAACATGGAAAGGTATATGTGGTCATTTGATGGTAA

GAAATATTCAGAAAACAAAGAAATCACATTTTACAATGGAGAACGACTGCGCTTAATCTTTGTCAACGAC

ACCATGATGGAACATCCAATTCATCTACACGGAATGTGGATGGAATTAGATAATGGCGCAGGAGAATATA

AACCCCGCAAACATACAATCAATGTCAAACCTGCTGAAAGGCTACCTGTAGAAGTTAACGTCGATGCGCC

AGGTAAATGGGCATTTCACTGTCATTTGCTCTACCACATGGAGATCGGGATGTTTCGTACCGTAGCTGTT

GTCGATCGCCCTCTGGAGGCAAGCTAG

>gi|427733619:2240379-2242235 Rivularia sp. PCC 7116 chromosome, complete genome96

GTGAATTATAAGACTAATGCACTTACCAGACGCAACTTTTTACGTTTCACAACCGGAATCGGCATAGCAG

TAGGGCTAGATAGTCTTGTACCTGCATATGCCAGACAAGTAGTTGCGAATGAAACCGGAAAAGGACGTAA

TTACCCCGATGTTATCGATCTAAAAGTTCAGGAAACAGCAGTTAAAATCGGAAACAAGCAAGCCAAAGCC

CTAACAGTTAACGGCTCAGTTCCTGGTGCTTTAATCCGTCTGCGTGAAGGACAAAACGTCACCATTAATG

TAACCAATGAACTTAAAAAAGATACTTCAATCCACTGGCATGGTATCCTTCTTCGCCCGAATATGGATGG

TGTACCTGGAGTGAGTTTTGCAGGGATTAAACCGGGACAAACCTTCACTTATCGGTTTCCTGTGGCTCAA

AATGGCACGTACTGGTATCACAGTCACAGCGGATTACAAGAACAACGGGGTCATTATGGTGCGCTTATTA

TTGACCCGATTGAACCCGAACCTTTCGAGTATGACCAAGATTATGTCGTTATGCTCTCCGATTGGACGTT

TGATAATCCCCACGATATTCTCGCCAATCTCAAAAAAATGCCCGCTTACTACAATTATCAGCGACGAACC

ATCGCCGACTTGTCCAAAGATTTGGCATGGAGACGTATGCGGATGGACCCAGCAGATATCGCTGATGTTA

CTGGGGCAACCTACACCTACTTAATGAATGGTTTAGTCCCAGACTCAAACTGGACTGGACTTTTTCAAGC

TGGTAATAAAGTACGACTTCGGTTTATTAATGGTTCGGCAATGACTTTCTTTGATGTTCGCATACCTGGA

CTCAAAATGACCGTAGTGCAAGCAGACGGTCAAAATGTACAACCAGTAAGCGTCGATGAATTCCGTATTG

GCGTTGCAGAAACTTATGATGTGATTGTAGAACCTCAAGATGAGCAAGCCTACACGATTTTCGCAGAAAC

AATGGATCGTAGTGGTTATGCTCGCGGCACATTAGCAGTTCATGAGGGACTTAGGGCACCCCTTCCCGAA

CGTCGCGAACGTCCTTTACGTACTATGGCAGACATGGGGATGGCTCACGATATGTCAGGCATGAATGGTG

ACTCCTCCATGAAAGATATGCCAGGAATGAATAATAATTCTTCCATGAACCACGATATGTCTGGTATGAA

TAGTAATTCCTCCATGAAAGATATGCCAGGAATGAATGATAATTCTTCCATGAACCACGATATGTCAGGC

ATGAATGGTGATGATTCCTCCATGAAAGATATGCCCGGAATGAATCATGATATGTCCAAGAATGATGCAG

GAGTTCCCCACGGTCCTGACGATCATGGTTTAGGAAATGCAACAGTACCAATGATGGTAAAAAGTCGGTT

GCATGAACCTGGTGTTGGTTTAGAAAATACAGGACATCGCGTTTTAGTTTATACCGATTTACGTAGTTTA

AAACCCGGATATGACAAAAGAAAGCCAGAGCGAGAACTAGAACTACATCTTACCGGAATTATGGAACGAT

ATATGTGGTCATTTGATGGTAAGAAATATTCCGAAGACAAGGAAATCACTTTTTATAATGGGGAACGACT

GCGCCTAATTTTTGTGAATGACACAATGATGGAACATCCAATTCATCTACACGGAATGTGGATGGAATTA

GATAATGGTGCAGGAGAATATAAGCCCCGCAAACATACAATTAATGTCAAACCTGCTGAAAGACTACCTG

TAGAGATTAATGTAGATGCCCCCGGTAAATGGGCATTTCATTGTCATTTACTTTATCACATGAAAGTAGG

GATGTTCCGTACAGTAGCTGTTGTTAATCGGGGGTAA

>gi|113473942:551971-553365 Trichodesmium erythraeum IMS101 chromosome, complete genome97

ATGGCAACTTTAATCACTCAATGTAATATAAATAATACTCAAAAAAATCAACAACTTATATCATCATACC

CTCAAATACATCAGAGCCAAGATGGAATTCTAGAAATTGATTTGACAGCTAATATTAGTCAAATAAATCT

GGGAAATAAACAAGCTTATGTATTAAATTATAACGGACAAATTCCCGGACCAAGATTAGAAGTAAAACCA

AGAGATACAATTAGGATTAATTTCACTAACAATTTACCCCAACCTACAAATATTCATTATCACGGACTAC

ATATTCCCATGACTGGAAATGCTGATAACGTATTTCTGAATATTTCTCCAGGCGATCGCTTGACATATCA

GTTTCAAATTCCTGAAAATCACCATGCTGGAATGTTTTGGTATCATCCACATCTCCATGGTTTAGTAGCA

GAACAAATTTTTGGGGGGTTAGCTGGATTATTTATTGTCCGTGGAGAGCTCGATGAAATTCCCGAAGTAA

AAGCTGCTTCAGAACAATTTTTAGTGTTGCAGGATTTTGCCCTAGACCAAAAAGGAAATATACTCCCATC

TCATGAGCTTTCAATAATGACAGGAAGGCAAGGAAATTTGATCACTGCTAACGGTCAGTCTAACCCTAAA

TTAACCATTCCCCCTCAAAATTTATTAAGAATTCGTATTCTTAATGCTTCAAGTTCCAGATTTTATCGGT

TATCTTTAGAAGAACACCCATTTTATTTAATAGCTACTGATGGAGGAGCATTAGCTGAACCTGTAGAAAT

GTCAGAACTATTATTGACTCCTGGAGAACGTGCAGATGTATTAGTCCAAGGTAACCGAGAGCCAGGAAAA

TATCGCTTGTTGAATTTACCTTATGAACGAGTGGGTATTGGTATGATGGGGAGAAATGTATCTGAAAGTC

AACCACAAGTTTTAGCAACAGTGAGTTATGACCAACAGTCCACTGCTGTGCCACTGCCAACCAAACTCAT

CCCTGTCCCAGCATTGCCCGAACCTCAAAAAGTACGTCGTTTTCAGATGAACCATGGCATGATACCTGGT

CAAGGGATGGCATTTTTGTTTAATGGTCAGCCATATCAACCAGAAAGAATTGATACTCAAGTTCAGCTCA

ATACCGTTGAAGACTGGGAATTAATTAACACAGGCATAATGGATCATCCCTTTCATATTCATAGCAATGC

TTTTCAAGTTTTGAGTCGGAATGGCAAACCAGAACCCTATCGTGCCTGGAAAGATGTAGTCTTAGTCAGA

CAGGGAGAAACAGTTTTAATTCGGATGCCATTACAAGATTTTGCTGGAAAAACTGTTTATCATTGTCATA

TTCTTGACCACGAAGATTTAGGAATGATAGGAAATTTGGAAATTAAAGCAAGACTTTCTATTTAA

>gi|347534971:1017667-1019919 Flavobacterium branchiophilum FL-15, complete genome98

ATGAAAAATGAAAAAATAAAAAACATATCAATATTGCCTACGGCAATATTGATATTGTTTATTTCTCAAT

CCATTTTCGCACAAAAAGTAGTGAGATATGATTTATATGTACGTGATACAATAGTAACTTTTGGCGACAA

ACCAAAACACGCCATTGCGGTAAACGGACAAATCCCAATGCCAACACTAACATTTACCGATGGCGACATT

GCGGAAATTTATGTACATAATGAATTAAACGAAGAAACATCTTTGCATTGGCACGGATTATTTTTACCAA

ACAAAGAAGATGGCGTTCCTAATTTGACACAAATGCCAATAAAACCCAAAACGACACACAAATATTCTTT

TCCAATTATTCAACACGGCACACATTGGTATCACAGTCATTCGGGACTGCAAGAACAAATCGGAATGTAC

GGAAATTTTGTAATGCTCAAAAAACCAACCGACCCAACTTTCAGAAAAGGAATTGACGATTTGCCAAGCA

TTCCAATAGTTTTAAGCGAATGGACAGATGCAAATCCTGATAATGTACACAGAATGTTGCACAACGCAAC

CGATTGGTTCGCTATTCAAAAAGGCACAACACAAAGTTATGCAGAAGCAATCCAACAAGGTCATTTCAAA

ACCAAAGTAACCAACGAATGGAAACGAATGAACGCAATGGACGTGAGCGATGTTTATTACAATAAATTCT

TAATAAACGGAAAAAACGAAAGCCAACTTTCGCAATTCAAAGGTGGCGACAAAGTGCGATTACGCATTTC

AAACGGTGGAGCATCAACCTACTTTTGGCTCAACTATGCAGGCGGAAAAATTACCGTTGTTGCCAACGAT

GGTAACGATGTAGAACCTGTCGAAGTCGATAGACTAATCGTTGCAGTTTCTGAAACCTACGATATTATTG

TAACCATTCCAGCCGACAAAACCGCTTACGAATTTTTGGTAACTTCCGAAGACCGCACAAAATCCGCATC

CTTATATTTGGGCGATGGCATCAAACAACTTACTTCTACCCTACCAAAATTAAAATATTTTGAGGGTATG

AAAATGATGAATGATATGATGAAAATGAATGGAGATTTAGACGATATGGGAATGAGTATGTCATACAATC

AAATGGATATGAACGTAGTAATGTATCCCGAAATTACAGGCGAAGCAAAACCCAAAAGCGACAAAATGGG

CGATATGAAAATGACCGAAGACGATTATAATAGCAATAAACTTTCAGACATTGTAACACTAAATTACGCA

ATGTTAAAATCACCTTCCAAAACAACACTCCCAAAAGACGCACCAATCAGAGAATTAAAATTTGAACTTA

CAGGAAATATGAACCGCTACGTTTGGAGTTTAGACAACAAAGTAGTTTCTGAAACTGATAAAATTTTAAT

CAAAAAGGGCGAAAATGTACGCATCACAATATATAACGGCTCAATGATGCGACACCCAATGCACTTACAC

GGACACGATTTTAGAATTATAAACGGTCAAGGCGACTACGCTCCACTCAAAAACATCATTGATATTATGC

CAATGGAAACTGATATTTTAGAATTCAACGCCAATGTAGAGGGCGATTGGTTTTTTCATTGTCATATTCT

CTACCATATGATGGCAGGAATGGGCAGAGTTTTTACTTACGAAAACCAAGCACCTAATCCATTAATTTCA

AATCCAAAACTCGCACAGCGCAAACTTTTTGCCGACGACAGAGCATTTCATTTTATGGCAGAAAATGATT

TTGCTACTAATGGTAATGATGGTAGCGCAATGTATCAAAATACACGTTGGAGTATTGGTACGGAATGGAG

ATTAGGTTACAATGATATGCACGGTTACGAAATCGAAACACACATAGGTAGATATATTGGTAAGATGCAA

TGGCTAATGCCTTTTATTGGATTTGATTGGAGATACAGAAAAATGGAAGATAACGAGATGGAAAAAAACC

TTTTCAATCAAACAAATACCAAAGACAAACGTTCTGTTTTTAGTCTTGGATTAGAATACACCTTGCCAAT

GCTAATAAAGGCACAAGCCGAAGTGTTTACTGATGGAAATGTACGTTTACAATTCGAACGAATGGACATT

CCAGTAACCAAGCGATTACGAATGAACTTAATGTGGAATACTGATAAAGAATATATGGCAGGATTACGAT

ATATAGTAAAACGAAATTTTGGTATAACCACACATTACGACAGCGATATGGGAATAGGCTTTGGAGCAAG

TCTAAATTATTAA

>gi|410109000:c120156-117277 Fibrisoma limi BUZ 3 plasmid pFLIM01, complete sequence99

ATGAAACATTTCAATATTCTTTCGGTCGTCGTTTCTACGCTCCTGCTGTCAACGGCATACGCCCAGCATC

AGCAACACACCCCGGCTGCAACTGACCAGGCTACTGATAAACCGTATGACCCCGATAAGTCGAACTTTTC

CGCGGTCAGAACAACCTTACCTGTTTCCCGGCCGCAGGCGTCGGCGAAGGTAAAATTAACCGGTAAACGG

ATAACCTACGACCTGGTCATTGACGAACAAACGGTTAACATTACCGGCAAGCCAACCAAGGCGATGACCA

TCAATGGCGGCATTCCAGGCCCAACCATGCGGTTTACCGAAGGGGATGTAGCCGTAATACGCGTTCACAA

TAAATTAAAGACAGAAACCTCGTTGCACTGGCACGGTCTACTGCTTCCCAATAAACAGGATGGGGTTTCT

TACTTAAATACGCCCCCCATTCATGCCGGTGATACCTACACATTCGAGTACCCGCTGGTGCAGTCAGGTA

CGTATTGGTTCCACTCGCATACCCGCTATCAGGAACAGGTTGGCGTATACGGATCAATCGTTATTCAGCC

TCAGCGAATCACCCATAAAGTCGACCATGATCTGGTGCTGTTGCTGTCGGACTGGACCAATGAAAACCCG

GCTTACATTTTGAAAAACCTAAAACGGCGCAACGAGTGGTATTCCATTAAAAAAGGTAACGTCCAGTCAC

TTAACCGCATCATCCGGCACAAAGCCGTAGGGGCTTATCTACGCCAGTCCATGATGAAAATGGCCCCGAT

GGATATTTCGGACGTCTACTACGAACGGTTTCTGATCAACGGTAAAGACACCACTCGTCACCCGGAGATC

AAGCCTGGCCAGACCGTTCGACTGCGGGTCATCAATGCCTCCGCTTCGACCTATTTTCATGTGAACTTTG

CGGGCGGTTCCCTCAAATTGATTGCAGCCGACGGGTTGGACGTTCAGCCTGTGGACGTTGACCGTCGGCT

GATTGCCATTGCCGAAACGTATGATTTTCTACTTACGGTGCCCCAAGGGGGTGCCTTCGAGATACGGGCA

ACGGCTCAGGATGGTTCCGGTTATGCATCGGCCCTGCTGGGTAGTGGCCTGGTTCACTACGCCCCGACCA

TACCCCGCCCTGATCTCTACAAGCTGACGGCGAACATGAGCCGTATGGGAGGCATGGGTATGGGTTCTAT

GCGTATGGGACGTGCCGACACAACGGCCCAGGCAAACGTAGAGATGAACGGCCCGATGATGCACAATATG

ACCGGAATGAATCATGTACCCAGCCAACACCCTACGGATGCCCCAAAACCGACCCCGAAAGCAGCAGACA

TGGGCGCCATGGACCATAGTCAGATGCAGATGGAGCCATCCAAGCCAACCGGGACTAAACCCCAGAAACA

GGCGTCAATGGCTGGAATGGACCATAGCCGGATGGATATGGGCGGAAAGCCGGCAGCGAAGAAGCCGACT

GCGGCCAAAAACACTACTAAGTCATCAAAATCGGCCGGATCAATGGCGGGCATGGATCATAGTGGTATGA

ACATGGGCGACATGAATCCAGCCAAAGATGGTAAAATGGACATGACAGGAATGGACCACGGGCAAATGGA

TCATTCACCCATGCCCGGCATGGATCATAGTAAAATGGCCATTAGTGCGGATTCGATGCCTGCCACGCAA

ACGGACGGTAAAAAGCCGGATCATATGGTAGGCATGGACCACAGCCAGATGAACAGGACCGAACCCGCAT

CAGCGGGCCGGTCGAATCGCAGACAGTCCGGGGGTATGGCCGGAATGAAGGGCATGAACCACGGGGATAT

GGCCGATGCGTCGGCGGACGGGGCGGACCACGGCATGAGCATGATGGACGAACAAAACACCATTGACTAC

AGCATTCTCAAGTCTCCCGAGCCGATTGTTTTTCCGGCTAATCACCCCATCCGTGAAATTCCGCTCACCC

TAACGGGCAACATGTTTCGCTACATCTGGGGCTTCAACGGACAGCCCCTGTCGCGGGCTGACATGATTCA

GATTCGGCGCGGTGAAATCGTGCGTATTCGCATGATGAACAACACGATGATGATGCACCCCATTCACCTG

CACGGCCATTACTTTCGGGTGCTCAACGGGAAGGGACAGTACTCACCCTTGAAGCATACGGTTAACGTAT

CGCCCATGGAAAGCGTCACCATTGAGTTCATGGCCGACGATGAAAAGGACTGGTTCTTTCACTGCCACCT

GCTGTACCACATGCTCAGCGGCATGGCTCGTGTGGTCAGCTATAGCGACCAACCCGACTCATCCATCGCC

CAGATTCAGAAGCTGCACCTGCGTGACATGCGCGATAACCAGCCCTTCGTATGGGGCTACCTACAACCGG

GTTACCCCCTCAACTATTTTGCCTTAAACGTCTCCAATAACAAAAACGCGATTGTCGCTGGCGGTGATCA

CGACTGGCGAACCAATCGGTTCGAGTTCGATCTGGATTACGAACGATACTTCGGCGACTGGTTCCGGGTA

TTTGCCGGTATTGACGGCGGTAATGAAGAGTTTCTACGTGTGGTACGTCCTGAGGAGGGGGGGCAGCCCA

TTGGCAGTCGGCGCATTGTTAGGGCTGTAGCTGGTGTCCGATATATGTTGCCGTTCCTAATTCAATCGGA

GGTTAAAGTAGATACACGGGGCAACGTCCGGTTTCAACTGAGTGGTCAGCAAAGTCTGTTTCCCAGGCTT

GACTTCCAATATCAGGCACAGTGGCTGGTCAATAGTTATCTGCGAGCACACGTGGAACTGCAATACACCC

TTACCAAGAATCTGTTTCTTCACACCGACTATGACACGCGTTACAAGACCTTCGGAGGTGGATTAGGATA

CAATTTCTAG

>gi|443241923:766433-768817 Nonlabens dokdonensis DSW-6, complete genome100

ATGAAAAAACTACTAATATTTTTCTTAATACTATTTGGATTTGCAATGCAAGCACAAAAACCAGACAATC

CTTTTGTGGCCAACAAATCCGAAGAAAATGTTGATAATTGGCCAGAACGTGTCTATGATTTAACTATTGA

TTATGAGACTGTAAACTACACGGGTAAAGATGTAAAAGCGATGACCTTTAATGGTAGTATTCCTGGTCCA

ACTTTAGAATTTAATGAAGGGGAATTTGCTATTATTAACGTAACCAATAAAATGGATGTAGAAACATCTG

TGCACTGGCACGGTTTAATACTTCCACATTTTTACGATGGTGTACCTTATCTAAATACACCACCTATTAA

GCCAGGCGAAACTCAACAGTACAAATTTGCGCTCAAACAATCTGGAACTTATTGGTATCACTCACATACG

GGATTGCAAGAGCAACGTGGTGTTTATGGCTCAATTCAAATCAATCCTAAAGAGACCACATTAGAGTATG

ATAAAGATTTGGTACTCGTACTTTCGGACTGGATGGATGAAAATCCTAAAACACAACTCCGTAACTTAAA

ACGAGGTAATGAATGGTACCTCATTAAGAAAAATCAAGTACAAAGTTGGGATAAAGTAATCGCCAGAGGT

GCTGTTGGAGCTAAGTTGAAAATGATGTGGCAACGTATGCCAGATATGGCTATACTTGACAATTACTACG

ATTATTTTTTTATAAATGGAAAACCTGAACAGAACTATACTGATTTTAAACCTGGTGAAAAGGTAAGGGT

ACGTTTTGTAAATGCAGCTGCTGCCACTTATTTCTGGCTTACATTCGGTGGCGAAGACCCTACGCTAGTA

GCAGCGGATGGGTTAGATGTTGTTCCTGTACCACACAATAAAACATTAATCGGAGTTGCCGAAACATATG

ACTTTATAGTAACTATTCCAGAAAGCGGAAAGCTGGAAATCCTTGCAACTTCACAAGACGGTTCTGGTAA

AGCATCTACCTTTTTAGGAACAGGCAATACATTGAAAGCTCCAGAAGTTCCTAGACCTGATTTGATTAAA

CAGATGCAAGAGATGATGTCAATGGATATGAAAATGGGAGCCCCAGCAGTTAAGTTCAACCCTTCAAAAA

ATGATAGTATTGAAAAAATGAAGAAATACGCAATGAAGATGGATGGAATGCAAATGGATGGAATGAATAT

GAAAGATGACAAAATGCAGATGGATGGGATGCAGAAAGATAAAGATTCCATCAAAATGAAAGACAACGGT

ATGGCTCATCAGGATATGAAAATGAAGGAAGATAAGATGCAAATGGATGGTGGAATGAAAATGGGCTATA

TGGTTCCAAAAGACAAAGTGATAGGTGATAATATGAAAACAGGTGGTAATCCTGAATTTAATTATCACTA

TTTAAAAGCGCCAGAAAGTACCGAATTTGACGAAGACAGACCTACACGTGAGATGCTATTTAACCTAACT

GGTAATATGAACCGATATGTATGGTCAATAAACGGAGTACCACTTTCAGAAACCGATAAAATAAAAATCA

ATCAGGGAGAAGTTGTACGAATAACGCTTAACAATATGACAATGATGCACCACCCAATGCATCTACACGG

ACACTTTTTTAGGGTACTTAATGAAAATGGTGAGCGCAGTCCATTAAAACACACGGTTAATGTTGCACCG

ATGCAAAAGGTAGTCATTGAGTTTGCTGCGGATGAAAAGGGTGATTGGTATTTTCATTGTCACGTTCTAT

ATCATGTAAACAGTGGTATGGCTAGAATTTTTAGCTACGATACACCACGAGATGAACGATTAGAAGGATA

TCCTTTGACCAATCTTACCAATGAGGCAAATCATATGTTCACTTGGGGAGAAGTCACTGGGGCAAGCCAT

ATGACAGAATTATATTATACGGCAACGAACATTCGTAATCAGTTTACCTTAAGAGGAGAATATGGCTGGA

ATGAAAATTTAGAAGCAGAGTTTACATACGAGCGTTGGCTCAATAGTTATTTCAGGGTTTTTGGAGGCGT

AAATGTTGAAAACGAAGGCGTGGATAGCCTTGAAGAAATATCTACCACAGGTGTCGTAGGTGTTCGTTAT

TTGCTTCCACTTCTAATCGATTCCGATTTACGGATTGATAACAAGTTACGTCCTACTATCTCTTTTGCGA

CAGCGACTATGATATTTAGAAATGTTGCTCTTTATGGTGAGTATGAATACCAAATGGATTTTGGTTGGGT

AAATGATTTTGAAGAAGGGACTGATTTTGAAGAAGAAACGACTTGGCAAGTAGGCCTTGAATATGTACTG

AGTAGAGATTTCTCTTTAATGGGAAGCTATGACAACCGTTTTGGATGGGGTGGCGGACTTTCATTAAGAT

TTTAA

>gi|374290092:c400946-398721 Blattabacterium sp. (Cryptocercus punctulatus) str. Cpu chromosome, complete genome101

ATGAAACAAAATGAAAATTTTAAATTTCTTGATGATGAAAAAATTGCCGAAAAAATAATTGATTTTAAAG

ATAAAAATATCACTTCCGTTCGTTTTTTTATTCCATCTATTCATTGCAGTTCATGTATCATGATTTTGGA

AAATTTATCTAATATTCATAAAAATATAATTGAATCCACTGTTGATTTTCCAAGTAAAATAATTAGAATA

ATATTCAATCATTCTGGATTAAAATTAAGCTATTTAGCAATTATTCTTGAAAAAACAGGTTATAAACCTT

CTATTAATTTTGAATTTTTAGAAAAAAAAAAATAAGAAAAAAATATTTGATAGAAAATTAATAGGAAAATT

AGCTATTTCTTTTTTTTGTTTTGGAAACATTATGCTTTTATCTATTCCAGAATATGTAGGATCTCATGAA

GATATATGGTATTTAGAAAATCGTAATTTCTTTCGTTATTTAATGTTAATTCTTTCGTTACCTATTGTAT

TATTTTCTTTCACAGATCATATAAAATATGCTATTTTAGGATTTAAAAAACATATTATTAATATGGATAT

TCCTATTTCTATTGGAATATTAGTTCTTTTTTCATGGAGTTGTTATGAAGTATTTTTTGATTTAGGATCT

GGATATTTTGATAGTTTATCTGGATTTTCCTTTTTTTTACTTATTAGTAAAATATTCCAAATTCATACTC

ATAATAAAATATTTTCTTTTGAAAAAAGTTATAAATCCTTTTATCCTATTTCAATATCAAAAATTAAAAA

AAATAATCAAGAAGAAAATATTTTACTTTTTTCCTTAAAAAAAGGAGATCATATTATAATTAGAAATGAA

GAAGTTATTCCTGTAGATTCTATATTAATAAAAGGATCTGCATTATTAGATAATAGTTTTCTTACAGGAG

AATCTTATTTAATAAGAAAAAAAAATAGGAGAACGAATTTATGCTGGATCTAAACAAAAGGGGGAAGCTAT

TTATTTAAAAGTAATCAAAAATGTAGATCAAAGCTATCTAAGTTTATTATGGAATAATAAAAAATTTCGT

AAAAATAAAAAAAATTTTCATTTAAATTCAATAACTAATCAATTTAGTCAATATTTTACTCCTATTGTTT

TAATCATTTCTATAATAACTGGAATATATTGGTATTTTATTAATGTATCAAAAATTTTTCAAACTGTTTT

TTCCGTATTAATTATTACTTGTCCTTGTGCAGTAGTACTTGCCACTCCATTTATACTTGGAAATTTCATA

CGAATATTTTCTAAAAAAGGATTTTATGTAAAAGATATTTCAACTATGGAACGAATTTCCAATATAAGTA

CTTTAATTTTTGATAAAACAGGAACTATTACAGATCAAAATAAAGAAAAAATTTCTTTTATTGGTCCATA

TCTTAAATGTAAAGAAAAAAAAAATGATTTCCTCTTTATTAAGAAATTCAATTCATCCATTAAGTAAAAAA

ATTTTTTCAGAATTATCTATCAATCAATTTTATCCTATAAAAAATTTTCGAGAAATAATAGGTTATGGTA

TAGAAGGAATTATAAATAAAGTTCCAGTTAAAATTGGATCTTCTAAATATATAGGTATTACAATAAATAA

TTCTAAAGGAACTTCAGTTGCTATTTCTATAAATAAAAAATTCATAGGTTATTTTTTATTTAGAAATTAT

TATAGAAAAGGAATAGAACAAATATTCCAAAATCTAAAAAAAAAATATAAAATAATTATTCTTTCTGGTG

ATAATAATAAATTAGAAAAAGAATATTTAGAATCAATTTTACCAAAATCTAGTAAAATTATTTTTAATCA

AAGTCCAGAAAAAAAACTAGATTATGTAAAAAAAATACAAAAAAATGGAGAAAAAGTAATGATGATTGGA

GATGGTATTAATGATTCTTCTGCATTAAATCAAAGTGAAGTCGGCATTTCAATATCAGAAACTCCAAGTA

GTTTTTTTCCTAATTGTGATGCTTTTATTCAATCTAATTATTTGAATAAAATTTTTTTATTTCTAAAAAT

ATCCAGGATATCAATCAAATTGATTATTGTAAATTTTATGATTAGTTTATTTTACAATTGTATAGGAATT

ATATTTGCTATAACCGGTTTTTTAACTCCTTTTATTGCAGCAATTTTAATGCCTTTAAGTTCTTTATCCG

TAATTATTTTTTCTCTATTATCTACTTGGATAGTTTCACGAAGATTTTTATCTTAG

>gi|294505815:c2045657-2043036 Salinibacter ruber M8 chromosome, complete genome102

ATGAGTCGCCCTTCTCCCGAGACTGAGACGTCGCCACCAGACGCTCCCTCCCACGCCCCGTCCGCCGAGG

GGGACGCCTGTGCGGTCGAGCCGGTCCCTTCCGAACGTCGGGAGGGCCATTCCTCCGGGGAGCGAGGAGA

GCGCCTGACGCTGCCCATAAAGGGCATGTCGTGCGCCTCCTGCGCGAAGAGCGTGGAGGGGGGGCTCGCG

GGTGTTGATGGGGTGGCGGAGGCCCGTGTCAACTTTGCAACCGAGCGGGCCCAGGTGGGGCTCCAGAAGG

GAAGACGGGTGCCGGTCGGGAAACTGGCGCAGGCCGTCGCCGACAGCGGCTACGAGGTGCGCACCGCCGA

AACGGCCCTCACGGTGCAGGGGATGACCTGCGCCTCGTGCGTCTCCCGCGTCGAAGACGCCTTGGGCGCG

GTGGAGGGGGTGCTGGAGGCGACTGTGAATCTCGCCACCGACCGCGCCACGGTGCGCTACGTGCCGGGTG

CAACCGAGCGCACGGACCTCACGCGGGCCATTCGCGACGCCGGCTACGAGGGGGGCGGGACGGAAGACGG

GGGCGACCGTGCCGACGTGGAGAACCAGGCCCGAGAAGACGAGCAGCGGCCGATGAGGCAGCGGTTCCTC

TGGGCGCTGGCCTTCGCGCTTCCGGTATTCGTGTTGGAGATGGGCTTCATGCACATCCCGGCAATGGAGG

CCTGGATCACCGATCAGGTGTCCACCCAGACGCTGTACTACGTGCTGTTTGCCCTGACGTCGGTGGTGCA

GTTTGGGCCGGGGCGCTACTTTTACAAGCACGGCTGGCCTGCGCTCAAGAGCGGCGCCCCCGACATGAAC

ACGCTCGTGATGATGGGCACCTCGGCGGCGTACGGCTACTCGGTGGTGGCGACCTTCCTCCCGACCGTGC

TGCCGGAGGGAACCGTGCACGTCTACTACGAGGCCGCCGCGACGATCATCACCCTCATCCTGGCGGGCAA

CTACATGGAGGCGCTCGCCAAGGGCCGCGCCAGCGACGCGATCCGGGCGCTGCTCGACCTGCAGGCCACC

ACGGCCCGCGTTCGTCGCGACGGCGAGGAGGAAGAGATCGACGTGCGCGACGTGGTGCCGGGCGATGTGG

TTCGGGTGCGGCCCGGCGAGAAGGTGCCGGTCGACGGCGAGGTGCTCGACGGCCAATCCCACGTCGACGA

GTCGATGGTGACCGGCGAGCCGGAGCCGGTGCGCAAGGCCGAGGGCGACGAGGTCGTGGGGGGTACGATC

AACGAGACCGGCAGCTTCACTTACCGCGCCACGCGGGTGGGGGAGGAGACGGTGCTCTCCCAGATCGTAG

AGATGGTGGAGCAGGCGCAGGGATCGGCCCCGCCCATTCAGTCGCTCGCCGACCGGGTGGTCCGGGTGTT

CGTCCCGTTCGTCCTGGCGACGGCCGCCCTCACCTTCGGCGTCTGGATGGTGTGGGGGCCGGACCCGGTG

CTCACATACGCCCTGGTCTCCGCCGTATCCGTCCTCATCATTGCGTGTCCCTGCGCGATGGGCATCGCCA

CGCCGATTTCGGTGATGGTGGGGACGGGGCGCGCGGCGGAGCTGGGCGTGTACGTCCGGGAGGGCGACGC

GCTGCAGGCCCTCCACGCGGCGGACCTCGTGGCGCTCGACAAAACCGGCACGCTCACGAAGGGCCGGCCC

GAGGTGACGGACGTGCGCCCGCGAGATGGATTCGGGGAGACGGACGTGCTCCGCTGGGCGGCCGCCGTAG

AGGCCCAGTCCGAGCACCCGATTGGGGCGGCGCTGGCGGCCCGGGCCGAGGAGAGGGGGCTCGCCCTGCC

GGACGCGACGGACTTTGAAGCGATCCCCGGACACGGCGTGCGGGCCACCGTGGAGGCGCGCGATGTCGCG

GTGGGGGCCGCCCGCCTCATGGAACGGCACGACGTTGCGATTCCCGCCGGGGCCGAAGAGACGGCCGACC

GCCTGGCCGACGCCGCCAAGACGCCCCTGTACGTGGCGGTGGACGGGACGCTGGCCGCCGTGGTGGCGGT

GGCCGATCCCATTAAGGAGTCGACGCCCGCGGCGCTCGACGCCCTGCACACCCTGGGCATTGAGGTTGCC

ATGGTTACGGGCGACGACGAGCGCACCGCCCGGGCCATTGCCGACCAGCTCGGCATCGACCGCGTGCAGG

CCGAGGTGCTGCCCGACGAGAAGGCCGCGGCGGTGGACGACTTTCAGGCAGGGGGACGGACGGTCGCGTT

CGTGGGCGACGGCATCAACGACGCGCCCGCCCTGGCCCAGGCCGATGTCGGCATTGCCATCGGCACCGGC

ACCGACGTGGCCATCGAGAGCGGCGACATCGTCCTAATGGCCGGGGACCTGCGCGGCGTCCCCAACGCGG

TGCACCTGTCGCGGTCCACCCTCCGCAACATCAAGCAGAATCTGTTCTGGGCCTTCGCGTACAACGTGCT

CCTGATTCCCGTGGCGGCGGGCGTGCTCTACCCGGCGTTCGGCCTGCTGCTGTCGCCCGCCCTGGCGGCC

CTCGCCATGGTGTTCTCCGACCTCTTCGTCGTGGGGAATGCGCTGCGGCTGCGGCGCCTAAGTGTGCCGC

TTGGAGGCGAGCAGGCCGTGGCGACGGCCTAG

>gi|110636427:c1729671-1727257 Cytophaga hutchinsonii ATCC 33406 chromosome, complete genome103

ATGGAAACGAAAGACATATTTGAAATACCTTTATCAGGTGTACATAGTGAGCATTGTGCTCTTTTAGTAG

ACAAGACATTTGCTCAGAATACCGACATTTCTACTCATAAGGTAGACTTCAATAATGAACGTGCTTTACT

TAATTTTAAATCATTAACTCCTGATTTTACGAAGGTGGTTAAAGATATCCGCAGTATCGGATATGATGTA

TTAACTGTTAAAAAATCATTTCCGGTTCTTGGAATGTCTTGTGCATCCTGTGCAAGCAGTTCACAGAGTA

TTCTTGAAACACAACCTGGTGTGATAAGTGTTGCTGTAAATTATGCAAACACAACAGCGCAGATAGAATA

TATTCCAACGATTACAAATCCAAAAAAACTTAAAGCTTCATTACAATCTATCGGTTACGACTTGATGGTT

GATGAAAGCGAAGAAGCAAAAGACACGCTAGAGGAAATTCATAAAAAAAATTATACGATTCTTAGGAATA

AAACAATTGCAGCAATCGTACTCTCATTACCTATCGTTGTAATAGGGATGTTTTTTATGGACATACCATA

TGCAAATTATATTATGTGGGCATTGTCTACACCTGTGATTGGCTATATAGGGAGTTCTTTTTTATCAAT

GCCTGGAAACAAGCCAAACATAGGTCTGCTAATATGGATACACTGGTTGCATTAAGCACAGGTGTAGCAT

ATTTCTTTAGTGTATTTAACACTTTGTTTCCTGAATACTGGCATCAGAAAGGCCTTCATGCACATGTATA

TTTTGAAGCGGCTTCGGTAGTTATTGCTTTTATTTTACTCGGAAAATTATTAGAAGAAAAGGCTAAAGGA

AATACATCTTCTGCAATTAAAAAATTAATTGGTTTGCAGCCTAAAACGGTTACGGTCGTTCATCAAGGTG

GTCATCAGGTTGAAACGCCGATAGCTAATGTAAAGATTGGTGATGTACTATTAGTGAAGCCGGGAGAAAA

GATTGCTGTAGACGGCAAACTTGTTTATGGCAACTCTTTTGTGGATGAAAGTATGATAAGTGGCGAACCT

ATCCCTATTGAAAAAAATATAAATGATTCTGTGCTTGCAGGCACGATAAATCAAAAGGGAAGTTTTCAAT

TCGTTGCAGAAAAAGTCGGTGGAGATACGATGCTGGCACACATAATTAAAATGGTGCAAGATGCACAAGG

TAGTAAAGCTCCGGTTCAAAAACTTGTTGATAAAATCGCAGGTGTATTTGTACCGATAGTTATTGTAATA

GCAATCTTAAGTTTAATAGTGTGGGTGTTGTTTGGAGGCGAAAACGGTTTCACGCAAGGTATGCTGGCAA

TGGTTACTGTTCTTGTAATAGCGTGCCCTTGTGCGTTAGGATTGGCCACTCCTACGGCAATAATGGTTGG

TGTCGGGAAAGGTGCTGAAGCTGGCATCTTGATAAAAGATGCTGAAAGTCTTGAGACTGCAAGGAAGATA

AATGCAATTATCCTCGATAAAACAGGAACGATTACAGAAGGCAAGCCTGCTGTGACGGATGTTATTTGGT

TGAATGAAAACGCAGACAAAATTAACGTTTTCGCATCCATTGAAAAACAATCCGAACATCCCCTTGCGGA

AGCTGTTGTTAACTATTTTGGAGAAATGAGTTACCTAAAAGTTGATAATTTTGAAAGCATTACCGGTTCT

GGAGCAAAAGCGGTTGTAGATAATACATCGTATTTCATAGGTAATATGAAATTGATTGATGCACAAAAAA

TCAACATACATACAAATCTTAGAAATAAATCTAATCTATGGCTTAAAGAAGCTAAGACTGTCATTTACTT

TGCTAATACCACAGAAGTTTTAGCAGTATTAGCTATTTCGGATAAAATCAAAACTACTTCCATAGATGCA

ATTAAACAACTCAAAACGTTGGGCATAGAAGTATATATGCTTACAGGCGACAATACCTTTACTGCTGAAG

CTATAGCCAAGCAAGTCGGTATAAGTCAATTTAAAGCAGAAGTGTTACCATCTGATAAAGCGAATTTTGT

TAAGGAGCTGCAGGCACAAGGTAAAGTTGTTGCAATGGTAGGTGACGGAATCAACGACAGTAATGCATTG

GCACAGGCTAATGTTAGTATAGCCATGGGCAAAGGAAGTGATATTGCAATGGATGTTGCAAAAATGACTA

TCATTTCATCTGACCTTTCTAAAATACCTCAAGCAATAAAACTATCTACCTATACGGTTAATGCCATCCG

ACAAAATTTATTCTGGGCATTTATCTATAACATAATCGGTATCCCTTTGGCAGCTGGTATCCTCTACCCT

TTTACAGGTTTTTTATTAAACCCAATGATTGCCGGAGCAGCTATGGCACTGAGCTCTGTAAGTGTTGTTA

GTAACAGTTTGAGATTAAAATTTAAATCCATTTAA

>gi|379728134:2860809-2863028 Saprospira grandis str. Lewin chromosome, complete genome104

ATGTCTAAATATATAACAACTTCTTACCTCATAGAGGGCATGTCCTGCGGGGCCTGTGCCCAAAGTGCAG

AAAAAATTCTCTCCCGCCAAAAACAACTCAGCCGAGTATCGGTCAATTATGCCAGCAAAAAGGTCCAAAT

TACCGCAGAGCAATCGCCAGATTTAGAGCAGCTCAATGCCGCCCTCAAAAAAGCAGGTTTTCAGTTGCTG

CCCCCCGCTCCCGATCAAAGAGCAGTAGCCCGAGAACGGGCCAAAAAGAAGCAACAACAAAGTTTCCGCC

AGTTATTTTTGGCCAGCTTGTTTGCCCTTCCTCTTTTTGTCGTCTCTATGCTGATGCCGCCTTTTGCAGG

CGATAATTACCTCATGCTATTTTGGGCCTCTATGATTTTGTTTGGCCCCGGACGACAGTTTTTTGTCAAG

GCTTGGCAACAACTGAAAGTAGGCCAATCTAATATGGATACTTTGGTGGCTCTAGGCACAGGAACGGCTT

ATGTATATAGTCTGGTCGCTACTTTTTTGCCCAATTGGTGGAGCAGCCAAGGCCTTAGCCCTCATGTTTA

TTTTGAAACAGCTGGCCTGCTGCAAACCTTTATCCTTTTGGGCCGATACTTAGAGGGCCGAGCCAAGGCC

AAAGCCTCTAAAGCCTTAGATGCCCTCTTAGATTTACAACCCCCTACGGTCCAAATTTGGCAGCATGAAA

TCTGGACCGAAATGCCCTTGGCCGCAGCTCAAGCAGGGGACCGCCTGCTCATCCGTCCAGGCGAACAAAT

TCCCCTAGATGCGACTATCCTCGAAGGTGAATCAGAGGTGGATGAACAGCTGTTGACTGGAGAATCTCTG

CCCCAACACAAACTAGTCGGTGCCCCCATTTGGGCAGGCACGCTCAACCAAACGGGCCGTTTGCTGGTAG

AGGCCAAAAAAGGAGCCGACCAAAGCCGTTTGGCCCAAATTATTGAGCTGGTCGAAACTGCCCAAAGCTC

TAAGGCCCCCATTCAAAAATTGGTGGATAAGATTTCAGGGATTTTTGTGCCTGTTATTTTGGTTTTGGCC

CTGCTTACCGCCTTAATTTGGTTCTTTTTTGGACCAGCACCGGTCTGGAGCCATGCCCTAAATAATATGG

TGGCTGTTTTGGTCATTGCTTGTCCCTGCGCTCTAGGATTAGCTACTCCCATGGCTATTATGCTAGGCAT

TGGCCGAGGGGCCAAGTTGGGCTTGCTGCTCAAAGGGGCGGAGAGTCTAGAAGCCGCCCAAGCCATTGAT

GCCTTTGTCTTTGATAAAACGGGTAGCCTTACCGAGGGCCAAATGAAGCTTCAGGCAGAAGAATGGCGGC

AATCTATGGCCCATTATTGGCCCGCACTTTACGCCTTGGAAGAAAACTCAGAGCATCCATTAGCCAAGGC

TTTGCTAAACTTGAGCGAGCGCCCCAGCCATTTACCAAAGGTAGACCATTTTCAGGCCCGCTCTGGCCGA

GGCGTTTTGGGCCAGATTGCTGGACAAACTTATGCGGTGGGCAATCGCCAGTTATTAGTAGAGCAAGGGC

TCGATCCTAAGGATTTAGCCCATGGACCTTGGCGAAGCTGGGAGGCCGAAGGCCAATCGGTCCTCTTTTT

TGCCGTAGAACGGCAGCTCATTGCCGCCTTTGCCTTCTCCGATGTATTAAGAGATGGGGCCAAGGAAACG

GTAGCGGCCCTACAAGCTCGTGGCATCAAGACCTATATGTTGAGCGGTGACCAAAAAGTGGTAGTGGACC

GCCTAGCCAATGAGTTAGGCATTGCCCATGCCCAAGCGGAACTCTTACCAGAAGATAAAATCAACTTCAT

TAAGGAGCTGCAGGCTCAAGGACTAAGGGTGGCTATGCTCGGAGATGGCCTAAATGATGCCCCCGCTTTG

GCCCAAGCCGATTTAGGAGTATCCCTAAATGCAGGAACAGGAGTGGCCCTAGAAGCTGCAGAAGCCTTGT

TGCTCAAGAATGAACCCTGGCAGCTCATTCAACTACTCGATTTGGGCCAAGCGAGTATGAAAATTTTGCG

ACAGAATTTATTCTGGGCCTTTGCCTATAATTTGCTGGGGATTCCCATTGCAGCAGGACTGCTTTATCCT

TTTACGGGTTATTTATTGGATCCAATGTTAGCTGGCGCCGCTATGGCTTTTAGTTCGGTTTCTGTAGTGC

TCAACAGTTTGCGCCTGCTCTCTTGGCCCATTTCCAAAATGGCTGCCTAG

>gi|268315578:1607644-1609509 Rhodothermus marinus DSM 4252 chromosome, complete genome105

ATGAAGAGCTTCACACGTCGTGATTTTCTGCAGGCCCTGGCGTTGCTGGGCATTTCGACGGGGATCGAGG

CGCTTCTGCCGGCCTATGCGCGCTCGCGTCCGCGCGTGCCCGCTCCCCGTCGGCCACGCCGAGGCCCGGT

AACCTACGACCTGACCATTGCCGAAACGCCGATCCGCATCGGCGGCCGCAGGGCAAAGGCCACCACCATC

AACGGCACGGTGCCGGGGCCGCTGCTGCGCTTCCGCGAAGGCGACGAGGTGATCATCCGCGTCACCAACC

GCCTGAAAGAAGACACCTCCATCCACTGGCATGGCGTGCTGGTGCCCTTCGACATGGACGGCGTGCCGGG

CGTGAGCTTTCCGGGCATCAAACCCGGTGAAACCTTCACGTACCGGTTCCGGCTGCGCCAGCACGGCACC

TACTGGTACCACAGCCACAGCGGGCTGCAGGAACAGACGGGCATCTACGGCCCGCTCATCATCGACCCGG

CCGAAGAGCCCTATCCCTACGATCGCGAATACGTGATCGTGCTGAGCGACTGGACCTTTGAAAACCCATA

CCGCGTGCTGGCCCGGCTGCGCAAGTACCCCGGCTACTACAACTTTCAGCGGCGCACGCTGGCCAATCTG

TTCGAGGAAGCCCGTGAGATGGGCTTCTGGAAGGCGCTGAAGGATCGGCTGAGCTGGGGCCGCATGCGTA

TGGGTGCCACGGACATCGTGGACATCACCGGCGCCACCTACACGTACCTGATGAACGGCCACGCACCCGA

AGACAACTGGACGGCCCTGTTCCGGCCGGGCGAGCGCGTGCGGCTGCGGTTCATCAACGCGGCGGCCGGC

AGCATCTTCGATGTACGCATCCCCGGTCTGCCGATGACGGTGATTCAGGCCGACGGTCAGTACGTGCAGG

CGGTTACGGTCGATGAGTTTCGCATCGGCATTGCAGAAACGTACGATGTAATTGTCGAGCCGAAAGAAGA

GCAGGCCTACACGATCTTTGCCGAGTCGATGGATCGCAGTGGCTATGCCCGCGGTACGCTGGCGCCGCGC

GAGGGCATGGAAGGTCCCATCCCGCCGCTCCGGAAGCGCCCGCTGCGCACGCACGCCGACATGGGCATGG

TGCACGAAGGACACGAAGGCCATGCGGGCATGCCTCACGAGGCCATGGCCGCGCCCACCGAACACCGGGG

GCACCAGATGCCCGCCGACACGACCGGACATCACATGCACGGCGGACATGACCACCATGGCGGACATGCC

ATGATGGCGGGCATGATGGGCGGCGTGGGGCAGCCGCCGGGTATGCCACCGGAAGCGCAGCCTCACGGCG

ACGACACGCACGGCCCGGGCAACGCGGCCGTACCGATGATCACGCGCAGCCGCCTGCACGAGCCGGGAGT

AGGCCTGGGCGAAGACGGCTGGCGTGTGCTGGTCTATACCGACCTCAAGAGCCTGCACCCCCGCGAGGAC

TTCCGTCCACCCACCCGTGAGATCGAGCTGCACCTGACCGGCAACATGGAGCGCTTCCTCTGGGGCATCG

ACGGCAAAACCTATTCGGAAGCGCCCGAGCCGATTCGTCTGCGCTACGGCGAGCGGGTGCGGCTCGTGCT

CGTCAACGACACGATGATGGAGCACCCCATGCACCTGCACGGGATGTGGATGGAACTCGAAAACGGCCAT

GGCCGGCACATTCCACGCAAACACACGATCCTGGTCAAGCCGGCCGAGCGGCTTTCCGTGCTGATCACGC

CGGACGAACCCGGTCCGTGGGTGTTCCACTGCCACATCCTCTATCACATGGAGATGGGGATGTTTCGCGT

CTTTGAAGTATCCGAACCGGAAACGGCAACCCGAACGTCATCATGA

>gi|313205511:1994409-1996637 Riemerella anatipestifer ATCC 11845 = DSM 15868 chromosome, complete genome106

ATGGATAATCAAAAAAATATTTATAAAAAATTGTTGCCGATAGTTCTAATGCTATTGGCAACTACAACCA

TTTTCGCCCAAAAAGTGGTGCGATATGACCTTTACGTAAAAGACACTATTGTTAATTTTACAGGAAAAGA

AAAAAGGGCAATAGCCGTAAACGGACAAATTCCTATGCCAACACTCACCTTTACCGAAGGCGATACGGCA

GAAATTCATGTTCATAATAGGCTCAGAGAAAGCACATCGTTGCATTGGCACGGATTGTATTTGCCCAATA

AAGAAGATGGTGTGCCCTACTTGACCCAAATGCCCATTGAACCGAATACGACCCACGTATATCGTTTCCC

AATTATTCAAAACGGTACACATTGGTATCACAGCCACAGCGGACTGCAAGAACAAATCGGAATGTATGGC

TCAATGGTTTTGCTCAAAAGAGCAGACGACCCAACTTTCAGAAAAGGCATTGATGATTTGCCTTCTGTGC

CTATTATTTTGAGCGAATGGACAGACTATAATCCTGATAATGTACACCGAATGTTGCACAATGCTAATGA

TTGGTTCGCCATTAAAAAAAACACTGTGCAGAGTTATGCAGAAGCCAAAAAAGCAGGACATTTCAAAACC

AAATTGACCAACGAATGGAAACGAATGTTGGCAATGGACGTGAGTGATGTATATTATGACAAATTCTTGA

TTAACGGAACATCTGAAAGCCAACTCGCTCAATTCAAAGCAGGCGACAAAGTAAGGTTGCGAATATCAAA

CGGTGGTGCATCTTCCTATTTTTGGCTCACGTATGCAGGTGGAAAAATGACTGTCGTTGCCAATGATGGC

AATGATGTTGAGCCTGTTGTGGTGGATAGACTAATTATAGGAGTTTCAGAAACCTATGATGTCATCGTTA

CCATTCCGGCAGAAAATACTTCCTATGAATTTTTGGCAACACCCGAAGACCGAACTAAATCAGCATCTAT

TTACATAGGTTCAGGTATTAAGCAGTTGGCAAGTCCATTGCCTAAACTGAAATACTTTGAAGGTATGAAG

ATGATGAACGATATGATGAAAATGGATGGCACAATGAACGATATGGGTATGGATATGTCATTGCAACAAA

TGGATATGAATACGGTAATGTACCCTGAGATTACAGGAGAAAATAAACCCAAAAAATCCGAACATTCAGA

CCATAGCAATCATACAATGCCCAGTAATGACATTGTTACCTTAAACTACGCAATGCTGAAATCACCTACC

AAAACCACTTTGCCAAAAGATGCACCTATCAGAGAATTAAGGTTTGAATTAACAGGCAATATGAACCGCT

ATGTGTGGAGTATGGACAACCGAGTGCTTGCTGAAACTGATAAAATTCTAATCAAAAAAGGAGAAATTGT

AAGAATAACACTCTACAACAACTCGATGATGCGACACCCGATGCACTTGCACGGACACGATTTTAGGGTT

ATAAACGGGCAAGGCGATTATGCACCGCTTAAAAATGTTTTGGACATAATGCCTATGGAAACCAATGTGA

TTGAGTTTGAAGCCAATTTAGAAGGCGATTGGTTTTTCATTGTCATATTCTCTACCATATGATGGCAGG

GATGAACCGAGTTTTTAGTACAGAAAACCAAGCACCCAATCCATTGTTGCCTGACAAAAAATGGGCTTAC

AAAAAACTACAAAGAGAAAGCAACGAACTACATTTTATGTTTCAAAACGACTTTGCAACAAATGGAAATG

ATGGTATGACAATGTTACAAAATACCCGATGGAGTTTTGGCACTGAATGGCGTTTGGGATATAGCGACAA

ACACGGATACGAAACAGAAACACACATAGGGCGATACATAGGCAGAAACCAATGGCTAATGCCCTTTATT

GGCTTTGATTGGCGATACAGAAAGCACGAAACACCAGAAAAAAATCTTTTTGGGCAGACTAATACCAAAG

ACAATCGGACACAGTTTAGTTTAGGGGTTGCATATACTCTGCCAATGCTTATAATTTTACAAACAGAAGT

CTATCACGATGGAAACGTAAGAGTACAATTAAGACGTGAAGACATTCCACTTTCAAAAAGGTTTAGAGCT

GCATTTATGGTAAATACAGACAGAGAATATATGCTTGGACTGAATTATATCGCAAGTAAAAACTTAGGTT

TCAGAACGCATTACGACAGTGATATGGGTTTTGGAGTAGGACTGACATTTAATTATTAA

>gi|327402011:c4250481-4248232 Fluviicola taffensis DSM 16823 chromosome, complete genome107

ATGGTTCTACAAAATATTCTTTCAAAAAAATTGCTGCCGATAGTATTTTTACTATTGGTCAGTAATAATA

TATCCGCTCAAAAAACAGTTCGTTACGACCTTTATGTAAAAGACACTTTGGTAAACTTTACTGGGAAATT

CAAAAGAGCCATAGCCGTAAACGGACAAATCCCTATGCCAACGCTCGTCTTTACAGAAGGCGACACAGCA

GAAATTTACGTTCATAATTTGCTTAAAGAAGAAACATCTTTGCATTGGCACGGCTTATTTTTACCCAACA

AAGAAGATGGCGTTCCTAATTTGACGCAAATGCCAATCAAGCCAAATACTACACACGTTTACCGTTTTCC

AATTATTCAAAATGGCACACATTGGTATCACAGTCATTCGGGTTTGCAAGAACAAATAGGAATGTATGGC

AATTTTATAATGCTCAAAAAATCTGATGACAAAACATTTCGAAAGGGAATTGATGATTTACCTACCGTTC

CCATTGTATTAAGCGAATGGACAGACTTAAAACCTGAAAACATTCACAGAATGTTGCATAATGCCAACGA

TTACCCAGCATTGAAAAAAAATGCCGTTCAAAGTTATGCAGAAGCCATTAAAGCTGGGCATTTCAAAACT

AAACTCAAAAACGAATGGAAACGAATGTTGGCAATGGATGTAAGCGATGTGTATTACGAAAAAATATTGA

TGAATGGCAAATCCATTACCGACCTTTCAATAATTGACGGAAAGGAATTAAAGGCAGGCGACAAAGTGCG

TTTAAGAATTTCAAATGGTGGTGCTTCATCTTACTTTTGGCTTACTTATGCAGGTGGAAAAATTACCGTA

GTTGCTAATGATGGCAACGATGTAGAGCCTGTTGAAGTAGATAGATTGATTATTGCTGTTTCTGAAACCT

ACGATATTATTGTAACTATTCCTGCCGAAAATACGGCTTTTGAATTTTTGGCAACCACCGAAGACAGAAC

CAATTCTGCATCATTATACATTGGCAACGGCATAAAACAACTGAAATCATCACAACCAAGATTGAAGTAT

TTTGAAGGAATGAAGATGATGAATGATATGATGAAAATGAATGGCGATTTGGACGATATGGGAATGAATA

TGAGCCTAAATCAAATGGATATGAATGTGGTAATGTATCCGGAAATTACTGGAGACAGCAAACCAAAACA

AAGTGATAACGACCCAAACCGATACAACGCCAATGCTTTGGCAGATATTGTTACGTTAAACTATGCAATG

TTGAAATCACCCAACAATACTTCACTTCCAAAAGATGCACCCGTAAAAGAGTTAAAATTTACACTAACAG

GAAATATGAACCGCTATGTTTGGAGTTTAGATAATAGAGTAATATCTGAAACTGATAAAATTCTAATCAA

AAAAGGTGAAAATGTTCGTATCGTTTTATATAACAACTCTATGATGCGACATCCAATGCACTTGCACGGA

CACGATTTTCGTTTACTCAATGGTCAAGGCGCCAATGCACCCTTAAAAAATGTTGTGGACATTATGCCAA

TGGAAACCGATACATTGGAATTTAACGCCAATGTAGAAGGCGATTGGTTTTTTCACTGTCATATTCTCTA

CCATATGATGAGTGGTATGGGCAGAGTATTTACTTATGAAAATCAAGCTCCAAATCCTTTAATAACAAAT

CCTAAATTGGCTCAACGTAAACTATTTGCAGACGACCGTAAATTTCATTTTATGGCAGACAACGATTTTG

CTACCAACGGAAACGATGGAATGGCAATGTTACAAAACACTCGTTGGAGCATAGGTTCAGAATGGCGTTT

AGGCTACAATGATATGCACGGCTATGAAACCGAAACACATATAGGACGATACATTGGAAAAATGCAATGG

CTGATGCCGTTTATTGGTTTTGATTGGCGTTACAGAAAAATGGGAATTGATGAACAAGAAGAAAATTTAT

TCGGACAAGTAAATAAAAAAGACAACAGAACTGCTGTAAGTTTAGGGTTTATGTACACGCTTCCAATGTT

GGTGAATTTTCAGGCAGAAGTGTATCACGATGGAATTGTTCGACTTTCTTTAATGCGAGAAGATATACCG

ATTACGAAAAGAATAAGAGCTGGTTTTATGGTAAATACAGACAAAGAATTTATGGTTGACTTGCGTTATA

TCATAAATAGAAATATGGGGATTAGAACACACTACGATAGCGATATGGGATTTGGAGTTGGACTGACATT

AAATTATTAA

>gi|325102757:1876259-1878529 Pedobacter saltans DSM 12145 chromosome, complete genome108

ATGAATATACCCATAAATAGTAAAATAAGGTTGCTGCAAGCAGTATTTATACTGTTATGTTCGCAATCCC

TATTTGCACAGAAAGTAGTGCATTACGACTTGTATGTAAAAGATACGCTTGTCAACTATGCAGGAAAAAC

AAAAAGAGCCATTGCCGTAAATGGGCAAATACCTATGCCCACGCTCACATTTACAGAGGGCGACACTGCC

GAGATTGTAGTGCATAACCAGTTAAAAGAAAGTACATCACTGCATTGGCACGGCGTATTTTTGCCCAACA

AAGAGGACGGTGTGCCCTGGCTTACACAAAAGCCTATTGAAGCCGGAGCAACATACACCTATCGTTTTCC

AATTATCCAACACGGAACGCACTGGTATCATTCCCATTCAGGTTTGCAGGAACAGATTGGAATGTATGGC

AGTTTTGTAATGAAAAAGCGGGATGACGACAAAACCTTTAGAAAGGGCATTGATGATTTGCCTACCGTAC

CCATTATTTTAAGCGAATGGACAAATTATAACCCCAACAACATTAACCGAATGTTGCACAACGCCAATGA

TTGGGCAGCTATCAAAAAAGGTGCAACACAATCTTATGCAGAAGCTATTAGAGAAGGGCATTTCAAAACC

AAATTAACCAACGAATGGAAACGTATGTTGGCAATGGACGTAAGCGATGTGTATTATGACAAAATATTAA

TTAACGGAAATCATACTACCGATTTAAAAACCGTTGACGGCAAAATACTAAAAGCAGGTGATAAAGTAAG

GTTAAGAGTTTCAAATGGTGGCGCATCCTCTTACTTTTGGTTACGGTATGCCGGAGGTAAAATTACCGTA

GTAGCCAATGATGGTAACGATGTAGAACCTGTTGAAGTAGATAGGTTAATTATAGGAGTTTCTGAAACAT

ACGATGTCGTTGTAACAATCCCCGAAGAAGGTGTTTCTTATGAATTTTTAGCCACTACTGAAGACCGAAC

ACAATCAGCCAGTTATTTTTTAGGTAATGGTATCAAACAATTAATTTCCCCACTGCCCAAATTAAAATAT

TTTGAAGGGATGAAGATGATGAACGATATGATGAAGATGAATGGTGATTTGGACGATATGGGAATGAAAA

TGAGCCTGAACCAAATGGATATGAACGTGGTAATGTATCCCGAAATTACAGGAGAAGCCAATCAAAAGCA

AGACCATAGCCAACACAATATGAATATGGATAACGACCCCAATCGTTACAATGCAAATGCTTTAGGAGAA

ATTAAAACCCTAAACTATGCAATGTTGCAATCGCCCTACAATACTTCACTTCCCAAAGATGCACCCGTAA

AAGAACTAAAATTTACACTTACCGGAAATATGAACCGCTATGTGTGGAGTATGGATAACAAAATTCTTTC

AGAAGTTGATAAAATTCCTGTAAAGAAAGGAGAAATTTTACGCATCACCATTTACAATAACTCAATGATG

CGACACCCAATGCACCTGCACGGTTTCGACTTTAGAGTAATTAATAGAAAAGGCGAAAAATCACCACTGA

AAAATGTGTTAGATATAATGCCAATGGAAACAGATACCATTGAATTTTTGGCAAACGAAGAAGGAGATTG

GTTTTTCCATTGCCATATCCTATATCATATGATGTCGGGGATGAACAGGGTTTTTGCTGTGGATGACTAT

AAAAATCCAAACTTACCCAACAAGAAACAAGCATACAATAAATTACAGAGAGAGAGCAATATGCCACATT

TTATGGCACAGAACGATTTTGCAACCAATGGTAATGATGGCGAGGCAATGTTTCAAAATGCAAGATGGAG

TTTAGGCACAGAATGGCGTTTAGGTTACAATGATATGCACGGTTACGAAGTAGAAACACATTTAGGAAGA

TACATCGGAAAAATGCAGTGGTTTATGCCATTTATAGGCTTTGACTGGCGTTACCGTAAAATGGGAATAG

ATGAACACGAAACCAATTTATTCGGGCAGAAAAACGAAAAAGATACACGTAGAGCCTTTAGTTTAGGTTT

TATGTACACCTTGCCAATGTTAGTCAATTTTCAGGCAGAAGTATATCACGATGGTATCGTTCGCCTGTCT

TTAATGCGAGAAGATATACCAATTTCAAAAAGATTAAGAGCTGGTTTTATGGTCAATACCGATATGGAAT

ATATGGCAGACTTGAAGTATATCATCAATAAGAATATAGGTATTCGTACACACTATGATAGTGATATGGG

CTTGGGTATAGGGCTGTCACTAAATTATTAA

>gi|312128809:c582187-579914 Leadbetterella byssophila DSM 17132 chromosome, complete genome109

ATGAACAGATATAATAAAATACCTATAAGAATTTTGCAAACAGTGCTGATACTGCTTTGCACGCAAACGC

TGTTTGCACAGAGAGTGGTGCATTACGAGCTGTATGTAAAAGATACGCTTGTCAACTATGCAGGTAAGCA

AAAAAGGGCAATTGCCGTAAACGGGCAAATCCCCATGCCCACCCTTACCTTTACCGAGGGCGACACTGCC

GAAATAGTGGTGCACAACCAATTGAAAGAAAGCACATCACTTCACTGGCATGGTGTGTTCCTGCCCAATA

AAGAAGACGGTGTGCCCTGGCTTACACAAAAACCCATCGCACCGGGCACAACCTACACCTACCGTTTTCC

GATTATCCAGCATGGTACGCACTGGTACCATTCCCACTCAGGATTGCAGGAGCAGATTGGCATGTACGGA

AGTTTTATCATGAAGAAAAGAAGCGATGATAAGACATTCAGAAAAGGAATTGACGATTTGCCGACCGTTC

CAATTATATTAAGTGAGTGGACAAATCTTAACCCCGATAATATCAACCGTATGCTACACAATGCAAATGA

TTGGGCGGCCATTAAGAAAGGCGCAACACAATCATATGTTGAAGCTATTAAGGAGGGAAAATTTGGCGTG

AAAGTAACCAATGAGTGGAAGCGGATGCTGGCAATGGATGTGAGCGATGTATATTATGATAAAATCCTTA

TCAATGGCAGCAATGCACCGGAGCTAAAAACGGTTGACGGCAAAACCTTAAAGGCGGGGGACAAAGTACG

GCTTCGCATATCCAATGGCGGTGCATCTTCCTATTTCTGGCTGCGTTACGCCGGAGGTAAAATTACAGTA

GTAGCGAATGATGGGAATGATGTTGAGCCTGTAGAGGTGGACAGGTTAATCATTGCCGTTTCTGAAACTT

ACGATATTGTAGTGACTATCCCTCAAGATGGTTTGGCTTACGAGTTCTTAGCAACAACCGAAGACAGGAC

ACAATCTGCGAGTTACTTTATAGGTGATGGGGTCAAACAGCTTATTTCTCCACTCCCACGATTGAAGTAT

TTTGAGGGGATGAAGATGATGAACGATATGATGAAAATGAATGGCGATTTGGACGATATGGGCATGAAGA

TGAGCCTTAACCAAATGGACATGAATGTAGTGATGTATCCCGAAATTACCGGAGATGCAACAAAAAAAGC

AGACCACAGTAAGCATGAGGGTATGGATATGGATAACGACGCTAACCGTTACAACGCTAATGCTTTGGGA

GAAATCAAAACATTAAATTATGCAATGTTACAATCGCCACACAATACTACTCTTCCTAAAGATGCACCCG

TAAAGGAGCTGAAATTTACACTTACTGGCAACATGAACCGCTATGTATGGAGCATGGATAATAAAATACT

TTCGGAAACAGATAAAATTCCTGTAAAAAAAGGCGAAGTCCTGCGGATTACCATATATAATAATTCAATG

ATGCGGCATCCAATGCATCTTCACGGATTTGATTTTAGGGTTATTAATGGAAAGGGAGAAAAATCTCCAT

TGAAAAATGTTTTAGATATTATGCCAATGGAAACCGATATTATTGAGTTTTTGGCGAACGAAGAGGGTGA

TTGGTTTTTCCATTGCCATATCCTTTATCATATGATGGCAGGAATGAACAGGGTGTTTGAAGTTGGAGAC

TATAAAAACCCTTACTTACCCGACAAGGCAAAAGCCTATAAGGAATTGCAAAGAGAAAGTAATATGCCTC

ATTTTATGGCACAAAATGATTTTGCAACCAATGGTAATGATGGAGAAGCTATGCTGATGAACGCACGTTA

TCAATTAGGTACTGAATGGCGTTTAGGTTATAATAGTATGCATGGTTATGAAGTAGAAACCCATTTGGGA
AGATATATTGGTAAGATGCAATGGTTCATGCCGTTTGTAGGGTTTGATTGGCGATACCGTAAAATGGGAG
AAGATGAGCATGAAAAAAACATCTTTGGACAAATCAACAAGAAAGATACCCGAAGAGCCTTTAGTGTAGG
GTTTATGTACACCTTACCTATGTTGGTCAATTTTCAGGCAGAAGTGTACCATGACGGAATTGTCAGATTA
CAGTTAATGCGTGAAGATATTCCTATTTCAAAAAGGTTAAGGGGGGGCTTTATGGTAAATACTGATTTAG
AATATATGGGTGAACTCAGATACATCATCAATAAAAATATTGGTATTCGCACCCACTATGATAGTGATAT
GGGCTTCGGCGTGGGACTGGCATTGACTTATTAA
>gi|284005577:6864-9704 Spirosoma linguale DSM 74 plasmid pSLIN01, complete sequence110
ATGAAACGTTTTTTTCTCATCACCTGTCTTGTTCTGGTGTTTACGGCCAGCAATGGACAACATCAACAGC
ACACAATGCCCGATGCCAATCGGTCGGCTGAGCAACCTCACAATCCTGACAAGTCTAACTTCCCAGCTGT
CAAAACAAGTTTACCAGTGTCGCGGCCACAGGCGTTGGCGAACGTAAAGCCGACAGGGAAACGGGTACCG
TATGATCTGATCATTGATGAACAGATGGTTAATATTACGGGCAAACCCAAAAAGGCCATGACCATAAACG
GTGGGATTCCCGGACCCACCATGCGGTTTACAGAAGGTGATGTAGCCGTTATCCGGGTTCATAACAAACT
GAAAACAGAAACCTCACTGCACTGGCACGGCATCCTGCTCCCCAACAAACAGGATGGTGTTTCGTATTTG
AATACCCCCCCTATTCAAGCGGGCGACACCTACACGTTTGAGTACCCCCTCGTTCAGTCAGGTACGTACT
GGTTTCACTCCCACACGCGCTATCAGGAGCAGATTGGCGTATACGGCTCGATTGCCATTCAACCTCAGCA
GCCCACTCACAAAGTAGATCATGATTTGGTGTTACTATTGTCGGATTGGACGGATGAAAATCCGGCCCAT
ATCCTGAAAAACCTGAAACGGCGCAACGAATGGTACTCGATCAAAAAAAACAATGTCCAATCGCTCAACC
GCATTATTCAGCACAAGGCCGTGGGGGCGTACCTGCGGCAGTCGATGATGAAAATGGCACCGATGGATAT
TTCCGACGTTTACTACGACCGGTTCCTGGTAAATGGAAAAGACACGGTTCGATACCCGGAAATCAAAGCA
GGTCAAACCGTTCGGCTTCGGGTTATCAACGCCAGTGCGTCCACCTATTTTCACGTTAATTATGCCGGTG
GTCCCATGAAGTTGATTGCTGCCGATGGTCTGGATGTTCAACCGGTGGACGTTGACCGGCGGCTAATTGC
CATTGCTGAGACCTATGACTTTATTGTCACTGTCCCAAACGGTGGCGCTTTTGAAGTACGGGCGACGGCA
CAAGACGGTTCTGGTTACGCAACGGCTTTGCTGGGAAAGGGTAAGGCGAAGTACGCCCCCACTATCCCCC
GCCCCGATCTGTATAAATTAACCGCCAACATGAGTCGAATGGGGGGGGTGGGCATGGGTACGATGAACAT
GGGCAAAACCGACACAACTGCTCAGACTAACTCCGACATGAACGGCCCCATGATGCACGATATGGCCGGG
ATGAATCATGGGGCTAGCCAACAACCCGTAAATTCGGCAAAACCGGGCACCAAAGAACCAACCATGGGGA

ACATGGACCATAGTCAGATGCAGATGGGACAGACTAAGCCAGCACAGGCCGTACCCGTCAAACCGAAAAA

GCAGGATCCGATGGCCGGGATGGACCACAGCCAGATGGATATGGGTGAAAAGCCGTCTGCCAAGCAGCCG

ACTCCGGCAAAAAAGGCCTCAAAGCCGTCAAAAGCGGTTGGATCAATGGCTGGGATGGATCACAGTGGCA

TGAACAGGGACGGCATGAAAACGGCCAAAGATGGTAAGATGGACATGACGGGGATGGACCATGAACAGAT

GGACCACTCCTCCATGCCCGCGTCGGCGGACCGGGGCATGGATCATAGTAACATGGCCATGAAAATGGAT

TCGATGCCCACTACGAAAACGGACAGCAAAAAGCCGGATGACATGGCTGGCATGGATCACAGCAAAATGA

ACATGGACAGTAAAACTGGCGATATGGCCGGGATGAAAGGAATGAGCAGTGGGGGCGGGGGTATGGAAGG

AATGAATATGATGGACGAGCAGAATACCATCGACTACAGCGTTCTCAAGTCTCCCGAACCGATTGTCTTT

CCGGCGAATCGCCCTATCCGCGAAATACCGCTCACCCTGTCGGGCAATATGTTCCGCTACATCTGGGGCT

TCAACGGACAACCACTGTCCCGTGCGGATATGATTCAGATTCGGCGGGGCGAAATTGTGCGTATCCGTAT

GACGAACAATACCATGATGATGCACCCGATTCACCTGCATGGTCATTACTTCCGCGTACTGAACGGGCAG

GGTCAGTATTCACCCCTGAAGCATACGGTGAACGTATCGCCCATGGAAAACGTCACGATTGAGTTTATGG

CTGATGACGAGAAAGACTGGTTTTTTCACTGTCACCTACTGTATCACATGCTAAGCGGAATGGCCCGCGT

GGTTAGCTATGGCGACAAACCGGACTCATCCATTGCTGCTATTCAAAAACTTCACCTGCGCGACATGCGC

GATAATCAGCCGTTTGTCTGGGGCTTCCTGCAACCCGGTTATCCGATCAACTATTTTAGTCTAAATGTCT

CTACTAACAAAAACGCCATCGTTGCCGGGGGAGACCACGACTGGCGCACAAATCGGTTCGAGTTCGACTT

GGACTATGAACGCTATCTCGGCGATTGGTTGCGGGTGTTTGCCGGGATTGACGGCGGGAATGAAGAGTTT

TTGCGCCGGGTACGGCCCGAAGATGGGGTCGTTACCGACCGGCGTATCGTCCGGGCTGTGGCGGGTATTC

GGTATATGTTGCCGTTTTTGATTCAGTCCGAAGTAAAACTCGATACACGCGGTAACGTCCGTTTCCAGCT

AAACGGCCAGCAAATGTTGTTTCCCCGCCTGGATTTCCAGTATCAAACGCAGTGGTTAGTGGGGAGTTAT

GTGCGGGCGCATGTTGAGCTACAGTACACCGTCACCAAAAATCTGTTTCTGCACACCGATTACGATACCC

GGTACAAAACAGTTGGTGGTGGGCTGGGTTACAATTTCTGA

>gi|13357558:246891-249020 Ureaplasma parvum serovar 3 str. ATCC 700970 chromosome, complete genome111

ATGTTAAAGAATAATTTAATTAAAAAATTTCAATTACTACCTATTAATAAAAGAAGATATTTAATTTCTT

TAATGAAAACATCAATAGCACTTCTAATTAGTATTCCTTTAATGGTTTTTGAAATGCTATTTATGTTTAA

AAGTAATTTAATTTTAAGCATTGATGGTTATTTTATATATGGATGAATTGTTTTTGTTTTAAGTATTTTC

ATTGTCTTTGGTTTAGGTTTTAGTTTTTTTAAGGGTGCTTTTTTTGAAGTTTTTAAATGAAAAAAACCTG

GAATGTCTTTACTTGTTGTTATTTCAACATGTGTTGCTTTCATATATAGTACTTATAGTTTAATTTCTAA

CACAATTATTTATGAACCTAAACTACATGGTTTTTTTGAAACAGCTTGCATGATTATAGCGACAATGAGT

GTTGGTCAATTAGTTAGTGATCGTATTAAACTAAAAGCAAATCAAGATCTACAATCTTTAAATAATTTAC

AAGTCAAAAAATATAATAGTTATGATTTGAACACAAAACAAGTTAGTGAAAAAGTTGTTTTTCAAGCTAA

AATTAACGAATACGCACTAGTTAAAAAAGGTGAAATTGTTCCATTAGATGGCGTATTATATTCACAAATT

GCTGAAGTTGATGAATCATCATTAACTGGTGAAGCTCGTCCAATTCTAAAAACAATTAATAATGATATTA

TTGCTGGATCAATTAATGTTGGAGATAATTTTATTTTTAAAATTACTAAACTTTATAATGATTCAACGAT

TAAAAAAATTATTAATGGTGTTAATCAAATTGCTAGTAGCAAGCCAAAAATTCAAGTTGTTGCTGATAAG

ATTTCGTTATGATTCACTCCATTTATCTTATTAATGGCCATTCTAGCTTTTTTACTACAAGCTTTTGTAC

CAAGTATTCAAGAGTTACCAATTGCTTTTTTAAATCTTCATGGATCAAATAATGATTCTAATTTATATGA

AAAAGCAGCATATGTAGCAGTTTCTGTTCTAGTTATATCATGTCCATGTGCATTTGGTATTGCTGTGCCT

TTAGCAGTTTTAATTGGTGCAGGGCATGGTGCTAAAAGTGGGATTACATTTAACAATAGTAATATTTTTG

AAAAGATTAAAAAAGTTAATGCAATTGCTTTTGATAAAACAGGGACATTAACTTATGGAAAATTACAATT

AAAACAAGTTATAGGAAATGATCAATTTTTAGATTTAATTTATCAAATGGAATTAATTTCTTTACATCCA

CTAGCAAAATCATTCGTAACTTATGCCATTATTAATAATATCGTCATGAGTACAAAATTAATTGATATTA

AAGAAGTTGCAGGAGTGGGGATTATTGCTAAAGATGTTGATGGTAATATTTATGAATTAACATCAGAACA

TTATGCAAATGAAAATCAATTTGATTTTTCATTGATTAATCAAAAATCTTCTACTTCTACTAATTTATTA

GCTTCAAATATTATTTTTAGTATTAACAAAAAGGTGCAATCAATTCTTGTTTTTGAAGATGAAATTCGTG

CTGATGCTTATGAAACAATTAAGGTATTGCATGAAAATAATATTGAAACTTATATGATTACTGGTGATAG

TTTTAATGTTGCTCAAAAAATCGCAAAGGAATTAGGAATTAAACATTTTTATGCTCAAGTTAAACCAGAA

GAAAAAGCTAACATTATTAAAAAAATTCAAAATGATCAAAAAACTGTAATGTATGTTGGCGATGGTATTA

ATGATTTATTAGCTTTAAAACAAGCTAATGTTTCTATATCAATTGGTGAAACTAATAAAGCAACTAATGC

AGTTGCTGATATTAGTTTAATCAAACCAGATATTTTAAATATTTACAAAGTAATTAAACTAACAAAAATT

ACTAAGATGTTTATTGTAAGTAGTTTACTATGAGCGTTTGGTTATAATCTAATTTTTATTCCACTAGCAT

TAATTGGTATTATTCCACCATTTATTTCTGTTTTAATTATGACAACTAGTGATATTGCTGTTGTATTAAA

CTCATTAATTTTTAGATTATTAAAAATGCGTCTAGTTAATAAAAAGCAAATTCATAAACTTAATTTAAAA

ATTATCAATGAGGCTATGTTACATAAATAA

>gi|525938956:546649-548553 Spiroplasma taiwanense CT-1, complete genome112

ATGACTAGAAAAGTTAAAAATAGAATTTTATTTTATTGTTTAATACTACTTTGATTATTAGGTATTTGAA

TTGTAGTATCAATGTTTTTAGCAAAATCAAATAATTGATTTATTATGATAGGTCATAATAAATTCTTTTT

ATTATCAATTGCTATATTTGTTATTTATTTTTTTGGGTGAAAGTATATTCATAGATGTTATTTAGAAATT

TTTAAATGAAAAAGAATTGGAATGAACTTTCTAGTAACTATGTCCACTCAAATTGCACTTTATTTTTCAA

TATACGAAATTGCAGTGGGGTTATTTCCAAATTTAATTGAAGTTTCAATTTTTGTAATTTTATTTTTACA

AACAGGAGATATAATTAATGACAAATTAAAGAAAAAAATTTCAAATGAATTGAGTGAAATTACAAAAATT

CAACCAAAAACAGTAAAAGTCATTTTAAATAATGAAATTATTGAAAAAAATATTAAAACTGTTGAAATAG

GAGAAATTATTTTTATTGAAAAAAATTTTGTAATTCCAATTGATGGAAAATTGATAAATGAAAAAGCTTC

AGTTTTAACCCAAATAATTGATGGTGAAAATGATTCAAAAACTTTTTTTAAAGATCAAAACTTATTTGCT

GGAATGCAAAATTTAAATCAAGATTTAAAAATGGAAGTAAATAATTTATCAAAAGACTCTTTATTAAATC

AAATGATTTCAAAAATATTAAAAATTCAATCTACAAAATCTGGAATGCAAAATTTAGTAGATAAATTATT

AATTTATTTCACACCATTTCTAATTTTATGTTCAATTATTGGATTTTTAGTTTCATATTTTTGATTAAAT

CCAAATAATCTTTATTTTGCAATGCAATCATTTGTAACAATTTTGATTGCAGCCTGTCCTTGTGCAATTG

GTATTGCAATTCCAATAGCAGTTGCTATTGGAGCTTCAAATGCTGGAAAAAAAGGAGTAATTTTTAATAA

ACCTGATGCTTTTCAAAAATTAATTGGAATAAAACATATTGCATTTGATAAAACAGGTACTTTAACTCAA

GGACAAGTAAGTGTTAAAAACTATCAAGGAGAATTCAAATATTTATCAATTATAGGACTTATTGAAAAAA

ACCAAATACATCCACTTGCAACGGGTTTTATGAAATATTTAAAGGAAAAAAATATTGAGCTTAATTCTAA

TAGAATTATTGAAAAACTTGGAGAATTAGAATACAAAATTGATGAAAAAATTTATAAATTAGTTTCATTA

AATGATTTAGATAAAAAATTGGATTCAGATATTAGTATAGATTATTCTTATTTAGAAAACACAACTTCTT

TTTTACTTGAAGAAAATAGGGTAGTTGCTTATATTGAATTTAGTGATGAAATTAGAAAAAATGCACTTAT

TTCCTTAGAAAAATTAAAAAAACAAAATTACAATTTAATAATGATTACAGGTGATAATGAAATTATTGCT

AATAAAGTTGCATCAACTTTAAAAATTGATGAAGTATATTCAAATCAAAAAGCAAATGACAAATATGAAA

TAATAAAAAAATATCAAGAAAAAAATCAAAAAGTGGTTTTTGTGGGTGATGGTGCAAATGATGTACTTGC

AATTCAACAAGCAGATTTGTCAATTTCAATAATATCACCAACAACTTATTTATCATTAGATTCAGATATT

AGTTTAATAAAACCAGACATTGAATTAATTGTAAATGCAATTTACTATACTAAAAAAACTAGAAATATTA

TTTTAACCAATTTATTTTGAGCTTTTGCATATAATTTTACAATAATATCGTTAGCAATGTTTGGATTTAT

AACTCCTTTAGTGGGTATGATTGTAATGTTTTGTTCAACTTTATTAGTTTTATTAAATTCATTGTTATTT

AAAATTTCAAAATAA

>gi|651865246:977671-979821 complete chromosome Acholeplasma palmae113

ATGAGAAAGAGTGTAAAAGTAAGTAATATAACATGTGCACAATGTGCTAAGACAATTGAGAACTACTATA

AAAAACAAGATGGTGTAGATGCCAAAGTATTAGTAAGTGCTAAAAAAGTGATTTTTAATTATGACGAAAG

AAAAACAAACGAGCATACACTGTTAGAAGGACTTCAAAGTATTGGTTACTACCCAGTCATTAATAGTGAA

GATCAAATAAAGGCACTTAGGAAAGACAGAATAGATCTATTTATTGCAGTTATATTTTCTATTCCATTAT

TATGGACTATGTTTGCTCACTTAAACATTCCTCTACCAGTACCTGATATTTTAATGAATGGATATTTTCA

ATGGATCATTACTACCCCAGTTCAATTTTACGCAGGGAGAAGATTTTATAAAGCTGCTTATGAACAACTT

AAGAATAAAGCTTTTGGAATGGATGTTTTAGTAGTTATTGGTACTATGGCTGCCTATATTTACAGTATAT

ACCAAGTATTTATGCACCATGGTACATCACATCACTATGATTTGTACTTTGAAACAAGTGCAGTTATCTT

CTTGATGGTTTTAATAGGTAATTACTTTGAAAGCCGTGTAAAAGAAAAAACAAGTGACTCCTTACAAGCC

TTAATGAGTCTAGGAGCTAAAGAAGCTAGAATAAAAATTAATAATGAAACTCAAACTGTATTAGTTGAAG

AAGTTAAAATAGGCGACATCGTTGTTGTACTAGCAAATGAAAAAATTCCTTTAGATGGAACTGTAGTATC

AGGAACAAGTTATTTAGATGAATCTATGATTACAGGAGAATCAATGCCAGCCTTTAAAGAAATAGGATCA

AAGGTTGTCGGCGCTTCAATGAATTTAACTGAAACCTTAGAAATAAGCGTGAGCGCAACCGGAAGTGATA

CTGTACTTTCTAAAATTATTCAAACGGTTGAGGAAACTGCACTTATCAAACCTAAAGTACAAAGGGTTGC

TGATAGAATCGCAAATTATTTTGTGCCAGCTGTTATTGTAATTAGTATTTTGGTTTTTATTATTCAATTA

ATTATTTCTAAAGACATTAATATTGCCTTTAGCACTTCAATTGCTGTCTTAGTGATTAGTTGTCCGTGTG

CCTTAGGACTAGCAACACCTACAAGCATTTCAGTTGCTAGTGGCATATCATTTAAAAAAGGTATTTTATA

TAAAGGTGGAGAATTCTTTGAAATAGCTCATGAACTAAAAGCAATCGCCTTTGATAAAACTGGAACACTA

ACTAATGGTACACCTAAAGTTGTTGGTTTCTATGGAGATAAAAGCACATATGCATATACGGCATCACTAG

AAGCTCACTCTAATCATCCACTTGCACTTGCAGTACTAGAAGACTTTACAGATGAATACTTAGAAGTAAA

AAGTTTTAATGTGCTTTTAGGGATTGGACTAAACGGAATGATTAATGGAAAAAATATCTATGTGGGGTCT

AAGAAGATTTTAGATCAATTAAAGATTGAACAAACATTTGATACCTACGAAACTTTAAGTTCTGAAGGTA

AAACAGTTATCTTTACAATTGTTGATGGGATACTAGTGAATATGATTGCTATCCAGGATGAACTTAAAGA

AAGTGCTAAGAATTTGATTTTAGAACTTAAAAGAAGAAATATTACACCTTACATGATTACTGGAGATAAT

GAAAAAAACTGCCACCTATATTGCAAGTCAAGTAGGGATTGATAAAGTCTTTTCAGAAGTCCTACCACATC

AAAAAGCAGAGATCATTAAAGATATTCAAAAACAAGGTTTTAAGACTGCGTTTGTTGGGGATGGAATAAA

TGATGCTCCAGCACTTAAAATGGCAGACATTGGGTTTGCAATTGGCAGTGGAAGCGATATAGCAATTGAT

ACATCTGATGTAACCTTAATGTCGTCAAGCTTAGGATTAGTAGTAGATGCAATTGATTTATCGAAAGCAA

CATTAAGGAATATTTATTTGAATTTTTTCTGGGCTTTCATATATAATATAATTATGATTCCAGTTGCGGC

AATGGGTTTTATCAATCCTGCACTTGCTGGAATTGGAATGGGATTTTCAAGTATCGCAGTAGTGCTTAAT

GCACTCAGCTTAAAGTTATTTAAATTTAAATATAAGGAGAATGAAGAATGA

>gi|33241335:c983966-981990 Chlamydophila pneumoniae TW-183, complete genome114

GTGTTCTCCCGTTTGTTTTTCACTTCATTTTCTGCAGAAGTAGTGAATACATTTTTTGAATCAGGGATGT

CTGAAGATACGAGTCCCCTTCTATCTAAACAAAATCGCAAATTGAGTCACAATCTTCCTTTAAAATCGGC

GTATTTATCTTTAGGGACCTACTTGATTGCTCTTCTAAGTTTCTGGCTCCATGCAAAAAATCTCTCTAAC

TTATTTGTAGTATTTACATTTTTTCTTGCTGGCACTCCTGCTCTGATTAAGTCGTTAGATAACATATGTC

AAAAGGTAGTGAACATTGATATTTTGATGACATCAGCAGCCTTCGGCTCTATTTTTATTGGAGGAGCTTT

AGAAGGAGCCTTGCTTCTTGTCTTGTTTGCGATTTCAGAAGCGTTGGGACAGATGGTATCGGGGAAAGCA

AAGAGCACTTTAGTCTCTTTGAAGCAGCTCGCTCCTACTACGGGTTGGCTTGTCTTAGAAGATGGCAACT

TACAAAAAGTCGCAATCAACAAGATTGAGGTTGGAAATATCCTACGCATTAAAAGTGGCGAGGTTGTGCC

TTTGGACGGAGAAATCCTTCATGGTTCTTCTTCTATCAACCTCATGCATCTTACTGGAGAAAAAGTTCCT

AAATCCTGTCATCCAGGGTCGATTGTTCCTGCGGGAGCCCATAATATGGAAGGGAGTTTCGATCTTCGTG

TCTTACGTACGGGATCGGATTCTACCATCGCTCATATTATCAATTTAGTCATTCAGGCACAGAACTCTAA

ACCTAGGTTACAGCAACGCTTAGATAAATATTCTTCAGTATATGCTCTCTCTATTTTTGCAATTGCCTGT

GGAATTGCCCTTTTGGTTCCTTTATTTACTTCCATTCCATTGTTAGGACCTCAGAGTGCATTTTACCGTG

CCCTAGCGTTTCTTATTGCAGCGTCTCCGTGTGCTTTGATCATTGCGATTCCTATTGCCTATTTAAGTGC

TATCAATGCCTGTGCGAAACATGGGGTTCTCCTGAAAGGCGGTGTGATTTTAGATCGTTTAGTTTCTTGT

AATTCTGTAGTGATGGATAAAACGGGAACGTTAACGACTGGCGAGCTTACCTGTATAGGTTGCGACTACT

TCGGTTCTAAAAATGAGACCTTTTTCCCTTCAGTATTAGCTTTAGAGCAGTCTTCATCTCATCCGATTGC

TGAGGCTATTGTTTCTTACCTTATGGAGCAGAAGGTATCCTCGCTTCCTGCAGATCGGTATCTTACAGTT

CCTGGAGAGGGGGGTTCGTGGCTATTTCAATGAACAAGAAGCTTTTGTAGGTCGGGTAGAAACAGGACTAG

GGAAGGTGCCTTCTGAGTATCTTGAAGATATAGAACAAAAAATTTATCAGGCGAAGCAACATGGGGAGAT

TTGTTCTCTAGCGTATGTTGGGAATAGTTTTGCTCTATTTTATTTTCGAGATATTCCTCGTCCTCAAGCT

AAAGAAATCATTCAAGATCTCAAAGATCTCGGCTATCCCGTGAGCATGCTCACGGGAGATCATAAAGTCA

GTGCTGAAAATACAGCGGAAATTTTAGGAATTTCTGAGGTATTTTTCGATCTCACCCCTGAGGATAAGTT

AGCGAAAATACGCGAGCTTGCAACACAACGTCAAATTATGATGGTCGGCGATGGAATTAATGATGCTCCT

GCTTTAGCACAAGCTACTGTAGGCATTGCTATGGGAGAAGCTGGAAGCGCTACAGCAATAGAAGCTGCTG

ATATTGTTCTCCTGCACGACTCCTTGTCGTCCCTCCCTTGGATCATTCAAAAAGCAAAACAAACGAAAAA

AGTTGTTTCGCAGAACCTTGCACTAGCGCTTGCTATTATTCTTTTAGTCTCCTGGCCTGCATCGTTAGGG

ATCATTCCTCTATGGCTCGCGGTAATTCTTCATGAGGGAAGTACGGTGATTGTGGGGTTAAATGCTCTCC

GTTTGCTGAAAAGTTAG

>gi|46445634:136133-138322 Candidatus Protochlamydia amoebophila UWE25 chromosome, complete genome115

ATGGAGAAAAAAAAATTGTTAATTCGAGGGATGCACTGTGTTTCATGCGCGCAAAATCTTGAAAACAGTT

TGAAGAAAGTTTCTGGATTACAAGAGATTCATGTTGATTATTCGACTAGTCAAGCTGATATTATTTTTGA

CGAAAACAAAGTTTCATTTGCCTCACTAGAAAAGCATATAAAAAAAATGGGATATGAGAGTTATTCATTT

AGGCCTTCTTTAGATGAAGATAGACGAATAAAGCAAAAAGAAATTCAAAATACTTTTAAAACCTTTTTGA

TCGCTGCTGTTTTGTCTTTGCCTTTTGTGATTCAGATGTTGGCGATGATGTTAGGATATGCTTTTTCCAT

TCCTCCAACATGGCAATTTTTTTTAGCCACCCTTGTACAATTTGGGTGTGGAGGAACATTTTATCGATCC

TCTTTTTATGCTCTTAAAAACTGGCAAATTAATATGGATGTTTTGGTTGTTTTGGGATCAACTGCGGCTT

ATCTATTTAGCTTTTATGTTTATCTTTTAAAACTTTCTTACTCTTTTTATTTTGAAAGTAGCGCGCTCAT

TATTACATTGATTTTATTTGGACGTTGGTTAGAAGCCGTCACTAAAGGAAAAACCTCTAAAGCACTAGAG

CGTTTATATCATTTACAGCCTAAAATTGCTCGTATTGAAAAAAATGGTGAAATTATAGAGGTTGATGTTG

CCTCGATTCAAGTAGGTGATATTTTTATGGTACGTCCTGGAGATCATATTCCAGTTGATGGATGGGTGAT

AGAAGGAGAGTCTTCCATCAATGAATCTTTGTTAACGGGAGAAAGTTTGCCGGTTTATAAGAAACAGGGA

TCGAAACTTTATGCAGCGACAATCAATGAAACTGGTTACTTGAAAGGGCAGGCCTCTTCAGTTGGATCCG

ATACAATTTTAGCTCAGATACTTCGAGCTGTAGAAAAAGCTCAATCTTCGAAAGCACCTATTCAAAAATT

TGTGGATAAAATTTCTGCAGTTTTCGTTCCCTGCGTACTTCTAATCAGTTGTTTGACGATATTAATTTGG

ACATTTCTAATAGGTGATTTTACAAATGGATTGATTAATGCGGTCTCTGTTCTAGTGATTGCATGCCCTT

GTGTTTTAGGATTAGCCACGCCGGCAGTTCTTGTGGTAGCAAGTGGGGTTGGAGCACAATACGGAATATT

TTTTAAACAAGCTTCATCGATCGAGCAGTCTGAAAAAATCAAATTTTTATTTTTAGATAAGACCGGTACC

TTAACGAAAGGAATTCCTTCTGTGATGGGAATCTATCCAAATGCCAAATATCTTAAAAATGACGTATTAC

AAATAGCAGCTTCGCTTGAAAATTGCTCTTCCCATCCTTTAGCTAAGGCTATATTAAATGAGGCCAAAGA

AGAGGGAATTCTTTTAAAAATTGTCAAAGAATTTAATTCATTTATTGGAAAAGGAGTTACAGGAACAATT

GATGAAAAGGTTTATTATTTAGGCTCTGCTAACTTTGCAAAAGAAATGGGAATTGAAATCGACCCTGCTT

TTTCTCTTCTTGAAGAACAAGGAGAATCACTGGTCTTTGTTTGGACAGACCATCAATTATTAGGCATGAT

TACCATCATGGATGAGCTAAGAAAGGAAAGTATGGAAGTAATTCGTTTGTTGAAATTGAAAGGAATTTAT

CCCATCCTTTTAACTGGAGATCGAAAATATGTTGCTGAAACTATTGCAAAAAAATTGGGGATTGAAGATG

CGCGGTTTGAATTGCTTCCAGACGATAAAATGCAGATTATCCGAGAAGCTAAAAATAGTGGAAAAGTAGG

AATGATTGGAGACGGAATTAACGATGCTCCAGCCCTTGCAGAAGCTGATGTGAGTTTTGCTCTTGCCTTT

GGTAGCGATATTGCTATTGAAGTCGCTGATATTACTTTAATACGAAATGATTTAATGAGTTTAGTGGACG

CTATAGATCTTTCCCAGCAAACCATGCGTAAAATGAAATATAATTTATTTTTAGCATTTATTTATAATGT

TCTTGCGATTCCTTTGGCAGCTTTTGGACTACTTAATCCAATGATTGCGGCAGGAACAATGGCAATGAGC

TCTGTTTCTGTGATCGCCAATGCGTTATTACTACGTTATTGGAAGCCAGAAATAAATAGAAATAATGTGG

AGAGGATTGTCAAAGTTTAA

>gi|338731896:106931-109072 Simkania negevensis Z chromosome gsn.131, complete sequence116

ATGGCTCATTTTGATTTACATATTCGGAATATGAATTGCTCTTCTTGCATTGCGAAAATTGAAGGGCGTG

TGACGAAGATTGAAGGAACAATCAGTTGCTCTGTTAATTTTGCAACAGGACAAGCCCGAGTCGAATATGA

AGAAGGAAACGACATCAAACAAACGGTTGCCGATGCCATTACTGAATTGGGGTATCCAGCAACAGTCATT

GAAGAAGGGCAGGTTTACGACGAAGAAAAGGATCGCTCTTTTTTTTGGCTCAAAGTTCGCACCATTTTTG

CTATTGTTTTAACCCTTCCTCTCACCGTTCCAATGTTGGGGGAATTTTTTGGAATCGACGTTTTGTTTCC

CCATTGGATTCAGCTCATTTTGGCAACAATCGTTCAGTTTGGCGCAGGCTACTCTTTTTACATTGGATCT

TGGAAGGCGATTAAAAATAAGGCGGCCAACATGGATGTTCTTGTTGCACTGGGGACAACAGCCGCCTTTT

TGTTTAGTGCAGTTGTCGTCCTTTTTCATGTTCAGGGTTACCTCTACTTTGAAACGAGTACGGTATTAAT

TTCACTGATATTACTCGGACGAGTTATTGAACAGCACTCTAAACACAACGCTCAAGGGGGAATGAAAGCG

TTGCTCAAAATGCAGGCCAAATCGGCCCGCATTAAAAAGGGAACTGAGGTTGTTGAAGTTCCAATTGATG

CAGTGCACAAAGGAGATATTGTAGTTGTCAAGCCAGGAGAACGGATTCCAGTCGATGGAGAAGTTGTTGA

CGGAGAGTCTCATGTCGATGAATCGATGCTAACAGGAGAAAGCATGCCTGTGCGAAAAGACAAAGGGGAA

AACGCTTATGCCGGAACAATCAATGGGGAAGGCATGATCGAAGTCCAATCCTCTCGTTTGGGAAAAGAAA

CCAGTTTGGGCAACATCATTCGTCTTGTGGAAGAAGCTCAACGTTCAAAAGCCCCTATCCAAAAATTGGC

CGATCGGATTTCAGGGGTGTTTGTGCCGATTGTTTTAGGGATTGCGATTCTCACTTTTTTCTTGTGGGGG

TTTGTTGCAGGTGACTGGCAAGAGGGACTGATCAGCGGCATTGCTGTTCTCGTTATTGCATGCCCTTGCG

CCCTTGGCTTGGCCACTCCGACTGTGATTATGGTCGCATGTGGTCGGGGTGCACGTGAAGGCATCTTAAT

CAAAGATGCTGTCGGCTTGGAAAAGGCTGGAAAACTTGATGCCATTATCGTCGATAAGACAGGAACAGTG

ACTGAGGGAAAACTTTCTGTCGATGAAATCACGTCTGAAGATGACAACCTTTTGCGAAAAGCAGTTAGCT

TGACAACCCACTCTGATCACCCCATTTCAAAAGCCATTACAAATCATGCCAAAGATCAGGGGGTGCAAAT

TCAAAGATGCGAAGCTTTCCATTCTCATACTGGGCAAGGACTCAGCGGCAGTTTTCAGGGAAAGGCTTAC

TTACTTGGCTCGGTTCGCTTTATGAAGCAGCAAAACATCGATCTTGGCCCATTCAATGATAAGTTGCAGA

CCGATGTCCGTGTGATTGCAGCCATTGCGGAAGAAGGAAAGTGCATTGGATTCATCGCACTTGCCGATAA

ACTGAAAGAGGGAAGCCAAGAAGCGGTTGCAAAACTTCATCAGATGAAGAAAGAAGTCTATTTACTCAGC

GGAGATCGGAAAGCTGTGGTGGAATCTGTTGGAAAGATGCTGAATGTCGATGGCTTGTTTGCAGAAGTCT

TACCCGAAGAGAAGGCCTCATATGTCAAGAGACTTCAAGATGAAAAGAAGGTCACGGGAATGGTCGGTGA

TGGGGTGAATGATGCTCCTGCCCTTGCTGTTGCCGATGTAGGCTTTGCGATTGCTGATGGTACAGATGTT

GCGATGGAAAGTGCCTCTATTGGACTCATGCACAGTAGCCTCTTGAATCTTCTGACAGCGCTATCTCTTT

CAAAGGTGACCTTTTTGAAAATTCGGCAAAATCTCTTCTTTGCCTTTGTCTACAACTGTGTGGGCATTCC

TTTAGCAGCCTTTGGGCTGCTCAATCCTATGATTGCCGGTGCCGCCATGGCAATGAGCTCGATCTCAGTG

GTTCTTAACTCTCTTACACTTCAGCGTAAAAAGCTTTTTTAA

>gi|225871699:c765048-763015 Acidobacterium capsulatum ATCC 51196 chromosome, complete genome117

ATGTCCGCTGCTGACCGATCCATCCCTGAACTCGTCCCCATCTCGCGCCGCCGCTTCGTGCAGGGCATCG

CCGCCGGAGCCGCCATCGCCGCTCTCGACGGGCACGCTCTGCCCACCTTCGCGGAAGCCGCCCCGTATCA

TCCGCCTGTCCTCGCCGGAACGCACTTCGACCTCACCATCGACACGCTGCCCGTCAACTTCACCGGCCGC

CGCGCCCGCGCCCTGGCCGTCAACGGCTCGGTGCCTGGCCCTACGCTGCGCTGGCGCCAGGGCGACACCG

TGAGCATCGCCGTCACTAATCGCATGCGCGTGCCCACCTCCGTGCACTGGCATTCCTTGCGTCTGCCCAA

CCCCATGGATGGCGTCCCCGGCCTCACCTATCGCGGCATCATGCCCGGCGAAACCTTCCACTACCGCTTT

CCCGTGCTCCAGAACGGCACCGCCTGGTACCACAGCCACACGCGCTTTCAAGAGCAGAGCGGCCTCAGCG

GAGCCATCATCATCGATCCGCGCGGCACAGACCCCGTCCAGGCCGATCGCGATTACGTCATCTTTCTCTC

CGATTGGACAGATACCAATCCCGAAACCGTCTTCAGCAACCTCAAAGAGAACAGTGACTATTACAATTAC

GAGCGCATCACCGCCGGCGGCTTCTTCCATCAGGCAAAGCAGCAGGGCCTCGGCAAAGCCATCTCCGCAC

GCCTCGCCTGGGCGCGCATGAACATGAGCCCCAGTGACATCTCTGACGTCTCCGGCGCCACCTACACCTA

TCTGCTCAACGGCAACAGCCCCGCCCGCAACTGGACCGGCCTCTTCCAGCGCGGCGAAAAGCTCCGCCTG

CGCTTCATCAACGGCTCGTCCATGACCTTCTTTGACGTCCGCATCCCAGGCCTCAAAATGACCGTCGTGC

AGGCCGACGGCAACTACGTCGAGCCGGTGCCCGTCGATGAATTCCGCATCGGCGTTGCCGAGCGCTACGA

CGTGCTCATCGAGCCGGAGGACAACCGCGCCTACACCATCTTTGCCCAGTCGGAAGATCGCAGCGGCTAC

GCGCGCGGCACCCTCGCCCCACGCCCCGGCATGACCGCTCCCGTTCCGCCCATGGACCCCGTCCCTCGCC

GCACCATGGCCGACATGGGCATGGCCATGAAAATGAACATGAGCGGCATGGCCTCCATGGCCGGCATCGA

CGGCATGACCGCCAAAGACGCTCAAGGCATGGACGTCCAGAAAATGAACGCCCAGAAAATCAACCAAGAC

ATGCCCGGAATGAACATGCCCGGTGGAAGCATGTCTGAGATGAACATGCGCAGCGAAATGTCCGGCATGA

AGATGCGCGGCAACAGTATGCCTGGCATGGCAATGTCCGCCATGACGGCTGAAGAATCCCCGGAACCTCC

GCCCATTCCCGGCAGCTTCGGCAAGACACCCTTTCCGCAGCCCTGGACCGACACCCATCCTGCCGCAAAC

GTGCCTGAAAGCGCCATCCACGCGGCTGAGGCAAAGCTCCAGCCCGCCGATCCCGTTCATCTGCATCCCG

GCCCGCAGGTCGCCGAGGTCGCCCGCCACGTCGAGAGCCGCCTCAACCAACCCGGTGACGGACTCAACCA

CAACGGCCGCCGCGTGCTCACCTACGCCGATCTGCGCGCCCGTTTTCGCGGCGTCGATGACCGCCCGCCC

ACGCGCGAAATCGAGCTGCACCTCACCGGCAACATGAACCGCTACATCTGGGGCTTCAACGGAGAAAAAT

TCAGCCAGGCCGAGCCCATCGTCCTGCGTCTCGGCGAGCGTGTTCGCTTCGTGCTCATCAACGACACCAT

GATGGAGCACCCCATCCACCTCCACGGCCTCTGGAGCGAGCTCGAAAACGGCCACGGCCCCTACCGCCCC

TTCAAAGACACCATCATCGTCAAGCCCGCCGAGCGCGTCAGCTACCTCGTCAGTGCCGACACGCCCGGCC

GCTGGGCCTACCACTGCCACCTGCTCTACCACATGGAGGCAGGCATGTTCCGCACCGTGGTGGTGCTCCC

GTGA

>gi|94967031:c2545752-2544004 Candidatus Koribacter versatilis Ellin345 chromosome, complete genome_118

ATGTTCCGTTGGCTGTCTTCCCTTTTCGCCTTCACATTCCTTCTCTATCCCGCGCTGGCTCGTGCTGCCA

CCGAGGCCTGCCCTCGTCCCGCGCTAGGTAGCGTGGTGCAGGAGCCGCCTGATCTCTTTAGCCATAACGG

CGTTCTTGAATTGACTCTGACGGCACGCAACGCTGCTGCCACCAACGGCTCCGCGCGGTACTGCTTCATC

GATGCCAGCGGCAACGAGTCGCCCACACTGCGCGTGAAGCCCGGCGATCTGGTTACCATCCATCTGAAGA

ACGAACTGACGAACATCACGCCGGATAAAACATCGCCACACCAGCACCATGCCTCCGCCTCGACCGGTCC

TTGTACCGGAGGGGCAATGACGCCGGTGACCACGAATCTCCATTTTCATGGGCTGACCATCCCGCCCACG

TGCCACCAGGATGAGGTGCTGCAGACACTGATCCACCCCGGTGATCCGGTCTTCGATTACAGCTTTCGCA

TTCCCGACGACGAGCCTCCCGGGCTCTACTGGTATCACCCGCACGTGCACGGCTTCAGCCGCGTCGAACT

GCTGGGTGGGGCCTCGGGCGCATTGATTGTGGAAGGCACTGAGCGTGCCGATCGCGCTGTGGCGGGACTA

CCCGAGCGAGTCTTCGTGATTCGCGACCAGGATCTGGTCAATCCCAATGCACCGTCCGCGAAATCGGAAC

CGGTGATTCCGAAATTCCTGCTCGACAACGATGGCGATGCGGTGAATACCGGCACCGGCTTCGGCAAGCC

CGCCAAAGATCTCTCGATCAACTTCGTCACGGTGCCTTACCCGAACTACGAACCAGCAACCATTCTCATG

AAGCCGGGGGAACGCCAGCTTTGGCGAGTGCTCAACGCTTCGGCGATCACCTATCTCAATCTCTCGATTT

TGCGTAACCGCGCAGCGCAGCGCGTGGGATTGATCGCGATTGATGGAGTGCCCCTGAATCTTAACGGAGG

TCCTGCCGACTTTGTCGATTGGCAAACTCACATCGGATTGCCGCCCGGAAGCCGGGCTGAGTTCATCGTC

GAAGCGCCGGAGGCTGGCGTCAGCGAGGCCTTCGTCACACATACTGTGGACACAGGCCCAGGCGGCGAGA

ACGATCCCAATCGGCCGCTGGCGATCATTCGATCTTCAGCCGATGCGCCCGAGCCGCGCGTAAAGCTAGA

TGCTGCGCCGCAACCGCTTCCGGCCCCCAAACAACCATGGCTCGGCAGCGTGGCGCCGACGCAGGTCCGC

AAGTTCTACTTTTCGGAAAAGCTCGACGACCCTAACAATCCCGCGAGCGCCAGTGAGTTCTACATTACGG

AGGAAGGCCACACGCCACGCGCCTTCGATCCAAACTCCAAGCAGCCCGATGTCACCGTGACGCAAGGCAC

TGTCGAAGACTGGATCATCGAGAACCGCTCCTCTGAACTTCACGCGTTCCACATTCACCAGTTGCATTTC

ATGCTGCTTGATTACGACGGCAAGACCGTGAACGAGCCGTTCCTGCGCGACATCGTAAACGTGCCGTATT

TCAACGGACACACACTGTCGTATCCCAGCATTCGCATTCGCATGGATTTTCGCGATCCGAACATCGTGGG

AACATTCGCGTTTCATTGCCATCTCCTCGAGCATGAAGATCGCGGAATGATGGCAACGATTCAAGTTGTG

CCGAGCGAGACTACGAGAAATACCCCGTCAAAAGCGGCCTTAGACGCACCAAAAGGTGTGGCGCGGTAG

>gi|374308961:2638157-2639833 Granulicella mallensis MP5ACTX8 chromosome, complete genome_119

GTGCTCGCCACAGCCGCCTGCGTGACTTTTTCCCTGGAGGCGCAAACTGCGTTATGTCGCCCGCCTGCAC

CCGGAAGCGTCGTCGCCGAGCCGGAGCAGGTGCGCAGCCGTAATGGACTCCTGGAGTTCGACCTGAACTT

CAAAAACATGCAGGATGCCGACGGCCAGGAGGAGTACTGCTATCAATCGTCCAACGGCGGTCGATCTCCC

ACGTTGAGGGTTCAGCCGGGAGACAAGCTCGTTCTCCATCTCAAGAACTCGCTGAAGCTCGAGCCAGGCG

CCGTGAATCCGATGCCGCACTCCGTGGGCATGCCGATCGAATCACCGTGCGCGGACGCGCAGATGACGGC

ATCCTCGACGAACCTGCATTTCCACGGAATGAACGTTCCTGCTTCTTGCGGTCAGGATGATGTCATGCAC

ACGGCGATCTCACCGAAGAACTCGCCGTTCACCTATCACTTGACGATTCCGGTCGACCAGCCCCCGGGAT

TGTACTGGTATCACCCGCATCTCCATGGGTTGACGAATGCGCAGGTGTCCGGCGGAGGTTCGGGCGCAAT

TGTGGTCGAAGGCATCGAGCGCGCGAACCGGCTGGTAGCGGGCATGCATGAACGCACCCTCGTGATTCGC

GACCAGTACCTCATCCATCCCAACTCGCAACAGACTGGGCCGGGACTTACGCCGCCCGTCTTTCGCGATG

CTGAAGGGGACATTCTTAATACGGGGACGGGAGGAGGGAAGCCGTCGCGCGATCTCTCCATCAACTTTGT

TCCAGTACCCTGGCCGGACTATCCGCCTGCGAAGATTCTCAGCAGGCCGAACGACCGGCAGTTGTGGCGC

GTCCTCAACGCCTCGTCGCTTACGTTCCTGGATCTGCAGATCGTCGTGGCCGGCAAACCACAGTCGATTG

GGGTAGTGTCGCTCGATGGAGTGCCCATCGACGAGAACGGGCTTACGCCGGACCGCGTTTTTTGGACGGA

CCATATCTTTCTGCCTCCCGCTGCACGAGCGGAGTTCATCGTTAAGGGGATGCCCGCAGGCACACAAGCG

CAGTTCATTACGCGCAGCGTGGATACGGGGCCTGCCGGTGAGAACGATCCCGTGCGCCCTCTTGCAGATG

TCGTCGTTACAGACCAGGCGCCGGATTTGCTCGCGCTGCCGTCCTCAGCGGATCCGAGGTCTCGCCCGAG

TCAGATATGGATCGGCAACGTCCAACCCGCACGGACGCGCAAGCTCTACTTCTCTGAGCAGCCGTCCGAC

CCGAACCGGCCGGACAGCCCAACGGTCTTCATGCTCACCGTGGAAGGCAAGCCGGCTGCTCCGTTCAATC

CGCACCAGACCGAGCCTGATATCGTCGCGCGGCAGGGAGACGTCGAGGACTGGGTCATCGAAAATCGTTC

TACGGAAGCGCATGCCTTCCACATTCACCAGGTCCACTTCATGCTCACGCAATGGAACGGCGTTGCCGTT

GATGAACCTTTTCTGCGAGACACCATCAACGTTCCGTACTGGCGAGGCCCCGGCACACCGTACCCGAGCA

TCACCCTACGCATGGACTTTCGAGATCCGAATGTAGTGGGAACCTTCATGTATCACTGTCATCTCCTTGA

ACATGAAGATGGAGGGATGATGGGGGTGTTGCGCGTTTTGCCTCGAATGGATGACAGAACAAATTGA

>gi|383760955:c2651622-2649541 Caldilinea aerophila DSM 14535 = NBRC 104270, complete genome_120

ATGGCTGAACTGCAAACGCTCGAAGTTCCTGTCAAAGGCATGGACTGTGCGGAATGCACGCTGCACGTTC

AACAGGCCATCGCCAGACTGCCAGGCGTGGAGTCGGTCAACGTTCTGCTTACTACCGAAAAAGCCGTCAT

TCAACTCGACCCTGCTCGCGTCGACTTGTCCGCTATTCGTAAAGCCGTAGAGAGCGCGGGTTATTCCGTT

CCTGACTCGGCCACGCCGCTGGCTACTTCTATGGACAGCTTCAATCGCCGGATGACGGTATTGCTTGCCA

TCGTGTTTAGCGTTGTTCTGAGTATCGTGATTGCCGGCGAATGGCTTGGCCTGTTCGATGAACTGAATGA

ACTTGTCCCCCTTCCCATTGGCACTGCCCTTGTCATTGTAGGCGGCTTTCCGATCTTCCGGAATGTTGTA

CGAGCTACTCTAAAGCGGCAAATCACCTCGCATACACTGATGACAGTTGGAGCGATTGCGGCCCTCATCG

TTGGTGAATGGGTAGCAGCGGCGATTGTTGTGGTGTTCATGCGTGTGGGCGATTATGTCGAACGCTTTAC

CACGGAAAGCGCCCGCCGCGCCGTGAAAGAACTCACGGCAATGGCGCCACAAACCGCACGCGTGGAACGA

GACGGTATAGAAGTGGAAGTGCCGGTAGCTGAAGTGAGCGTTGGCGAAATCGTCATCGTTCGCCCCGGCG

AGAAGATTCCGGTGGATGGCGAAGTCATCGCCGGACATGCCACGATTGATCAGGCAACCATCACCGGCGA

ATCTATGCCCGTTGAAGTGGCAAAGGGTTCGCATGTGTTCGCTGCTACCATTGCGAAGCTGGGCAGTCTG

CGCGTGAAAACGCTCCGCACCGGCACAGATACAACGTTTGGACGCGTGATCAAGATGGTGGAAGAAGCCG

AAGCCCATCGCGGCGATGTGCAACGCCTTGCCGACAAGTTCAGCGCATACTATCTGCCGATTGTCGTTGG

AATTGCTGCGCTCACCTTTCTCATCAGCGGCAATGCCCTGGCTACGGCTGCCGTTCTCGTGGTGGCCTGT

TCCTGCTCCTTTGCTTTGGCCACGCCCGTCGCCATGCTGGCGTCCATAGGCGCAAGCGCAAAGCACGGCC

TGCTGATTAAAGGCGGCAAGTATCTTGAAGCGTTGGTGCGCGCGGATGTGGTGCTGGTAGATAAAACCGG

CACGCTCACGTTGGGTCAACCGCAAATCACCGAAGTGGTTCCGCTCAACGGTATGCCCTCCTCTGAAATT

CTTGCCCTTGCGGCTTCTGCTGAACGTTATTCGGAACATCCCCTGGCTGAAGCCGTACGCGCCGCCGCCC

GCGCTCAAAACCTACCGCTCGCCGAACCGCAAGATTTTGAAGCCCTGCCCGGCATGGGCGTTCGGGCATG

GGTGAATGGCTACACAGTTGCCGTCGGAAACCGTCGTCTGATCCCGGCGACAGAATCCTTGCCGATCGCT

CAACAACTGGAAGCGCAGGGTAAAACGCTCCTGTGGGTTGCCCTGGATGGTGAACCTGCCGCCATTCTTG

CTGCCGCCGATGCGCTCCGTCATGAAGTGCCCGCCGCTTTTGCCGAACTGCGTAACCTTGGCATCAAGCA

TGTGGAACTGCTCACAGGTGATCATGAACGCACCGCGGCCGCGCTTGCTGCAAGTCTCGGTATCCAATAT

CGCGCCCACCTCCTGCCCGAACATAAGATTGACGTTGTAAAGGACTATCAAGCCAAAGGACATACGGTTA

TCATGGTAGGAGATGGCGTAAACGACGCCCCGGCGCTGGCGCAGGCGAATGTTGGCATCGCCATGGGCGC

GGCAGGTACAGACATCGCCATTGAAGCGGCGCATATAGCCCTGATGCGTGAAGACTGGGCGCTGATCCCC

GAAGTGTTACGAATCGCCCGCCGCACAATGGGCGTAGTCAAGATGAACATCGCCTTTACAGCGCTGTATA

ACCTGTTCGGCCTGACGCTGGCTGCCGTGGGCATCTTACCGCCTACCCTGGCAGCGGCAGCCCAGTCCTT

GCCAGATTTGGGCATCCTTGCCAATTCGGCGCGGCTGCTGCGACAAAGGTAA

>gi|561112193:775989-778475 Dehalococcoides mccartyi GY50, complete genome_121

ATGTCTGAAAATAAGCGGCCGGATGCTCTGCCCAAGATAATTATAGGTATAAGCGGCATGTCATGTACCA

GATGTGCCGCCAGCATCGAAGCGCGTTTATCAAAAACGGCTGGTGTAATAAACCCTAAACTTAATTTTGC

CTCCGGTAAGCTCATTTTTAGTTATGATCCGTCTGTTATCAATCTGTCAGATATAAAGGCCATTATTACC

GATTTAGGTTACGGTATTATCAGTAATAAATCTATTTTCCCCATAAAAGGCCTTCATTGTACGTCCTGTG

CAGCCAGAGCCGAAAAGGCTCTGATAAATACAAGCGGTGTAATTAATGCAGGTGTTAATCTGGCCAACCA

GACTGCCAGCATAGAATATCTGGATTATATTTCTTTCCGCGAACTCAGCCAGTCCATAAGCAATATCGGC

TATGAACTATTGCCCGAAGAAACACCTCGGGATGAAATTTCTCACTCAGAAGCTACTGAAACCCGGCAAT

TAAAACGGGAACTTTCAGTAGCTTTGGTGCTGGGAATAAGCCTTATGATTATAGGATTCTTGCCAGCTTT

TGGCGGCAAGGAATTTATTATGTTTTTGCTGGCTACGCCAGTGCAATTCTGGGCGGGACTGCGTTTTTAC

CGCGGGGCATTTGCCGCCTTGAAAAACCGCACCACAGATATGAATACCCTTATAGCGCTGGGTACTTCCG

CTGCCTACTTGTATAGTATGATAGCTTTGGTGTTCCCGCCTTTATTTGATTCACCTTTGCTGGAAAAACA

CTTGTATTTTGATACATCCGCTATGATTATCGCCCTTATCCTGACCGGACGTTTTCTGGAATCCCGTGCT

AGAGGCCGTACTTCTGATGCCATTCGCCGTTTGATTGGTCTTCAGCCAAGTACAGCCTCAATTATAAAAG

ACGGCAAAGAAATTTTGGTAAGTATAAACGAAGTAATGGCAGGCGACAAAATTATTATTCGCCCGGGTGA

AAGACTACCCGTGGACGGGCTGATACTGGAAGGTTATTCCAGCCTGGACGAATCTATGGTAACCGGCGAA

AGTATTCCGGCTGAAAAAAAAGCGGGGGATAATGTTATCGGCGGTACGTTTAACCAAACAGGTGCGTTCA

CTTACGAAGCTCAAAAGGTGGGAGCGGATACCGCACTTGCCCGTATAATACGGCTGGTTGAGGAAGCTCA

GGGAAGCAAAGCTCCCATTCAGCGTTTGGCGGATAAAATAGCCTCGGTATTTGTGCCTGTAGTTATAGGA

ATCTCCGTTTTAACTTTTATTTTCTGGCTTTTTTTCGGGCCGGAACCTGCCTTCACTTATGCTTTTTTAA

ACCTTATTACAGTATTGATTATAGCCTGCCCGTGCGCATTAGGTTTGGCCACACCTACAGCCCTTATTGT

CGGTATGGGCAAAGGAGCTGAGAACGGCATACTTATCCGCAGCGCAGTAGCTCTGGAGAAAATACACAAA

CTGGATACTATAGTACTGGATAAAACAGGCACACTGACACGCGGCAAACCGGTGTTGAGCAATCTGGTAA

GCCACCGTTTTGATAAAGATGCCTTTTTAACTCTGGTTGCCTCTGCCGAACAGTTTTCCGAACACCCTTT

AGCCAAAGCTGTTTTAAAAGAGGCCGGGCGTAAAAAACTTGCACTTACAAATCCTGCGGAATTTTCAGCT

TTGCCCGGTGCAGGGTTAAAAGCTACAGTTAACAGCGAGCAGATACTCATAGGAAACACAACCCTGATGC

AGTCTCATAATATATCTCTGGCCGAATACCAATCTGAAGCTGACAAACTTTGGGAAGCGGGTGAAAGCCT

GATTTTTGTGGCGGCAAACGGGAAACTCGAGGGTATTGTAGCCGTAAGGGATATATTGAAACGGGAATCA

GCTGCCGTTATTTCCGAACTAAAAGCACATAAACTTAATACAATCATGCTAACAGGTGATAATCACCGGG

CAGCCAAGCGCATTGCAGATGAACTGGGTATAGACCAATACGTAGCTGAAATAAAACCCGAAGACAAATC

CAAACTTATACAAGATCTGCAAAACAAAGGCCGTTTAGTAGCTATGGTGGGAGACGGCATAAATGATGCT

CCGGCACTTGCCAAGGCTGATGTGGGTATCGCCATTGGCACAGGCACCGATATAGCCATGGAAACCGGTG

ATATTACCCTTATTTCCGGTGACCTTTTTGGCATCACCAAAGCCATCCTGCTCAGCAGGGCGACTTTTAA

TACCATCCGCCAAAACCTCTTCTGGGCATTTTTCTACAATATCATCCTTATACCCGTAGCTGCAGGTGTA

CTTTATCTGTTTTTCAGCCAGAACGGTGTTCCTCAATCACTGCATTTCTTTCTGGGTGAATACGGTTTTC

TAAACCCTATACTGGCAGCTCTAGCTATGGCTATAAGTTCTCTGACTGTAGTTGGCAATTCCCTCCGTTT

GCGCGGTATAAAACTAAGTACCTATCTAAAGCATTAA

>gi|221635406:216088-217638 Thermomicrobium roseum DSM 5159 plasmid unnamed, complete sequence_122

ATGATGCGCTTTGCTCCATTATTGCTGGCCCGGCGCTGGACCCGCCGTGGCCTGTTTCCGGTGATGACTG

GGAGCGTGCTCCTGGCGGTGGCCTGCAGTCGGCGACCGGTCGCACCCACCCCGAGTCCGCAAACGGCTGC

GGAGCCGACGGTCACGGTGCTGCCGACTCGATCAAGCGGATCAGCAGCCGGTCCGATCCGGGAGTACACC

ATCGAGGCGGTGGAGGCGGAAATCGAACTCGCTGGTCGGCGGGTGCGCACGTTCACCTATGGAGGTCAGG

TTCCGGGTCCGGAGCTGCGTGTTCGGGAGGGCGAGACGCTGCGCGTCCTGCTGGAGAACCGGCTCCCTGA

GCCGACGACGATCCACTGGCACGGTATCCCGGTCCCCAATGCGATGGACGGGGTGCCGGAGGTCACGCAG

CCAGCGGTTCCGCCTGGAGAGCGCTACCTCTACGAGTTCCCTGTGCCGGTGGCTGGCACGTACTTTTACC

ACACCCATGTTGGTGTCCAGCTCGATCGGGGGTTGGTGGGTGCACTCATCGTCGAGCCAGGGCACGAGGA

ACTAGCCTATGACCGCGAGGTGGTGCTGGTGCTCGACGACTGGCTGGATGGGATCGCCGGGACACCGGAG

GAAGTGCTGCGGCAGCTGGTCGGTGCTGGCCACGGTGGACACGGCATGGGACATGGGGCGATGCCGGGTG

GAAGCATGCCTGGCATGGGAACGATGCCCGGAATGACCGGAACGAGCGGTGAGCCGCCCGAGGTCGAACC

GGATCTCGTCTATCCGTTGTACGTGGTCAATGGGCGTCCACTGGAGCATCCCTTCGAGTTCGTCGGCAAG

CGCGGGGATCTCCTCCGGCTGCGGCTCGTCAACGCGGCATCAGCGATTATCTTCCGCGTCGCGCTCGCTG

GACATCGCTTCACCGTCGTGCAGGCCGATGGACAGCCGATCGAGCCGCTCGAAGGGGACGCTCTGCGGAT

CGGGATGGGTGAGCGGTACGATCTCCTCGTCCGCTTGGACAATCCTGGCATCTGGCCGCTCGTGGCCTGG

GCCGAAGGGACGGATCGTGCAGCGCAGGCGGTACTGCGCTACGAGGGGGCGAGCGGCCAAGTGCCGGCGA

GGGTGACGCGACCGCGGGAATTGTCGGGGGAGCTCGTGCACGGGCTGCGCTGTCGGGCGCGCTCGGCGGG

GACGGAGTTCGTTCCCGATGTCGAGCGGCCGATCGTGCTCGGTGGCGGGATGGGTCAGTATGTCTGGACG

ATCGATGGGCAAGCCTATCCTGCTGCGGAGCCGATCCGGGTGCGGCGAGGCCAGCGGGTACGCTTCCGGT

TGCAGAACATGACGACGATGCCGCATCCCATGCACCTGCACGGTCACTTCTTCCGCGTGGGCGAGAGCGC

GACGGCCCCGGTGCGGGATACCGTGCTCGTCGATCCGATGCGGGAAGTAGTGATGGACTGGATCGCGGAG

AACCCGGGTCGGTGGGCGTTCCACTGCCATCACCTCTACCACCAGGAAACCGGAATGATGCGGATCGTCG

AGGTCACCTGA
